# Do the CONSORT and STRICTA Checklists Improve the Reporting Quality of Acupuncture and Moxibustion Randomized Controlled Trials Published in Chinese Journals? A Systematic Review and Analysis of Trends

**DOI:** 10.1371/journal.pone.0147244

**Published:** 2016-01-25

**Authors:** Bin Ma, Zhi-min Chen, Jia-ke Xu, Ya-nan Wang, Kuang-yang Chen, Fa-yong Ke, Jun-qiang Niu, Li Li, Cheng-ben Huang, Jian-xun Zheng, Jia-hui Yang, Qian-ge Zhu, Ya-ping Wang

**Affiliations:** 1 Evidence-Based Medicine Center, Institute of Traditional Chinese and Western Medicine, School of Basic Medical Sciences, Lanzhou University, Lanzhou, Gansu, China; 2 Traditional Chinese Medicine Ache Department, the First Hospital of Lanzhou University, Lanzhou, Gansu, China; 3 Interventional Latrology Department, the First Hospital of Lanzhou University, Lanzhou, Gansu, China; 4 Key Laboratory of Evidence Based Medicine and Knowledge Translation of Gansu Province, Lanzhou, Gansu, China; Stavanger University Hospital, NORWAY

## Abstract

**Background:**

We investigated whether there had been an improvement in the quality of reporting for randomised controlled trials of acupuncture and moxibustion published in Chinese journals. We compared the compliance rate for the quality of reporting following the publication of both the STRICTA and CONSORT recommendations in China.

**Methods:**

Four Chinese databases were searched for RCTs of acupuncture from January 1978 through to December 2012. The CONSORT and STRICTA checklists were used to assess the quality of reporting. Data were collected using a standardised form. All included RCTs were divided into three distinct time periods based on the time that CONSORT and STRICTA were introduced in China, respectively. Pearson's χ2 test and/or Fisher's exact test were used to assess differences in reporting among three groups.

**Principal Findings:**

A total of 1978 RCTs were identified. Although the percentage of all the items has increased over time with the introduction of CONSORT and STRICTA in China, the actual compliance in several important methodological components, including sample size calculation (0% vs. 0% vs. 1.2%, for pre-CONSORT and pre-STRICTA, post-CONSORT but pre-STRICTA, and post-CONSORT and post-STRICTA, respectively), randomisation sequence generation (1.4% vs. 15% vs. 26.3%) and implementation (0% vs. 0% vs. 1.3%), allocation concealment (0% vs. 1.4% vs. 4.9%), and blinding (0% vs. 5.7% vs. 9.1%), remains low. Moreover, no RCTs have reported the setting and context of treatment and no descriptions of the participating acupuncturists have been provided thus far.

**Conclusions:**

Overall, the quality of the reporting of RCTs of acupuncture and moxibustion published in Chinese journals has improved since CONSORT and STRICTA were introduced in China, though the actual compliance rate of some important items were still low as of 2012. In the future, Chinese journals should enhance the adoption of the CONSORT and STRICTA statement to improve the reporting quality of the RCTs of acupuncture and moxibustion and to ensure the truth and reliability of the conclusions.

## Introduction

Randomized controlled trials (RCTs) have been regarded as the “gold standard” for assessing the effectiveness of most interventions in the field of medicine all over the world [1,2].

In China, randomized controlled trials (RCTs) have become increasingly popular and have been published in large numbers, particularly over the past two decades. However, only adequate reporting of RCTs can improve transparency and aid in the interpretation and replication of studies.

It is well known that acupuncture & moxibustion are one of the most famous Chinese Traditional Medicine therapies. Evidence-based perspectives are increasingly being applied to traditional medicine, including acupuncture & moxibustion; thus, a wide variety of condition-focused studies have evaluated the reporting quality of acupuncture RCTs on the basis of the Standards for Reporting Interventions in Controlled Trials of Acupuncture (STRICTA) or Consolidated Standards of Reporting Trials (CONSORT) statements [3,4,5,6]. STRICTA guidelines expand on CONSORT item 5 (i.e., intervention) for use in acupuncture trial studies. Currently, STRICTA has been designed to improve the standards for reporting interventions in acupuncture clinical trials. A study performed by Gen et al. investigated the reporting quality of acupuncture RCTs [7]; however, it was only based on some RCTs that were only published in one Chinese journal, Chinese Acupuncture and Moxibustion and did not describe the characteristics of acupuncture RCTs. Furthermore, there have been no studies describing the degree of adherence to acupuncture RCTs or the extent to which adherence to the CONSORT or STRICTA checklists will improve the reporting quality of acupuncture intervention RCTs published in Chinese journals since the CONSORT and STRICTA statements were introduced in China in 1997 and 2003, respectively [8,9]. As far as we know, our previous study was the only one that has been designed to determine the overall quality of RCT reporting published in Chinese pediatrics journals and to examine if there is any improvement over time in China [10].

Thus, we used a similar method as was used in our previous study to describe the characteristics and to assess the effect of the introduction of the CONSORT and STRICTA statement guidelines on the reporting of acupuncture trials. In addition, we aimed to investigate how the studies were reported in the literature and whether the reporting had improved over time in China.

## Methods

### Study selection criteria

All of the RCTs that assessed the efficacy of acupuncture and/or moxibustion that were published in Chinese journals were included. Reports were included only if they involved human subjects. Acupuncture or moxibustion interventions were either administered alone or in combination with conventional Western medicine or herbal medicine. There were no limitations in techniques or styles of acupuncture and moxibustion treatments.

### Database search (S1 File)

We performed a database search of RCTs written in Chinese using the following search terms, “randomized controlled trials”, “RCTs”, “acupuncture”, “electroacupuncture”, “acupuncture manipulation” and “moxibustion”, among others (see S1 File for the complete list of terms used). Four Chinese databases (Chinese Biomedicine Literature Database (CBM), Chinese Scientific Journal Full-text Database (CSJD), Chinese Journal Full-text Database (CJFD), and Wanfang Database) were searched from Jan. 1978 to Dec. 2012. We also examined the reference lists of the retrieved articles and reviews.

### Screening and training of assessors

Two researchers independently screened the titles and abstracts of the identified studies. One reviewer subsequently screened the full text articles of potentially included studies (Jia-ke Xu), while a second reviewer (Bin Ma) independently screened a 20% random sample that was selected from all of the articles that were located by the initial searches. The inter-rater reliability was calculated (Kappa = 0.69), and disagreements between the two researchers were discussed and resolved by the entire team.

### Data extraction

Data were extracted independently by at least two reviewers (Jun-qiang Niu, Li Li, Cheng-ben Huang or Kuang-yang Chen) in accordance with the pre-prepared data extraction forms. Two researchers (Zhi-min Chen and Bin Ma) compiled a table to list general and important information for the final included trials, such as different interventions, including acupuncture and moxibustion, total citation counts, first author role, hospital level, condition focused on in the RCTs, number of authors, research centers involved, placebo use, treatment intention, ethics review and informed consent reporting [11], conflict of interest declaration [12], clinical trial registry [13], and sample size. In addition, the extracted variables included reporting characteristics as well as items from the CONSORT and STRICTA checklists. The conditions examined were classified using the International Classification of Diseases (ICD-10). The total citation counts from every included RCT published in Chinese journals were calculated by the Chinese Science Citation Database which is similar to the Science Citation Index.

### Assessor training

To ensure interpretation consistency, the two assessors (YFK and MZC) underwent training on each item of the CONSORT and STRICTA in detail by a qualified trainer who had undergone training in the Chinese Cochrane Center. A 10% random sample was independently interpreted by both assessors. Next, the inter-rater reliability was calculated (Kappa = 0.70), and disagreements were resolved by joint discussion with a third assessor (BM).

### Data analysis

We investigate the potential changes in the reporting quality between three distinct time periods. We aimed to establish a baseline for reporting quality and to track changes over time after the publication of CONSORT and STRICTA. CONSORT and STRICTA were first introduced in China in 1997 and 2003, respectively, and all of the trials were subsequently grouped in three publication periods, i.e., before 1996 (pre-CONSORT), 1997–2003 (post-CONSORT and pre-STRICTA) and 2004–2012 (post-STRICTA), according to when the reporting was assessed. Each item of CONSORT and STRICTA was assessed as “yes” if it was described in the paper or “no” if it was not (S2 File).

Dichotomous data were summarized with descriptive statistical analyses (frequency, median, interquartile range (IQR)). Pearson's χ2 tests were used when sample sizes were more than 40 and expected counts were more than five, and Fisher's exact tests were used when sample sizes were less than 40 or expected counts were more than zero but less than one to assess reporting differences among the three groups. P values less than 0.05 were considered to be significant. Analyses were performed using Excel (version Microsoft Excel 2003; http://office.microsoft.com/zh-cn/) and SPSS (version 18.0; http://www.spss.com).

## Results

### Sample selection and flow (Fig 1)

**Fig 1:**
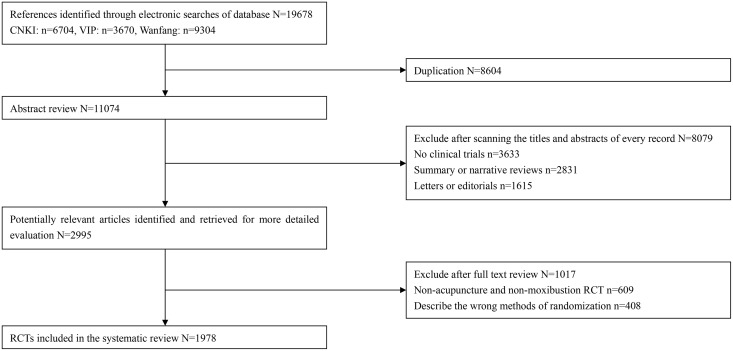
Flow chart of the articles identified, included and excluded. RCTs, Randomisation Controlled Trials.

The searches initially identified 19,678 potentially relevant records. The process used to select potentially relevant studies for inclusion in our study is shown in Fig 1. Of the 19,678 references, 8604 were duplications. An additional 8,709 references were excluded, 1615 of which were letters or editorials, 3633 were not clinical trials, and 2831 were summary or narrative reviews. Then, an additional 1,017 articles were excluded, 609 of which were non-acupuncture RCTs and 408 described incorrect randomization methods. The remaining 1978 RCTs were included in our study; one hundred forty-four RCTs were published before 1996, 353 RCTs were published from 1997 to 2003, and 1481 RCTs were published from 2004 to 2012 (S3 File). The first RCTs using acupuncture or moxibustion intervention were published in 1987 [14] and 1989 [15] in China, respectively.

### Summary of the RCTs (Table 1)

A summary of RCTs is presented in Table 1. The number of RCTs using acupuncture and moxibustion intervention has rapidly increased over the years in China; one hundred forty-four RCTs were published before 1996. Of these RCTs, the intervention in 122 RCTs focus on acupuncture and 22 RCTs focus on moxibustion. Furthermore, 353 RCTs were published from 1997 to 2003; in 312 of those RCTs, the intervention was acupuncture, and in the remaining 41 RCTs, the intervention was moxibustion. In addition, 1481 RCTs were published from 2004 to 2012; in 1301 of those RCTs, the intervention was acupuncture, and in the remaining 180 RCTs, the intervention was moxibustion. Nearly one- fourth of the RCTs (24.4%) published between 2004 and 2012 have not been cited by any author in the same research field; however, this proportion was less than the other two periods, and significant differences were also found in the other two periods (time 3 vs time 1, P = 0.024; time 3 vs time 2, P = 0.025). Only 14.2% of the studies published between 2004 and 2012 had been cited more than 10 times, and no significant differences were found in the other two periods.

**Table 1:** Summary of Acupuncture and Moxibustion RCTs Published in Chinese Journals.

Category	Characteristic	All RCTs n = 1978(%)	≤1996 n = 144(%)	1997–2003 n = 353(%)	2004–2012 n = 1481(%)
Intervention type	acupuncture intervention	1735(87.7)	122(84.7)	312(88.4)	1301(87.8)
	moxibustion intervention	243(12.3)	22(15.3)	41(11.6)	180(12.2)
Total citation counts	0	574(29.0)	60(41.7)	152(43.0)	362(24.4)*#
	1–5	880(44.5)	55(38.2)	130(36.8)	695(46.9)
	6–10	243(12.3)	12(8.3)	50(14.2)	181(12.3)
	11–15	148(7.5)	10(6.9)	30(8.5)	108(7.2)
	>15	133(6.7)	7(4.9)	23(6.5)	103(7.0)
Role of first author	Clinician	1108(56.0)	4(2.8)	87(24.6) *	1016(68.6) *#
	Researcher	172(8.7)	12(8.3)	10(2.8)	162(10.9)#
	Graduate student	99(5.0)	0(0.0)	3(0.8)*	97(6.5)*
	Other	599(30.3)	128(88.9)	253(71.7)	206(13.9) *#
Hospital level	Primary hospital	359(18.1)	61(42.4)	112(31.7)	186(12.6) *#
	Secondary hospital	665(33.6)	46(31.9)	138(39.1)	481(32.5)
	Tertiary hospital	954(48.3)	37(25.6)	103(29.2)	814(54.9) *#
Condition that the RCT focused on (Common ICD-10)	Diseases of the circulatory system	410(20.8)	25(17.4)	73(20.7)	312(21.1)
	Diseases of the digestive system	133(6.7)	13(9)	24(6.8)	96(6.4)
	Diseases of the nervous system	310(15.7)	32(22.2)	64(18.1)	214(14.4)
	Diseases of the genitourinary system	137(6.9)	9(6.3)	18(5.1)	110(7.4)
	Diseases of the respiratory system	53(2.7)	4(2.8)	7(2.0)	42(2.8)
	Diseases of the blood and blood-forming organs and immune mechanism	20(1.0)	2(1.4)	4(1.2)	14(0.9)
	Diseases of the musculoskeletal system and connective tissue	414(20.9)	28(19.4)	72(20.4)	314(21.2)
	Mental and behavioral disorders	139(7.0)	4(2.8)	17(4.8)	118(8.0)
	Diseases of the skin and subcutaneous tissue	74(3.7)	4(2.8)	12(3.4)	58(3.9)
	Endocrine, nutritional and metabolic diseases	107(5.4)	4(2.8)	19(5.4)	84(5.7)
	Neoplasms	11(0.6)	3(2.1)	2(0.6)	6(0.4)
	Infectious and parasitic diseases	9(0.5)	0(0)	3(0.8)*	6(0.4)*
	Pregnancy, childbirth and puerperium	20(1.0)	3(2.1)	8(2.3)	9(0.6)
	Diseases of the ear and mastoid process	7(0.5)	0(0)	2(0.6)*	5(0.5)*
	Diseases of the eye and adnexa	23(1.2)	1(0.7)	6(1.7)	16(1.1)
	Injury, poisoning and certain other consequences of external causes	83(4.2)	6(4.2)	21(5.9)	56(3.8)
	Specific conditions originating during the perinatal period	18(0.7)	10(2.8)	1(0.3)	7(0.5)
	Factors affecting health status and contact with health services	17(0.7)	5(1.4)	0(0)*	12(0.8)#

* indicates a significant difference with the RCTs published in 1996 or earlier (P≤0.05);

^#^ indicates a significant difference between the RCTs published between 1997 and 2003 (P≤0.05).

There is an increasing number of clinicians who play an important role in clinical trial research in China. Specifically, after 2003, 68.8% of articles had clinicians as primary authors. There were significant differences among the three groups (time 3 vs time 1, P<0.001; time 3 vs time 2, P = 0.005; time 2 vs time 1, P = 0.016). The most common conditions examined were the same in three different periods, including diseases of the musculoskeletal system and connective tissue (21.2%), diseases of the circulatory system (21.1%) and diseases of the genitourinary system (14.4%).

### Study description of the RCT characteristics (Table 2)

Changes in the percentage of each RCT characteristic in studies published before and after the implementation of CONSORT and STRICTA are presented in Table 2. The RCTs included a median of three authors (IQR: 2.0–4.0) and a median of 80 for sample size (IQR: 60–120). These two values did not improve over different publication years, and there was no significant difference in these values. Only 39.2% of the RCTs published between 2004 and 2012 were from multi-centers in China, although this proportion was improved compared to the other two periods, and a significant difference was found between the RCTs published in 1996 or earlier (time 3 vs time 1, P = 0.017).

**Table 2:** Descriptive Characteristics of Acupuncture and Moxibustion RCTs Published in Chinese Journals.

Category	All RCTs n = 1978(%)	≤1996 n = 144(%)	1997–2003 n = 353(%)	2004–2012 n = 1481(%)
Number of authors Median (IQR)	3(2,4)	2(1,3)	3(1,4)	3(2,4)
Sample size Median (IQR)	80(60,120)	89(60,134)	90(60,120)	80(60,118.5)
Involving research centers				
Single center n(%)	1260(63.7)	109(75.7)	250(70.8)	901(60.8)
Multi-center n(%)	718(36.3)	35(24.3)	103(29.2)	580(39.2)*
Informed consent Yes n(%)	328(16.6)	1(0.7)	5(1.4)	322(21.8) *#
Ethical review Yes n(%)	14(0.7)	0(0)	0(0)	14(0.9) *#
Use of placebo (Sham acupuncture) Yes n(%)	26(1.3)	1(0.7)	2(0.6)	23(1.5)
Treatment Intention Yes n(%)	14(0.7)	0(0)	0(0)	14(0.9) *#
Declaration for conflict of interests Yes n(%)	0(0)	0(0)	0(0)	0(0)

* indicates a significant difference with the RCTs published in 1996 or earlier (P≤0.05);

^#^ indicates a significant difference with the RCTs published between 1997 and 2003 (P≤0.05).

In addition, although the authors of the RCTs published after 2004 paid more attention to informed consent and ethical review compared with the other two periods and a significant difference was also found in each of the other two periods (time 3 vs time 1, P<0.001; time 3 vs time 2, P<0.001 for informed consent; time 3 vs time 1, P = 0.014; time 3 vs time 2, P = 0.012 for ethical review), this proportion remained very low (21.8% versus 0.9%). Likewise, the same situation was found in studies that used placebos (1.5%) and in treatment intention (0.9%). None of the authors of the included RCTs declared any conflicts of interest up to 2012.

### Characteristics and variation in CONSORT reporting over time (Table 3)

The differences in the percentage of individual items that were reported in studies published before and after the implementation of CONSORT are presented in Table 3. However, none of the acupuncture or moxibustion RCTs published up to 2012 in Chinese journals reported certain items, including methods (3b “Trial design”, 6b“Outcome”, 7b“Sample size”), results (14b“Recruitment”, 19“Harms”) or other information (23“Registration”, 24“Protocol”). Although the percentage of the inclusion of other items has increased over the past years, the actual compliance remains low.

**Table 3:** CONSORT Assessment of the Reporting Characteristics of Acupuncture and Moxibustion RCTs Published in Chinese Journals.

Section/Topic	Item No	Checklist item	All RCTs n = 1978(%)	≤1996 n = 144(%)	1997–2003 n = 353(%)	2004–2012 n = 1481(%)
**Title and abstract**						
	1a	Identification of a randomized trial in the title	950(48.0)	4(2.7)	79(22.4)*	867(58.5) *#
	1b	Structured summary of the trial design, methods, results, and conclusions	1692(85.5)	50(34.7)	257(72.8)*	1385(93.5) *#
**Introduction**						
Background and objectives	2a	Scientific background and explanation of the rationale	594(30.0)	26(18.1)	101(28.6)*	467(31.5)*
Trial design	2b	Specific objectives or hypotheses	431(21.8)	4(2.8)	38(10.7)	389(26.2) *#
	3a	Description of the trial design (such as parallel, factorial), including the allocation ratio	817(41.3)	14(9.7)	106(30.0)*	697(47.1) *#
	3b	Important changes to the methods after trial commencement (such as eligibility criteria), with reasons	0(0.0)	0(0.0)	0(0.0)	0(0.0)
Participants	4a	Eligibility criteria for participants	1554(78.6)	69(47.9)	231(65.4)*	1254(84.7) *#
	4b	Settings and locations where the data were collected	1137(57.5)	18(12.5)	87(24.6)*	1032(69.7) *#
Outcomes	6a	Completely defined pre-specified primary and secondary outcome measures, including how and when they were assessed	827(41.8)	38(26.4)	121(34.3)	668(45.1) *#
	6b	Any changes to the trial outcomes after the trial commenced, with reasons	0(0.0)	0(0.0)	0(0.0)	0(0.0)
Sample size	7a	How the sample size was determined	17(0.8)	0(0.0)	0(0.0)	17(1.2) *#
	7b	When applicable, explanation of any interim analyses and stopping guidelines	0(0.0)	0(0.0)	0(0.0)	0(0.0)
**Randomization**						
Sequence generation	8a	Method used to generate the random allocation sequence	445(22.5)	2(1.4)	53 (15.0)*	390(26.3) *#
	8b	Type of randomization; details of any restriction (such as blocking and block size)	117(5.9)	0 (0.0)	13(3.7)*	104(7.0)*
Allocation concealment mechanism	9	Mechanism used to implement the random allocation sequence (such as sequentially numbered containers), description of any steps taken to conceal the sequence until interventions were assigned	78(3.9)	0(0.0)	5(1.4)*	73(4.9)*
Implementation	10	Who generated the random allocation sequence, who enrolled participants, and who assigned participants to interventions	21(1.0)	0(0.0)	2(0.6)*	19(1.3)*
Blinding	11a	If performed, who was blinded after assignment to interventions (for example, participants, care providers, those assessing outcomes) and how	155(7.8)	0(0.0)	20(5.7)*	135(9.1)*
	11b	If relevant, a description of the similarity of interventions	109(5.5)	0(0.0)	13(3.7)*	96(6.5)*
Statistical methods	12a	Statistical methods used to compare groups for primary and secondary outcomes	936(47.3)	18(12.5)	112(31.7)*	806(54.4) *#
	12b	Methods for additional analyses, such as subgroup analyses and adjusted analyses	319(16.1)	0(0.0)	24(6.8)*	295(19.9) *#
**Results**						
Participant flow (a diagram is strongly recommended)	13a	For each group, the numbers of participants who were randomly assigned received the intended treatment and were analyzed for the primary outcome	346(17.5)	0(0.0)	41(11.6)*	305(20.6) *
	13b	For each group, losses and exclusions after randomization, together with reasons	343(17.3)	0(0.0)	39(11.0)*	304(20.5) *
Recruitment	14a	Dates defining the periods of recruitment and follow-up	65(3.3)	3 (2.1)	10(2.8)	52(3.5)
	14b	Why the trial ended or was stopped	0(0.0)	0(0.0)	0(0.0)	0(0.0)
Baseline data	15	A table showing the baseline demographic and clinical characteristics for each group	591(29.9)	13(9.0)	63(17.8)	515(34.8) *#
Number analyzed	16	For each group, the number of participants (denominator) included in each analysis and whether the analysis was performed by the original assigned groups	488(24.7)	8(5.6)	66(18.7)*	414(28.0) *#
Outcomes and estimation	17a	For each primary and secondary outcome, the results for each group and the estimated effect size and its precision (such as 95% confidence interval)	904(45.7)	26(18.1)	127(36.0)*	751(50.7) *#
	17b	For binary outcomes, a presentation of both the absolute and relative effect sizes is recommended	52(2.6)	0(0.0)	4(1.1)*	48(3.2) *#
Ancillary analyses	18	Results of any other analyses performed, including subgroup analyses and adjusted analyses, distinguishing pre-specified from exploratory	116(5.9)	0(0.0)	21(5.9)*	95(6.4)*
Harms	19	All important harms or unintended effects in each group (for specific guidance see CONSORT for harms)	0(0.0)	0(0.0)	0(0.0)	0(0.0)
**Discussion**						
Limitations	20	Trial limitations, addressing sources of potential bias, imprecision, and, if relevant, multiplicity of analyses	159(8.0)	11(7.6)	27(7.6)	121(8.2)
Generalizability	21	Generalizability (external validity, applicability) of the trial findings	875(44.2)	42(29.2)	125(35.4)	708(47.8) *#
Interpretation	22	Interpretation consistent with the results, balancing benefits and harms, considering the other relevant evidence	965(48.8)	52(36.1)	167(47.3)*	746(50.4)*
**Other information**						
Registration	23	Registration number and the name of the trial registry	0(0.0)	0(0.0)	0(0.0)	0(0.0)
Protocol	24	Location where the full trial protocol can be accessed, if available	0(0.0)	0(0.0)	0(0.0)	0(0.0)
Funding	25	Sources of funding and other support (such as supply of drugs), role of funders	333(16.8)	2(1.4)	39(11.0)	292(19.7) *

* indicates a significant difference with the RCTs published in 1996 or earlier (P≤0.05);

^#^ indicates a significant difference with the RCTs published between 1997 and 2003 (P≤0.05).

Most RCTs published between 2004 and 2012 were compliant with the following checklist items: title, abstract (1b) and methods (4a); significant differences were found among the three groups (P = 0.001). More than half of the RCTs published between 2004 and 2012 were compliant with the following checklist items: title, abstract (1a), methods (4b“Participants”), randomization (12a“Statistical methods”), results (17a“Outcome estimation”) and discussion (22“Interpretation”); significant differences were also found among the three groups (P = 0.000). Less than half of the RCTs published between 2004 and 2012 were compliant with the following checklist items: introduction (2a“Background”, 2b“Objective”), methods (3a“Trial design”, 6a“Outcome”), randomization (8a“Sequence generation”, 12a“Statistical methods”, 12b“Statistical methods”), results (13a“Participant flow”, 13b“Participant flow”, 15“Baseline data”, 16“Number analyzed”), discussion (21“Generalisability”) and other information (25“Funding”); however, the percentage of the above items was increased compared to the other two periods. A significant difference was also found either with RCTs published in 1996 or earlier (P = 0.000) or between 1997 and 2003 (P = 0.001). Few RCTs published between 2004 and 2012 were compliant with the following checklist items: methods (7a“Sample size”), randomization (8b“Sequence generation”, 9“Allocation concealment”, 10“Implementation”, 11a“Blinding”, 11b“Blinding”), results (14a“Recruitment”, 17b“Outcoms and estimation”, 18“Ancillary analyses”) and discussion (20“Limitations”); however, the percentage of the above items was increased compared to the other two periods. Significant differences were also found either with RCTs published in 1996 or earlier (P = 0.000) or between 1997 and 2003 (P = 0.021), with the exception of items 14a and 20.

### Characteristics and variation in STRICTA reporting over time (Table 4)

Differences in the percentage of individual items reported in studies published before and after the implementation of STRICTA are presented in Table 4. Overall, the percentage of items on the checklist increased over time. However, up to 2012, none of the acupuncture or moxibustion RCTs published in Chinese journals reported components of the treatment (4b).

**Table 4:** STRICTA Assessment of the Reporting Characteristics of Acupuncture and Moxibustion RCTs Published in Chinese Journals.

Section/Topic	Checklist item	All RCTs n = 1735 (%)	≤1996 n = 122(%)	1997–2003 n = 312(%)	2004–2012 n = 1301(%)
Acupuncture rationale	1a. Style of acupuncture (e.g., Traditional Chinese Medicine, Japanese, Korean, Western medical, Five Element, ear acupuncture, etc.)	204(11.8)	8(6.6)	31(9.9)	165(12.7)
	1b. Reason for the treatment provided, based on the historical context, literature sources and/or consensus methods, with references where appropriate	14(0.8)	0(0.0)	1(0.3)*	13(1.0) *
	1c. Extent to which treatment was varied	208(12.0)	6(4.9)	56(17.9)*	146(11.2)
Details of needling	2a. Number of needle insertions per subject per session (the mean and range where relevant)	3(0.2)	0(0)	1(0.3)*	2(0.2) *
	2b. Names (or location if no standard name) of the points used (uni-/bilateral)	1343(77.4)	89(73.0)	218(69.9)	1036(79.6)
	2c. Depth of insertion, based on a specified unit of measurement or on a particular tissue level	793(45.7)	29(23.8)	113(36.2)*	651(50.0) *#
	2d. Responses sought (e.g., de qi or muscle twitch response)	1217(70.1)	68(55.7)	214(68.6)*	935(71.9)*
	2e. Needle stimulation (e.g., manual or electrical)	1475(85.0)	101(82.8)	260(83.3)	1114(85.6)
	2f. Needle retention time	1523(87.8)	98(80.3)	274(87.8)	1151(88.5)
	2g. Needle type (diameter, length and manufacturer or material)	1140(65.7)	54(44.3)	175(56.1)*	911(70.0) *#
Treatment regimen	3a. Number of treatment sessions	1659(95.6)	105(86.1)	300(96.2)	1254(96.4)
	3b. Frequency and duration of treatment sessions	1656(95.4)	104(85.2)	297(95.2)	1255(96.5)
Other components of treatment	4a. Details of other interventions administered to the acupuncture group (e.g., moxibustion, cupping, herbs, exercises, lifestyle advice)	557(32.1)	24(19.7)	94(30.1)	439(33.7)*
	4b. Setting and context of treatment, including instructions to practitioners, and information and explanations to patients	0(0)	0(0)	0(0)	0(0)
Practitioner background	5. Description of participating acupuncturists (qualification or professional affiliation, years in acupuncture practice, other relevant experience)	5(0.4)	0(0)	0(0)	5(0.4) *#
Control or comparator interventions	6a. Rationale for the control or comparator in the context of the research question, with sources that justify the choice(s)	77(4.4)	0(0.0)	6(1.9)*	71(5.5) *
	6b. Precise description of the control or comparator. If sham acupuncture or any other type of acupuncture-like control was used, provided details as for items 1–3 above	110(6.3)	5(4.1)	16(5.1)	89(6.8)

* indicates a significant difference with the RCTs published in 1996 or earlier (P≤0.05);

^#^ indicates a significant difference with the RCTs published between 1997 and 2003 (P≤0.05).

Most RCTs that were published in the three time periods were compliant with the following checklist items, details of needling (2b, 2e, 2f) and treatment regimen (3a, 3b); however, no significant differences were found among the three groups. More than half of the RCTs published between 2004 and 2012 were compliant with the details of needling (2c, 2d, 2 g) checklist item, and significant differences were also found either with the RCTs published in 1996 or earlier (P = 0.001) or between 1997 and 2003 (P = 0.018). Less than half of the RCTs published between 2004 and 2012 were compliant with the following checklist items: acupuncture rationale (1a, 1c) and other components of treatment (4a); a significant difference was only found with the RCTs published in 1996 or earlier in item 4a (P = 0.031). Few of the RCTs (i.e., ≤10%) published between 2004 and 2012 were compliant with the following checklist items: acupuncture rationale (1b), details of needling (2a), practitioner background (5), and control or comparator interventions (6a, 6b); significant differences were also found either with the RCTs published in 1996 or earlier (P = 0.029) or between 1997 and 2003 (P = 0.033) with the exception of item 6b.

## Discussion

Our study identified 1978 acupuncture and moxibustion intervention RCTs published in Chinese medical journals. This is the first review to examine compliance and to track changes of reporting quality of acupuncture and moxibustion intervention RCTs over time after the publication of the CONSORT and STRICTA in China.

Many of the earlier researchers/acupuncturists in China focused on an appropriate choice of acupuncture and moxibustion and paid more attention to its specific effects. This may be a result of the social perception of acupuncture and moxibustion, which is completely different in China than in other countries. Because these acupuncture techniques were invented by Chinese medical practitioners and have been used in the medical field for more than 2000 years in China, Chinese clinical researchers may be more interested in the types of acupuncture and moxibustion techniques used in clinical practice. With the introduction and influence of the conception of evidence-based medicine in China, a growing number of Chinese researchers have begun to focus on the evaluation of acupuncture and moxibustion efficacy, its potential role in health care, and the quality of these RCTs. Overall, though the actual compliance rate of items related to the methodology and registration information remain low up to 2012, our survey demonstrated that the reporting quality of acupuncture and moxibustion intervention RCTs that were published in Chinese journals has improved over time since the introduction of CONSORT and STRICTA in China in 1997 and 2003, respectively.

Many deficits in reporting were evident in these RCTs, and we identified areas of particular concern. Our survey revealed that the total citation counts in 71.3% of the included RCTs were lower than five and the total citation counts of nearly one-third (24.4%) of the studies were zero. In addition, the reporting of ethical issues was inadequate, with less than 1% of the included RCTs reporting ethical committee approval up to 2012, even though it is a legal requirement in China [16]. The above findings in this study were similar with our previous study [10] that focused on the reporting all of the RCTs published in Chinese pediatrics journals. Compared with the RCTs published prior to 2003, more trials published between 2004 and 2012 have described that participants attended of “their own free will” and provided details about informed consent procedures, although the actual percentage remained low (21.8%). However, this level was lower than Tsukayama et al.’s reviews [17] focusing on the Japanese literature. Similarly, sham acupuncture was used in very few RCTs (1.3%), and the proportion of these procedures in three different periods had not improved over time (P>0.05). Moreover, no RCTs reported conflicts of interest up to 2012. This may be a result of a lack of compulsory policies in Chinese journals, in which the authors must report conflicts of interest.

Our survey also found some disappointing results. For example, only 17 (1.2%) RCTs published between 2004 and 2012 reported how the sample size was determined or described the procedure of the sample estimate, which may be challenging for most of the RCTs published in Chinese journals. Although the International Committee of Medical Journal Editors (ICMJE) requires all clinical trials to be registered in an effort to increase transparency and accountability [18,19], no acupuncture or moxibustion RCTs published in Chinese journals have registered or reported a registration number up to 2012. This may be because of a lack of compulsory policies in China, because the Chinese periodicals association only recommends priority for published clinical trials that have been registered [20]. All of these observations were also made in our previously published study on pediatric journal articles [10]. In addition, more serious challenges were found in the randomization description; up to 2012, many trials did not report detailed information on the method used to generate the random allocation sequence, allocation concealment mechanism or implementation. Although the compliance rate of items related to randomization increased over time and significant differences were also found, the actual percentage was still very low. These findings that the low percentage of items related to randomization appeared to be similar to Dr. Mao’s study [21]. Similarly, most trials still did not provide detailed information about the blinding of either participants or investigators. Participants who are aware of their treatment may behave differently or have particular expectations [22], thus affecting the results. Further, blinding may affect the measurement of subjective outcomes. However, a systematic review found that for subjective outcomes, trials that used inadequate or unclear allocation concealment yielded 31% larger estimates of the effect compared to those that used adequate concealment, and the trials that were not blinded yielded 25% larger estimates [23].

In addition, the actual percentage of most of the STRICTA items remained very low over time in China. Six items detailing needling (except item 2a) and the compliance rate were good in the three different periods. However, as for the other items, the actual percentage was still very low after STRICTA was introduced in China in 2003. For example, the percentage of the items, such as the reason for treatment, number of needle insertions per subject per session, and description of participating acupuncturists, as still very low (<1%) after 2003. This was mainly because some researchers in the field of acupuncture did not understand the reporting guidelines and the periodical editorial department did not require the authors to report acupuncture intervention RCTs according to STRICTA items in China [21]. Furthermore, a study found that some acupuncture trials reported that the STRICTA items originally included in the submitted manuscripts were removed during the editorial process [24]. Moreover, a study by Li XQ et al. assessed the endorsement of the CONSORT statement by high-impact medical journals in China by reviewing the instructions for authors and showed that only 6 (6/195, 3.08%) and 14 (14/200, 7.00%) medical journals in China mentioned “CONSORT” or “ICMJE” in their instructions for authors, respectively, and all of these journals used ambiguous language on what was expected of the authors [25]. Of course, because of the time-lag effect, not all of the authors or medical journals could comply with these reporting guidelines from the beginning of their study. Thus, we strongly recommend the use of the CONSORT and STRICTA statements by authors. We also recommend that editors of Chinese medical journals recognize and promote the use of the CONSORT and STRICTA statements in their publications.

In summary, though our study found that the reporting situation of acupuncture RCTs published in Chinese journals was unsatisfactory, from the data in the tables, we are encouraged to observe that the reporting percentages of each item in the STRICTA and CONSORT statements have improved from before.

There are also some limitations to our study. We focused our assessment on examining compliance and tracking changes of reporting quality of acupuncture RCTs over time after the publication of CONSORT and STRICTA statements in China. Thus, we cannot make inferences regarding the relationship between CONSORT adherence and trial quality or the validity of the trial results. We only selected acupuncture and moxibustion RCTs published in Chinese journals indexed in the Chinese Science Citation Database; thus, our findings may not represent the reporting quality of RCTs published in foreign journals. We assessed the quality of the current RCTs as judged by the authors' description in articles; we did not attempt to contact the authors to verify the detailed method, and thus, the quality of the RCTs included in our study may be exaggerated. In addition, only 20% random sample were screened by a second reviewer though inter-rater reliability was 0.69 and only 10% random sample was independently interpreted by both assessors in our review; thus, both them may impact the reliability of the ratings of the total sample.

Overall, the quality of reporting acupuncture and moxibustion RCTs published in Chinese journals has improved since CONSORT and STRICTA statements were introduced in China in 1997 and 2003, respectively. However, up to 2012, current RCTs, as judged by their publication, have not comprehensively reported the information recommended in the CONSORT and STRICTA statements and are often not adequate to enable readers to assess trial validity. In the future, Chinese journals should enhance the adoption of the CONSORT and STRICTA statements to improve the reporting quality of acupuncture and moxibustion RCTs and to ensure the truth and reliability of the conclusions.

## Supporting Information

S1 File Search strategy of the four Chinese databases. (DOC)

S2 File Definitions of reporting items of CONSORT & STRICTA checklist. (DOC)

S3 File Included 1978 RCTs of acupuncture & moxibustion published in Chinese journals. (DOC)

S4 File Raw extraction table. (XLS)
