# Supplementary material for: Do the CONSORT and STRICTA Checklists Improve the Reporting Quality of Acupuncture and Moxibustion Randomized Controlled Trials Published in Chinese Journals? A Systematic Review and Analysis of Trends
Source: PLoS One. 2016 Jan 25;11(1):e0147244. doi: 10.1371/journal.pone.0147244 (PMC4726495; doi:10.1371/journal.pone.0147244)
Supplement: S1 File — (DOC) [file pone.0147244.s001.doc]

**The four databases are not free for search in China, so we searched all the data by using our campus LAN(Lanzhou University).**

**Chinese Biomedicine Literature Database(CBM) search strategy:**

***Hyperlink address：***<http://sinomed.imicams.ac.cn/index.jsp>

#1 Randomized controlled trials/exp

#2 Randomized controlled trials

#3 RCTs

#4 Clinical trial/exp

#5 Clinical trial

#6 #1 or #2 or #3 or #4 or #5

#7 acupuncture /exp

#8 acupuncture

#9 electroacupuncture /exp

#10 electroacupuncture

#11 acupuncture manipulation

#12 moxibustion /exp

#13 moxibustion

#14 #7 or #8 or #9 or #10 or #11 or #12 or #13 or #14

#15 #6 and #14

**Chinese Scientific Journal Full-text Database (CSJD) search strategy**

***Hyperlink address：***<http://www.cnki.net/>

#1 Randomized controlled trials

#2 RCTs

#3 Clinical trial

#4 #1 or #2 or #3

#5 acupuncture

#6 electroacupuncture

#7 acupuncture manipulation

#8 moxibustion

#9 #5 or #6 or #7 or #8

#10 #4 and #9

**Chinese Journal Full-text Database (CJFD) search strategy**

***Hyperlink address：***<http://www.cqvip.com/>

#1 Randomized controlled trials

#2 RCTs

#3 Clinical trial

#4 #1 or #2 or #3

#5 acupuncture

#6 electroacupuncture

#7 acupuncture manipulation

#8 moxibustion

#9 #5 or #6 or #7 or #8

#10 #4 and #9

**Wanfang Database search strategy**

Hyperlink address：<http://www.wanfangdata.com.cn/>

#1 Randomized controlled trials

#2 RCTs

#3 Clinical trial

#4 #1 or #2 or #3

#5 acupuncture

#6 electroacupuncture

#7 acupuncture manipulation

#8 moxibustion

#9 #5 or #6 or #7 or #8

#10 #4 and #9
