# Supplementary material for: Do the CONSORT and STRICTA Checklists Improve the Reporting Quality of Acupuncture and Moxibustion Randomized Controlled Trials Published in Chinese Journals? A Systematic Review and Analysis of Trends
Source: PLoS One. 2016 Jan 25;11(1):e0147244. doi: 10.1371/journal.pone.0147244 (PMC4726495; doi:10.1371/journal.pone.0147244)
Supplement: S2 File — (DOC) [file pone.0147244.s002.doc]

**Text S2: Definitions of reporting items(CONSORT)**

| **Section/Topic** | **Item No** | **Definition of “yes”** |
| --- | --- | --- |
| **Title and abstract** | | |
|  | 1a | Reported “randomised trial” in the title |
|  | 1b | Reported structured summary of the trial design, methods, results, and conclusions |
| **Introduction** | | |
| Background and objectives | 2a | Reported scientific background and explanation of the rationale |
|  | 2b | Reported specific objectives or hypotheses |
| **Methods** | | |
| Trial design | 3a | Reported the trial design (such as parallel, factorial), including the allocation ratio |
|  | 3b | Reported important changes to the methods after trial commencement (such as eligibility criteria), with reasons |
| Participants | 4a | Reported eligibility criteria for participants |
|  | 4b | Reported settings and locations where the data were collected |
| Outcomes | 6a | Reported completely defined pre-specified primary and secondary outcome measures, including how and when they were assessed |
|  | 6b | Reported changes to the trial outcomes after the trial commenced, with reasons |
| Sample size | 7a | Reported How was determined the sample size |
|  | 7b | Reported when applicable, explanation of any interim analyses and stopping guidelines |
| Sequence generation | 8a | Reported method used to generate the random allocation sequence |
|  | 8b | Reported type of randomisation; details of any restriction (such as blocking and block size) |
| Allocation concealment mechanism | 9 | Reported mechanism used to implement the random allocation sequence (such as sequentially numbered containers), description of any steps taken to conceal the sequence until interventions were assigned |
| Implementation | 10 | Reported who generated the random allocation sequence, who enrolled participants, and who assigned participants to interventions |
| Blinding | 11a | Reported who was blinded after assignment to interventions (for example, participants, care providers, those assessing outcomes) and how |
|  | 11b | Reported the similarity of interventions |
| Statistical methods | 12a | Reported statistical methods used to compare groups for primary and secondary outcomes |
|  | 12b | Reported methods for additional analyses, such as subgroup analyses and adjusted analyses |
| **Results** | | |
| Participant flow (a diagram is strongly recommended) | 13a | For each group, reported the numbers of participants who were randomly assigned received the intended treatment and were analysed for the primary outcome |
|  | 13b | For each group, Reported losses and exclusions after randomisation, together with reasons |
| Recruitment | 14a | Reported dates defining the periods of recruitment and follow-up |
|  | 14b | Reported why the trial ended or was stopped |
| Baseline data | 15 | Reported a table showing the baseline demographic and clinical characteristics for each group |
| Number analysed | 16 | For each group, reported the number of participants (denominator) included in each analysis and whether the analysis was performed by the original assigned groups |
| Outcomes and estimation | 17a | For each primary and secondary outcome, reported the results for each group and the estimated effect size and its precision (such as 95% confidence interval) |
|  | 17b | For binary outcomes, reported a presentation of both the absolute and relative effect sizes |
| Ancillary analyses | 18 | Reported results of any other analyses performed, including subgroup analyses and adjusted analyses, distinguishing pre-specified from exploratory |
| Harms | 19 | Reported all important harms or unintended effects in each group (for specific guidance see CONSORT for harms) |
| **Discussion** | | |
| Limitations | 20 | Reported limitations of trial, addressing sources of potential bias, imprecision, and, if relevant, multiplicity of analyses |
| Generalisability | 21 | Reported generalisability (external validity, applicability) of the trial findings |
| Interpretation | 22 | Interpreted consistent with the results, balancing benefits and harms, considering the other relevant evidence |
| **Other information** | | |
| Registration | 23 | Reported registration number and the name of the trial registry |
| Protocol | 24 | Reported location where the full trial protocol can be accessed if the trial was registered |
| Funding | 25 | Reported sources of funding and other support (such as supply of drugs), role of funders |

**Text S2: Definitions of reporting items(STRICTA)**

| **Section/Topic** | **item** | Definition of “yes” |
| --- | --- | --- |
| Acupuncture rationale | 1a | Reported the style of acupuncture, e.g.,Traditional Chinese Medicine, Japanese, Korean, Western medical, Five Element, ear acupuncture, etc |
|  | 1b | Provided reason for treatment, based on the historical context, literature sources and/or consensus methods, with references where appropriate |
|  | 1c | Reported extent to which treatment was varied |
| Details of needling | 2a. | Reported number of needle insertions per subject per session (the mean and range where relevant) |
|  | 2b | Reported names (or location if no standard name) of the points used (uni-/bilateral) |
|  | 2c | Reported depth of insertion, based on a specified unit of measurement or on a particular tissue level |
|  | 2d | Reported responses sought (e.g., de qi or muscle twitch response) |
|  | 2e | Reported needle stimulation (e.g., manual or electrical) |
|  | 2f | Reported needle retention time |
|  | 2g | Reported needle type (diameter, length and manufacturer or material) |
| Treatment regimen | 3a | Reported number of treatment sessions |
|  | 3b | Reported frequency and duration of treatment sessions |
| Other components of treatment | 4a | Reported details of other interventions administered to the acupuncture group (e.g., moxibustion, cupping, herbs, exercises, lifestyle advice) |
|  | 4b | Reported setting and context of treatment, including instructions to practitioners, and information and explanations to patients |
| Practitioner background | 5 | Reported the participating acupuncturists (qualification or professional affiliation, years in acupuncture practice, other relevant experience) |
| Control or comparator interventions | 6a | Reported rationale for the control or comparator in the context of the research question, with sources that justify the choice(s) |
|  | 6b | Reported precise description of the control or comparator. If sham acupuncture or any other type of acupuncture-like control was used, provided details as for items 1–3 above |
