# Supplementary material for: Do the CONSORT and STRICTA Checklists Improve the Reporting Quality of Acupuncture and Moxibustion Randomized Controlled Trials Published in Chinese Journals? A Systematic Review and Analysis of Trends
Source: PLoS One. 2016 Jan 25;11(1):e0147244. doi: 10.1371/journal.pone.0147244 (PMC4726495; doi:10.1371/journal.pone.0147244)
Supplement: S3 File — (DOC) [file pone.0147244.s003.doc]

[1] 张福顺（2000）左右交替针刺治疗周围性面瘫168例疗效观察.北京针灸骨伤学院学报7:31-33.

[1] Zhang Fu-shun(2000). Acupuncture for Peripheral facial paralysis: 168 cases observation. Journal of Beijing College of Acupuncture ＆ Orthopedics. 7:31-33.

[2] 熊云，夏玉卿，王岱. (1994)电热针治疗脾胃虚寒型慢性萎性胃炎临床观察. 北京针灸骨伤学院 学报 1: 4-8.

[2] Xiong Yun, Xia Yu-qing, Wang Dai. (1994) Clinical observation of chronic atrophic gastritis with insufficiency of spleen-stomach treated by electrothermic needle. Journal of Beijing College of Acupuncture ＆ Orthopedics. 1:4-8

[3] 余伯亮，陈秋明. (2006)《内经》刺法治疗周围性面瘫临床观察. 上海针灸杂志25:19-20.

[3] Yu Bo-liang, Chen Qiu-ming. (2006）Clinical observations on the treatment of peripheral facial paralysis by “Internal Canon” needling method. SHANGHAI JOURNAL OF ACPUNCTURE AND MOXIBUSTION. 25:19-20.

[4] 朱沁，张丽萍，李胜利,[李激祥](http://202.201.7.11/kcms/detail/search.aspx?dbcode=CJFQ&sfield=au&skey=%C0%CF%E9&code=07400547;07409943;07400538;07409916;07412125;),[陈敏](http://202.201.7.11/kcms/detail/search.aspx?dbcode=CJFQ&sfield=au&skey=%B3%C2%C3%F4&code=07400547;07409943;07400538;07409916;07412125;)，等. (2003)B超动态观察针灸配合自主牵引治疗腰椎间盘突出症.上海针灸杂志 22:12-13.

[4] ZHU Qin, Zhang Li-ping, Li Sheng-li, Li JI-Xiang Chen Min，et al.(2003) Dynamic B-ultrasonographic Observations on the Treatment of Lumber Intervertebral Disc Protrusion by Acupuncture Plus Independent Traction. SHANGHAI JOURNAL OF ACUPUNCTURE AND MOXIBUSTION. 22:12-13.

[5] 邵伟立. (2008)阿是穴齐刺治疗枕神经痛疗效观察. 上海针灸杂志27:27-28.

[5] Shao Wei-li. (2008)Observations on the Efficacy of Triple Puncture of the Ashi Point in Treating Occipital Neuralgia. SHANGHAI JOURNAL OF ACUPUNCTURE AND MOXIBUSTION. 27:27-28.

[6] 朱才丰,杨骏, 费爱华, [俞红五](http://202.201.7.11/kcms/detail/search.aspx?dbcode=CJFQ&sfield=au&skey=%D3%E1%BA%EC%CE%E5&code=22974321;23385946;25232160;22961982;). (2010)艾灸督脉组穴治疗轻度认知功能障碍疗效观察. 上海针灸杂志29:695-697.

[6] Zhu Cai-feng, Yang Jun, Fei Ai-hua, Yu Hong-wu. (2010)Observations on the Efficacy of Moxibustion on Du Meridian Points in Treating Mild Cognitive Impairment. SHANGHAI JOURNAL OF ACUPUNCTURE AND MOXIBUSTION. 29:695-697.

[7] 李漾，杨华, 张丽，[郑德采](http://202.201.7.11/kcms/detail/search.aspx?dbcode=CJFQ&sfield=au&skey=֣%B5²%C9&code=06835269;24823548;23897703;10652761;).(2010)艾灸配合电针治疗感音神经性耳鸣疗效观察. 上海针灸杂志 29：521-522.

[7] LI Yang, YANG Hua, ZHANG Li, ZHENG De-cai. (2010)Observations on the Efficacy of Moxibustion plus Electroacupuncture in Treating Sensorineural Tinnitus. SHANGHAI JOURNAL OF ACUPUNCTURE AND MOXIBUSTION. 29：521-522.

[8] 揭桂莲. (2009)巴曲酶高压氧及针灸治疗突发性耳聋疗效观察. 上海针灸杂志 15：688-690.

[8]JIE Gui-lian. (2009)Clinical Observation of Batroxobin Combined Acwpuncture Point and Hyperbaric oxygen to Treat Sudden Deafness. SHANGHAI JOURNAL OF ACUPUNCTURE AND MOXIBUSTION. 15：688-690.

[9] 成汝梅. (1996)傍针刺治疗梨状肌综合征50例. 上海针灸杂志 15:147-148.

[9] Cheng Ru-mei. (1996)Treatment of piriformis syndrome with proximal needling:50 cases. SHANGHAI JOURNAL OF ACUPUNCTURE AND MOXIBUSTION. 15:147-148.

[10] 李磊, 陈汉平, 奚永江,[顾杰](http://202.201.7.11/kcms/detail/search.aspx?dbcode=CJFQ&sfield=au&skey=%B9˽%DC&code=21299531;21217128;21491931;21394438;). (1992)不同时辰针刺内关穴对收缩时间间期昼夜节律的影响. 上海针灸杂志 4：21-22.

[10]Li Lei, Chen Han-Ping, Xi Yong-jiang, Gu Jie. (1992Clinical Observation of Day and Night of STI treatment by Acupuncture on Neiguan point in Different Time. SHANGHAI JOURNAL OF ACUPUNCTURE AND MOXIBUSTION. 4：21-22.

[11] 金孟梓, 谢作群, 陈先威,[陈德秀](http://202.201.7.11/kcms/detail/search.aspx?dbcode=CJFQ&sfield=au&skey=%B3µ%C2%D0%E3&code=09412118;09415829;11393844;09376962;09415839;),[陈德沛](http://202.201.7.11/kcms/detail/search.aspx?dbcode=CJFQ&sfield=au&skey=%B3µ%C2%C5%E6&code=09412118;09415829;11393844;09376962;09415839;). (2008)刺络拔罐治疗中老年带状疱疹疗效观察. 上海针灸杂志 27：20-21.

[11] JIN Meng-zhi, XIE Zuo-qun, CHEN Xian-wei, CHEN De-xiu, CHEN De-pei. (2008)Observations on the Efficacy of Blood-letting Puncture and Cupping in Treating Middle-aged and Senile Herpes Zoster. SHANGHAI JOURNAL OF ACUPUNCTURE AND MOXIBUSTION. 27：20-21.

[12] 廖方容. (2009)刺络放血配合拔罐治疗颈性眩晕疗效观察. 上海针灸杂志 28：399-400.

[12] LIAO Fang-rong. (2009)Observations on the Efficacy of Pricking Bloodletting pins Cupping in Treating Cervical Vertigo. SHANGHAI JOURNAL OF ACUPUNCTURE AND MOXIBUSTION. 28：399-400.

[13] 周黎，周智梁.(2010)刺血疗法对脑梗死致偏身感觉障碍患者脑血流的影响. 上海针灸杂志 29：83-85.

[13]ZHOU Li, ZHOU Zhi-liang. (2010)Effect of Pricking Bloodletting Therapy on Cerebral Blood Flow in Patients with Cerebral Infarction-caused Hemidysesthesia. SHANGHAI JOURNAL OF ACUPUNCTURE AND MOXIBUSTION. 29：83-85

[14] 黄劲柏, 曾红科, 甄享凡，[孙诚](http://202.201.7.11/kcms/detail/search.aspx?dbcode=CJFQ&sfield=au&skey=%CB%EF%B3%CF&code=06801703;06803138;06801819;20658175;06802582;),[李辉](http://202.201.7.11/kcms/detail/search.aspx?dbcode=CJFQ&sfield=au&skey=%C0%EE%BB%D4&code=06801703;06803138;06801819;20658175;06802582;),. (2002)刺血疗法治疗急性脑梗塞近期疗效观察. 上海针灸杂志 21：7-8.

[14]Huang Jin-song, Zeng Hong-ke, Zhen Heng-fan, Song Cheng, Li Hui. (2002)Short-term Effect of First-aid Acupoint Pricking Blood Therapy on Acute Cerebral Infarction. SHANGHAI JOURNAL OF ACUPUNCTURE AND MOXIBUSTION. 21：7-8.

[15] 郭艳明, 顾钧青, 周帅亮，[许洁](http://202.201.7.11/kcms/detail/search.aspx?dbcode=CJFQ&sfield=au&skey=%D0%ED%BD%E0&code=11384739;11503496;24286187;25121682;).(2010)单穴注射治疗神经根型颈椎病疗效观察. 上海针灸杂志 29：650-651.

[15] GUO Yan-ming, GU Jun-qing, ZHOU Shuai-liang, XU Jie. (2010)Observations on the Efficacy of Single Acupuncture Point Injection in Treating Cervical Spondylotic Radiculopathy. SHANGHAI JOURNAL OF ACUPUNCTURE AND MOXIBUSTION. 21：7-8

[16] 李国安. (1996)电极板刺激穴位对催产的作用.上海针灸杂志 15：16.

[16] Li Guo-an. (1996)The oxytocic effect of stimulating points with electrode plate.SHANGHAI JOURNAL OF ACUPUNCTURE AND MOXIBUSTION. 21：7-8

[17] 钱奕胜. (1987)电针加红外线治疗腰膝痛106例疗效观察.上海针灸杂志 3,27-28

[17] Qian Yi-sheng. (1987)Observation on the treatment of waist-knee pain with electroacupuncture and infrared:106 cases. SHANGHAI JOURNAL OF ACUPUNCTURE AND MOXIBUSTION. 3,27-28

[18] 李圣平. (1992)电针配合穴位敷贴治疗面神经炎88例.上海针灸杂志1:6

[18] Li Sheng-ping. (1992)Will cooperate with acupoint sticking therapy facial paralysis: 88 cases. SHANGHAI JOURNAL OF ACUPUNCTURE AND MOXIBUSTION. 1:6

[19] 刘滨, 刘雪峰, 王红梅.(2010)电针配合注射玻璃酸钠治疗膝骨关节炎临床观察.上海针灸杂志29：593-595.

[19] LIU Bin, LIU Xue-feng, WANG Hong-mei. (2010)Clinical Observations on Electroacupuncture plus Injections of Sodium Hyaluronate for the Treatment of Knee Osteoarthritis. SHANGHAI JOURNAL OF ACUPUNCTURE AND MOXIBUSTION. 29：593-595

[20] 吴耀持, 张峻峰, 李石胜, [周景辉](http://202.201.7.11/kcms/detail/search.aspx?dbcode=CJFQ&sfield=au&skey=%D6ܾ%B0%BB%D4&code=23052483;22918201;23151514;22942184;23151513;25232172;),[张圣宏](http://202.201.7.11/kcms/detail/search.aspx?dbcode=CJFQ&sfield=au&skey=%D5%C5ʥ%BA%EA&code=23052483;22918201;23151514;22942184;23151513;25232172;),等.(2010)电针治疗急性腰扭伤的临床疗效与红外热像研究.上海针灸杂志 29：716-718.

[20] WU YAO-chi, ZHANG Jun-feng, LI Shi-sheng, ZHOU Jing-hui, ZHANG Sheng-hong. et al.(2010)Clinical Therapeutic Effect of Electroacupuncture on Acute Lumbar Sprain and Infrared Thermography Research. SHANGHAI JOURNAL OF ACUPUNCTURE AND MOXIBUSTION. 29：716-718.

[21] 刘洁，刘智艳. (2009)电针治疗肾精亏虚型轻度认知功能障碍临床观察. 上海针灸杂志 28：319-321.

[21]LIU Jie, LIU Zhi-yan. (2009)Clinical Observations on Electroacupuncture Treatment for Mild Cognitive Impairment of Kidney Essence Deficiency Type. SHANGHAI JOURNAL OF ACUPUNCTURE AND MOXIBUSTION. 28：319-321.

[22] 李岩峰, 郑晓, 车涛，[裘敏蕾](http://202.201.7.11/kcms/detail/search.aspx?dbcode=CJFQ&sfield=au&skey=%F4%C3%C3%F4%C0%D9&code=08627858;08630843;09674877;09674917;).(2010)电针治疗下颈椎不稳的椎动脉型颈椎病疗效观察. 上海针灸杂志 29：708-710.

[22]LI Yan-feng, ZHEN Xiao, CHE Tao, QIU Min-lei. (2010)Observations on the Therapeutic Effect of Electroacupuncture on Cervical Spondylotic Vertebral Arteriopathy with Lower Cervical Vertebral Instability. SHANGHAI JOURNAL OF ACUPUNCTURE AND MOXIBUSTION. 29：708-710.

[23] 陈尚杰, 朱芬, 董佳鸣, [梁明华](http://202.201.7.11/kcms/detail/search.aspx?dbcode=CJFQ&sfield=au&skey=%C1%BA%C3%F7%BB%AA&code=14203923;06806824;25232166;17329214;25232167;06806789;),[李书银](http://202.201.7.11/kcms/detail/search.aspx?dbcode=CJFQ&sfield=au&skey=%C0%EE%CA%E9%D2%F8&code=14203923;06806824;25232166;17329214;25232167;06806789;),等.(2010)调督补肾法治疗老年失眠症疗效观察. 上海针灸杂志 29：700-701.

[23]CHEN Shang-jie, ZHU fen, DONG Jia-ming, LIANG Ming-hua, LI Shu-ying, XU Qiong-yu. (2010)Observations on the Efficacy of Du-regulating and Kidney-Reinforcing Acupuncture in Treating Senile Insomnia.SHANGHAI JOURNAL OF ACUPUNCTURE AND MOXIBUSTION. 29：700-701

[24] 刘月芝. (2007)调神法治疗周围性面瘫100例临床观察. 上海针灸杂志 26:16-17.

[24]Liu Yue-zhi. (2007）Cinical observationof the divine law in treatment of surrounding facial paralysis SHANGHAI JOURNAL OF ACUPUNCTURE AND MOXIBUSTION. 26:16-17.

[25] 王凤荣,李秋平. (2006)督脉刮痧配刺血治疗急性乳腺炎的临床研究. 上海针灸杂志 25:22-23.

[25] WANG Feng-rong, LI Qiu-ping. (2006)Clinical Study of Treatment of Acute Mastitis by Dermal Scraping on Du Channel plus Blood-letting Puncture,.SHANGHAI JOURNAL OF ACUPUNCTURE AND MOXIBUSTION. 25:22-23.

[26] 吴奇方. (1999)短针浅刺法治疗面瘫60例. 上海针灸杂志 18:28.

[26]Wu Qi-fang. (1999) Observation on the curative effect of peripheral facial paralysis with the superficial needling treatment: 134 cases. SHANGHAI JOURNAL OF ACUPUNCTURE AND MOXIBUSTION. 18:28.

[27] 冯玉俊,吴奇方. (1996)多向透穴法治疗面瘫22例. 上海针灸杂志 15:115.

[27] Feng Yujun Wu Jifang. (1996) multidirectional through hole method treatment of facial paralysis:22cases. SHANGHAI JOURNAL OF ACUPUNCTURE AND MOXIBUSTION. 15:115.

[28] 王炜, 吴殷夏,唐唯岚. (2010)耳穴贴压加中药治疗糖调节受损的临床研究. 上海针灸杂志 29:94-96.

[28] WANG Wei, WU Yin-xia, TANG Wei-lan. (2010)Clinical Study of the Treatment of Impaired Glucose Regulation(IGR)by Auricular Point Plaster Therapy plus Chinese Herbal Medicine. SHANGHAI JOURNAL OF ACUPUNCTURE AND MOXIBUSTION. 29:94-96.

[29] 熊大武, 王黎, 刘承华，[陈涛](http://202.201.7.11/kcms/detail/search.aspx?dbcode=CJFQ&sfield=au&skey=%B3%C2%CC%CE&code=13985685;06939653;06936296;06933776;). (1996)耳穴压丸配合药流的临床观察. [上海针灸杂志](http://202.201.7.11/kns55/loginid.aspx?uid=MnRaRjI5cWl2b2hHTHFmL1NHNGkzQ1FYbTFQVlpXTFZFcGtwSlp1ZkFLUGlWSlE2&p=Navi/Bridge.aspx?LinkType=BaseLink&DBCode=cjfq&TableName=CJFQbaseinfo&Field=BaseID&Value=SHZJ) 4:18.

[29]Xiong Da-wu, Wang Li, Liu Bing-hua, Chen Tao. (1996)Ear pills with medicine flow clinical observation.SHANGHAI JOURNAL OF ACUPUNCTURE AND MOXIBUSTION. 4:18.

[30] 刘智艳，姚小红. (2005)耳针治疗青少年地方性甲状腺肿的临床研究. 上海针灸杂志24：3-4.

[30] LIU Zhi-yan, YAO Xiao-hong. (2005)Clinical Study on Ear Acupuncture Treatment of Juvenile Endemic Goiter. SHANGHAI JOURNAL OF ACUPUNCTURE AND MOXIBUSTION. 24：3-4.

[31] 周立武. (2009)燔针劫刺治疗肩峰下滑囊炎临床观察. 上海针灸杂志 28：406-407

[31]ZHOU Li-wu. (2009)Clinical Observations on Fire Needling Treatment for Subacromial Bursitis. SHANGHAI JOURNAL OF ACUPUNCTURE AND MOXIBUSTION. 28：406-407

[32] 陆菁,陈百先. (2000)放血疗法加中药对脑梗塞患者脑血流的影响. 上海针灸杂志 19：10-11.

[32]Lu Jing, Chen Bai-xian. (2000)Bloodletting therapy and the influence of cerebral blood flow in patients with cerebral infarction. SHANGHAI JOURNAL OF ACUPUNCTURE AND MOXIBUSTION. 19：10-11.

[33] 沈克艰，吴梅珍. (2005)伏天铺灸治疗慢性盆腔炎的临床研究. 上海针灸杂志 24:19-20.

[33] SHEN Ke-jian, WU Mei-zhen. (2005)Clinical Study on Treatment of Chronic Pelvic Inflammation by Long Snake Moxibustion in Dog Days. SHANGHAI JOURNAL OF ACUPUNCTURE AND MOXIBUSTION. 24:19-20.

[34] 李静.(2010)浮针配合腹针治疗腰椎间盘突出症疗效观察. 上海针灸杂志 29:532

[34]Li Jing. (2010)Cooperate with abdominal floating needle observed the curative effect of treatment of lumbar intervertebral disc protrusion. SHANGHAI JOURNAL OF ACUPUNCTURE AND MOXIBUSTION. 29:532

[35] 方震宇,郎伯旭.(2010)浮针配合针刺阳陵泉治疗肱二头肌长头肌肌腱炎疗效观察. 上海针灸杂志 29:787-788.

[35]FANG Zhen-yu, LANG Bo-xu. (2010)Observations on the Efficacy of Superficial Needling plus Acupuncture at Yanglingquan in Treating Tendinitis of Long Head of Biceps Brachii Muscle. SHANGHAI JOURNAL OF ACUPUNCTURE AND MOXIBUSTION. 29:787-788.

[36] 李璟,李洪珠. (1996)刮痧法治疗肩周炎30例. 上海针灸杂志 15：240.

[36]Li Jing, Li Hong-zhu. (1996)Scrapping method of treatment of periarthritis of shoulder..SHANGHAI JOURNAL OF ACUPUNCTURE AND MOXIBUSTION. 15：240

[37] 丛文杰, 方剑乔, 王晨瑶,[李邦伟](http://202.201.7.11/kcms/detail/search.aspx?dbcode=CJFQ&sfield=au&skey=%C0%EE%B0%EEΰ&code=25232168;15937584;16660853;25232169;25232170;),[石慧](http://202.201.7.11/kcms/detail/search.aspx?dbcode=CJFQ&sfield=au&skey=ʯ%BB%DB&code=25232168;15937584;16660853;25232169;25232170;). (2010)毫针的不同针灸疗法治疗肩关节周围炎疗效比较. 上海针灸杂志 29:713-715.

[37]CONG Wen-jie, FANG Jian-qiao, WANG Chen-yao, LI Bang-wei, SHI Hui. (2010)Comparison of the Therapeutic Effects of Filiform Needle-based Different Acupuncture Methods on Scapulohumeral Periarthritis. SHANGHAI JOURNAL OF ACUPUNCTURE AND MOXIBUSTION. 29:713-715.

[38] 金泽, 杨菲,王玉琳. (2010)合谷刺法配合局部注射治疗斑秃疗效观察. 上海针灸杂志 29:728-729.

[38]JIN Ze, YANG Fei, WANG Yu-lin. (2010)Observations on the Efficacy of Multi-direction Needling plus Local Injection in Treating Alopecia Areata. SHANGHAI JOURNAL OF ACUPUNCTURE AND MOXIBUSTION. 29:728-729.

[39] 盛明山. (1996)经皮穴位电刺激治疗周围性面神经麻痹. 上海针灸杂志 15：113-114.

[39] Sheng Ming-shan. (1996)Transcutaneous acupoint electrical stimulation treatment of surrounding facial nerve paralysis..SHANGHAI JOURNAL OF ACUPUNCTURE AND MOXIBUSTION. 15：113-114.

[40] 郑晓斌, 朱其广, 林远方， [刘特熹](http://202.201.7.11/kcms/detail/search.aspx?dbcode=CJFQ&sfield=au&skey=%C1%F5%CC%D8%EC%E4&code=23679015;06817576;06819349;23679014;).(2010)颈夹脊穴位注射配合卧位牵顿治疗神经根型颈椎病疗效观察. 上海针灸杂志 29:711-712.

[40] ZHENG Xiao-bin, ZHU Qi-guang, LIN Yuan-fang , LIU Te-xi. (2010)Observations on the Efficacy of Cervical Huatuo Jiaji Point Injection plus Decubital Manual Traction in Treating Cervical Spondylotic Radiculopathy. SHANGHAI JOURNAL OF ACUPUNCTURE AND MOXIBUSTION. 29:711-712.

[41]张晓华,艾群. (2010)列缺穴对椎动脉血流动力学影响的研究. 上海针灸杂志25:36-38

[41]ZHANG Xiao-hua, AI Qun. (2010)Study on the Influence of Point Lieque on Vertebroarterial Hemodynamics. SHANGHAI JOURNAL OF ACUPUNCTURE AND MOXIBUSTION. 29:711-712.

[42]徐志凤. (2006)埋线与中药综合治疗癫痫40例临床观察. 上海针灸杂志25:13-14.

[42]XU Zhi-feng. (2006)Clinical Observations on Treatment of Epileptic Seizure by Combind Catgut Embedding and Herbal Medicine：40 cases. SHANGHAI JOURNAL OF ACUPUNCTURE AND MOXIBUSTION. 25:13-14.

[43]黄亮,何俊.(2010)全息穴电针不同留针时间对脑梗死患者血液流变学的影响. 上海针灸杂志19:762-764.

[43]HUANG Liang, HE Jun. (2010)Temorheological Effects of Different Retention Times of Electroacupuncture at Holographic Points in Cerebral Infarction Patients. SHANGHAI JOURNAL OF ACUPUNCTURE AND MOXIBUSTION. 19:762-764.

[44]杨晓凌,倪立青. (2003)水针联合甲氨蝶呤治疗类风湿关节炎临床观察. 上海针灸杂志22:11-12.

[44]Yang Xiao-lin, Ni Li-qing. (2003)Treatment of Rheumatoid Arthritis by Acupoint Injection of Compound Angelica in Combination with Amethopterin. SHANGHAI JOURNAL OF ACUPUNCTURE AND MOXIBUSTION. 22:11-12.

[45]徐书华. (2008)斯奇康穴位注射配合中药治疗变应性鼻炎疗效观察. 上海针灸杂志27:16-17.

[45] XU Shu-hua. (2008)Treatment of Allergic Rhinitis by Point Injection of BCG Polysaccharide Nucleic Acid plus Oral Administration. SHANGHAI JOURNAL OF ACUPUNCTURE AND MOXIBUSTION. 27:16-17.

[46]李赟. (2006)陶道透身柱治疗睑腺炎120例临床观察. 上海针灸杂志25:18-19.

[46]Li Yun. (2006)Clinical observations on treatment of 120 hordeolum patients with Taodao-through-Shenzhu acupuncture. SHANGHAI JOURNAL OF ACUPUNCTURE AND MOXIBUSTION. 27:16-17.

[47]梁建情. (2010)头针顶颞前斜线治疗中枢性三叉神经痛疗效观察. 上海针灸杂志29:528.

[47]Liang Jian-qing. (2010)The needle tip temporal front slash the central trigeminal neuralgia treated by observation. SHANGHAI JOURNAL OF ACUPUNCTURE AND MOXIBUSTION. 29:528.

[48]夏阳, 朱天忠, 宋亚光, [杨林](http://202.201.7.11/kcms/detail/search.aspx?dbcode=CJFQ&sfield=au&skey=%D1%EE%C1%D6&code=06436101;06429381;08784674;08117067;07842660;08784687;08784682;08784680;08784685;05982906;),[冯祯钰](http://202.201.7.11/kcms/detail/search.aspx?dbcode=CJFQ&sfield=au&skey=%B7%EB%EC%F5%EE%DA&code=06436101;06429381;08784674;08117067;07842660;08784687;08784682;08784680;08784685;05982906;),等. (2004)头针结合CT影像定位治疗缺血性中风80例临床观察. 上海针灸杂志23:10-12.

[48]Xia Yang, Zhu Tian-zhong, Song Ya-guang, Yang Zhen-yu. et al. (2004)Clinical Observation on the Treatment of 80 Ischemic Stroke Cases by Scalp Acupuncture Plus CT Image Location. SHANGHAI JOURNAL OF ACUPUNCTURE AND MOXIBUSTION. 23:10-12.

[49]赵玉侠. (1999)温针灸器在妇产科手术后的应用. 上海针灸杂志18:16

[49]Zhao Yu-xia. (1999)Temperature of the application of acupuncture and moxibustion apparatus in gynecology and obstetrics surgery. SHANGHAI JOURNAL OF ACUPUNCTURE AND MOXIBUSTION. 18:16

[50]李赟. (2016)悬灸三阴交治疗胎位不正临床观察. 上海针灸杂志25:11-12.

[50]Li Yun. (2006)Clinical Observations on Treatment of Fetal Malposition by Suspended Moxibustion over Sanyinjiao. SHANGHAI JOURNAL OF ACUPUNCTURE AND MOXIBUSTION. 25:11-12.

[51]练汉健, 孔令深,黄柳和. (2000)穴位磁疗法治疗中风偏瘫的临床观察. 上海针灸杂志19:18-19.

[51]Lian Han-jian, Kong Li-shen, Huang Liu-he. (2000)Clinical observation of acupuncture point magnetic therapy treatments for stroke hemiplegia. SHANGHAI JOURNAL OF ACUPUNCTURE AND MOXIBUSTION. 19:18-19.

[52]闫怀士. (2010)穴位注射干扰素治疗带状疱疹疗效观察. 上海针灸杂志19:780-781.

[52]Yan Huai-shi. (2010)Observations on the Efficacy of Acupuncture Point Injection of Interferon in Treating Herpes Zoster. SHANGHAI JOURNAL OF ACUPUNCTURE AND MOXIBUSTION. 19:780-781.

[53]马民玉, 李红, 马民瑞,[马君志](http://202.201.7.11/kcms/detail/search.aspx?dbcode=CJFQ&sfield=au&skey=%C2%ED%BE%FD־&code=07267506;07273575;07263682;07263679;). (2001)穴位注射减轻人流术后综合征的临床观察. 上海针灸杂志20:16-17.

[53]Ma Ming-yu, Li Hong, Ma Ming-rui, Ma Jun-zhi. (2001)CLINICAL OBSERVATION OF ACUPOINT-INJECTION IN REDUCING COMPREHENSIVE REACTION AFTER ARTIFICIAL ABORTION. SHANGHAI JOURNAL OF ACUPUNCTURE AND MOXIBUSTION. 20:16-17.

[54]何颖妉,何方红. (2008)穴位注射结合抗抑郁药物治疗抑郁症的临床观察. 上海针灸杂志27:15-16.

[54]HE Ying-dan, HE Fang-hong. (2008)Clinical Observations on Treatment of Depression by Point Injection plus Antidepressants.SHANGHAI JOURNAL OF ACUPUNCTURE AND MOXIBUSTION. 27:15-16.

[55]陶玉东, 王东雁, 贺天喜, [于春梅](http://202.201.7.11/kcms/detail/search.aspx?dbcode=CJFQ&sfield=au&skey=%D3ڴ%BA÷&code=15590779;05979152;06726334;17519970;). (2009)穴位注射配合推拿治疗颈源性头痛疗效观察. 上海针灸杂志28:332-333.

[55]TAO Yu-dong, WANG Dong-yan, HE Tian-xi, YU Chun-mie. (2009)Observations on the Efficacy of Acupuncture Point Injection plus Manipulation in Treating Cervical Headache. SHANGHAI JOURNAL OF ACUPUNCTURE AND MOXIBUSTION. 28:332-333.

[56]王少贞,朱首豪. (2008)穴位注射配温灸治疗变应性鼻炎临床观察. 上海针灸杂志 27:11-12.

[56]WANG Shao-zhen, ZHU Shou-hao. (2008)Clinical Observations on the Efficacy of Point Injection pins Gentle Moxibustion in Treating Allergic Rhinitis.SHANGHAI JOURNAL OF ACUPUNCTURE AND MOXIBUSTION. 27:11-12.

[57]来心平. (1990)穴位注射治疗腱鞘炎102例. 上海针灸杂志 1:22.

[57]Lai Xin-ping. (1990)Point injection treatment of 102 cases of tenosynovitis. SHANGHAI JOURNAL OF ACUPUNCTURE AND MOXIBUSTION. 27:11-12.

[58]王学诗. (1998)穴位注射治疗咳嗽变异性哮喘32例. 上海针灸杂志17:15-16.

[58]Wang Xue-shi. (1998)Point injection treatment of 32 cases of cough variant asthma. SHANGHAI JOURNAL OF ACUPUNCTURE AND MOXIBUSTION. 17:15-16.

[59]程世平,周厚荣,吴玉坤. (1999)穴位注射治疗慢性肾小球肾炎的疗效观察. 上海针灸杂志18:8-9.

[59]Cheng Shi-ping, Zhou Hou-rong, Wu Yu-kong. (1999)Curative effect observation of point injection treatment of chronic glomerulonephritis.SHANGHAI JOURNAL OF ACUPUNCTURE AND MOXIBUSTION. 18:8-9.

[60]刘媛媛,庄素卿. (2010)穴位注射治疗月经过少临床观察. 上海针灸杂志 29:776-777.

[60]LIU Yuan-yuan, ZHUANG Su-qing. (2010)Clinical Investigation on the Treatment of Oligomenorrhea by Point Injection.SHANGHAI JOURNAL OF ACUPUNCTURE AND MOXIBUSTION. 18:8-9.

[61]王鹏琴,王健,周鸿飞. (2008)眼针对急性脑梗死患者神经功能缺损及血浆纤维蛋白原水平的影响. 上海针灸杂志 27:5-7.

[61]WANG Peng-qin, WANG Jian, ZHOU Hong-fei. (2008)Influences of Eye Acupuncture on Neural Deficit and Plasma Fibrinogen Level in Acute Cerebral Infarction Patients. SHANGHAI JOURNAL OF ACUPUNCTURE AND MOXIBUSTION. 27:5-7.

[62]陈晓军,方针,罗高权. (2009)养血补脑针刺方治疗气血不足型椎基底动脉供血不足的临床观察. 上海针灸杂志 26:211-212.

[62]ZHANG Xiu-guo, ZHANG Yue. (2009)Effect of Yangxue Bunao acupuncture prescription in treating insufficiency of blood supply through vertebro-basilar artery in patients with insufficiency of vital energy and blood. SHANGHAI JOURNAL OF ACUPUNCTURE AND MOXIBUSTION. 26:211-212

[63]陈跃来,岑珏,侯文光, [虞先敏](http://202.201.7.11/kcms/detail/search.aspx?dbcode=CJFQ&sfield=au&skey=%D3%DD%CF%C8%C3%F4&code=22992769;22711388;22761116;23064672;22827233;),[马雪梅](http://202.201.7.11/kcms/detail/search.aspx?dbcode=CJFQ&sfield=au&skey=%C2%EDѩ÷&code=22992769;22711388;22761116;23064672;22827233;). (2007)腰骶段穴位对不稳定膀胱调节的穴位特异性研究. 上海针灸杂志 26:6-8.

[63]CHEN Yue-lai, CHEN Ju , HOU Weng-nang, YU Xian-min. (2007)Study of the Specificity of Lumbosacral Points in Regulating Unstable BladderMA Xue-mei. SHANGHAI JOURNAL OF ACUPUNCTURE AND MOXIBUSTION. 26:6-8.

[64]鲍春龄,袁萍,李立秋. (2008)阴阳气血配穴法针刺治疗慢性疲劳综合征临床观察. 上海针灸杂志 27:11-12.

[64]BAO Chun-ling, YUAN Ping, LI Li-qiu. (2008)Clinical Observations on Treatment of Chronic Fatigue Syndrome with Acupuncture Based on Yin-yang and Qi-blood Prescription of Points. SHANGHAI JOURNAL OF ACUPUNCTURE AND MOXIBUSTION. 27:11-12.

[65]欧阳泠星,吴远华,苏红梅,[吴邦启](http://202.201.7.11/kcms/detail/search.aspx?dbcode=CJFQ&sfield=au&skey=%CE%E2%B0%EE%C6%F4&code=11528691;06937669;10846283;23439820;06940170;),[朱广旗](http://202.201.7.11/kcms/detail/search.aspx?dbcode=CJFQ&sfield=au&skey=%D6%EC%B9%E3%C6%EC&code=11528691;06937669;10846283;23439820;06940170;). (2009)阴阳透刺法对急性脑梗死患者t-PA及PAI-1的影响. 上海针灸杂志 28:504-506.

[65]OUYANG Ling-xing, WU Yuan-hua, SU Hong-mei, WU Bang-qi, ZHU Guang-qi. (2009)Study of the Influence of Yin-yang Penetration Needling Method on T-PA and PAI-1 in Patients with Acute Cerebral Infarction. SHANGHAI JOURNAL OF ACUPUNCTURE AND MOXIBUSTION 28:504-506.

[66]孙远征,李虹霖. (2006)俞募通经配穴法治疗慢性疲劳综合征疗效观察. 上海针灸杂志 25:3-4.

[66]SUN Yuan-zheng, LI Hong-lin. (2006)Observations on the curative effect of channel-unblocking Back-Shu and Front-Mu points prescription on chronic fatigue syndrome. SHANGHAI JOURNAL OF ACUPUNCTURE AND MOXIBUSTION. 25:3-4.

[67]李滋平,闫晓燕.(2010)俞原配穴法针刺治疗血管性痴呆临床观察. 上海针灸杂志 29(4):227-228.

[67]LI Zi-ping, YAN Xiao-yan. (2010)Clinical Observations on the Treatment of Vascular Dementia by Transport-source Point Combination. SHANGHAI JOURNAL OF ACUPUNCTURE AND MOXIBUSTION 29(4):227-228.

[68]杨自威. (2004)远近相伍取穴治疗椎动脉型颈椎病临床观察. 上海针灸杂志 23:19-21

[68]Yang Zi-wei. (2004)Clinical Observations on the Treatment of Vertebroarterial Cervical Spondylopathy by Selection of Compatible Adjacent and Distant Points. SHANGHAI JOURNAL OF ACUPUNCTURE AND MOXIBUSTION 23:19-21

[69]赵宇,刘新桥,李岩. (2007)早期针刺对心脏骤停患者脑复苏的影响. 上海针灸杂志 26:7-9.

[69]ZHAO Yu, LIU Xin-qiao, LI Yan. (2007)Influence of Early Acupuncture on Cerebral Resuscitation in Cardiac Arrest Patients. SHANGHAI JOURNAL OF ACUPUNCTURE AND MOXIBUSTION 26:7-9.

[70]沙岩. (2007)针砭罐综合疗法治疗颈型颈椎病疗效观察. 上海针灸杂志 26:19-20.

[70]SHA Yan. (2007)Treatment of Neck-type Cervical Spondylopathy by A Composite Treatment with Acupuncture, Stone Scraping and Cupping. SHANGHAI JOURNAL OF ACUPUNCTURE AND MOXIBUSTION 26:7-9.

[71]王俊华,周嘉澄,刘刚.(2010)针刺背俞穴对中风偏瘫患者运动功能的影响. 上海针灸杂志 29:770-772.

[71]WANG Jun-hua, ZHOU Jia-cheng, LIU Gang. (2010)Effect of Acupuncture at Back-Shu Points on Motor Function in Hemiplegic Stroke Patients.S HANGHAI JOURNAL OF ACUPUNCTURE AND MOXIBUSTION 29:770-772.

[72]黄凡,刘悦,姚国新,[周飞雄](http://202.201.7.11/kcms/detail/search.aspx?dbcode=CJFQ&sfield=au&skey=%D6ܷ%C9%D0%DB&code=06767968;06758883;06762090;17719464;17719465;17719466;),[王小寅](http://202.201.7.11/kcms/detail/search.aspx?dbcode=CJFQ&sfield=au&skey=%CD%F5С%D2%FA&code=06767968;06758883;06762090;17719464;17719465;17719466;),等. (2008)针刺背俞穴为主治疗缺血性中风临床观察. 上海针灸杂志 27:4-7.

[72]HUANG Fan, LIU Yue, YAO Guo-xin, ZHOU Fei-xiong, WANG Xiao-yin, YANG Dong. (2008)Clinical Observations on Treatment of Ischemic Stroke with Acupuncture at Back-Shu Points. SHANGHAI JOURNAL OF ACUPUNCTURE AND MOXIBUSTION 27:4-7

[73]王茵萍,龚传美. (1995)针刺不同耳穴对胆囊收缩功能的影响. 上海针灸杂志 14:199.

[73]Wang Yin-ping, Gong Chuan-mei. The influence of different acupuncture points on the gallbladder contraction function. SHANGHAI JOURNAL OF ACUPUNCTURE AND MOXIBUSTION 14:199.

[74]蔡玉梅,黄文燕,郑继范,[吴毅](http://202.201.7.11/kcms/detail/search.aspx?dbcode=CJFQ&sfield=au&skey=%CE%E2%D2%E3&code=08618494;08595579;17515028;06721622;). (2010)针刺从脾胃论治神经根型颈椎病疗效观察. 上海针灸杂志 29:451-453.

[74]CAI Yu-mei, HUANG Wen-yan, ZHEN Ji-fan, WU Yi. (2010)Observations on the Effect of Spleen and Stomach Theory-based Treatment on Cervical Spondylotic Radiculopathy. SHANGHAI JOURNAL OF ACUPUNCTURE AND MOXIBUSTION 29:451-453.

[75]陈少宗,侯文静,丛茜. (2010)针刺单穴与多穴治疗痛经时止痛作用时效规律的比较. 上海针灸杂志 29:623-625.

[75]CHEN Shao-zong, HOU Wen-jing, CONG Xi. (2010)Comparison of the Analgesic Time-effect Regularities of Acupuncture at a Single Point and at Multiple Points in Treating Dysmenorrhea. SHANGHAI JOURNAL OF ACUPUNCTURE AND MOXIBUSTION 29:623-625.

[76]胡智海,王毅. (2010)针刺对非胰岛素抵抗型多囊卵巢综合征作用机理的研究. 上海针灸杂志 28:197-198.

[76]HU Zhi-hai, WANG Yi. (2010)Study of the Mechanism of Acupuncture Action on Polycystic Ovary Syndrome of Non-insulin Resistance Type, SHANGHAI JOURNAL OF ACUPUNCTURE AND MOXIBUSTION 28:197-198.

[77]吕德,杜兰民,任庆霞,[李相利](http://202.201.7.11/kcms/detail/search.aspx?dbcode=CJFQ&sfield=au&skey=%C0%EE%CF%E0%C0%FB&code=08310924;14397284;08357165;09067813;08305028;),[张爱田](http://202.201.7.11/kcms/detail/search.aspx?dbcode=CJFQ&sfield=au&skey=%D5Ű%AE%CC%EF&code=08310924;14397284;08357165;09067813;08305028;). (1993)针刺对肛门疾病术后止痛的疗效观察. 上海针灸杂志 12:72.

[77]Lv De, Du Lan-ming, Ren Qing-xia, Li Xiang-li, Zhang Ai-tian. (1993)Acupuncture to the anus disease curative effect observation of postoperative pain. SHANGHAI JOURNAL OF ACUPUNCTURE AND MOXIBUSTION 12:72.

[78]周继人,范德培,杜玉珍,[尤海清](http://202.201.7.11/kcms/detail/search.aspx?dbcode=CJFQ&sfield=au&skey=%D3Ⱥ%A3%C7%E5&code=07828801;10919146;14400706;14949259;). (1992)针刺对扩张型心肌病心衰病人左心功能及体液内分泌的影响. 上海针灸杂志,11:3-4.

[78]Zhou Ji-ren, Fan De-pei, Du Yu-zhen, You Hai-qing. (1992)Acupuncture for DCM heart failure patients with left heart function and the influence of body fluids, endocrine. SHANGHAI JOURNAL OF ACUPUNCTURE AND MOXIBUSTION , 11:3-4.

[79]史春娟. (2008)针刺分类治疗泌尿系结石疼痛疗效观察. 上海针灸杂志 27:21-22.

[79]SHI Chun-juan. (2008)Observations on the Efficacy of Acupuncture in Treating Different Types of Pains Due to Urinary Calculus. SHANGHAI JOURNAL OF ACUPUNCTURE AND MOXIBUSTION 27:21-22.

[80]刘明,任燕,殷亚楠. (2009)针刺和理疗联合应用在脑卒中早期康复中的疗效观察. 上海针灸杂志 28:77-79.

[80]LIU Ming, REN Yan, YIN Ya-nan. (2009)Observations on the Therapeutic Effect of Combined Use of Acupuncture and Physiotherapy in the Early Convalescence of Cerebral Stroke Patients. SHANGHAI JOURNAL OF ACUPUNCTURE AND MOXIBUSTION 28:77-79.

[81]黄海明，高强. (2003)针刺加TDP为主治疗颞下颌关节综合征疗效观察. 上海针灸杂志 22:23-24.

[81]Huang Hai-ming, Gao Qiang. (2003)Observations on the Effect of Acupuncture Plus TDP as Main Therapy in Treatment of Temporomandibular Joint Syndrome. SHANGHAI JOURNAL OF ACUPUNCTURE AND MOXIBUSTION 22:23-24.

[82]芮兴国.(2010)针刺加叩刺拔罐治疗臀上皮神经炎疗效观察. 上海针灸杂志 29(8):515-516.

[82]RUI Xing-guo. (2010)Observations on the Efficacy of Acupuncture plus Cutaneous Needle Tapping in Treating Inflammation of Superior Clunial Nerves. SHANGHAI JOURNAL OF ACUPUNCTURE AND MOXIBUSTION 29(8):515-516.

[83]赵欢初,曹媛,乔光甫,[李欣](http://202.201.7.11/kcms/detail/search.aspx?dbcode=CJFQ&sfield=au&skey=%C0%EE%D0%C0&code=20393571;20441875;20784788;20530969;). (1997)针刺加闪罐治疗周围性面神经麻痹58例. 上海针灸杂志 16:24-25.

[83]Zhao Huan-chu, Cao Yuan, Qiao Guang-pu, Li Xin. (1997)Acupuncture plus flash tank in treatment of around 58 cases of facial nerve paralysis. SHANGHAI JOURNAL OF ACUPUNCTURE AND MOXIBUSTION 16:24-25.

[84]阎兴洲,冯玲媚. (2009)针刺加咽后壁点刺治疗中风后假性球麻痹疗效观察. 上海针灸杂志 28:274-275.

[84]YAN Xin-zhou, FENG Ling-mei. (2009)Observations on the Efficacy of Acupuncture plus Pricking on the Posterior Pharyngeal Flap in Treating Postapoplectic Pseudobulbar Palsy. SHANGHAI JOURNAL OF ACUPUNCTURE AND MOXIBUSTION 28:274-275.

[85]付磊,刘未艾,吴清明,[李向荣](http://202.201.7.11/kcms/detail/search.aspx?dbcode=CJFQ&sfield=au&skey=%C0%EE%CF%F2%C8%D9&code=22474190;10975007;11185497;11061286;10944610;11126291;10966134;),[李丹丹](http://202.201.7.11/kcms/detail/search.aspx?dbcode=CJFQ&sfield=au&skey=%C0%B5%A4&code=22474190;10975007;11185497;11061286;10944610;11126291;10966134;)等. (2009)针刺结合刺络拔罐治疗脑卒中后肩手综合征疗效观察. 上海针灸杂志 28:132-134.

[85]FU Lei, LIU Wei-ai, WU Qing-ming, LI Xiang-rong, LI Da-da, SHI Xue-hui,YANG Zhi-hui. (2009)Observations on the Efficacy of Acupuncture Plus Pricking-cupping Bloodletting in Treating Postapoplectic Shoulder-hand Syndrome. SHANGHAI JOURNAL OF ACUPUNCTURE AND MOXIBUSTION 28:132-134.

[86]张永臣. (2009)针刺结合华佗药枕对颈椎病患者颈椎生理曲度的影响. 上海针灸杂志 28:151-153.

[86]ZHANG Yong-cheng. (2009)Influence of Acupuncture Plus Huatuo Medicinal Pillow on the Physiologic Curvature of Cervical Spine in Cervical Spondylosis Patients. SHANGHAI JOURNAL OF ACUPUNCTURE AND MOXIBUSTION 28:151-153.

[87]贾红玲,张永臣. (2006)针刺结合康复训练治疗中风后吞咽障碍40例. 上海针灸杂志 25:5-6.

[87]JIA Hong-ling, ZHANG Yong-cheng. (2006)Treatment of 40 postapoplectic dysphagia patients by acupuncture plus rehabilitation training. SHANGHAI JOURNAL OF ACUPUNCTURE AND MOXIBUSTION 25:5-6.

[88]周晓平,何锦添. (2007)针刺结合龙氏手法对神经根型颈椎病的镇痛作用研究. 上海针灸杂志 26:12-14.

[88]Zhou Xiao-ping, He Jin-ping. (2007)Study on the Analgesic Effect of Acupuncture Plus Long's Manipulation on Nerve root-type Cervical Spondylopathy. SHANGHAI JOURNAL OF ACUPUNCTURE AND MOXIBUSTION 26:12-14.

[89]顾忠平. (2007)针刺结合水针治疗腰背肌筋膜炎临床观察. 上海针灸杂志 1:4-9.

[89]CU Zhong-ping. (2007)Clinical observations on treatment of lumbodorsal myofascitis by acupuncture plus point injection. SHANGHAI JOURNAL OF ACUPUNCTURE AND MOXIBUSTION 1:4-9.

[90]李萍,孙国锋,姚桂棉,[阎如玉](http://202.201.7.11/kcms/detail/search.aspx?dbcode=CJFQ&sfield=au&skey=%D1%D6%C8%E7%D3%F1&code=07116153;07122034;11345986;11345985;11345984;07112987;),[夏梅华](http://202.201.7.11/kcms/detail/search.aspx?dbcode=CJFQ&sfield=au&skey=%CF%C4÷%BB%AA&code=07116153;07122034;11345986;11345985;11345984;07112987;),等. (2008)针刺联合康复治疗脑卒中后吞咽障碍的临床观察. 上海针灸杂志 127:10-11.

[90]LI Ping, SUN Guo-feng, YAO Gui-mian, YAN Ru-yu, XIA Mei-hua, ZHANG Wen-jun. (2008)Clinical Observations on Treatment of Pseudobulbar Palsy Induced Dysphagia by Acupuncture plus Rehabilitation Therapy.SHANGHAI JOURNAL OF ACUPUNCTURE AND MOXIBUSTION 127:10-11.

[91]张立峰 ,王晓臣.(2009)针刺疗法对2型糖尿病患者高脂血症临床研究. 上海针灸杂志 28:623-625.

[91ZHANG Li-feng. (2009)WANG Xiao-chengClinical Study of Acupuncture Treatment for Hyperlipidemia in Patients with Type 2 Diabetes Mellitus. SHANGHAI JOURNAL OF ACUPUNCTURE AND MOXIBUSTION 28:623-625.

[92]罗丽平,沈仲元,陈汉平,[余平](http://202.201.7.11/kcms/detail/search.aspx?dbcode=CJFQ&sfield=au&skey=%D3%E0ƽ&code=22481824;08653667;09674818;09698223;). (2009)针刺内关-间使对健康人心脏自主神经的调节作用. 上海针灸杂志 28:603-606.

[92]LUO Li-ping, SHEN Zhong-yuan, CHEN Han-ping, YU Ping. (2009)Regulating Effect of Acupuncture at Neiguan-Jianshi on the Cardiac Autonomic Nerve in Healthy Persons. SHANGHAI JOURNAL OF ACUPUNCTURE AND MOXIBUSTION 28:603-606.

[93]朱芬,陈尚杰,查思想,[黄石钊](http://202.201.7.11/kcms/detail/search.aspx?dbcode=CJFQ&sfield=au&skey=%BB%C6ʯ%EE%C8&code=06806824;14203923;22594237;06806530;). (2009)针刺配合康复训练对中风后肩手综合征疗效观察. 上海针灸杂志 28:272-273.

[93]ZHU Fen, CHEN Shang-jie, CHA Si-xiang, HUANG Shi-zhao. (2009)Observations on the Efficacy of Acupuncture plus Rehabilitation Training in Treating Postapoplectic Shoulder-hand Syndrome. SHANGHAI JOURNAL OF ACUPUNCTURE AND MOXIBUSTION 28:272-273.

[94]李红星,岳国荣,刘东坡,[周红芳](http://202.201.7.11/kcms/detail/search.aspx?dbcode=CJFQ&sfield=au&skey=%D6ܺ췼&code=14028347;17465070;23233228;20352384;). (2009)针刺配合康复训练改善脑卒中后吞咽障碍临床观察. 上海针灸杂志 28:388-389.

[94]Li Hong-xing, Yue Guo-rong, Liu Dong-po, Zhou Hong-fang. (2009)Clinical Observations on Acupuncture plus Rehabilitation Training for Improving Postapoplectic Dysphagia LI Hong-xing1. SHANGHAI JOURNAL OF ACUPUNCTURE AND MOXIBUSTION 28:388-389.

[95]倪欢欢,胡永善,崔晓, [吴毅](http://202.201.7.11/kcms/detail/search.aspx?dbcode=CJFQ&sfield=au&skey=%CE%E2%D2%E3&code=17380726;05965851;11486940;06721622;25283202;16155903;23744776;24389001;22653852;),[黄德权](http://202.201.7.11/kcms/detail/search.aspx?dbcode=CJFQ&sfield=au&skey=%BBƵ%C2Ȩ&code=17380726;05965851;11486940;06721622;25283202;16155903;23744776;24389001;22653852;)等.(2010)针刺配合康复训练治疗脑卒中后上肢痉挛临床观察. 上海针灸杂志 29:767-769.

[95]NI Huan-huan, HU Yong-shan, CUI Xiao, WU Yi, HUANG De-quan, et al. (2010)Clinical Observation on the Treatment of Post-stroke Upper Limb Spasticity by Acupuncture plus Rehabilitation. SHANGHAI JOURNAL OF ACUPUNCTURE AND MOXIBUSTION 29:767-769.

[96]裴君. (2010)针刺配合康复训练治疗脑卒中偏瘫疗效观察. 上海针灸杂志 29:428-430

[96]PEI Jun. (2010)Observations on the Therapeutic Effect of Acupuncture plus Rehabilitation on Hemiplegic Stroke. SHANGHAI JOURNAL OF ACUPUNCTURE AND MOXIBUSTION 29:767-769.

[97]李国臣,赵新. (2006)针刺配合康复治疗脑卒中失语的临床观察. 上海针灸杂志 25:13-14.

[97]LI Guo-chen, ZHAO Xin. (2006)Clinical observations on treatment of apoplectic aphasia by acupuncture plus rehabilitation. SHANGHAI JOURNAL OF ACUPUNCTURE AND MOXIBUSTION 29:767-769.

[98]刘淳. (2007)针刺配合牵引治疗椎动脉型颈椎病的临床观察. 上海针灸杂志 26:26-28.

[98]LIU Chun. (2007)Clinical Observations on Treatment of Cervical Spondylopathy of Vertebral Artery Type by Acupuncture plus Traction. SHANGHAI JOURNAL OF ACUPUNCTURE AND MOXIBUSTION 26:26-28.

[99]郄玉兰. (2005)针刺配合闪罐治疗儿童多动症的临床观察. 上海针灸杂志 24:13-14.

[99]QIE Yu-lan. (2005)Clinical Observations on the Treatment of Child Hyperkinetic Syndrome by Acupunctrue plus Flash Cupping. SHANGHAI JOURNAL OF ACUPUNCTURE AND MOXIBUSTION 24:13-14.

[100]孙奎,鲍学梅,周忠良, [宋阳春](http://202.201.7.11/kcms/detail/search.aspx?dbcode=CJFQ&sfield=au&skey=%CB%CE%D1%F4%B4%BA&code=22867880;24190725;22974215;22847457;22811361;),[刘德春](http://202.201.7.11/kcms/detail/search.aspx?dbcode=CJFQ&sfield=au&skey=%C1%F5%B5´%BA&code=22867880;24190725;22974215;22847457;22811361;).(2010)针刺配合手法治疗膝骨关节炎疗效观察. 上海针灸杂志 29:181-183.

[100]SUN Kui, BAO Xue-mei, ZHOU Zhong-liang, SONG Yang-chun, (2010)LIU De-chunObservations on the Efficacy of Acupuncture plus Manipulation in Treating Knee Osteoarthritis. SHANGHAI JOURNAL OF ACUPUNCTURE AND MOXIBUSTION 29:181-183.

[101]孙奎, 杨永晖, 罗建明, [刘德春](http://202.201.7.11/kcms/detail/search.aspx?dbcode=CJFQ&sfield=au&skey=%C1%F5%B5´%BA&code=22867880;22811361;22928729;22823759;). (2007)针刺配合推拿治疗膝骨关节炎的临床观察. 上海针灸杂志 26:11-12.

[101]SUN Ku, YANG Yong-hui, LUO Jian-ming, LIU De-chun. (2007)Clinical Observations On Treatment of Genual Osteoarthritis by Acupuncture plus Massotherapy. SHANGHAI JOURNAL OF ACUPUNCTURE AND MOXIBUSTION 26:11-12.

[102]俞冬生, 方周林, 王冬梅,[李玲](http://202.201.7.11/kcms/detail/search.aspx?dbcode=CJFQ&sfield=au&skey=%C0%EE%C1%E1&code=06176450;06176448;15341617;06176455;),[张锐](http://202.201.7.11/kcms/detail/search.aspx?dbcode=CJFQ&sfield=au&skey=%D5%C5%C8%F1&code=06176450;06176448;15341617;06176455;). (1996)针刺配合推拿治疗周围性面瘫53例. 上海针灸杂志 15:134.

[102]Yu Dong-sheng, Fang Zhou-lin, Wang Dongp-mai, Li ling,Zhang Rui. (1996）Around of massage and acupuncture for the treatment of 53 cases of facial paralysis. SHANGHAI JOURNAL OF ACUPUNCTURE AND MOXIBUSTION 15:134.

[103]彭支莲, 黄剑. (2009)针刺配合推拿治疗椎动脉型颈椎病疗效观察. 上海针灸杂志 28:29-30.

[103]PENG Zhi-lian, HUANG Jian. (2009)Observations on the Efficacy of Acupuncture plus Massotherapy in Treating Vertebroarterial Cervical Spondylopathy. SHANGHAI JOURNAL OF ACUPUNCTURE AND MOXIBUSTION 28:29-30.

[104]郑智, 文胜, 蔡章健,[段俊峰](http://202.201.7.11/kcms/detail/search.aspx?dbcode=CJFQ&sfield=au&skey=%B6ο%A1%B7%E5&code=06768196;15461956;15238792;05977021;). (2008)针刺配合推拿治疗椎动脉型颈椎病临床观察. 上海针灸杂志 27:17-19.

[104]ZHENG Zhi, WEN Sheng, CAI Zhang-jian, DUAN Jun-feng. (2008)Clinical Study of Treatment of Cervical Spondylotic Vertebral Arteriopathy by Acupuncture plus Massotherapy. SHANGHAI JOURNAL OF ACUPUNCTURE AND MOXIBUSTION 27:17-19.

[105]陶玉东, 杜建梅, 王桂英,[杨铖](http://202.201.7.11/kcms/detail/search.aspx?dbcode=CJFQ&sfield=au&skey=%D1%EE%EE%F1&code=15590779;24190720;06736730;06726394;24190721;22536455;),[李锋](http://202.201.7.11/kcms/detail/search.aspx?dbcode=CJFQ&sfield=au&skey=%C0%EE%B7%E6&code=15590779;24190720;06736730;06726394;24190721;22536455;),等.(2010)针刺配合退热口服液治疗外感发热疗效观察. 上海针灸杂志 29:158-160.

[105]TAO Yu-dong, DU Jian-mei, WANG Gui-ying, YANG Cheng, LI Feng, LIU Xiao-fu. (2010)Observations on the Efficacy of Acupuncture plus Antipyretic Oral Liquid in Treating Exopathic Fever. SHANGHAI JOURNAL OF ACUPUNCTURE AND MOXIBUSTION 29:158-160.

[106]汪令崇. (1997)针刺配合穴位注射治疗网球肘30例. 上海针灸杂志 16:20.

[106]Wang Ling-chong. (1997)30 cases of point injection and acupuncture for the treatment of tennis elbow. SHANGHAI JOURNAL OF ACUPUNCTURE AND MOXIBUSTION 29:158-160.

[107]陶红星, 谷风. (2009)针刺配合中药治疗慢性盆腔炎疗效观察. 上海针灸杂志 28:375-376.

[107]TAO Hong-xing, GU Feng. (2009)Observations on the Efficacy of Acupuncture plus Chinese Herbal Medicine for Draining Dampness and Resolving Stasis in Treating Chronic Pelvic Inflammation. SHANGHAI JOURNAL OF ACUPUNCTURE AND MOXIBUSTION 28:375-376.

[108]苏春燕, 解建国. (2004)针刺配合走罐治疗五更泻的疗效观察. 上海针灸杂志 23:8-9.

[108]Su Chun-yan, Jie Jian-guo. (2004)Observations on the Efficacy of Acupuncture Plus Movable Cupping for Treatment of Predawn Diarrhea. SHANGHAI JOURNAL OF ACUPUNCTURE AND MOXIBUSTION 23:8-9.

[109]胥林波, 段红梅, 刘云兴. (2008)针刺牵引配合药枕治疗椎动脉型颈椎病疗效观察. 上海针灸杂志 27:20-21.

[109]XU Lin-bo, DUAN Hong-mei, LIU Yun-xing. (2008)Observations on the Efficacy of Acupuncture and Traction plus Medicinal Pillow in Treating Vertebral Artery Type Cervical Spondylosis. SHANGHAI JOURNAL OF ACUPUNCTURE AND MOXIBUSTION 27:20-21.

[110]李悦. (2010)针刺四关穴治疗偏头痛32例. 上海针灸杂志 29:733.

[110]Li Rui. (2010）acupuncture four closed cavity treatment of migraine：32cases. SHANGHAI JOURNAL OF ACUPUNCTURE AND MOXIBUSTION 29:733.

[111]房晓宇, 唐勇, 王中铎, [袁玉民](http://202.201.7.11/kcms/detail/search.aspx?dbcode=CJFQ&sfield=au&skey=Ԭ%D3%F1%C3%F1&code=22482123;06482721;20918262;20309893;). (2009)针刺太溪穴治疗脑卒中后吞咽障碍疗效观察. 上海针灸杂志 28:75-76.

[111] FANG Xiao-yu, ANG Yong, WANG Zhong-duo, YUAN Yu-minObservations on the Efficacy of Acupuncture at Point Taixi in Treating Poststroke Dysphagia. SHANGHAI JOURNAL OF ACUPUNCTURE AND MOXIBUSTION 28:75-76.

[112]马广昊, 郭长青, 张佛明. (2006)针刺条口穴治疗肩关节周围炎临床疗效研究与评价. 上海针灸杂志 25:23-24.

[112]GUO Chang-qing, ZHANG Fo-ming, MA Guang-hao. (2006)Study and evaluation on clinical therapeutic effect of acupuncture at Tiaokou (ST 38) for treatment of periarthritis of shoulder. SHANGHAI JOURNAL OF ACUPUNCTURE AND MOXIBUSTION 25:23-24.

[113]王敏, 王敏华, 华启海. (2005)“针刺头三神”穴为主治疗血管性痴呆疗效观察. 上海针灸杂志 24:12-14.

[113]Wang Mei, Wang Min-hua, Hua Qi-hai. (2005)"First three god" acupuncture clinical observation on treatment of vascular dementia. SHANGHAI JOURNAL OF ACUPUNCTURE AND MOXIBUSTION 24:12-14.

[114]阎固林.(2010)针刺推拿与功能锻炼治疗腰椎间盘突出症疗效观察. 上海针灸杂志 29:386-387.

[114]YAN Gu-lin. (2010)Observations on the Efficacy of Combined Acupuncture, Massotherapy and Functional Training in Treating Lumbar Intervertebral Disc Herniation. SHANGHAI JOURNAL OF ACUPUNCTURE AND MOXIBUSTION 29:386-387.

[115]王军英, 张惠佳, 王益梅, [覃蓉](http://202.201.7.11/kcms/detail/search.aspx?dbcode=CJFQ&sfield=au&skey=%F1%FB%C8%D8&code=07460587;07446390;07460591;07446352;07447218;07460580;07460575;),[汤孟平](http://202.201.7.11/kcms/detail/search.aspx?dbcode=CJFQ&sfield=au&skey=%CC%C0%C3%CFƽ&code=07460587;07446390;07460591;07446352;07447218;07460580;07460575;),等. (2002)针刺为主治疗小儿脑瘫200例临床研究. 上海针灸杂志 21:13-15.

[115]Wang Jun-ying, Zhang Hu-gui,Wang Yi-mei, Tan Rong,T ang Meng-ping,et al.(2002)Clinical research on the treatment of 200 cases of infantile cerebral palsy with acupuncture as main therapy. SHANGHAI JOURNAL OF ACUPUNCTURE AND MOXIBUSTION 21:13-15.

[116]曾学清, 滕东时, 林岫,等.(2010)针刺为主治疗中风后吞咽障碍疗效观察. 上海针灸杂志 29:431-432.

[116]ZENG Xue-qing, TENG Dong-shi, LIN Xiu. (2010)Observations on the Efficacy of Acupuncture as Main Treatment for Post-stroke Dysphagia. SHANGHAI JOURNAL OF ACUPUNCTURE AND MOXIBUSTION 29:431-432.

[117]姚配勇. (2009)针刺委中穴对脑卒中急性期下肢肌力恢复即刻疗效观察. 上海针灸杂志 28:138-139.

[117]YAO Pei-yong. (2009)Observations on the Immediate Rehabilitating Effect of Acupuncture at Point Weizhong(BL40) on Lower Limb Myodynamia in the Acute Stage of Cerebral Stroke. SHANGHAI JOURNAL OF ACUPUNCTURE AND MOXIBUSTION 28:138-139.

[118]柏亚萍, 傅杰英. (2007)针刺胃经穴治疗胃肠实热型肥胖症的临床研究. 上海针灸杂志 26:11-14.

[118]BAI Ya-ping, FU Jie-ying. (2007)Clinical Observations on Treatment of Gastrointestinal Excess Heat-type Obesity by Acupuncture of Stomach-meridian Points. SHANGHAI JOURNAL OF ACUPUNCTURE AND MOXIBUSTION 26:11-14.

[119]朱国祥, 邹超. (2003)针刺益肾补气法治疗足跟痛45例临床观察. 上海针灸杂志 22:23--24.

[119]Zhu Guo-xiang, Zhou Chao. (2003)Clinical Observations on the Treatment of 45 Heel Pain Patients by Kidney Qi-reinforcing Acupuncture Technique. SHANGHAI JOURNAL OF ACUPUNCTURE AND MOXIBUSTION 22:23--24

[120]殷之放, 翟道荡. (2001)针刺与艾灸治疗血小板减少性紫癜疗效比较. 上海针灸杂志 120:14-15.

[120]Yin Zhi-fang, Zhai Dao-dang. Comparison of Curative Effects Between Acupuncture and Moxibustion in Treating Thrombocytopenic Purpura. SHANGHAI JOURNAL OF ACUPUNCTURE AND MOXIBUSTION 120:14-15.

[121]殷之放, 汪司右. (2000)针刺与穴位敷贴治疗高血压病的临床比较. 上海针灸杂志 19:9-10.

[121]Yin Zhi-fang, Wang Si-you. (2000)Acupuncture and acupoint sticking therapy of hypertension clinical comparison. SHANGHAI JOURNAL OF ACUPUNCTURE AND MOXIBUSTION 19:9-10.

[122]寇晓茹, 孙立明, 郭娜,李平.(2010)针刺治疗胆结石术后胃肠功能紊乱疗效观察. 上海针灸杂志 29:103-104.

[122]KOU Xiao-ru, SUN Li-ming, GUO Na, LI Ping.(2010)Observations on the Therapeutic Effect of Acupuncture on Post-cholelithotomy Gastrointestinal Dysfunction.SHANGHAI JOURNAL OF ACUPUNCTURE AND MOXIBUSTION 29:103-104.

[123]陈捷, 孙立明, 马尚伟,李平.(2010)针刺治疗胆囊结石术后胃肠功能紊乱临床观察. 上海针灸杂志 29:298-299.

[123]CHEN Jie, SUN Li-ming, MA Shang-wei, LI PingObservations on the Therapeutic Effect of Acupuncture on Post-cholelithotomy Gastrointestinal Dysfunction. SHANGHAI JOURNAL OF ACUPUNCTURE AND MOXIBUSTION 29:298-299.

[124]王晓军, 朱卫红 , 陆乾人.(2010)针刺治疗第三腰椎横突综合征疗效观察. 上海针灸杂志 29(1):38-39.

[124]WANG Xiao-jun, ZHU Wei-hong, LU Qian-ren. (2010)Observations on the Efficacy of a Triple Treatment for Transverse Process Syndrome of Third Lumbar Vertebra. SHANGHAI JOURNAL OF ACUPUNCTURE AND MOXIBUSTION 29(1):38-39.

  [125]杜革术. (2007)针刺治疗儿童抽动秽语综合征疗效观察. 上海针灸杂志 3:5-6.

[125]DU Ge-shu(2007).Observations on the Curative Effect of Acupuncture on Child Tourette's Syndrome. Shanghai Journal of Acupuncture and Moxibustion 3:5-6

[126]程枫, 徐永宏 , 卢乐苗. (2010)针刺治疗腹部手术术后呃逆疗效观察. 上海针灸杂志29:698-699.

[126]CHENG Feng, XU Yong-hong, LU Le-miao. (2010)Observations on the Therapeutic Effect of Acupuncture on Hiccups After Abdominal Surgery. Shanghai Journal of Acupuncture and Moxibustion. 29:698-699.

[127]樊留博, 马利中, 卢敏芳.(2010)针刺治疗骨盆骨折术后神经源性膀胱尿动力学分析. 上海针灸杂志 29:111-112.

[127]FAN Liu-bo, MA Li-zhong, LU Min-fang(2010). Urodynamic Analysis in Acupuncture Treatment for Neurogenic Bladder After Pelvic Fracture Surgery. Shanghai Journal of Acupuncture and Moxibustion. 29:111-112.

[128]倪爱民. (2006)针刺治疗过敏性鼻炎与药物治疗对比观察. 上海针灸杂志 25:16-17.

[128]NI Ai-min. (2006)Comparison of acupuncture and medicine for treating allergic rhinitis.Shanghai Journal of Acupuncture and Moxibustion. 25:16-17.

[129]宗蕾, 侯文光, 王晓晔,安伯维. (2009)针刺治疗海洛因依赖者稽延期睡眠障碍疗效观察. 上海针灸杂志 28:191-194.

[129]ZONG Lei, HOU Wen-guang, WANG Xiao-ye, AN Bo-wei. (2009). Observations on the Therapeutic

Effect of Acupuncture on Protracted Dyssomnia in Heroin Addicts.Shanghai Journal of

Acupuncture and Moxibustion.28:191-194.

[130]杨洪伟, 黄雪颜. (2010)针刺治疗黄体功能不全性不孕疗效观察. 上海针灸杂志 29:626-628.

[130]YANG Hong-wei, HUANG Xue-yan. (2010)Observations on the Efficacy of Acupuncture

Treatment on Luteal Phase Defect Infertility.Shanghai Journal ofAcupuncture and

Moxibustion. 29:626-628.

[131]陶琪彬，卢翠飞.(2005)针刺治疗急性寰枢关节半脱位疗效观察. 上海针灸杂志 24:14-15.

[131]TAO Qi-bin, LU Cui-fei. (2005)Observations on the Curative Effect of Acupuncture on patients

with Acute Atlantoaxial Subluxation.Shanghai Journal of Acupuncture and Moxibustion.24:14-15.

[132]郄玉芳, 郄玉兰. (1998)针刺治疗急性尿道综合征23例. 上海针灸杂志 17:13.

[132]Xi Yu-fang, Xi Yu-lan. (1998)Acupuncture Treatment of 23 Cases of Acute Urethral

Syndrome.Shanghai Journal of Acupuncture and Moxibustion.17:13.

[133]方向军. (2010)针刺治疗老年带状疱疹后遗神经痛疗效观察. 上海针灸杂志 29:513-514.

[133]FANG Xiang-jun. (2010)Observations on the Therapeutic Effect of Acupuncture on Senile Postherpetic Neuralgia.Shanghai Journal of Acupuncture and Moxibustion.29:513-514.

[134]陈兴奎. (2010)针刺治疗脑卒中后日间过度倦睡症疗效观察. 上海针灸杂志 29:565-566.

[134]CHEN Xing-kui. (2010)Observations on the Therapeutic Effect of Acupuncture on Post-stroke

Excessive Daytime Sleepiness. Shanghai Journal of Acupuncture and Moxibustion. 29:565-566.

[135]范翠芳, 江泓颖, 武连仲. (2007)针刺治疗脑卒中后吞咽困难临床观察. 上海针灸杂志 26:6-7.

[135]FAN Cui-fang, JIANG Hong-ying , WU Lian-zhong.（2007 Clinical Observations on Acupuncture Treatment of Postapoplectic Dysphagia. Shanghai Journal of Acupuncture and Moxibustion. 26:6-7.

[136]孙忠人, 吴燕璟 , 王薇. (2002)针刺治疗偏头痛的疗效及生化学机制研究. 上海针灸杂志 21:16-17.

[136]Sun Zhong-ren, Wu Yan-jing, Wang Wei. (2002)Research into the Clinical Curative Effect of

Acupuncture on Migraine and Its Biochemical Mechanism. Shanghai Journal of Acupuncture and

Moxibustion. 21:16-17.

[137]李桂敏, 严伟 , 殷建权.(2010)针刺治疗偏头痛急性发作疗效观察. 上海针灸杂志 29:439-441.

[137]LI Gui-min, YAN Wei, YIN Jian-quan. (2010)Observations on the Therapeutic Effect of

Acupuncture on the Acute Migraine Attack. Shanghai Journal of Acupuncture and Moxibustion

29:439-441.

[138]陈云龙, 王承荣 , 林研研. (2009)针刺治疗前列腺痛疗效观察. 上海针灸杂志 28:324-325.

[138]CHEN Yun-long,WANG Cheng-rong, LIN Yan-yan. (2009)Observations on the Therapeutic Effect

of Acupuncture on Prostatodynia. Shanghai Journal of Acupuncture and Moxibustion.28:324-325.

[139]郑萍. (2009)针刺治疗失眠症疗效观察. 上海针灸杂志 28:632-633.

[139]Zheng Ping. (2009)Observations on the Therapeutic Effect of Acupuncture on Insomnia. Shanghai Journal of Acupuncture and Moxibustion. 28:632-633.

[140]刘瑞云, 赵鹏台. (2009)针刺治疗糖尿病胃轻瘫疗效观察. 上海针灸杂志 28:390-391.

[140]Liu Rui-yun, Zhao Peng-tai. (2009)Observations on the Therapeutic Effect of Acupuncture on Mild Diabetic Gastroplegia. Shanghai Journal of Acupuncture and Moxibustion. 28:390-391.

[141]王相奇. (2009)针刺治疗痛风性关节炎急性发作疗效观察. 上海针灸杂志 28:260-261.

[141]Wang Xiang-qi. (2009)Observations on the Therapeutic Effect of Acupuncture on the Acute Attack

of Gouty Arthritis.Shanghai Journal of Acupuncture and Moxibustion.28:260-261.

[142]公维志, 王玉琦. (2010)针刺治疗纤维肌痛综合征疗效观察. 上海针灸杂志 29:725-727.

[142]Gong Wei-zhi, Wang Yu-qi. (2010)Observations on the Therapeutic Effect of Acupuncture on

Fibromyalgia Syndrome. Shanghai Journal of Acupuncture and Moxibustion. 29:725-727.

[143]朱慧勤. (2002)针刺治疗心悸的临床疗效观察. 上海针灸杂志 21:9-10.

[143]Zhu Hui-qin. (2002)Clinical Observations on the Curative Effect of Acupuncture on

Palpitation.Shanghai Journal of Acupuncture and Moxibustion. 21:9-10.

[144]于文, 沈帆霞, 翁中芳,胡大萌,吴建华,等. (2000)针刺治疗血管性头痛急性发作的临床研究. 上海针灸杂志 19:15-16.

[144]Yu Wen, ShenFan-xia, Weng Zhong-fang, Hu Da-meng, Wu Jian-hua.. (2000)Clinical research in

acupuncture treatment of acute attack of vascular headache. Shanghai Journal of Acupuncture and

Moxibustion. 19:15-16.

[145]罗仁瀚, 徐凯, 黄云声. (2009)针刺治疗抑郁症临床观察. 上海针灸杂志 28:69-71.

[145]Luo Ren-han, Xu Kai, Huang Yun-sheng. (2009)Clinical Observations on Acupuncture Treatment

for Depression.Shanghai Journal of Acupuncture and Moxibustion.19:15-16.

[146]陈兵, 涂小华. (2008)针刺治疗原发性痛经疗效观察. 上海针灸杂志 27:15-16.

[146]Chen Bing, Tu Xiao-hua. (2008)Clinical Observations on the Efficacy of Acupuncture in Treating

primary Dysmenorrhea.Shanghai Journal of Acupuncture and Moxibustion.27:15-16.

[147]王延红, 应盛国. (2006)针刺治疗中风后假性球麻痹疗效观察. 上海针灸杂志 25:15-16.

[147]Wang Yan-hong, Ying Sheng-guo. (2006)Observations on the Curative Effect of Acupuncture on

Postapoplectic Pseudobulbar Paralysis. Shanghai Journal of Acupuncture and Moxibustion.

25:15-16.

[148]嵇强, 徐前方, 周芸, 顾红,张艳. (2004)针刺治疗中风后假性球麻痹吞咽困难35例. 上海针灸杂志 23:11-12.

[148]Ji Qiang,Xun, Qian-fang, Zhou Yun, Gu Hong,Zhang Yan. (2004)Acupuncture Treatment of 35

Patients with Dysphagia due to Apoplectic Psudobulbar Paralysis.Shanghai Journal of

Acupuncture and Moxibustion.23:11-12.

[149]张桂波, 杨玲, 张倩. (2010)针刺治疗中风后抑郁症疗效观察. 上海针灸杂志 29,636-637.

[149]Zhang Gui-bo, Yang Ling, Zhang Qian. (2010)Observations on the Therapeutic Effect of

Acupuncture on Post-stroke Depression.Shanghai Journal of  Acupuncture and Moxibustion.

23:11-12.

[150]曾学清, 李艳, 滕东时,杨涛 . (2008)针刺治疗中风后足下垂疗效观察. 上海针灸杂志 27:23-24.

[150]Zeng Xue-qing, Li Yan,Teng , Dong-shi, Yang Ta. (2008)Observations on the Therapeutic Effect of

Acupuncture on Postapoplectic Foot Drop.Shanghai Journal of  Acupuncture and Moxibustion.

27:23-24.

[151]彭拥军, 李忠仁 , 杨永清. (2006)针刺治疗中风假性球麻痹30例临床观察. 上海针灸杂志 25:10-12.

[151]Peng Yong-jun, Li Zong-ren, Yang Yong-qing. (2006)Clinical observations on acupuncture

treatment of 30 patients with apoplectic pseudbulbar paralysis.Shanghai Journal of  Acupuncture

and Moxibustion.25:10-12.

[152]李国辉, 陈惠. (2009)针刺治疗中风假性球麻痹临床观察. 上海针灸杂志 28:213-214.

[152]Li Guo-hui, Chen Hui. (2009)Clinical Observations on Acupuncture Treatment for Apoplectic

Pseudobulbar Palsy. Shanghai Journal of  Acupuncture and Moxibustion. 28:213-214.

[153]谢潇侠. (1996)针刺综合治疗面瘫83例. 上海针灸杂志 15:125-126.

[153]Xie Xiao-xia. (1996)Comprehensive therapy Effect of Acupuncture on 83 patients of

facioplegia.Shanghai Journal of  Acupuncture and Moxibustion. 15:125-126.

[154]朱国庆, 韦兆玲, 苏慧,梁英. (2009)针刀微创治疗膝骨关节炎疗效观察. 上海针灸杂志 28:98-99.

[154]Zhu Guo-qing, Wei Zhao-ling, Su Hui, Ling Ying. (2009)Clinical Study of Micro-invasive Needle Knife

Treatment for Genual Osteoarthritis. Shanghai Journal of  Acupuncture and Moxibustion.

28:98-99.

[155]杨城, 王琴. (2009)针刀为主治疗腰椎间盘突出症疗效观察. 上海针灸杂志

[155]Yang Cheng, Wang Qin. (2009)Observations on the Efficacy of Needle Knife as a Main Treatment for Lumbar Intervertebral Disc Protrusion.Shanghai Journal of  Acupuncture and Moxibustion. 27:17-19.

[156]叶忠. (2004)针刀为主治疗腰椎间盘突出症临床研究. 上海针灸杂志 23:11-12.

[156]Ye Zhong. (2004)Clinical Studies on the Treatment of Lumbar Intervertebral Disc Protrusion with a Needle Knife as the Main Method. Shanghai Journal of  Acupuncture and Moxibustion. 23:11-12.

[157]杨城, 王琴. (2010)针刀治疗紧张型头痛疗效观察. 上海针灸杂志 29:161-162.

[157]Yang Cheng, Wang Qin. (2010)Observations on the Effect of Knife Needle Therapy on Tension Headache.Shanghai Journal of  Acupuncture and Moxibustion. 29:161-162.

[158]万国强. (2008)针灸辨证分型治疗单纯性肥胖症疗效观察. 上海针灸杂志 27:19-20.

[158]Wan Guo-Qing. (2008)Observations on the Efficacy of Syndrome Differentiation and Classification-based Acupuncture and Moxibustion in Treating Simple Obesity. Shanghai Journal of  Acupuncture and Moxibustion.27:19-20.

[159]王占奎, 王伟志, 傅立新, 赵建国,宋平. (2005)针灸对颈动脉粥样硬化患者颈动脉形态学和动力学的影响. 上海针灸杂志 24:8-11.

[159]WANG Zhan-kui, WANG Wei-zhi, FU Li-xin, ZHAO Jian-guo, SONG Ping. (2005)Influence of Acupuncture and Moxibustion and Carotid Morphology and Dynamics in Patients with Carotid Atherosclerosis.Shanghai Journal of  Acupuncture and Moxibustion. 24:8-11.

[160]王伟志, 王占奎, 赵建国,傅立新. (2005)针灸对缺血性脑血管病颈动脉粥样硬化患者血脂、血流变、LPO和SOD、ET和CGRP的影响. 上海针灸杂志 24:19-23.

[160]WANG Wei-zhi, WANG Zhan-kui, ZHAO Jiao-guo, FU Li-xin. (2005)Influence of Acupuncture and Moxibustion on Lipid, Blood Flow Deformation, LPO, SOD, ET and CGRP in Patients with Carotid Atherosclerosis due to Ischemic Cerebrovascular Diseases. Shanghai Journal of  Acupuncture and Moxibustion. 24:19-23.

[161]粟胜勇, 邓柏颖, 李扬帆,周恩华. (2004)针灸对中风后偏瘫肩痛及其全血黏度的近期影响. 上海针灸杂志 23:10-12.

[161]Li Sheng-yong, Deng Pai-ying, Li Yang-fan, Zhou En-hua. (2004)Observations on the Short-term Effect of

Acupuncture and Moxibustion on Post-apoplectic Sequela Omodynia and Its Whole Blood

Viscosity.23:10-12.

[162]范钰, 杨兆民, 万铭,吴学飞,颜吉丽. (2001)针灸防治恶性肿瘤化疗毒副反应的临床研究. 上海针灸杂志 20:12-14.

[162]Fan Yun, Yang Zhao-min, Wan Ming, Wu Xue-fei, Yan Ji-li. (2001)Clinical Study on Preventing the Virulent and Side-effect of Malignant Tumor Du e to Chemotherapy by Acu-mox. Shanghai Journal of  Acupuncture and Moxibustion. 20:12-14.

[163]吴玲, 杨学珍, 牛俊明. (2005)针灸防治支气管哮喘的临床研究. 上海针灸杂志 24:8-9.

[163]Wu Lin, Yang Xue-zhen, Niu Jun-ming. (2005)Clinical Observations on the Prevention and Treatment of Bronchial Asthma by Point Application and Point Injection plus Ginger-separated Moxibustion.Shanghai Journal of  Acupuncture and Moxibustion.24:8-9.

[164]徐玉华, 李清, 郝永清. (2005)针灸加康复综合治疗失语症的疗效观察. 上海针灸杂志 24:30-31.

[164]Xu Yu-hua, Li Qing, Hao Yong-qing. (2005)Observation on the Efficacy of Acupuncture plus

Rehabilitation Composite Treatment for Apoplectic Aphasia.Shanghai Journal of  Acupuncture

and Moxibustion.24:30-31.

[165]向贤德, 冯斌. (2004)针灸加穴位注射治疗椎动脉型颈椎病90例临床观察. 上海针灸杂志 23:13-14.

[165]Xiang Xian-de, Feng Bin. (2004)Clinical Observations on the Treatment of 90 Cases of

Vertebroarterial Cervical Spondylopathy by Acupuncture and Moxibustion plus Point

Injection. Shanghai Journal of  Acupuncture and Moxibustion. 23:13-14.

[166]牟淑敏, 陈彦华. (2008)针灸加中药治疗糖尿病神经源性膀胱疗效观察. 上海针灸杂志 27:25-26.

[166]Mou Shu-min, Chen Yan-hua. (2008)Observations on the Efficacy of Acupuncture and Moxibustionplus Herbal Medicine in Treating Diabetic Neurogenic Bladder. Shanghai Journal of  Acupunctureand Moxibustion. 27:25-26.

[167]程继君, 费建中, 张晨光. (2005)针灸结合推拿治疗腰椎间盘突出症疗效观察. 上海针灸杂志 24:17-18.

[167]Cheng Ji-jun, Fei Jian-zhong, Zhang Chen-guang. (2005)Observations on the Efficacy of

Acupuncture plus Massotherapy for Treating Lumbar Intervertebral Disc Protrusion. Shanghai

Journal of  Acupuncture and Moxibustion.27:25-26.

[168]邹一超. (2004)针灸结合行为疗法治疗重复使力损伤疗效观察. 上海针灸杂志 23:21-22.

[168]Zou Yi-chao. (2004)Observations on the Efficacy of Acupuncture plus Behavior Therapy for

Treatment of Repeated Strain Injury. Shanghai Journal of  Acupuncture and Moxibustion.

23:21-22.

[169]卢泽强. (2010)针灸配合拔罐治疗带状疱疹疗效观察. 上海针灸杂志 29:601-602.

[169]Lu Ze-qiang. (2010)Observations on the Efficacy of Acupuncture and Moxibustion plus Cuppingin Treating Herpes Zoster. Shanghai Journal of  Acupuncture and Moxibustion. 29:601-602.

[170]裴瑜, 王伟明. (2009)针灸配合耳穴磁珠贴压治疗失眠症疗效观察. 上海针灸杂志 28:629-631.

[170]Pei Yu, Wang Wei-ming. (2009)Observations on the Efficacy of Acupuncture plus Auricular Point Magnetic Bead Plaster Therapy in Treating Insomnia. Shanghai Journal of  Acupuncture andMoxibustion 28:629-631.

[171]张雅萍, 尚亚婷, 来进花. (2007)针灸配合颈部制动治疗颈椎病的远期疗效观察. 上海针灸杂志 26:10-11.

[171]Zhang Ya-ping, Shang Ya-ting, Lai Jin-hua. (2007)Observations on the Long-term Efficacy ofAcupuncture and Moxibustion Lus Neck Immobilization for Treating Cervical

Spondylopathy. Shanghai Journal of  Acupuncture and Moxibustion 26:10-11.

[172]卢泽强. (2006)针灸配合推拿治疗多发性神经炎48例临床观察. 上海针灸杂志 25:23-24.

[172]Lu Ze-qing. (2006)Clinical Observations on Treatment of 48 Multiple Neuritis Patients by

Acupuncture and Moxibustion plus Massotherapy. Shanghai Journal of  Acupuncture and

Moxibustion 25:23-24.

[173]杨春光, 廖明霞. (2010)针灸配合推拿治疗心脾两虚型失眠症疗效观察. 上海针灸杂志 29:654.

[173]Yang Chun-guang, Liao Ming-xia. (2010)Clinical Observations on Treatment of two deficiency typeinsomnia casesby Acupuncture and Moxibustion plus Massotherapy. Shanghai

Journal of  Acupuncture and Moxibustion 29:654.

[174]刘登红. (2009)针灸配合心理疗法治疗围绝经期综合征疗效观察. 上海针灸杂志 28:373-374.

[174]Liu Deng-hong. (2009)Observations on the Efficacy of Acupuncture and Moxibustion plus

Psychotherapy in Treating Female Climacteric Syndrome.Shanghai Journal of  Acupuncture and

Moxibustion 28:373-374.

[175]陈莉秋, 苗荃, 范鹏,张娜.(2010)针灸配合心理疗法治疗腰椎间盘突出症疗效观察. 上海针灸杂志 29:383-385.

[175]Chen Li-qiu, Miao Quan, Fan Peng,Zhang Na. (2010)Observations on the Efficacy of Acupunctureand Moxibustion plus Psychotherapy in Treating Lumbar Intervertebral Disc

Herniation.Shanghai Journal of  Acupuncture and Moxibustion 29:383-385.

[176]赵伟忠, 叶国传. (2003)针灸推拿治疗慢性软组织损伤180例疗效观察. 上海针灸杂志 22:16-17.

[176]Zhao Wei-zhong,Ye Guo-chuan. (2003).Observations on the Curative Effect of Acupuncture and

Tuina Therapy on 180 Patients with Chronic Soft Tissue Injury.Shanghai Journal of  Acupuncture

And Moxibustion.22:16-17.

[177]刘和春. (2009)针灸为主治疗类风湿性关节炎临床观察. 上海针灸杂志 28:224-225.

[177]Liu He-chun. (2009)Exploration of the Way to Treat Rheumatoid Arthritis by Acupuncture and

Moxibustion as Main Therapy.Shanghai Journal of  AcupunctureAnd Moxibustion.28:224-225.

[178]宋文革, 吴弢, 刘萌,高翔. (2001)针灸为主治疗膝骨关节炎临床分析. 上海针灸杂志 20:22-23.

[178]Song Wen-ge, Wu ?, Liu Meng, Gao Xiang. (2001)Analysis of the Therapeutic Effect of

Acupuncture plus Spectrograp Irradiation on Treating Gonitis.Shanghai Journal of

 Acupuncture And Moxibustion.20:22-23.

[179]刘朝生, 周晖, 唐晓萍,刘芳. (2006)针灸与高压氧促醒颅脑损伤昏迷患者45例疗效观察. 上海针灸杂志 25:21-22.

[179]Liu Chao-sheng, Zhou Hui,Tang Xiao-ping, Liu-fang. (2006)Observations on the Efficacy of

Acupuncture plus Hyperbaric Oxygen for Reviving Coma Patients with Craniocerebral

Injury. Shanghai Journal of  Acupuncture And Moxibustion 25:21-22.

[180]邢丹, 倪姗姗, 高旭超,朱在波,杜学杰,等. (2010)针灸择期治疗周围性面瘫疗效观察. 上海针灸杂志 29:289-291.

[180]Xing Dan, Ni Shan-shan, Gao Xun-chao, Zhun Zai-bo, Du Xue-jie. (2010)Observations on the Therapeutic Effect of Time Acupuncture and Moxibustion on Peripheral Facial Paralysis. Shanghai Journal of  Acupuncture And Moxibustion. 29:289-291.

[181]刘胜, 王玲, 牛京权,王国金. (1996)针灸止癌痛. 上海针灸杂志 15:42-43.

[181]Liu Sheng, Wang Ling, Niu Jing-quan,Wang Guo-jin. (1996)Acupuncture to relieve pain of

Cancer. Shanghai Journal of  Acupuncture And Moxibustion.15:42-43.

[182]安广青, 李娜, 翟国华, 刘慧荣,孙记冯,等.(2010)针灸治疗肠易激综合征疗效评价. 上海针灸杂志 29:354-356.

[182]An Guang-qing, Li Na, Qu Guo-hua, Liu Hui-rong, Sun Ji-feng. (2010)Evaluation of the Therapeutic Effect of Acupuncture and Moxibustion on Irritable Bowel Syndrome.Shanghai Journal of  Acupuncture And Moxibustion. Shanghai Journal of  Acupuncture And Moxibustion 29:354-356.

[183]张道武, 郑秀芝, 王秋景. (1999)针灸治疗更年期综合征临床研究. 上海针灸杂志 18:20-21.

[183]Zhang Dao-wu, Zheng Xiu-zhi,Wang Qiu-jing. (1999)Clinical Research in Acu-moxibustior

Treatment of climacteric syndrome.Shanghai Journal of  Acupuncture And Moxibustion. Shanghai Journal of  Acupuncture And Moxibustion 18:20-21.

[184]尹淑英, 刘心莲, 孙丽琴,肖君,刘成贵,等. (1992)针灸治疗类风湿性关节炎对NK细胞活性的调整. 上海针灸杂志:14-15.

[184]Yi Shu-ying, Liu Xin-lian, Sun Li-qin, Xiao Jun, Liu Cheng-gui. (1992)The influence of Acupunctureto tratement rheumatoid arthritis to the activity of NK cell. Shangha i Journal of  AcupunctureAnd Moxibustion 14-15.

[185]李坚将, 刘辉. (2001)针灸治疗面瘫及对口唇、甲襞微循环的影响. 上海针灸杂志,

[185]Li Jian-jang, Liu Hui. (2001)INFLUENCE OF DIFFERENTIAL ACUPUNCTURE ON THEACUTE PERIPHERAL FACIAL PARALYSIS A ND MICRO-CIRCULATION IN LIP AND

NAILS. Shanghai Journal of  Acupuncture And Moxibustion.

[186]张必萌, 黄文燕, 徐斯伟, 陈蓓琳,俞芳,等. (2009)针灸治疗糖尿病性胃轻瘫综合征临床观察. 上海针灸杂志 28:696-697.

[186]Zhang Bi-meng, Huang Wen-yan, Xun Si-wei, Chen Bei-lin,Yu Fang. (2009)Clinical Observationson Acupuncture Treatment for Diabetic Gastroparesis Syndrome. Shanghai Journal of Acupuncture And Moxibustion. 28:696-697.

[187]徐斯伟. (2008)针灸治疗膝骨关节炎疗效观察. 上海针灸杂志 27:11-12.

[187]Xu Si-wei. (2008)Observations on the Therapeutic Effect of Acupuncture on Genual

Osteoarthritis. Shanghai Journal of  Acupuncture And Moxibustion. 27:11-12.

[188]姚志芳, 林源. (2007)针灸治疗腰椎间盘突出症疗效观察. 上海针灸杂志 26:25-26.

Yao Zhi-fang, Lin Yuan. (2007)Observations on the Curative Effect of Acupuncture and

Moxibustion on Lumbar Intervertebral Disc Protrusion.Shanghai Journal of  Acupuncture And

Moxibustion. 26:25-26.

[189]肖珍科, 王保, 周仆, 陈陈燕,尧新华,等. (2009)针灸治疗仪结合电火针在神经阻滞定位中的应用. 上海针灸杂志,322-323.

[189]XIAO Zhen-ke, WANG Bao, ZHOU Pu, CHEN Chen-yan, YAO Xin-hua. (2009)Application of Acupuncture Device plus Electrical Fire Needling to Nerve Block Location. Shanghai

Journal of  Acupuncture And Moxibustion. 322-323.

[190]庞勇, 赵利华, 农泽宁,叶仿武,梁劲松,等. (2008)针灸治疗原发性骨质疏松症临床观察. 上海针灸杂志 27:15-16.

[190]PENG Yong, ZHAO Li-hua, NONG Zhe-hua, YE Fang-wu, LIANG Jing-song. (2008)Clinical Study of Acupuncture-moxibustion Treatment for Primary Osteoporosis.Shanghai Journal of  Acupuncture And Moxibustion.27:15-16.

[191]谢登岗. (1996)针灸综合疗法治疗面瘫135例. 上海针灸杂志 15:118-119.

[191]Xie Deng-gang. (1996)Comprehensive therapy Effect of Acupuncture on 135 patients of

Acioplegia. Shanghai Journal of  Acupuncture and Moxibustion. 15:118-119.

[192]张道武, 张小瑞 , 梁晓菲. (2001)针推结合治疗颈椎病的临床观察. 上海针灸杂志 20:21-22.

[192]Zhang Dao-wu, Zhang Xiao-rui, Liao Xiao-fei. (2001)linical Observations on the Treatment of Cervical Spondylopa thy by Acu-moxibustion and Massage. Shanghai Journal of

Acupuncture And Moxibustion. 20:21-22.

[193]张道武, 梁晓菲, 王秋景. (2002)针推结合治疗腰椎间盘突出症临床研究. 上海针灸杂志 21:22-23.

[193]Zhang Dao-wu, Liang Xiao-fei, Wang Jing-qiu. (2002)Clinical Research on the Treatment of Lumbar Intervertebral Disc Protrusion by the Combination of Acupuncture and Massage. Shanghai Journal of Acupuncture And Moxibustion. 21:22-23.

[194]朱吕杰. (2006)针药并施治疗周围性面神经麻痹急性期疗效观察. 上海针灸杂志 25:17-18.

[194]Zhu Lv-jie. (2006)Observations on the efficacy of combined acupuncture and medication for treating the acute stage of peripheral facial paralysis. Shanghai Journal of

Acupuncture And Moxibustion. 25:17-18.

[195]余志辉, 胡建芳. (2008)针药并用调和阴阳对中风后肢体痉挛状态的影响. 上海针灸杂志 27:18-20.

[195]Yu Zhi-hui, Hu Jian-fang. (2008)Effect of Combined Use of Acupuncture and Medicine to Regulate Yin and Yang on Postapoplectic Limb Spasm. Shanghai Journal of  Acupuncture And

Moxibustion.27:18-20.

[196]姜劲峰, 刘兰英, 王俊, 王玲玲,张建斌. (2007)针药并用对抑郁症患者HAMD量表因子的影响. 上海针灸杂志 26:3-5.

[196]JIANG Jin-feng, LIU Lan-ring, WANG Jun, WANG Ling-ling, ZHANG Jian-bin. (2007)Influence of Combined Acupuncture and Medication on HAMD Factors in Depression Patients. Shanghai Journal of  Acupuncture And Moxibustion. 26:3-5.

[197]刘霞. (2010)针药并用对椎动脉型颈椎病血流动力学的影响. 上海针灸杂志 29:109-110.

[197]Liu Xia. (2010)Effect of Combined Acupuncture and Medicine on Hemodynamics in Cervical Spondylotic Vertebral Arteriopathy. Shanghai Journal of  Acupuncture And Moxibustion. 29:109-110.

[198]陈莉秋. (2007)针药并用为主治疗周围性面神经麻痹疗效观察. 上海针灸杂志 26:16-17.

[198]Chen Li-qiu. (2007)Observations on the Efficacy of Combined Acupuncture and Medicine as Main Treatment for Peripheral Facial Paralysis.Shanghai Journal of  Acupuncture And Moxibustion. 26:16-17.

[199]庞声航, 刘国君, 赵文圣. (2009)针药并用治疗2型糖尿病高血压及对血浆神经肽Y的影响. 上海针灸杂志 28:442-444.

[199]PANG Sheng-hang, LIU Guo-jun, ZHAO Wen-sheng. (2009)Effect of the Combined Use of Acupuncture and Medicine on Hypertension and Plasma Neuropeptide Y in Type 2 Diabetes. Shanghai Journal of  Acupuncture And Moxibustion. 28:442-444.

[200]陆倩, 王海东, 康冰. (2010)针药并用治疗格林-巴利综合征患者肌力恢复疗效观察. 上海针灸杂志 29:300-302.

[200]LU Qian, WANG Hai-dong, KANG Bing. (2010)Observations on the Restoring Effect of Combined Acupuncture and Medicine on Myodynamia in Patients with Guillain-Barre Syndrome. Shanghai Journal of  Acupuncture And Moxibustion. 29:300-302.

[201]夏勇, 舒适, 李艺, 刘世敏,何金森.(2010)针药并用治疗甲亢突眼症临床观察. 上海针灸杂志 29:498-500.

[201]Xia Yong, Shu Shi, Li Yi, Liu Shi-min, HE Jin-sen. (2010)Clinical Observations on the CombinedTreatment of Hyperthyroid Exophthalmos with Acupuncture and Drugs.Shanghai

Journal of  Acupuncture And Moxibustion.29:498-500.

[202]夏勇, 舒适, 李艺,刘世敏,何金森. (2009)针药并用治疗甲状腺功能亢进性突眼症临床效应观察. 上海针灸杂志 28:691-693.

[202]XIA Yong, SHU Shi, LI Yi, LIU Shi-min, HE Jin-sen. (2009)Observations on the Clinical Efficacy of Combined use of Acupuncture and Drugs in Treating Hyperthyroid Exophthalmos. Shanghai Journal of  Acupuncture And Moxibustion. 28:691-693.

[203]胡涛, 田明. (2005)针药并用治疗慢性胆囊炎52例. 上海针灸杂志 24:14-15.

[203]HU Tao, TIAN Ming. (2005)Treatment of 52 Chronic Cholecystitis Cases by Combined

Acupuncture and Herbs.Shanghai Journal of  Acupuncture And Moxibustion.24:14-15.

[204]张银开, 蒋应星. (2007)针药并用治疗脑卒中后抑郁症30例疗效观察. 上海针灸杂志 26:5-6.

[204]ZHANG Yin-kai, JIANG Ying-xing. (2007)Observations on the Efficacy of Combined Acupunctureand Medicine for Treating 30 Post-stroke Depression Patients. Shanghai Journal of  AcupunctureAnd Moxibustion. 26:5-6.

[205]孙远征, 李昆珊. (2010)针药并用治疗轻度认知功能障碍临床观察. 上海针灸杂志 29:759-761.

[205]SUN Yuan-zheng, LI Kun-shan. (2010)Clinical Observation on the Treatment of Mild CognitiveImpairment by Combined Acupuncture and Medication.Shanghai Journal of  AcupunctureAnd Moxibustion. 29:759-761.

[206]李景义, 魏占华. (1999)针药并用治疗90例. 上海针灸杂志 18:19-20.

[206]Li Jing-yi, Wei Zhan-hua. (1999)Treatment of neurasthenia syndrome by Combined Acupuncture of90 patients.Shanghai Journal of  Acupunctur And Moxibustion. 18:19-20.

[207]王湃, 张跃全, 楚国庆. (2001)针药并用治疗术后胃肠综合征临床观察. 上海针灸杂志 20:10-11.

[207]Wang Pai, Zhang Yue-quan, Chu Guo-qing. (2001)Clinical Observation of Gastrointestinal

Postoperative Syndrome Treated by Combi nation of Acupuncture and Chinese Herbal

Medicine. Shanghai Journal of  Acupuncture And Moxibustion. 20:10-11.

[208]孙远征, 罗义玲.(2010)针药并用治疗糖尿病视网膜病变疗效观察. 上海针灸杂志 29:446-448.

[208]Sun Yuan-zheng, Luo Yi-ling. (2010)Observations on the Efficacy of Combined Acupuncture andMedicine in Treating Diabetic Retinopathy. Shanghai Journal of  Acupuncture And

Moxibustion. 29:446-448.

[209]张红智, 张秋娟, 东红升.(2010)针药并用治疗糖尿病周围神经病变疗效观察. 上海针灸杂志 29:702-705.

[209]ZHANG Hong-zhi, ZHANG Qiu-juan, DONG Hong-sheng. (2010)Observations on the Efficacy of Combined Acupuncture and Medicine in Treating Diabetic Peripheral Neuropathy. Shanghai Journal of  Acupuncture And Moxibustion.29:702-705.

[210]徐慧卿.(2010)针药并用治疗特发性肺纤维化疗效观察. 上海针灸杂志 29:641-642.

[210]Xun Hui-qing. (2010)Observations on the Efficacy of Combined Use of Acupuncture and

Medicine in Treating Idiopathic Pulmonary Fibrosis.Shanghai

Journal of  Acupuncture And Moxibustion.29:641-642.

[211]憨兰. (2008)针药并用治疗膝关节创伤性滑膜炎疗效观察. 上海针灸杂志 27:29-30.

[211]Han Lan. (2008)Observations on the Efficacy of Combined Acupuncture and Medicine in Treating Traumatic Gonarthromeningitis. Shanghai Journal of  Acupuncture And Moxibustion 27:29-30.

[212]高扬. (2009)针药并用治疗阳痿疗效观察. 上海针灸杂志 28:83-84.

[212]Gao Yang. (2009)Observations on the Efficacy of Combined Use of Acupuncture and Medicine Treating Impotence. Shanghai Journal of  Acupuncture And Moxibustion .28:83-84.

[213]汪崇淼, 吴耀持, 张峻峰, 李石胜. (2010)针药并用治疗腰突症术后复发临床疗效观察. 上海针灸杂志 29:785-786.

[213]WANG Chong-mia, WU Yao-chi, ZHANG Jun-feng, LI Shi-sheng. (2010)Clinical Observation on the Treatment of Relapsed Lumbar Disc Herniation after Disc Surgery by Combined Acupuncture and Medication. Shanghai Journal of  Acupuncture And Moxibustion. 29:785-786.

[214]李振海. (2006)针药并用治疗腰椎间盘突出症的临床观察. 上海针灸杂志 25:24-25.

[214]Li Zhen-hai. (2006)Clinical Observations on the Treatment of Lumbar Intervertebral Disc Protrusion by Combined Acupuncture and Medicine. Shanghai Journal of  Acupuncture And Moxibustion. 25:24-25.

[215]卢岱静, 赵正焱. (2010)针药并用治疗腰椎间盘突出症疗效观察. 上海针灸杂志 29:176-177.

[215]LU Dai-jing, ZHAO Zheng-yan. (2010)Observations on the Efficacy of Combined Acupuncture and Medicine in Treating Lumbar Intervertebral Disc Herniation. Shanghai Journal of  Acupuncture And Moxibustion. 29:176-177.

[216]邹伟, 赵佳辉, 于学平. (2007)针药并用治疗抑郁症60例临床观察. 上海针灸杂志 26:5-7.

[216]ZHOU Wei, ZHAO Jia-hui, YU Xue-ping. (2007)Llinical observations on treatment of 120 depression patients by combined use of acupuncture and medicine. Shanghai Journal of  Acupuncture And Moxibustion .26:5-7.

[217]朱东晓. (2010)针药并用治疗支气管哮喘的疗效及对细胞因子的影响. 上海针灸杂志 29:91-93.

[217]Zhu Dong-xiao. (2010)Effect of Combined Acupuncture and Medicine on Bronchial Asthma and Cytokine. Shanghai Journal of  Acupuncture And Moxibustion .29:91-93.

[218]吴芳华, 朱启玉. (2009)针药并用治疗重型斑秃疗效观察. 上海针灸杂志 28:334-335.

[218]WU Fang-hua, ZHU Qi-yu. (2009)Observations on the Efficacy of Combined Use of Acupuncture and Medicine in Treating Severe Alopecia Areata. Shanghai Journal of  Acupuncture And Moxibustion. 28:334-335.

[219]王建林, 李宏伟. (2010)针药并用治疗椎-基底动脉供血不足的疗效观察. 上海针灸杂志 29:163-164.

[219]WANG Jian-lin, LI Hong-wei. (2010)Observations on the Efficacy of Combined Acupuncture and Medicine in Treating Vertebrobasilar Insufficiency. Shanghai Journal of  Acupuncture And Moxibustion. 29:163-164.

[220]付于, 夏天. (2005)针药并用治疗子宫内膜异位症的临床观察. 上海针灸杂志 24:3-7.

[220]FU Yu, XIA Tian.（2005）Clinical Observations on the Treatment of Endometriosis by Combined Acupuncture and Herbs. Shanghai Journal of  Acupuncture And Moxibustion. 24:3-7.

[221]史晓岚, 杨帅, 张国庆. (2010)针药不同疗法对围绝经期抑郁症的效应分析. 上海针灸杂志 29:756-758.

[221]SHI Xiao-lan, YANG Shuai, ZHANG Guo-qing. (2010)Effect Analysis on the Treatment of Perimenopausal Depression by Acupuncture and Medication Respectively. Shanghai Journal of Acupuncture And Moxibustion. 29:756-758.

[222]陆黎. (2005)针药复合麻醉对腹部术后患者使用镇痛泵的影响. 上海针灸杂志 24:6-7.

222]Lu Li. (2005)The Influence of Electroacupuncture-associated General Anesthesia on Use of Venous Analgetic Pump in Abdominal Postoperative Patients. Shanghai Journal of  Acupuncture And Moxibustion .24:6-7.

[223]张泳南, 刘智艳. (1999)针药结合治疗地方性甲状腺肿的临床观察. 上海针灸杂志 18:4-5.

[223Zhang Yong-nan, Liu Zhi-yan. (1999)Clinical Observations on the Treatment of endemic struma byCombination of Acupuncture and Medicine. Shanghai Journal of  Acupuncture And

Moxibustion. 18:4-5.

[224]贾红玲, 申康, 张永臣. (2005)针药结合治疗强直性脊柱炎临床观察. 上海针灸杂志 24:23-25.

[224]JIA Hong-ling, SHEN Kang, ZHANG Yong-cheng. (2005)Clinical Observations on the Treatment of 40 Ankylosing Spondylitis Patients by Combination of Acupuncture and Medicine. Shanghai Journal of  Acupuncture And Moxibustion. 24:23-25.

[225]郑蕙田, 李永方, 袁顺兴, 张晨光,陈国美,等. (2000)针药结合治疗糖尿病周围神经病变52例. 上海针灸杂志 19:8-9.

[225]Zheng Hui-tian, Li Yong-fang, Yuan Shun-xing, Zhang Chen-guang, Chen Guo-mei. (2000)Clinical observations on the treatment of diabetic peripheral neuropathy by combined acupuncture and medicament. Shanghai Journal of  Acupuncture And Moxibustion. 19:8-9.

[226]罗卫平, 缪英年, 钟福帮. (2004)针药结合治疗小儿高热惊厥的疗效观察. 上海针灸杂志 23:12-13.

[226]Luo Wei-ping, Mu Ying-nian, Zhong Fu-bang. (2004)Observations on the Curative Effect of Combined Acupuncture and Medication on Infantile Hyperpyretic Convulsion. Shanghai Journal of  Acupuncture And Moxibustion. 23:12-13.

[227]石红, 庄海春. (2004)针药结合治疗椎动脉型颈椎病的疗效观察. 上海针灸杂志 23:16-18.

[227]Shi Hong, Zhuang Hai-chun. (2004)Observations on the Curative Effect of Combined Acupuncture and Medicine on Vertebroarterial Cervical Spondylopathy. Shanghai Journal of  Acupuncture And Moxibustion. 23:16-18.

[228]梁谊深, 罗莎. (2010)指针配合穴位埋线治疗便秘型肠易激综合征疗效观察. 上海针灸杂志 29(3):168-169.

[228]LIANG Yi-shen, LUO Sha. (2010)Observations on the Efficacy of Finger Acupuncture plus Acupoint Catgut Embedding in Treating Irritable Bowel Syndrome of Constipation Type.

Shanghai Journal of  Acupuncture And Moxibustion. 29(3):168-169.

[229]李振楩, 吕慎从. (2010)指针治疗脾胃气虚型功能性消化不良疗效观察. 上海针灸杂志 29:165-167.

[229]LI Zhen-pian, LV Shen-cong. (2010)Observation on the Therapeutic Effect of Finger Acupuncture on Functional Dyspepsia of Plienogastric qi Deficiency Type. Shanghai Journal of  Acupuncture And Moxibustion. 29:165-167.

[230]孔祥飞. (2009)滞针刺法治疗肩关节周围炎疗效观察. 上海针灸杂志 28:648-649.

[230]Kong Xiang-fei. (2009)Observations on the Efficacy of Sticking of Needle in Treating Shoulder Periarthritis. Shanghai Journal of  Acupuncture And Moxibustion. 28:648-649.

[231]詹庆业, 查和萍, 周丽明. (2007)中脘浮线治疗慢性胃炎的临床疗效观察. 上海针灸杂志 26:3-5.

[231]ZHAN Qing-ye, CHA He-ping,. ZHOU Li-ming. (2007)Observations on Clinical Efficacy of Point Zhongwan Catgut Embedding for Treating Chronic Gastritis. Shanghai Journal of  Acupuncture And Moxibustion. 26:3-5.

[232]姚月根, 关建敏. (1998)中药和耳穴贴压治疗头痛的疗效分析. 上海针灸杂志 17:17-18.

[232]Yao Yue-gen, Guan Jian-min. (1998)Clinical analyze on the treatment of headache by push on HEERXUE. Shanghai Journal of  Acupuncture And Moxibustion. 17:17-18.

[233]骆乐, 寿依群, 陈文君. (2006)中药熏蒸加针刺治疗失眠症的临床观察. 上海针灸杂志 25:19-20.

[233]LUO le, SHOU Yi-qun, CHEN Wen-jun. (2006)Clinical Observations on the Treatment of Insomnia by Herbal Fumigation plus Acupuncture. Shanghai Journal of  Acupuncture And Moxibustion. 25:19-20.

[234]石红, 安丽萍, 陈雅民. (2009)中医内外合用法治疗糖尿病周围神经病变. 上海针灸杂志 27:69-70.

[234]Shi Hong, An Li-ping, Chen Ya-ming. (2009)Inside and outside the share of traditional Chinese medicine method to treat diabetes peripheral neuropathy. Shanghai Journal of  Acupuncture And Moxibustion. 27:69-70.

[235]苏苇, 赵宏, 刘元石. (2009)子午流注辨证低频治疗仪治疗膝骨关节炎的随机对照研究. 上海针灸杂志 28:95-97.

[235]SU Wei, ZHAO Hong, LIU Yuan-shi. (2009)Randomized Controlled Trial of Midnight-midday Ebb-flow Syndrome Differentiation Low-frequency Therapeutic Device for the Treatment of Genual Osteoarthritis. Shanghai Journal of  Acupuncture And Moxibustion. 28:95-97.

[236]缪奇祥. (2006)子午流注纳甲法治疗慢性咽炎38例临床研究.上海针灸杂志 25:17-18.

[236]Mu Qi-xiang. (2006)Clinical study on treatment of 38 chronic pharyngitis patients on the theory of midnight-noon ebb-flow. Shanghai Journal of  Acupuncture And Moxibustion. 25:17-18.

[237]邵志林, 费新应, 陈炎生,沈震,张炳煌. (2009)子午流注针法对减少慢性乙型肝炎HBV-YMDD变异的临床观察. 上海针灸杂志 28:694-695.

[237]SHAO Zhi-lin, Fei Xin-ying, CHEN Yan-sheng, SHEN Zhen, ZHANG Bing-huang. (2009)Clinical Observations on Reducing Effect of Midnight-midday ebb flow Acupuncture on Chronic Hepatitis B HBV-YMDD Mutation. Shanghai Journal of  Acupuncture And Moxibustion. 28:694-695.

[238]陈赟, 刘广霞. (2008)子午流注针法治疗胆绞痛临床观察. 上海针灸杂志 27:19-20.

[238]CHEN Yun, LIU Guang-xia. (2008)Clinical Observations on Midnight-noon Ebb-flow Acupuncture for Biliary Colic. Shanghai Journal of  Acupuncture And Moxibustion. 27:19-20.

[239]杨露, 谢娟, 文金莲. (2006)子午流注针法治疗黄褐斑的疗效观察. 上海针灸杂志 25:19-20.

[239]YANG Lu, XIE Juan, WEN Jin-lian. (2006)Observations on the efficacy of acupuncture based on midnight - noon ebb - flow for treating chloasma. Shanghai Journal of  Acupuncture And Moxibustion. 25:19-20.

[240]磨炳森. (1999)综合疗法治疗脂溢性脱发疗效观察. 上海针灸杂志 18:48.

[240]Mo Bing-sen. (1999)Observations on the efficacy of seborrheic alopecia by combined modality therapy. Shanghai Journal of  Acupuncture And Moxibustion. 18:48.

[241]黄聪阳, 胡翔龙, 阮传亮, 苏稼夫,庄垂加. (2005)足太阳膀胱经整体性功能的临床研究. 上海针灸杂志 24:27-29.

[241]HUANG Chong-yang, HU Xiang-long, RUAN Chuan-liang, SU Jia-fu, ZHUANG Chui-jia. (2005)Clinical Study on the Holistic Function of the Bladder Meridian of Foot-taiyang. Shanghai Journal of  Acupuncture And Moxibustion. 24:27-29.

[242]吴巧凤, 徐世珍, 颜贤忠,余曙光,唐勇. (2010)足阳明经穴特异性的代谢组学模式识别研究. 上海针灸杂志 29:552-555.

[242]WU Qiao-feng, XU Shi-zhen,Y AN Xian-zhong, YU Shu-guan, TANG Yong. (2010)Metabonomics and Pattern Recognition Study on the Specificity of Foot-Yangming Meridian Points. Shanghai Journal of  Acupuncture And Moxibustion. 29:552-555.

[243]廖华薇. (2006)作业疗法合针灸治疗肩手综合征45例疗效观察. 上海针灸杂志 25:9-10.

[243]LIAO Huan-wei. (2006)Clinical observations on the efficacy of occupational therapy plus acupuncture for treating reflex sympathetic dystrophy. Shanghai Journal of  Acupuncture And Moxibustion.25:9-10.

[244]俞雁彤. (1994)针刺内关穴治疗冠心病的临床研究. 上海针灸杂志 13:7-8.

[243]Yv Yan-dan. (1994)Clinical study to treatment of coronary heart disease by acupuncture MEIGUANXUE Shanghai Journal of  Acupuncture And Moxibustion. 13:7-8.

[245]龚小燕. (1994)电针配合中药治疗坐骨神经痛258例. 上海针灸杂志, 1994,13:266-267.

[245]Gong Xioa-yan. (1994)The treatment to ischioneuralgia of 258 patients by chinese herbal and acusector. Shanghai Journal of  Acupuncture And Moxibustion. 1994,13:266-267.

[246]孙伟, 黄成钰, 王真,何峰,马晓东,等. (1997)阑尾术中及术后针刺对植物神经功能的影响. 上海针灸杂志 16:14.

[246]Sun Wei, Huang Cheng-yu, Wang Zhen, He Feng, Ma Xiao-dong, et al. (1997)The appendix intraoperative and postoperative effect of acupuncture on plant nerve function. Shanghai Journal of  Acupuncture And Moxibustion. 16:14.

[247]汪令崇. (1997)电针加穴位注射治疗肩周炎50例. 上海针灸杂志 16:20.

[247]Wang Ling-chong. (1997)The treatment to scapulohumeral periarthritis of 50 patients by acusector and point injection. Shanghai Journal of  Acupuncture And Moxibustion. 16:20.

[248]朱必伟. (1997)针灸推拿治疗肩关节周围炎182例. 上海针灸杂志 16:23-24.

[248]Zhu Bi-wei. (1997)The treatment to scapulohumeral periarthritis of 182 patients by acupuncture and tuina.. Shanghai Journal of  Acupuncture And Moxibustion. 16:23-24.

[249]朱洪生, 陆黎, 王晶青.(1997)电针防止胆道手术中胆心反射的观察. 上海针灸杂志 16:11.

[249]Zhu Hong-sheng, Lu-Li, Wang Jing-qing. (1997)observation of electroacupuncture prevents biliary heart reflection During surgery. SHANGHAI JOURNAL OF ACUPUNCTURE AND MOXIBUSTION 16:11.

[250]Cai-Hong, Hu Zhi-hui, Fu Wan-zhen. (1998)observation of different ear poin ts on the effect of lung functions in copd patients, SHANGHAI JOURNAL OF ACUPUNCTURE AND MOXIBUSTION 17:7-8.

[251]侯安乐, 王雷, 卜渊. (1998)药氧针刺治疗多梗塞性痴呆的临床观察. 上海针灸杂志 17:12-13.

[251]Hou An-le, Wang-Lei, Pu-Yuan. (1998)clinical observation of treatment of muliti-in farctional dementia by herbs ,oxygen and acupuncture. SHANGHAI JOURNAL OF ACUPUNCTURE AND MOXIBUSTION 17:12-13.

[252]刘言寿. (1998)电针加气功治疗溃疡性结肠炎疗效分析. 上海针灸杂志,1998.17:19-20.

[252]Liu Yan-shou. (1998)analysis on treatment of ulcerative colitis by electro-acupun cture combining with qi-gong therapy, SHANGHAI JOURNAL OF ACUPUNCTURE AND MOXIBUSTION,17:19-20.

[253]王声强, 宋莉, 孙桂琴. (1998)针刺治疗肩胛肋骨综合征疗效观察. 上海针灸杂志 17:21-22.

[253]Wang Sheng-qiang, Song-Li, Sun Gui-qing. (1998)observation of the curative effect of acupuncture on scapulocostal syndrome, SHANGHAI JOURNAL OF ACUPUNCTURE AND MOXIBUSTION 17:21-22.

[254]于澎, 李春梅, 张士任. (1998)电针治疗泌尿系结石的若干影响因素分析. 上海针灸杂志 17:13-14.

[254]Yu-Peng, Li Chun-mei , Zhang Si-ren. (1998)analysis of correlative factors in acupuncture treatment of urinary lithiasis, SHANGHAI JOURNAL OF ACUPUNCTURE AND MOXIBUSTION 17:13-14.

[255]王大军, 张道敬, 童利民,胡永均,李江明. (1998)电针加颈动脉注药治疗脑梗塞临床疗效观察. 上海针灸杂志 17:5-6

[255]Wang Da-jun, Zhang Dao-jing, Tong Li-ming, Hu Yong-jun, Li Jiang-ming . (1998)clinical observation of the curative effect of electro-acupuncture plus carotid drug injection on cerebral in farction , SHANGHAI JOURNAL OF ACUPUNCTURE AND MOXIBUSTION 17:5-6

[256]孙伟, 何锋, 黄成钰,王真,翟珠妹,等. (1998)针刺、中药对胆道“T”字管外引流胆汁量的影响. 上海针灸杂志 17:9-10.

[256]Sun-Wei, He-Feng Huang, Chen-yu, Wang-Zhen, Zhai Zhu-mei , Zhang Rong-xiang. (1998)the effects ofacupuncture and Chinese materia medica on the amount of bile drained from gall duct t-shaped tube, SHANGHAI JOURNAL OF ACUPUNCTURE AND MOXIBUSTION 17:9-10.

[257]宋颖, 梁浩荣. (1999)头皮针治疗脑卒中后抑郁症疗效观察. 上海针灸杂志 18:8-9.

[257]Song-Ying, Liang Hao-rong. (1999)observation of the curative effect of scalp-acupuncture on cerebral postapoplectic depression, SHANGHAI JOURNAL OF ACUPUNCTURE AND MOXIBUSTION 18:8-9.

[258]王祥瑞, 杭燕南, 孙大金,张马忠,许灿然. (2000)针刺对心脏手术病人功能调节的观察. 上海针灸杂志 18:6-7.

[258]Wang Xiang-rui, Hang Yan-nan, Shun Da-jin, Zhang Maz-hong, Xu Chan-ran. (2000)clinical observation on the adjustment of body’s function by acupuncture in patients undergoing car-diac surgery, SHANGHAI JOURNAL OF ACUPUNCTURE AND MOXIBUSTION 18:6-7.

[259]张道武, 梁晓菲, 王秋景, 郭彤 . (2000)半夏泻心汤配针灸治疗眩晕临床观察. 上海针灸杂志 19:20-21.

[259]Zhang Dao-wu, Liang Xiao-fei, Wang Qiu-jing, Guo-Tong. (2000)clinical observation on the treatment of vertigo by pinellia decoction for purging stomach-fire plus acupuncture, SHANGHAI JOURNAL OF ACUPUNCTURE AND MOXIBUSTION 19:20-21.

[260]陈雁南, 韩霞, 邹海珠,蒋广华. (2000)针刺对急性胆囊炎患者胆囊收缩功能的影响. 上海针灸杂志 19:12-13.

[260]Chen Yan-nan, Han-Xia, Zou Hai-zhu, JIANG Guang-hua. (2000)the influence of acupuncture on biliary traction in acute cholecystitis patients, SHANGHAI JOURNAL OF ACUPUNCTURE AND MOXIBUSTION 19:12-13.

[261]欧阳钢, 冯军, 张春英,杨宇,于致顺. (2000)针刺对急性脑梗塞患者体感诱发电位的影响. 上海针灸杂志 19:8-9.

[261]Ou Yang-gang, Feng Jun, Zhang Chun-ying, Yang Yu, Yu Zhi-shun. (2000)the influence of acupuncture on somatosensory evoked potential in patients with acute cerebral in-faretion, SHANGHAI JOURNAL OF ACUPUNCTURE AND MOXIBUSTION 19:8-9.

[262]姜杰, 常向明, 唐勇. (2000)针刺对颈椎病患者LPO、SOD代谢的影响. 上海针灸杂志, 2000,19:11-12.

[262]Jiang-Jie, Chang Xiang-ming, Tang-Yong. (2000)TCM Department.influence of acupuncture on metabolism of lpo and sod in patients with cervical spondylosis, SHANGHAI JOURNAL OF ACUPUNCTURE AND MOXIBUSTION 2000,19:11-12.

[263]欧阳颀, 李忠仁, 穆艳云,龙浩文,赵涤,等. (2000)电针合并奋乃静治疗阿尔茨海默病精神症状的临床观察. 上海针灸杂志, 2000,19:16-17.

[263]Ou Yan-xin, Li Zhong-ren, Mu Yan-yun, Rong Hao-wen, Zhao-Di, WU-Ji. (2000)Theclinical observation of electric acupuncture combined with fentazin in treating mental symptom of alzheimer’s,SHANGHAI JOURNAL OF ACUPUNCTURE AND MOXIBUSTION 2000,19:16-17.

[264]陆黎, 朱洪生, 郭圆,毕宏达.(2001)电针应用于胆道手术中的临床观察. 上海针灸杂志 19,15-16.

[264]Lu-Li, ZhuHong-sheng, Guo-Yuan, BiHong-da, Long-Hua. (2001)clinica observation of electro-acupuncture in gallbladder surgery, SHANGHAI JOURNAL OF ACUPUNCTURE AND MOXIBUSTION 19,15-16.

[265]贾杰, 胡少辉, 毛光兰. (2001)针刺治疗创伤后颈脑综合征临床观察. 上海针灸杂志 20:7-8.

[265]Jia-jie, Hu Shao-hui, Mao Guang-lan. (2001)clinical observation on acupuncture treatment of posttracumatic cervicocranial syndrome, SHANGHAI JOURNAL OF ACUPUNCTURE AND MOXIBUSTION 20:7-8.

[266]符文彬, 樊莉, 李伟雄. (2001)眼针治疗急性脑梗塞. 上海针灸杂志 20:14-15.

[266]Fu Wen-bin, Fan-Li, LI Wei-xiong. (2001)treatment of acute cerebral infarction by eye acupuncture, SHANGHAI JOURNAL OF ACUPUNCTURE AND MOXIBUSTION 20:14-15.

[267]姜玲珍, 张莲瑛. (2001)电针加耳压治疗神经根型颈椎病47例. 上海针灸杂志 20:20-21.

[267]Jiang Ling-zhen, ZHANG Lian-ying. (2001)therapeutic effect of electroacupuncture and pressure of otopoint on cervical spondylopathy 20:20-21.

In 47 cases,SHANGHAI JOURNAL OF ACUPUNCTURE AND MOXIBUSTION

[268]吴耀持, 张一峰. (2001)电针结合药敷治疗腰椎间盘突出症160例. 上海针灸杂志 20:18-19.

[268]Ng Yiu-chi, Zhang Yi-feng. (2001)Treatment of prolapse of lumber intervertebral disc by combination of electro-acupuncture and medi-cal application, SHANGHAI JOURNAL OF ACUPUNCTURE AND MOXIBUSTION 20:18-19.

[269]蒋滨, 刘萌, 李蠡. (2001)针灸为主治疗腰椎间盘突出症35例. 上海针灸杂志 20:20-21.

[269]Jiang-Bin, Liu-Meng, LI -Li. (2001)treatment of 35 cases of prolapse lunbar intervertebral disc mainly by acupuncture, SHANGHAI JOURNAL OF ACUPUNCTURE AND MOXIBUSTION 20:20-21

[270]余明哲, 朱忠春, 彭美凤. (2001)针刺治疗十二指肠溃疡的腧穴特异性研究. 上海针灸杂志 20:4-6.

[270]Yu Ming-zhe, Zhu Zhong-chun, Pen Mei-feng, Yang Guang-zheng. (2001)the acupoint specificity in the treatment of duodenal ulcer, SHANGHAI JOURNAL OF ACUPUNCTURE AND MOXIBUSTION 20:4-6.

[271]莫飞智, 李建强, 储莉,赖新生,张家维,等. (2001)电针对血管性痴呆患者智力结构的影响. 上海针灸杂志 20:6-8.

[271]Mo Fei-zhi, Li Jian-qiang, Chu-Li, Lai Xin-sheng, Zhang Jia-wei, Liu Song-hao 1 1. (2001)the influence of electroacupuncture on intelligence structure in patients withvascular dementia, SHANGHAI JOURNAL OF ACUPUNCTURE AND MOXIBUSTION 20:6-8.

[272]周炜, 王丽平, 刘泓,边垠. (2002)头针对脑梗塞患者血清肿瘤坏死因子的影响. 上海针灸杂志 21:11-12.

[272]Zhou-Wei, Wang Li-ping, Liu-Hong, Bian-Eun. (2002)Influence of Scalp Acupuncture on Serum Tumor Necrosis Factor in Patients with Acute Cerebral Infarction, SHANGHAI JOURNAL OF ACUPUNCTURE AND MOXIBUSTION 21:11-12.

[273]戴宁, 柴雁. (2002)针刺加卧位手法牵引治疗颈性眩晕88例. 上,海针灸杂志, 2002,21:18-19.

[273]Dai-Ning ,Cai-Yan. (2002)Treatment of 175 Cervical Vertigo Cases by Acupuncture and Clinostatic Manual Traction, SHANGHAI JOURNAL OF ACUPUNCTURE AND MOXIBUSTION 2002,21:18-19.

[274]刘琪. (2002)针刺治疗带状疱疹后顽固性神经痛42例. 上海针灸杂志 21:26-27.

[274]Liu-Qi. (2002)Acupuncture Treatment of 42 Cases of Intractable Neuralgia Subsequent to Herpes Zoster, SHANGHAI JOURNAL OF ACUPUNCTURE AND MOXIBUSTION 21:26-27.

[275]倪至臻. (2002)针药治疗足跟痛的临床观察. 上海针灸杂志 21:17-18.

[275]Ni Zhi-zhen. (2002)Clinical Observations on the Treatment of Painful Heel with Acupuncture and Meidicament, SHANGHAI JOURNAL OF ACUPUNCTURE AND MOXIBUSTION 21:17-18.

[276]殷之放, 汪司右, 肖达. (2002)项针疗法治疗高血压病临床观察. 上海针灸杂志 21:10-11.

[276]Ying Zhi-fang, Wang Si-you, Xiao-Da. (2002)Clinical Observations on the Treatment of Essential Hypertension with Nape Acupuncture, SHANGHAI JOURNAL OF ACUPUNCTURE AND MOXIBUSTION 21:10-11.

[277]邵敏, 温凌洁, 黄万义. (2002)针刺配合耳穴贴压治疗失眠症的疗效观察. 上海针灸杂志 21:14-15.

[277]Shao-Min, Wen Ling-jie. (2002) Huang WanyiThe Observations on the Curative Effect of Acupuncture Plus Auricular-plaster Therapy on Insomnia, SHANGHAI JOURNAL OF ACUPUNCTURE AND MOXIBUSTION 21:14-15.

[278]张洪, 何竟. (2002)电针治疗抑郁症的疗效观察. 上海针灸杂志 21:25-26.

[278]Zhang-Hong, He-Jing. (2002)Observations on the Curative Effect of Electroacupuncture on Melancholia, SHANGHAI JOURNAL OF ACUPUNCTURE AND MOXIBUSTION 21:25-26.

[279]金肖青, 宋双临, 俞迈红,鲁冰丰. (2002)头穴电针结合穴位注射治疗血管性痴呆临床观察. 上海针灸杂志 21:7-9.

[279]Jin Xiao-qing, Song Shuang-lin, Yu Mai-hung , Lu Bing-feng. (2002)Clinical Observations on the Treatment of Vascular Dementia by Scalp Electroacupuncture Plus Acupoint Injection, SHANGHAI JOURNAL OF ACUPUNCTURE AND MOXIBUSTION

21:25-26.

[280]涂小华,李芳,张定强. (2002)齐刺治疗椎动脉型颈椎病的临床观察. 上海针灸杂志 21:10-11.

[280]Tu Xiao-hua, Li-Fang, Zhang Ding-jiang. (2002)Clinical Observations on Triple Puncture Treatment of Vertebroarterial Cervical Spondylopathy, SHANGHAI JOURNAL OF ACUPUNCTURE AND MOXIBUSTION 21:10-11.

[281]蔡国伟,贾玉英,戴春林. (2002)膈俞穴活血化瘀作用临床研究. 上海针灸杂志 21:1-2.

[281]Cai Guo-wei, Jia Yu-ying, Dai Chun-lin. (2002)Clinical Research on the Role of Point Geshu in Activating Blood and Removing Stasis, SHANGHAI JOURNAL OF ACUPUNCTURE AND MOXIBUSTION 21:1-2.

[282]倪承浩. (2002)艾灸膻中穴为主治疗心肌缺血的疗效观察. 上海针灸杂志 21:17-18.

[282]Ni Cheng-hao. (2002)Observations on the Curative Effect of Point Shanzhong Moxibustion on Myocardial Ischemia, SHANGHAI JOURNAL OF ACUPUNCTURE AND MOXIBUSTION 21:17-18.

[283]周红, 童稳圃, 施伶俐. (2003)针刺对肺癌手术患者T淋巴细胞免疫功能的影响. 上海针灸杂志 22:34-35.

[283]Zhou-Hong, Tong Wei-pu, Shi Shi-li. (2003)Influence of of Acupuncture on Cellular Immunity of T Lymphocyte in Patients with Lung Cancer Operation, SHANGHAI JOURNAL OF ACUPUNCTURE AND MOXIBUSTION 22:34-35.

[284]王顺, 蔡玉颖. (2003)透穴疗法治疗偏头痛的临床疗效与机理探讨. 上海针灸杂志 22:18-20.

W[284]ang-Shun, Cai Yu-ying. (2003)The Clinical Effect of Point-through-Point Acupuncture on Migraine and the Investigation into its Mechanism, SHANGHAI JOURNAL OF ACUPUNCTURE AND MOXIBUSTION 22:18-20.

[285]沈虹, 胡大文.(2003)针灸加超短波治疗慢性盆腔炎55例疗效分析. 上海针灸杂志 22:29-30.

[285]Shen-Hong, Hu-Dawen. (2003)Analysis of the Curative Effect of Acupmoxi Plus Ultra-short Wave on 55 Patients with Chronic Pelvic Inflammation, SHANGHAI JOURNAL OF ACUPUNCTURE AND MOXIBUSTION 22:29-30.

[286]张丽,李东书,盛丽. (2003)磁针治疗单纯性肥胖症并发高脂血症的疗效观察. 上海针灸杂志 22:7-9.

[286]Zhang-Li, Li Dong-Shu, Sheng-Li. (2003)Observations on the Effect of Magnetic Needle Treatment on Simple Obesity Complicated with Hyperlipidemia, SHANGHAI JOURNAL OF ACUPUNCTURE AND MOXIBUSTION 22:7-9.

[287]唐济湘, 关念红, 李林,刘菊芬. (2003)电针治疗中风后抑郁的疗效与对患者生存质量的影响. 上海针灸杂志 22:12-14.

[287]Tang Ji-xiang, Guan Nian-hong, Li-Lin, Liu Ju-fen, (2003)Influence of Electroacupuncture on Life Quality in Patients with Post-apoplectic Depression, SHANGHAI JOURNAL OF ACUPUNCTURE AND MOXIBUSTION 22:12-14.

[288]竺炯, 郭胜, 赖永贤,沈卫东. (2003)麦粒灸治疗急性期带状疱疹40例疗效观察. 上海针灸杂志 22:10-11.

[288]Zhu-Jiong, Guo-Sheng, Lai Yong-xian, Shen Wei-dong. (2003)Observations on the Curative Effect of Grain-sized Moxibustion on Acute Herpes Zoster 40 Cases, SHANGHAI JOURNAL OF ACUPUNCTURE AND MOXIBUSTION 22:10-11.

[289]乐旭华, 傅莉萍, 王瑞华. (2003)电针结合高压氧治疗突发性耳聋临床观察. 上海针灸杂志 22:22-23.

[289]Xu-hua, Fu Li-ping, Wang Rui-hua. (2003)Observations on the Curative Effect of Grain-sized Moxibustion on Acute Herpes Zoster 40 Cases, SHANGHAI JOURNAL OF ACUPUNCTURE AND MOXIBUSTION 22:22-23.

[290]黄勇, 刘桂珍. (2003)水针治疗呃逆临床观察. 上海针灸杂志 22:17-18.

[290]Huang-Yong, Liu Gui-zhen. (2003)Clincial Observations on Treatment of Hiccup by Acupoint Injection, SHANGHAI JOURNAL OF ACUPUNCTURE AND MOXIBUSTION 22:17-18.

[291]张银开, 张明选, 吴强. (2003)针药结合治疗慢性前列腺炎80例临床观察. 上海针灸杂志 22:19-20.

[291]Zhang Yin-kai, Zhang Ming-xuan, Wu-Qiang. (2003)Clinical Observations on Treatment of 80 Chronic Prostatitis Patients with Combined Acupuncture and Medicine, SHANGHAI JOURNAL OF ACUPUNCTURE AND MOXIBUSTION 22:19-20.

[292]倪至臻, 李丁霞, 周景炜. (2003)针刺治疗目赤肿痛临床疗效观察. 上海针灸杂志 22:21-22.

[292]Ni Zhi-hen, Li Ding-xia, Zhou Jing-wei. (2003)Clinical Observations on the Curative Effect of Acupuncture on Conjunctive Congestion with Swelling and Pain, SHANGHAI JOURNAL OF ACUPUNCTURE AND MOXIBUSTION 22:21-22.

[293]袁红香, 喻志冲, 成慧贞.(2003)艾灸对宫颈癌放疗患者血红蛋白的影响. 上海针灸杂志 22:33-34.

[293]Yuan Hong-xiang, Yu Zhi-chong, Cheng Hui-zhen, (2003)Influence of Moxibustion on Hemoglobin During Radiotherapy in Patients with Cervical Cancer, SHANGHAI JOURNAL OF ACUPUNCTURE AND MOXIBUSTION 22:33-34

[294]丰培学. (2003)长强穴埋线治疗便秘60例临床观察. 上海针灸杂志 22:14-15.

[294]Wei Pei-xue. (2003)Clinical Observations on the Treatment of 60 Constipation Patient by Catgut Embedding at Point Changqiang, SHANGHAI JOURNAL OF ACUPUNCTURE AND MOXIBUSTION 22:14-15.

[295]老锦雄, 邓聪. (2003)电温针配合中药治疗膝骨关节炎临床观察. 上海针灸杂志 22:26-27.

[295]Lao Jin-xiong, Deng-Cong. (2003)Clinical Observations on the Treatment of Genual Osteoarthritis by Electroacupuncture and Moxibustion Plus Chinese Medicine, SHANGHAI JOURNAL OF ACUPUNCTURE AND MOXIBUSTION 22:26-27.

[296]傅莉萍, 项琼瑶, 沈小珩.(2003)针刺配合穴位贴膜治疗中风偏瘫的疗效观察. 上海针灸杂志 22:9-11.

[296]Fu Li-ping, Xiang Qiong-yao, Sen Xiao-heng. (2003)Observations on the Curative Effect of Acupuncture Plus Acupoint Application on Apoplectic Hemiplegia, SHANGHAI JOURNAL OF ACUPUNCTURE AND MOXIBUSTION 22:9-11.

[297]孙冬梅, 单秋华, 吴富东. (2003)耳穴贴压与针刺治疗女性更年期综合征疗效对比观察. 上海针灸杂志 22:38-40.

[297]Sun Dong-mei, Shan Qiu-hua, Wu Fu-dong. (2003)A Comparative Observation on the Therapeutic Effects of Tracting Female Menopausal Syndrome with Auricular-point-pressing Therapy and Acupuncture, SHANGHAI JOURNAL OF ACUPUNCTURE AND MOXIBUSTION 22:38-40.

[298]陆黎, 朱洪生. (2003)电针对术中血糖的影响. 上海针灸杂志 22:41-42.

[298]Lu-Li, Zhu Hong-sheng. (2003)The Influence of Electro-acupuncture over the Blood Sugar during Operation, SHANGHAI JOURNAL OF ACUPUNCTURE AND MOXIBUSTION22:41-42.

[299]石奕丽. (2003)针刺配合中药熏洗治疗中风偏瘫后患肢水肿43例. 上海针灸杂志,2003，22:1-12.

[299]Shi Yi-li. (2003)Treatment of Edema by Acupuncture Plus Herbal Fumigating and Washing in 43 cases of Postapoplectic Hemiplegia, SHANGHAI JOURNAL OF ACUPUNCTURE AND MOXIBUSTION2003，22:1-12.

[300]迟旭,金泽,崔淑子,包大鹏,孙远征. (2003)头针加中药治疗多动秽语综合征临床研究.上海针灸杂志 2(10):15-16.

[300]Chi-Xu, Jin-Ze, Cui Shu-zi, Bao Da-peng, Sun Yuan-zheng. (2003)Clinical Study on Treatment of Hyperactive and Coprophasic Syndrome with Scalp Acupuncture Plus Chinese Medicine, SHANGHAI JOURNAL OF ACUPUNCTURE AND MOXIBUSTION 2(10):15-16.

[301] 曹友德,王敏华,王敏, 华启海,赵志. (2003)电针夹脊穴治疗根性坐骨神经痛临床观察. 上海针灸杂志，2003,22:19-20.

[301] Cao You-de, Wang Min-hua, Wang-Min. (2003)Clinical Observations on Treatment of Root Sciatica by Jiaji Point Lectroacupuncture, SHANGHAI JOURNAL OF ACUPUNCTURE AND MOXIBUSTION2003 ,22:19-20.

[302] 黄海明,高强.(2003)针刺加TDP为主治疗颞下颌关节综合征疗效观察. 上海针灸杂志 22:23-24.

[302] Huang Hai-ming, Gao-Qiang. (2003)Observations on the Effect of Acupuncture Plus TDP as Main Therapy in Treatment of Temporomandibular Joint Syndrome, SHANGHAI JOURNAL OF ACUPUNCTURE AND MOXIBUSTION 22:23-24.

[303] 李景良. (2003)针刺治疗中风后语言障碍68例临床观察. 上海针灸杂志 22:5-6.

[303] Li Jing-liang. (2003)Clinical Observations on Acupuncture Treatment of 68 Patients with Postapoplectic Dysphasia, SHANGHAI JOURNAL OF ACUPUNCTURE AND MOXIBUSTION 22:5-6.

[304] 陈小凯,吴虹. (2003)针刺华佗夹脊穴治疗脑血管意外后偏瘫疗效观察. 上海针灸杂志 22:9-10.

[304] Chen Xiao-kai, Wu-Hong. (2003)Clinical Observations on the Curative Effect of Needling Jiaji Points on Hemiplegia after Cerebrovascular Accidents, SHANGHAI JOURNAL OF ACUPUNCTURE AND MOXIBUSTION 22:9-10.

[305] 张庆华,朱洪生,陆黎.(2003)电针对术后使用硬膜外镇痛泵胃肠蠕动的观察. 上海针灸杂志 22:28-29.

[305]Zhang Qing-hua, Zhu Hong-sheng, Lu Li.(2003)Influence of Electro-acupuncture over Gastrointestinal Peristalsis in Postoperative Epidural Anaesthesia Pump, SHANGHAI JOURNAL OF ACUPUNCTURE AND MOXIBUSTION 22:28-29.

[306] 叶立汉,胡亚明.(2003)电针对颈椎病康复效果的临床观察. 上海针灸杂志 22: 17-18.

[306] Ye Li-han, Hu Ya-ming. (2003)Clinical Observations on the Curative Effect of Acupuncture on Cervical Spondylopathy, SHANGHAI JOURNAL OF ACUPUNCTURE AND MOXIBUSTION 22: 17-18.

[307] 黄勇. (2003)水针结合推拿治疗颈椎病的临床观察. 上海针灸杂志 22:19-20.

[307] Huang-Yong. (2003)Clinical Observation on the Treatment of Cervical Spondyiopathy by Acupoint Injection Plus Massotherapy, SHANGHAI JOURNAL OF ACUPUNCTURE AND MOXIBUSTION 22:19-20.

[308] 侯凤琴. (2003)头体针并用治疗中风后遗症临床疗效观察. 上海针灸杂志 22:7-8.

[308] Hou Feng-qin. (2003)Observations on the Clinical Effect of Combined Scalp and Body Acupuncture on Apoplectic Sequela, SHANGHAI JOURNAL OF ACUPUNCTURE AND MOXIBUSTION 22:7-8.

[309] 郭淑颖,孙远征. (2004)经前期综合征35例针药对比观察. 上海针灸杂志 23:5-6.

[309] Guo Shu-ying, Sun Yuan-zheng. (2003)Comparison Between Acupuncture and Medication in Treatment of Premenstrual Syndrome, SHANGHAI JOURNAL OF ACUPUNCTURE AND MOXIBUSTION 23:5-6.

[310] 周佐涛,林晓山. (2004)腹针治疗腰椎间盘突出症42例疗效观察. 上海针灸杂志 23:13-14.

[310] Zhou Zuo-tao, Lin Xiao-Shan. (2004)Observations on the Effect of Abdominal Acupuncture in Treating 42 Cases of Lumbar Intervertebral Disc Protrusion, SHANGHAI JOURNAL OF ACUPUNCTURE AND MOXIBUSTION 23:13-14.

[311] 周光涛,杨翊. (2004)远道辨经巨刺法治疗肩关节周围炎76例疗效观察. 上海针灸杂志 23:15-16.

[311] Zhou Guang-tao, Yang-Yi. (2004)Clinical Observations on the Treatment of 76 Shoulder Periarthritis Cases by Meridional Differentiation-based Distant Opposing Acupuncture, SHANGHAI JOURNAL OF ACUPUNCTURE AND MOXIBUSTION 23:15-16.

[312] 赵晓梅,李冬. (2004)絮刺火罐疗法治疗颈椎病疗效观察141例. 上海针灸杂志 23:16-17.

[312] Zhao Xiao-mei, Li-Dong. (2004)Observations on the Effect of Blood-letting Puncturing and Cupping in the Treatment of Cervical Spondylopathy, SHANGHAI JOURNAL OF ACUPUNCTURE AND MOXIBUSTION 23:16-17.

[313] 黄泳,龚伟,邹军,赵仓焕. (2004)头针治疗抑郁发作SCL-90评定结果分析. 上海针灸杂志 23:5-7.

[313] Huang-Yong, Gong-Wei, Zou-Jun, Zhao Cang huan. (2004)A Clinical Judgment of SCL-90 on Treating 50 cases of Depression with Scalp Acupuncture, SHANGHAI JOURNAL OF ACUPUNCTURE AND MOXIBUSTION 23:5-7.

[314] 王斌. (2004)项丛刺合围针治疗偏头痛. 上海针灸杂志 23:14-15.

[314] Wang-Bin. (2004)Nape Multiple Acupuncture Plus Surrounding Acupuncture for Migraine, SHANGHAI JOURNAL OF ACUPUNCTURE AND MOXIBUSTION 23:14-15.

[315] 张燕. (2004)头皮针围刺治疗脑出血30例疗效观察. 上海针灸杂志 23:5-6.

[315] Zhang-Yan. (2004)Observations on the Curative Effect of Scalp Surrounding Acupuncture on 30 Cerebral Hemorrhage Patients, SHANGHAI JOURNAL OF ACUPUNCTURE AND MOXIBUSTION 23:5-6.

[316] 吴耀持,张必萌.(2004)电针治疗腰椎间盘突出症的临床研究. 上海针灸杂志 23:15-17.

[316] Wu Yao-chi, Zhang Bi-meng. (2004)Clinical Observations on Electroacupuncture Treatment of Lumbar Intervertebral Disc Protrusion, SHANGHAI JOURNAL OF ACUPUNCTURE AND MOXIBUSTION 23:15-17.

[317] 邢崇慧,李冬梅,张蔚. (2004)电针配合超声波治疗运动员肌肉损伤108例. 上海针灸杂志 23:18-19.

[317] Xing Chong-hui, Li Dong-mei, Zhang-Wei. (2004)Treatment of 108 Cases of Athletic Muscular Injury by Electroacupuncture plus Ultrasonic Waves, SHANGHAI JOURNAL OF ACUPUNCTURE AND MOXIBUSTION 23:18-19.

[318] 周开斌,陈尚杰,黄鼎坚,庞勇,刘彪 . (2004)益肾养心通督针法对中风患者载脂蛋白的影响附27例临床疗效观察. 上海针灸杂志 23:3-5.

[318] Zhou Kai-bin, Chen Shang-jie, Huang Ding-jian, Pang-Yong ,Liu-Biao, ( 2004)Observations on the Effects of Kidney-reinforcing,Heart-nourishing and Du meridian-unblocking Acupuncture on Stroke and Apolipoprotein, SHANGHAI JOURNAL OF ACUPUNCTURE AND MOXIBUSTION 23:3-5.

[319] 周智梁,杨家荣.(2004)刺血疗法治疗脑血管疾病偏身感觉障碍疗效观察.上海针灸杂志 23:6-7.

[319] Zhou Zhi-liang, Yang Jia-rong. (2004)Observations on the Clinical Effect of Pricking Blood Therapy on Unilateral Sensory Disturbance in Cerebrovascular Diseases,SHANGHAI JOURNAL OF ACUPUNCTURE AND MOXIBUSTION 23:6-7.

[320] 赵晓峰,宋文俊,李庆海, 张桦,周运峰,等. (2004)通关利窍针法治疗中风致假性延髓麻痹临床观察. 上海针灸杂志 23:8-10.

[320] Zhao Xiao-feng, Song Wen-jun, Li Qing-hai, Zhang-Hua, Zhou Yun-feng. (2004)Clinical Observations on the Treatment of Apoplectic Psudobulbar Paralysis by Brain-refreshing and Orifice-opening Acupuncture , SHANGHAI JOURNAL OF ACUPUNCTURE AND MOXIBUSTION 23:8-10

[321] 倪卫民,沈洁 . (2004)刺络拔罐法对减低中风后上肢肌张力增高的临床研究. 上海针灸杂志 23:10-11.

[321]Ni Wei-min, Shen-Jie, (2004)Clinical Study on the Reduction of High Upper-limb Myodynamia by Blood-letting Puncture and Cupping in Cerebrovascular Accident Patients, SHANGHAI JOURNAL OF ACUPUNCTURE AND MOXIBUSTION 23:10-11.

[322] 米建平,朱晓平. (2004)舌针为主治疗中风失语症46例临床观察. 上海针灸杂志 23:8-9.

[322] Mi Jian-ping, Zhu Xiao-ping. (2004)Clinical Observations on the Treatment of 43 Apoplectic Aphasia Patients by Tongue Acupuncture as A Main Method, SHANGHAI JOURNAL OF ACUPUNCTURE AND MOXIBUSTION 23:8-9.

[323] 胡德志,江澄川,曹小定,乔健,尹士杰. (2004)针刺对脑肿瘤术后患者血清IL-6、NSE水平的影响. 上海针灸杂志 23:11-13.

[323] Hu De-zhi, Jiang Cheng-chuan, Cao Xiao-ding, Qiao-Jian, Yin Shi-jie. (2004)The Influence of Acupuncture on Serum IL-6 and NSE Levels After Encephaloma Operation, SHANGHAI JOURNAL OF ACUPUNCTURE AND MOXIBUSTION 23:11-13.

[324] 郑晓,车涛.(2004)电针治疗髋关节骨关节炎的临床观察. 上海针灸杂志 23:16-17.

[324] Zhen-Xiao, Che-Tao. (2004)Clinical Observations on Electroacupuncture Treatment of Hip Osteoarthritis, SHANGHAI JOURNAL OF ACUPUNCTURE AND MOXIBUSTION 23:16-17.

[325] 谢冬玲,朱丽芳,刘惠宇,曾春英,王素霞 ,等.(2004)电针对急性脑梗死患者下肢功能的影响. 上海针灸杂志 2004,23:21-22.

[325] Xie Dong-ling, Zhu Li-fang, Liu Hui-yu, Zeng Chun-ying, Wang Su-xia. (2004)The Effect of Electroacupuncture Plus Lower Limb Function Intensive Training on Lower Limb Function in Patients with Acute Cerebral Infarction, SHANGHAI JOURNAL OF ACUPUNCTURE AND MOXIBUSTION 2004,23:21-22.

[326] 聂志华,钟志伦. (2004)靳三针治疗脑血管性痴呆的临床观察. 上海针灸杂志 23:5-6.

[326] Nie Zhi-hua, Zhong Zhi-lun. (2004)Clinical Observations on the Treatment of Cerebrovascular Dementia by Jin Three-needle Therapy, SHANGHAI JOURNAL OF ACUPUNCTURE AND MOXIBUSTION 23:5-6.

[327] 吴耀持. (2004)耳穴平喘点治疗哮喘及其对FEV1与PEF的影响. 上海针灸杂志 2004,23:7-8.

[327] Wu Yao-chi. (2004)Treatment of Asthma at Asthma-relieving Earpoint and its Influence on FEV1 and PEF, SHANGHAI JOURNAL OF ACUPUNCTURE AND MOXIBUSTION 2004,23:7-8.

[328] 王琴玉,袁青,冯健强,罗广锋,靳瑞. (2004)速刺与留针治疗脑性瘫痪60例对比观察. 上海针灸杂志 23:15-17.

[328] Wang Qin-yu, Yuan-Qing, Feng Jian-qiang, Luo Guang-feng, Rui-Jin. (2004)Observations on a Comparison of Quick Needling and Retained Needle for Treating 60 Cerebral Palsy Cases, SHANGHAI JOURNAL OF ACUPUNCTURE AND MOXIBUSTION 23:15-17.

[329] 殷之放,肖达. (2004)针刺加埋针治疗血管性头痛临床观察. 上海针灸杂志 23:8-9.

[329] Ying Zhi-fang, Xiao-Da. (2004)Clinical Observation on the Treatment of Vascular Headache by Acupuncture Plus Needle-embedding Therapy, SHANGHAI JOURNAL OF ACUPUNCTURE AND MOXIBUSTION 23:8-9.

[330] 汪崇淼. (2005)电针与中药内服治疗股骨头缺血性坏死30例. 上海针灸杂志 24:15-16.

[329] Wang Chong-miao. (2005)Clinical Observations on the Treatment of Ischemic Necrosis of Femoral Head with Electroacupuncture and Oral Chinese Medicine, SHANGHAI JOURNAL OF ACUPUNCTURE AND MOXIBUSTION 24:15-16.

[331] 宁泽晖,谢敏,冯玲媚. (2005)针刺加艾绒及TDP照射治疗中风后瘫侧肢体肿胀的临床观察.上海针灸杂志 24:11-12.

[331] Ning Ze-hui, Xie-Min, Feng Ling-mei, (2005)Clinical Observation on the Treatment of Post-apoplectic Paralytic Limb Swelling with Acupuncture plus Moxa and TDP Irradiation, SHANGHAI JOURNAL OF ACUPUNCTURE AND MOXIBUSTION 24:11-12.

[332] 路瑜,苟春雁,田丰玮. (2005)早期头电针为主治疗高血压脑出血临床观察. 上海针灸杂志 24:13-14.

[332] Lu-Yu, Gou Chun-yan, Tian Feng-wei. (2005)Clinical Observations on the Treatment of 30 Hypertensive Cerebral Hemorrhage Cases with Early-stage Scalp Electroacupuncture, SHANGHAI JOURNAL OF ACUPUNCTURE AND MOXIBUSTION 24:13-14.

[333] 孙阁,赵援非. (2005)头体针治疗脑梗死致麻痹性斜视临床观察. 上海针灸杂志 24:15-16.

[333] Ge-Sun, Zhao Yuan-fei. (2005)Clinical Observations on the Treatment of Cerebral Infarction-caused Paralytic strabismus with Scalp and Body Acupuncture, SHANGHAI JOURNAL OF ACUPUNCTURE AND MOXIBUSTION 24:15-16.

[334] 陈兴华,赖新生. (2005)针刺对中风假性球麻痹患者血液粘稠度的影响. 上海针灸杂志 24:7-8.

[334]Cheng Xing-hua, Lai Xin-sheng. (2005)The Influence of Acupuncture on Blood Viscosity in Apoplectic Pseudobulbar Paralysis, SHANGHAI JOURNAL OF ACUPUNCTURE AND MOXIBUSTION 24:7-8.

[335] 庞勇,陈尚杰,周开斌,黄鼎坚. (2005)风府穴对针刺治疗假性球麻痹效果的影响. 上海针灸杂志 24:9-10.

[335] Pang-Yong, Chen Shang-jie, Zhou Kai-bin, Ding Jian-huang. (2005)The Influence of Point Fengfu on Acupuncture Treatment of Pseudobulbar Paralysis, SHANGHAI JOURNAL OF ACUPUNCTURE AND MOXIBUSTION 24:9-10.

[336] 牛俊明,吴玲,韩常青. (2005)穴位注射配合头体针治疗中风运动障碍临床研究. 上海针灸杂志 24:10-11.

[336] Niu Jun-ming, Eu-Ling, Han Chang-qing. (2005)Clinical Study on the Treatment of Apoplectic Dyskinesia by Point Injection plus Scalp and Body Acupuncture ,SHANGHAI JOURNAL OF ACUPUNCTURE AND MOXIBUSTION 24:10-11.

[337] 蒋戈利,肖蕾,李坚将,夏喜云,陈丽华. (2005)四步针药疗法治疗膝骨性关节炎疗效分析. 上海针灸杂志 24:12-13.

[337] Jiang Ge-li, Xiao-Lei, Li Jian-jiang, Xia Xi-yun, Chen Li-hua. (2005)Analysis of the Efficacy of Four-step Acupuncture and Medicine Therapy for Genual Osteoarthritis, SHANGHAI JOURNAL OF ACUPUNCTURE AND MOXIBUSTION 24:12-13.

[338] 朱洪生. (2005)电针在麻醉诱导与气管插管中的运用. 上海针灸杂志 24:16-17.

[337] Zhu Hong-sheng. (2005)Application of Electroacupuncture to Anesthesia Induction and Endotracheal Intubation, SHANGHAI JOURNAL OF ACUPUNCTURE AND MOXIBUSTION 24:16-17.

[339] 王天俊,黄玲,邱建英,胡美怡,力斌. (2005)针刺单双侧取穴辅助米非司酮终止早孕疗效比较. 上海针灸杂志 24:6-7.

[339] Wang Tian-jun, Huang-Ling, Qiu Jiany-ing, Humei-Yi, Li-Bin,(2005)Influences of Unilateral and Bilateral Point Selections on Acupuncture-assisted Termination of Early Pregnancy with Mifepristone, SHANGHAI JOURNAL OF ACUPUNCTURE AND MOXIBUSTION 24:6-7.

[340] 黄俏蓉,陈跃来. (2005)电针治疗不稳定膀胱的临床观察. 上海针灸杂志 24:8-9.

[340]Huang Qiao-rong, Chen Yue-lai. (2005)Observations on Electroacupuncture Treatment of Unstable Bladder, SHANGHAI JOURNAL OF ACUPUNCTURE AND MOXIBUSTION 24:8-9.

[341] 陶琪彬,卢翠飞. (2005)针刺治疗急性寰枢关节半脱位疗效观察. 上海针灸杂志 24:14-15.

[341] Tao Qi-bin, Lu Cui-fei. (2005)Observations on the Curative Effect of Acupuncture on patients with Acute Atlantoaxial Subluxation, SHANGHAI JOURNAL OF ACUPUNCTURE AND MOXIBUSTION 24:14-15.

[342] 赵利华. (2005)运动针法治疗急性腰扭伤并腰椎间盘突出的临床观察. 上海针灸杂志 24:17-18.

[342]Zhao Li-hua. (2005) clinical observation on treatment of acute lumbar sprain lumbar disc herniation by kinetic needling.SHANGHAI JOURNAL OF ACUPUNCTURE AND MOXIBUSTION 24:17-18.

[343] 朱玲丽,陆黎. (2005)电针对老年患者术中血流动力学的影响. 上海针灸杂志 24:13-14.

[343] Zhu Ling-li, LU-Li. (2005)Influence of Electroacupuncture on Intraoperative Hemodynamics in Old Patients, SHANGHAI JOURNAL OF ACUPUNCTURE AND MOXIBUSTION 24:13-14.

[344] 沈克艰,吴梅珍. (2005)伏天铺灸治疗慢性盆腔炎的临床研究. 上海针灸杂志 24:19-20.

[344]Shen Ke-jian , Wu Mei-zhen. (2005)Clinical Study on Treatment of Chronic Pelvic Inflammation by Long Snake Moxibustion in Dog Days, SHANGHAI JOURNAL OF ACUPUNCTURE AND MOXIBUSTION 24:19-20.

[345] 朱忠春,穆敬平,梁艳,宗蕾,胡军. (2005)电针治疗海洛因依赖者戒断后睡眠障碍的临床观察. 上海针灸杂志 24:6-8.

[345]Zhu Zhong-chun, Mu Jing-Ping, Liang-Yan ,Zong-Lei, Hu-Jun. (2005)Clinical Observation on Treatment of Dyssomnia of Heroin Addicts after Detoxification by Electro-acupuncture, SHANGHAI JOURNAL OF ACUPUNCTURE AND MOXIBUSTION 24:6-8.

[346] 毛开颜,郭转. (2005)针药结合治疗脑梗死继发癫痫的疗效观察. 上海针灸杂志 24:17-18.

[346] Mao Kai-yan, Gu-Zhuan. (2005)Observations on the Efficacy of Combined Acupuncture and Medicine for Treating Epilepsy Secondary to Cerebral Infarction, SHANGHAI JOURNAL OF ACUPUNCTURE AND MOXIBUSTION 24:17-18.

[347] 孙远征,刘婷婷. (2005)针刺、穴位注射和梅花针叩刺治疗糖尿病周围神经病变的疗效观察. 上海针灸杂志 24:3-5.

[347] Sun Yuan-zheng, Liu Ting-ting. (2005)Observations on the Efficacy of Acupuncture, Point Injection and Plum-blossom Needle Tapping for Treating Diabetic Peripheral Neuropathy, SHANGHAI JOURNAL OF ACUPUNCTURE AND MOXIBUSTION 24:3-5.

[348] 朱洁. (2005)电针结合温灸治疗肛肠病术后尿潴留疗效观察. 上海针灸杂志 24:17-18.

[348] Zhu-Jie. (2005)Observations on the Efficacy of Electroacupuncture plus Mild Moxibustion for Treating Anorectal Postoperative Uroschesis, SHANGHAI JOURNAL OF ACUPUNCTURE AND MOXIBUSTION 24:17-18.

[349] 刘清国,高贺瑜. (2005)梅花磁针综合疗法治疗前列腺增生症149例. 上海针灸杂志 24:35-37.

[349]Liu Qing-guo, Gao He-yu. (2005)Treatment of 149 Cases of Prostate Hyperplasia by Magnetic Plum-blossom Needle Composite Treatment, SHANGHAI JOURNAL OF ACUPUNCTURE AND MOXIBUSTION 24:35-37.

[350] 曾伶. (2005)透刺疗法治疗颈椎病30例. 上海针灸杂志 24:9-10.

[350] Zeng-Lin. (2005)Treatment of Cervical Spondylopathy by Point-through-point Acupuncture, SHANGHAI JOURNAL OF ACUPUNCTURE AND MOXIBUSTION 24:9-10.

[351] 李鹤,沈惠风. (2005)胃肠下合穴治疗手术后胃瘫综合征的临床研究. 上海针灸杂志 24:14-16.

[351] Li-He, Shen Hui-feng. (2005)Clinical Study on the Treatment of Postsurgical Gastroparesis Syndrome by Gastrointestinal Lower-Sea Point, SHANGHAI JOURNAL OF ACUPUNCTURE AND MOXIBUSTION 24:14-16.

[352] 宋春华,东贵荣,杨素清. (2005)曲池穴针刺治疗慢性荨麻疹的临床观察. 上海针灸杂志 24:17-18.

[352] Song Chun-hua, Dong Gui-rong, Yang Su-qing, (2005)Clinical Observations on the Treatment of 96 Chronic Urticaria Patients by Point Quchi Acupunctur, SHANGHAI JOURNAL OF ACUPUNCTURE AND MOXIBUSTION 24:17-18.

[353] 项琼瑶,傅莉萍. (2005)平衡阴阳针刺法治疗周围性面瘫后期的临床观察. 上海针灸杂志 24:32-34.

[353] Xiang Qiong-yao, Fu Li-ping. (2005)Clinical Observations on the Treatment of Late Peripheral Facial Paralysis with Yin-yang Balancing Acupuncture, SHANGHAI JOURNAL OF ACUPUNCTURE AND MOXIBUSTION 24:32-34.

[354] 杨鹏飞. (2005)电针加TDP照射治疗非感染性尿道综合征疗效观察. 上海针灸杂志 24:23-24.

[354] Yang Peng-fei. (2005)Observations on the Efficacy of Electroacupuncture plus TDP Irradiation for Treating Noninfectious Urethral Syndrome, SHANGHAI JOURNAL OF ACUPUNCTURE AND MOXIBUSTION 24:23-24.

[355] 冯国湘,刘未艾,曾碧枚, 吴清明,易宣超,等. (2005)祛风化痰针法对风痰型假性球麻痹患者TCD的影响. 上海针灸杂志 24:8-10.

[355] Feng Guo-xiang, Liu Ai-wei, Zeng Bi-mei, Wu Qing-ming, Yi Chao-xuan. (2005)The Influence of Wind and Phlegm-removing Acupuncture on TCD in Pseudobulbar Paralysis Patients of Wind-phlegm Type , SHANGHAI JOURNAL OF ACUPUNCTURE AND MOXIBUSTION 24:8-10.

[356] 张全明,靳瑞. (2005)针刺治疗智力迟缓儿童语言障碍80例临床研究. 上海针灸杂志 24:20-22.

[356] Zhang Quan-ming, Rui-Jin. (2005)Clinical Study on Acupuncture Treatment of Dysphasia in 80 Hypophrenia Children, SHANGHAI JOURNAL OF ACUPUNCTURE AND MOXIBUSTION 24:20-22.

[357] 鲍超. (2005)健脑调神针法治疗中风后抑郁症62例临床观察. 上海针灸杂志 24:9-10.

[357]Bao-Chao. (2005)Clinical Observations on the Treatment of 62 Post-apoplectic Melancholia Patients by Brain-reinforcing and Mind-regulating Acupuncture, SHANGHAI JOURNAL OF ACUPUNCTURE AND MOXIBUSTION 24:9-10.

[358] 唐燕萍. (2005)多针浅刺与行气法治疗面肌痉挛37例. 上海针灸杂志 24:17-18.

[358] Tang Yan-ping. (2005)Treatment of 37 Facial Spasm Patients by Several-Needle Shallow Puncture and Circulating Qi, SHANGHAI JOURNAL OF ACUPUNCTURE AND MOXIBUSTION 24:17-18.

[359] 庄子齐. (2005)电针郄穴为主对血瘀型颈椎病疗效及血液流变学的影响. 上海针灸杂志 24:3-5.

[359] Zhang Zi-qi. (2005)The Curative Effect of Xi-Cleft Point Acupuncture on Cervical Spondylopathy of Blood Stasis Type and its Influence on Hemorheology , SHANGHAI JOURNAL OF ACUPUNCTURE AND MOXIBUSTION 24:3-5.

[360] 杨国荣. (2005)头体针结合治疗假性延髓麻痹的临床观察. 上海针灸杂志 24:10-11.

[360] Yang Guo-rong. (2005)Clinical Observations on the Treatment of Pseudobulbar Paralysis by Combined Scalp and Body Acupuncture, SHANGHAI JOURNAL OF ACUPUNCTURE AND MOXIBUSTION 24:10-11.

[361] 王少贞. (2005)针药并用治疗假性延髓麻痹50例疗效观察. 上海针灸杂志 24:12-13.

[361]Wang Shao-zhen. (2005)Observations on the Efficacy of Combined Acupuncture and Medicine for Treating 50 Pseudobulbar Paralysis Patients, SHANGHAI JOURNAL OF ACUPUNCTURE AND MOXIBUSTION 24:12-13.

[362] 殷之放,汪司右. (2005)针刺加穴位贴敷治疗难治性高血压病临床初探. 上海针灸杂志 24:16-17.

[362] Yin Zhi-fang,Wang Si-you. (2005)Preliminary Clinical Study on the Treatment of Refractory Hypertension by Acupuncture plus Point Application, SHANGHAI JOURNAL OF ACUPUNCTURE AND MOXIBUSTION 24:16-17.

[363] 程玲,张春燕,甘志豪. (2005)针刺加拔罐治疗慢性单纯性咽炎30例临床观察. 上海针灸杂志 24:18-19.

[363] Cheng-Ling, Zhang Chuny-an, Gan Zhi-hao. (2005)Clinical Observations on the Treatment of 30 Chronic Simple Pharyngitis Patients by Acupuncture plus Cupping,SHANGHAI JOURNAL OF ACUPUNCTURE AND MOXIBUSTION 24:18-19.

[364] 吴耀持,汪崇淼,张必萌. (2005)电针后溪穴治疗急性腰扭伤的临床观察. 上海针灸杂志 24:22-24.

[364] Wu Yao-chi,Wang Chong-miao. (2005)Clinical Observations on the Treatment of Acute Lumbar Sprain by Electroacupuncture of Point Houxi , SHANGHAI JOURNAL OF ACUPUNCTURE AND MOXIBUSTION 24:22-24.

[365] 陈梅,徐斌,艾炳蔚, 李玉堂. (2005)电捻针配合耳压治疗单纯性肥胖病58例临床观察. 上海针灸杂志 24:8-9.

[365] Mei-Chen, Xu-Bin, Yi Bing-wei, Li Yu-tang. (2005)Clinical Observations on the Treatment of 58 Simple Obesity Patients by Electric and Twirling Acupuncture plus Auricular-plaster Therapy, SHANGHAI JOURNAL OF ACUPUNCTURE AND MOXIBUSTION 24:8-9.

[366] 车涛,裘敏雷,忻志平,邵萍. (2006)电针肩髃穴治疗肩关节周围炎疗效观察. 上海针灸杂志 25:21-22.

[366] Chao-Tao, Qiu Min-lei, Xin Zhi-ping, Shao-ping. (2006)Clinical study on the treatment of shoulder periarthritis by point Jianyu electroacupuncture, SHANGHAI JOURNAL OF ACUPUNCTURE AND MOXIBUSTION 25:21-22.

[367] 赵宏,刘志顺,赵婷,赵因,王麟鹏.(2006)电针调理髓海法治疗前庭中枢性平衡障碍疗效观察. 上海针灸杂志 25:7-10.

[367] Zhao-Hong, Liu Zhi-shun, Zhao-Ting, Zhao-Yin, Wang Lin-peng. (2006)Clinical study on the treatment of shoulder periarthritis by point Jianyu electroacupuncture, SHANGHAI JOURNAL OF ACUPUNCTURE AND MOXIBUSTION 25:7-10

[368] 李国萍,李宏军,李宏岩. (2006)耳穴针刺及放血治疗急性结膜炎20例. 上海针灸杂志 25:23.

[368] Li Guo-ping, Li Hong-jun, Li-Yan. (2006)Treatment of 20 cases acute keratitis patients byAuricular acupuncture and bloodletting. SHANGHAI JOURNAL OF ACUPUNCTURE AND MOXIBUSTION 25:23.

[369] 贾红玲. (2006)项七针与颈夹脊穴对椎动脉型颈椎病椎-基底动脉血流动力学影响的临床观察. 上海针灸杂志 25:5-7.

[369] Jia Hong-lin. (2006)Clinical observations on the influences of nape seven needles and cervical jiaji points on vertebrobasilar hemodynamics in cervical spondylopathy of vertebral artery type,SHANGHAI JOURNAL OF ACUPUNCTURE AND MOXIBUSTION 25:5-7.

[370] 杨淑贤,王秀芹,蔡永. (2006)温针灸配合神阙穴贴敷治疗肠易激综合征疗效观察. 上海针灸杂志 25:15-16.

[370] Yang Shu-xian, Wang Xiu-qin, Cai-Yong. (2006)Observations on the Efficacy of Warming Acupuncture plus Point Shenque Application for Treating Irritable Bowel Syndrome, SHANGHAI JOURNAL OF ACUPUNCTURE AND MOXIBUSTION 25:15-16

[371] 吴笛. (2006)穴位埋线治疗顽固性呃逆32例临床观察. 上海针灸杂志 25:17-18.

[371]Wu-Di. (2006)Clinical Observations on Treatment of 32 Intractable Hiccup Patients by Point Catgut Embedding, SHANGHAI JOURNAL OF ACUPUNCTURE AND MOXIBUSTION 25:17-18.

[372] 王胜,孙忠人,李春玲. (2006)电针治疗腰椎间盘突出症的疗效对比观察. 上海针灸杂志 25:21-23.

[372] Wang-Sheng, Sun Zhong-ren, Li Chun-ling. (2006)Comparative Observations on the Curative Effect of Electroacupuncture on Lumbar Intervertebral Disc Protrusion , SHANGHAI JOURNAL OF ACUPUNCTURE AND MOXIBUSTION 25:21-23.

[373] 曾小香,梁进娟. (2006)输穴刺络拔罐治疗急性踝关节扭伤临床观察. 上海针灸杂志 25:25-26.

[373]Zeng Xiao-xiang, Liang JinJuan. (2006)Clinical observations on the treatment of acute ankle sprain by blood-letting puncture and cupping.SHANGHAI JOURNAL OF ACUPUNCTURE AND MOXIBUSTION. 25:25-26.

[374] 周鸿飞,王恩龙,徐锦平,曹铁军. (2006)项丛刺对急性脑梗死患者外周血浆ET含量的影响. 上海针灸杂志 25:11-13.

[374]Zhou Hong-fei, Wang En-long, Xu Jing-ping, CAO Tie-jun. (2006)Influence of Nape Cluster Acupuncture on Peripheral Serum ET Content in Patients with Acute Cerebral Infarction. SHANGHAI JOURNAL OF ACUPUNCTURE AND MOXIBUSTION. 25:11-13.

[375] 李阳. (2006)针刺对急性脑梗死后血浆NO及NOS含量的影响. 上海针灸杂志 25:16-17.

[375]Li Yang, (2006)Influence of Acupuncture on Plasma NO and NOS Contents After Acute Cerebral Infarction. SHANGHAI JOURNAL OF ACUPUNCTURE AND MOXIBUSTION. 25:16-17.

[376] 史晓岚,李伟红. (2006)辨证选穴针刺配中药内服治疗更年期综合征38例. 上海针灸杂志 25:6-7.

[376]Shi Xiao-lan, Li Wei-hong. (2006)Treatment of 38 Climacteric Syndrome Patients by Acupuncture of Points Selected on the Basis of Syndrome Differentiation plus Oral Administration of Chinese Herbs. SHANGHAI JOURNAL OF ACUPUNCTURE AND MOXIBUSTION. 25:6-7.

[377] 黄勇,刘桂珍. (2006)水针结合体针治疗中风后遗症31例临床观察. 上海针灸杂志 25:13-14.

[377]Huang Yong, Liu Gui-zhe. (2006)Clinical observations on the treatment of 31 apoplectic sequela patients by point injection plus body acupuncture. SHANGHAI JOURNAL OF ACUPUNCTURE AND MOXIBUSTION. 25:13-14.

[378] 高莉萍,邹勇. (2006)安神清脑法针灸治疗焦虑症42例疗效观察. 上海针灸杂志 25:28-29.

[378]Gao Li-ping, Zhou Yong. (2006)Observations on the efficacy of mind-calming and brain-refreshing acupuncture and moxibustion for treating 42 anxiety patients. SHANGHAI JOURNAL OF ACUPUNCTURE AND MOXIBUSTION. 25:28-29.

[379] 姚光潮. (2006)针刺配合叩刺治疗厌食30例.上海针灸杂志 25:30.

[379]Yao Guang-chao. (2006)Acupuncture treatment of 30 cases of tapping anorexia. SHANGHAI JOURNAL OF ACUPUNCTURE AND MOXIBUSTION. 25:30.

[380] 朱文增,倪金霞,鲍春龄,东贵荣,唐强. (2006)头穴透刺结合康复治疗急性脑出血28例临床研究. 上海针灸杂志 25:7-9.

[380]Zhu Wen-zeng, Ni Jin-xia, Bao Chun-lin, Dong Gui-rong, Tang Qiang. (2006)Clinical study on the treatment of 28 acute cerebral hemorrhage patients by scalp point-through-point acupuncture plus rehabilitation therapy. SHANGHAI JOURNAL OF ACUPUNCTURE AND MOXIBUSTION. 25:7-9.

[381] 金孟梓. (2006)穴位注射加牵引治疗神经根型颈椎病疗效观察. 上海针灸杂志 25:13-14.

[381]Jin Men-zi. (2006)Observations on the Efficacy of Point Injection plus Traction for Treating Nerve Root Cervical Spondylopathy. SHANGHAI JOURNAL OF ACUPUNCTURE AND MOXIBUSTION. 25:13-14.

[382] 钟宾谟,潘长青.(2006)腹针加牵引综合治疗腰椎间盘突出症疗效观察. 上海针灸杂志 25:19-20.

[382]Zhong Bin-mo, Pan Chang-qing. (2006)Treatment of Lumbar Intervertebral Disc Protrusion by Abdominal Acupuncture plus Traction. SHANGHAI JOURNAL OF ACUPUNCTURE AND MOXIBUSTION. 25:19-20.

[383] 裘小玲,裘昊,黄涌, 吴圣荣,钱群等. (2006)钩针疗法治疗中风偏瘫后肩痛疗效观察. 上海针灸杂志 25:10-12.

[383]Qiu Xiao-ling, Qiu Hao,Huang Yong, Wu Sheng-rong, Qian Qun et.al. (2006)Observations on the curative effect of hook needle acupuncture on shoulder pain after apoplectic hemiplegia. SHANGHAI JOURNAL OF ACUPUNCTURE AND MOXIBUSTION. 25:10-12.

[384] 魏向阳. (2006)电针治疗中风偏瘫肩手综合征临床观察. 上海针灸杂志 25:13-14.

[384]WeiXiang-yang. (2006)Clinical study on electroacupuncture treatment of apoplectic hemiplegia shoulder-hand syndrome. SHANGHAI JOURNAL OF ACUPUNCTURE AND MOXIBUSTION. 25:13-14.

[385] 金孟梓.(2006)刺络拔罐加艾条灸治疗股外侧皮神经炎疗效观察. 上海针灸杂志 25:26-27.

[385]Jin Meng-zhi. (2006)Observations on the efficacy of blood-letting puncture and cupping plus moxa-stick moxibustion for treating inflammatio of lateral femoral cutaneous nerve. SHANGHAI JOURNAL OF ACUPUNCTURE AND MOXIBUSTION. 25:26-27.

[386] 李雁,庞勇,邹卓成. (2006)益肾调督针法治疗缺血性中风临床研究. 上海针灸杂志 25:7-9.

[386]Li Yan, Pang Yong, Zhou Zhuo-cheng. (2006)Clinical study on treatment of ischemic stroke by kidney-reinforcing and Du channel-regulating acupuncture method. SHANGHAI JOURNAL OF ACUPUNCTURE AND MOXIBUSTION. 25:7-9.

[387] 孙培华. (2006)针刺配合耳压治疗偏头痛临床观察. 上海针灸杂志 25:12-13.

[387]Sun Pei-hua. (2006)Clinical Observations on the Treatment of Migraine by Acupuncture plus Auricular-plaster Therapy. SHANGHAI JOURNAL OF ACUPUNCTURE AND MOXIBUSTION. 25:12-13.

[388] 郑晓,李岩峰,忻志平,徐培莉.(2006)电针治疗椎动脉型颈椎病的临床观察. 上海针灸杂志 25:18-19.

[388]Zheng Xiao, Li Yan-feng, Xin Zhi-ping, Xu Pei-li. (2006)Clinical Observations on Electroacupuncture Treatment of Vertebral Artery Cervical Spondylopathy. SHANGHAI JOURNAL OF ACUPUNCTURE AND MOXIBUSTIO. 25:18-19.

[389] 刘明. (2006)针刺拔罐配合TDP治疗膝关节疼痛的临床观察. 上海针灸杂志 25:20-21.

[389]LIU Ming. (2006)Clinical Observations on Treatment of Genual Arthralgia by Acupuncture and Cupping plus TDP. SHANGHAI JOURNAL OF ACUPUNCTURE AND MOXIBUSTION. 25:20-21.

[390] 连方,李海仙,张建伟,张宁,孙振高,等. (2006)电针促使LUFS病人排卵的临床研究. 上海针灸杂志 25:3-5.

[390]Lian Fang, LI Hai-xian, Zhang Jian-wei, Zhang Ning, Sun Zheng-gao et.al. (2006)Clinical Observations on Electroacupuncture Promotion of Ovulation in LUFS Patients. SHANGHAI JOURNAL OF ACUPUNCTURE AND MOXIBUSTION. 25:3-5.

[391] 王兵,张翠英,张军岐,苏一焕,倪承皓,等. (2006)针刺合谷穴治疗牙痛的临床研究. 上海针灸杂志 25:6-9.

[391]Wang Bing, Zhang Cui-ying, Zhang Jun-qi, Su Yi-huan, Ni Cheng-hao et.al. (2006)Clinical Study on Treatment of Odontalgia by Acupunctrue of Point Hegu. SHANGHAI JOURNAL OF ACUPUNCTURE AND MOXIBUSTION. 25:6-9.

[392] 朱首豪,林敉祥,王少贞.(2006)针刺治疗急性脑梗死临床观察.上海针灸杂志 25:11-12.

[392]Zhu Shou-hao, Lin Mixing,Wang, Shao-zhen. (2006)Clinical observations on acupuncture treatment of acute cerebral infarction. SHANGHAI JOURNAL OF ACUPUNCTURE AND MOXIBUSTION. 25:11-12.

[393] 李涛,田桂杰,张蓝,魏国威,马倩,等.(2006)针刺对大面积脑梗塞术后病人神经功能的影响. 上海针灸杂志 25:3-5.

[393]Li Tao, Tian Gui-jie, Zhang Lan, Wei Guo-wei,Ma Qian et.al. (2006)Clinical study on the effect of acupuncture on the neural function in extensive cerebral infarction-postoperative patients. SHANGHAI JOURNAL OF ACUPUNCTURE AND MOXIBUSTION. 25:3-5.

[394] 聂斌,聂涛. (2006)电针治疗中风后抑郁症的临床研究. 上海针灸杂志 25:6-8.

[394]Nie Bin, Nie Tao. (2006)Clinical study on electroacupuncture treatment of postapoplectic depression. SHANGHAI JOURNAL OF ACUPUNCTURE AND MOXIBUSTION. 25:6-8.

[395] 郭长青,芮娜,刘焰刚,刘云霞,沙岩,等. (2007)新砭石疗法治疗肩关节周围炎的临床疗效研究与评价. 上海针灸杂志 26:10-12.

[395]Guo Chang-qing, RUI Na, Liu Yan-gang, Liu Yun-xia, Sha Yan et.al. (2007)Study and evaluation of the clinical efficacy of new stone needle therapy for shoulder periarthritis. SHANGHAI JOURNAL OF ACUPUNCTURE AND MOXIBUSTION. 26:10-12.

[396] 郭言川. (2007)沿皮透刺治疗带状疱疹后神经痛的临床观察. 上海针灸杂志 26:18-19.

[396]Guo Yan-chuan. (2007)Clinical observations on treatment of post-herpes zoster neuralgia by along-skin point-through-point acupuncture. SHANGHAI JOURNAL OF ACUPUNCTURE AND MOXIBUSTION. 26:18-19.

[397] 赵小强,刘登娥,林成宏. (2007)体针与腹针疗法减肥疗效观察. 上海针灸杂志 26:28.

[397]Zhao Xiao-qiang, Liu Deng-e, Lin Cheng-hong. (2007)Observation of acupuncture and abdominal acupuncture slimming effect. SHANGHAI JOURNAL OF ACUPUNCTURE AND MOXIBUSTIO. 26:28.

[398] 于成山,陶鸿飞.(2007)穴位注射治疗化疗后白细胞减少的临床观察. 上海针灸杂志 26:11-12.

[398]Yu Cheng-shan, Tao Hong-fei. (2007)Clinical Observations on Treatment of Postchemotherapeutic Leukopenia by Point Injection. SHANGHAI JOURNAL OF ACUPUNCTURE AND MOXIBUSTION. 26:11-12.

[399] 刘志顺,杜仪. (2007)电针治疗中风后尿失禁的疗效评价. 上海针灸杂志 26:13-14.

[399]Liu Zhi-shun, Du Yi. (2007)Evaluation of the Curative Effect of Electroacupuncture on Postapoplectic Incontinence of Urine. SHANGHAI JOURNAL OF ACUPUNCTURE AND MOXIBUSTION. 26:13-14.

[400] 李卫东.(2007)神经傍刺治疗枕神经痛疗效观察. 上海针灸杂志 26:15-16.

[400]Li Wei-dong. (2007)Observations on the Efficacy of Nerve-proximal Needling for Treating Occipital Neuralgia. SHANGHAI JOURNAL OF ACUPUNCTURE AND MOXIBUSTION. 26:15-16.

[401] 牛文民,刘海洋. (2007)头针治疗血管性痴呆的临床研究. 上海针灸杂志 26:4-5.

[401]Niu Wen-min, Liu Haiyang. (2007)Clinical Study of Treatment of Vascular Dementia with Lasting Scalp Acupunture. SHANGHAI JOURNAL OF ACUPUNCTURE AND MOXIBUSTION. 26:4-5.

[402] 李智. (2007)石氏中风单元疗法”治疗急性期脑出血临床观察. 上海针灸杂志 26:6-8.

[402]Li Zhi. (2007)Clinical Observations on Treatment of Acute-stage Cerebral Hemorrhage Patients by "Shi's Stroke Unit Therapy". SHANGHAI JOURNAL OF ACUPUNCTURE AND MOXIBUSTION. 26:6-8.

[403] 马学青,姜淑洁. (2007)背俞挑刺闪罐治疗黄褐斑疗效分析. 上海针灸杂 26:20-21.

[403]Ma Xue-qing, Jiang Shu-jie. (2007)Analysis of the Efficacy of Back-Shu Point Pricking Therapy and Quick Cupping for Chloasma. SHANGHAI JOURNAL OF ACUPUNCTURE AND MOXIBUSTION. 26:20-21.

[404] 刘娜,邓玉霞. (2007)苍龟探穴治疗梨状肌综合征疗效观察. 上海针灸杂志 26:25-26.

[404]Liu Na, Deng Yu-xia. (2007)Observations on Treatment of Piriformis Syndrome by a Technique of Green Tortoise Exploring Point. SHANGHAI JOURNAL OF ACUPUNCTURE AND MOXIBUSTION. 26:25-26.

[405] 黄涛,陈宝英,何军琴, 白剑,古梅,等.(2007)针刺膻中穴治疗产后缺乳的临床疗效研究.

[405]Huang Tao, Chen Bao-ying, He Jun-qin, Bai Jian, Gu Mei et.al. (2007)Study of the Clinical Efficacy of Point Shanzhong Acupuncture for Treating Postpartum Hypogalactia. SHANGHAI JOURNAL OF ACUPUNCTURE AND MOXIBUSTION. 26:3-5.

[406] 吴际生,徐雀莺. (2007)针刺配合山冬瓜根治疗腰椎间盘突出症疗效观察. 上海针灸杂志 26:28.

[406]Wu Ji-sheng, Xu Que-ying. (2007)Observation of disc herniation in Dongguashan root treatment of lumbar with acupuncture. SHANGHAI JOURNAL OF ACUPUNCTURE AND MOXIBUSTION. 26:28.

[407] 黄思琴,李常度.(2007)新肩三针穴位注射为主治疗肩手综合征的临床研究. 上海针灸杂志 26):9-10.

[407]Huang Si-qin, Li Chang-du. (2007)Clinical Study of New-shoulder-three-needle Point Injection for Treating Postapoplectic Shoulder-hand Syndrome. SHANGHAI JOURNAL OF ACUPUNCTURE AND MOXIBUSTION. 26):9-10.

[408] 孙远征,王琳晶. (2007)针刺配合脑超声治疗仪对脑出血恢复期的疗效观察. 上海针灸杂志 26:17-18.

[408]Sun Yuan-zheng, Wang Lin-jing. (2007)Observations on the Efficacy of Acupuncture plus Transcranial Ultrasound Therapeutic Instrument for Treating Cerebral Hemorrhage of Convalescent Stage. SHANGHAI JOURNAL OF ACUPUNCTURE AND MOXIBUSTION. 26:17-18.

[409] 刘红,杨大男. (2007)穴位埋线治疗围绝经期综合征86例临床观察. 上海针灸杂志 26):5-7.

[409]Liu Hong,Yang Da-nan. (2007)Clinical Observations on Treatment of 86 Peri-climacteric Syndrome Cases by Point Catgut Embedding. SHANGHAI JOURNAL OF ACUPUNCTURE AND MOXIBUSTION. 26):5-7.

[410] 陈夏燕.(2007)深刺夹脊穴治疗腰椎间盘突出症的疗效观察. 上海针灸杂志 26:21-22.

[410]Chen Xia-yan. (2007)Observations on the Efficacy of Deep Acupuncture of Jiaji Points for Treating Lumbar Intervertebral Disc Protrusion. SHANGHAI JOURNAL OF ACUPUNCTURE AND MOXIBUSTION. 26:21-22.

[411] 吴洲红,周志华.(2007)浅刺多针法治疗单纯性肥胖症的疗效观察. 上海针灸杂志 26:7-9.

[411]Wu Zhou-hong, Zhou Zhi-hua. (2007)Observations on the Curative Effect of Multi-needle Shallow Puncture on Simple Obesity. SHANGHAI JOURNAL OF ACUPUNCTURE AND MOXIBUSTION. 26:7-9.

[412] 吴壮,高维滨. (2007)电针治疗脑梗死后痉挛性瘫的临床观察. 上海针灸杂志 26:10-11.

[412]Wu Zhuang, Gao Wei-bin. (2007)Clinical Study on Electroacupuncture Treatment of Spastic Paralysis Following Cerebral Infarction. SHANGHAI JOURNAL OF ACUPUNCTURE AND MOXIBUSTION. 26:10-11.

[413] 吴焕淦,施征,朱毅,马晓芃,姚怡,等.(2007)隔药灸治疗溃疡性结肠炎的临床研究. 上海针灸杂志 26:3-4.

[413]Wu Huan-jin, Shi Zheng, Zhu Yi, Ma Xiao-fan, Yao Yi et.al. (2007)Clinical Study on Treatment of Ulcerative Colitis by Medicinal Cake-separated Moxibustion. SHANGHAI JOURNAL OF ACUPUNCTURE AND MOXIBUSTION. 26:3-4.

[414] 宋玉娟,张力,周育瑾.(2007)电针辅助治疗对重型脑外伤昏迷病人促醒的疗效观察. 上海针灸杂志 26:11-12.

[414]Song Yu-juan, Zhang Li, Zhou Yu-qing. (2007)Observations on the Efficacy of Electroacupuncture-assisted Treatment for Resuscitating Coma Patients with Serious Brain Trauma. SHANGHAI JOURNAL OF ACUPUNCTURE AND MOXIBUSTION. 26:11-12.

[415] 魏红沁,丁春华,李艳芬,郁惠峰. (2007)针刺对混合痔剥扎术后镇痛的临床观察. 上海针灸杂志 26:32.

[415]Wei Hong-qin, Ding Chun-hua, Li Yan-fen, Yu Hui-feng. (2007)Clinical observation on acupuncture analgesia after ligation and stripping of mixed hemorrhoids. SHANGHAI JOURNAL OF ACUPUNCTURE AND MOXIBUSTION. 26:32.

[416]孙奎,杨永晖,罗建明,刘德春. (2007)针刺配合推拿治疗膝骨关节炎的临床观察. 上海针灸杂志 26(6):11-12.

[416]Sun Kui, Yang Yong-hui, Luo Jian-ming, Liu De-chun. (2007)Clinical Observations On Treatment of Genual Osteoarthritis by Acupuncture plus Massotherapy. SHANGHAI JOURNAL OF ACUPUNCTURE AND MOXIBUSTION. 26(6):11-12.

[417] 陈虹,朱洪生. (2007)电针应用于无痛胃镜检查的临床观察. 上海针灸杂志 26:19-20.

[417]Chen Hong, Zhu Hong-sheng. (2007)Clinical Observations on Application of Electroacupuncture to Painless Gastroscopy. SHANGHAI JOURNAL OF ACUPUNCTURE AND MOXIBUSTION. 26:19-20.

[418] 曾红文,聂斌,黄年斌.(2007)刺络拔罐配合温针治疗椎动脉型颈椎病的疗效分析. 上海针灸杂志 26:8-10.

[418]Zeng Hong-wen, Nie Bin, Huang Nian-bin. (2007)Analysis of the Efficacy of Blood-letting Puncture and Cupping plus Warming Acupuncture for Treating Cervical Spondylopathy of Vertebral Artery Type. SHANGHAI JOURNAL OF ACUPUNCTURE AND MOXIBUSTION. 26:8-10.

[419] 刘明清,刘俐,黄启嵩.(2007)隔姜灸加穴位注射治疗支气管哮喘临床观察. 上海针灸杂志 26:8-9.

[419]Liu Ming-qing, Liu Li, Huang Qi-song. (2007)Clinical Observations on Treatment of Bronchial Asthma by Ginger-separated Moxibustion plus Point Injection. SHANGHAI JOURNAL OF ACUPUNCTURE AND MOXIBUSTION. 26:8-9.

[420] 王延红,应盛国.(2007)项丛刺治疗椎动脉型颈椎病疗效观察. 上海针灸杂志 26:11-12.

[420]Wang Yan-hong,Ying Sheng-guo. (2007)Observations on the Curative Effect of Nape-aligned Acupuncture on Vertebral Artery-type Cervical Spondylopathy. SHANGHAI JOURNAL OF ACUPUNCTURE AND MOXIBUSTION. 26:11-12.

[421] 王希琳,黄海燕,蒋林剑. (2007)埋线疗法治疗肠易激综合征的临床观察. 上海针灸杂志 26:17-18.

[421]Wang Xi-lin, Huang Hai-yan, Jiang Lin-jian. (2007)Observation on Treatment of Irritable Bowel Syndrome by Catgut Embedding. SHANGHAI JOURNAL OF ACUPUNCTURE AND MOXIBUSTION. 26:17-18.

[422] 周密,王震虹,王祥瑞,杨华元,朱训生. (2007)急性疼痛及电针镇痛评估系统的临床研究. 上海针灸杂志 26:21-24.

[422]Zhou Mi, Wang Zheng-hong, Wang Xiang-rui, Yang Hua-yuan, Zhu Xun-sheng. (2007)Clinical Study of Acute Pain and Electroacupuncture Analgesia Evaluating System. SHANGHAI JOURNAL OF ACUPUNCTURE AND MOXIBUSTION. 26:21-24.

[423] 林强,胡玉莲,韩崇伟, 厉岩. (2007)眼针治疗由输尿管结石引起的肾、输尿管绞痛90例疗效观察. 上海针灸杂志 26:12-13.

[423]Lin Qiang,Hu Yu-lian, Han Chong-wei,Li Yan. (2007)Clinical Study of Eye Acupuncture Treatment of 90 Renal and Ureteral Colic due to Ureteral Calculus. SHANGHAI JOURNAL OF ACUPUNCTURE AND MOXIBUSTION. 26:12-13.

[424] 闫继红. (2007)针刺联合弥可保治疗糖尿病周围神经病变46例疗效观察. 上海针灸杂志 26:14-15.

[424]Yan Ji-hong. (2007)Treatment of 46 Diabetic Peripheral Neuropathy with Acupuncture plus Methycobal. SHANGHAI JOURNAL OF ACUPUNCTURE AND MOXIBUSTION. 26:14-15.

[425] 曾燕芬.(2007)腹针治疗腰椎间盘突出症67例临床观察. 上海针灸杂志 26:16-17.

[425]Zeng Yan-fen. (2007)Clinical Observations on the Treatment of 67 Lumbar Intervertebral Disc Protrusion by Abdomen Acupuncture. SHANGHAI JOURNAL OF ACUPUNCTURE AND MOXIBUSTION. 26:16-17.

[426] 吉健友,吉健礼. (2007)斜刺配合推拿治疗创伤性跟腱周围炎35例疗效观察. 上海针灸杂志, 2007,26:18-19.

[426]Jie Jian-you, Jie Jian-li. (2007)Observations on the Efficacy of Oblique Acupuncture plus Massotherapy for Treating Traumatic Peritenonitis of Achilles tendon in 35 Cases. SHANGHAI JOURNAL OF ACUPUNCTURE AND MOXIBUSTION. 26:18-19.

[427] 严兴科,杨波,高洋,陈谦 . (2007)穴位埋线治疗肌张力低下型脑瘫37例. 上海针灸杂志 26:20-21.

[427]Yan Xing-ke,Yang Bo,Gao Yang,Chen Qian. (2007)Treatment of 77 Atonic Cerebral Palsy Children by Point Catgut Embedding.SHANGHAI JOURNAL OF ACUPUNCTURE AND MOXIBUSTION. 26:20-21.

[428] 刘敏勇,聂容荣,刑志忠,区鹤龄. (2007)针刺合中药离子热导入治疗跟骨骨刺120例疗效观察. 上海针灸杂志 26:24-25.

[428]Liu Mei-yong, Nie Rong-rong, Xing Zhi-zhong, Qu He-ling. (2007)Clinical observation on 120 cases of ion heat into heat treatment calcaneum spur acupuncture combined with traditional Chinese medicine. SHANGHAI JOURNAL OF ACUPUNCTURE AND MOXIBUSTION. 26:24-25.

[429] 刘坚,徐斯伟,张仁. (2007)透刺为主治疗视疲劳的临床观察. 上海针灸杂志 26:9-11.

[429]Liu Jian, Xu Si-wei, Zhang Ren. (2007)Clinical Observations on Treatment of Ocular Fatigue by Point-through-point Acupuncture. SHANGHAI JOURNAL OF ACUPUNCTURE AND MOXIBUSTION. 26:9-11

[430] 黄传萍. (2008)透穴为主配合针刀松解治疗偏头痛疗效观察. 上海针灸杂志 27:13-14.

[430]Huan Zhuan-ping. (2008)Observations on the Efficacy of Point-through-point Acupuncture plus Needle Knife Lysis in Treating Migraine. SHANGHAI JOURNAL OF ACUPUNCTURE AND MOXIBUSTION. 27:13-14.

[431] 周子信,王仙梅. (2008)电针加星状神经节阻滞治疗面肌痉挛疗效观察. 上海针灸杂志 27:17-18.

[431]Zhou Zi-xin, Wang Xian-mei. (2008)Treatment of Facial Spasm Patients by Electroacupuncture plus Stellate Ganglion Block. SHANGHAI JOURNAL OF ACUPUNCTURE AND MOXIBUSTION. 27:17-18.

[432] 赵宏强,吴阳阳,葛诗翠, 王明泉,王奇强,等. (2008)腹针配合体针治疗多发性神经炎疗效观察. 上海针灸杂志 27:21-22.

[432]Zhao Hong-qiang, Wu Yang-yang, Ge Shi-cui, Wang Ming-quan, Wang Qi-qiang et.al. (2008)Observations on the Efficacy of Abdominal Acupuncture plus Body Acupuncture in Treating Multiple Neuritis. SHANGHAI JOURNAL OF ACUPUNCTURE AND MOXIBUSTION. 27:21-22.

[433] 陈敏. (2008)电针配合穴位注射治疗股外侧皮神经炎疗效观察. 上海针灸杂志 27:25-26.

[433]Chen Min. (2008)Treatment of Neuritis of the Lateral Femoral Cutaneous Nerve by Electroacupuncture plus Point Injection. SHANGHAI JOURNAL OF ACUPUNCTURE AND MOXIBUSTION. 27:25-26.

[434] 朱启玉,吴芳华.(2008)梅花针配合体针治疗斑秃疗效观察. 上海针灸杂志 27:27-28.

[434]Zhu Qi-yu, Wu Fang-hua. (2008)Observations on the Efficacy of Plum-blossom Needle plus Body Acupuncture in Treating 43 Alopecia Areata Patients. SHANGHAI JOURNAL OF ACUPUNCTURE AND MOXIBUSTION. 27:27-28.

[435] 卢永屹. (2008)针刺干预中风先兆疗效观察. 上海针灸杂志 27:10-11.

[435]Lu Yong-yi. (2008)Observations on the Intervening Effect of Acupuncture on Prodromal Symptoms of Stroke. SHANGHAI JOURNAL OF ACUPUNCTURE AND MOXIBUSTION. 27:10-11.

[436] 穆敬平,刘莉,程建明,周立志,敖金波. (2008)电针治疗吸毒者脱毒后焦虑抑郁情绪临床观察. 上海针灸杂志 27:1-3.

[436]Mu Jing-ping, Liu Li, Cheng Jian-ming, Zhou Li-zhi, Ao Jin-bo. (2008)Clinical Study of Electroacupuncture Treatment for Post-withdrawal Anxiety and Depression in Drug Addicts. SHANGHAI JOURNAL OF ACUPUNCTURE AND MOXIBUSTION. 27:1-3.

[437] 张永臣. (2008)针刺配合华佗药枕治疗颈椎病疗效观察. 上海针灸杂志 27:20-21.

[437]Zhang Yong-chen(2008). Observations on the Efficacy of Acupuncture pins Huatuo Medicinal Pillow in Treating Cervical Spondylosis. SHANGHAI JOURNAL OF ACUPUNCTURE AND MOXIBUSTION. 27:20-21.

[438] 张志松,孙谊. (2008)定点旋转复位配合芒针治疗腰椎间盘突出症疗效观察. 上海针灸杂志 27:24-25.

[438]Zhang Zhi-song, Sun Yi. (2008)Observations on the Efficacy of Fixed Rotation and Reposition plus Elongate Needles in Treating Lumbar Intervertebral Disc Herniation. SHANGHAI JOURNAL OF ACUPUNCTURE AND MOXIBUSTION. 27:24-25.

[439] 黄志刚. (2008)温针治疗肩关节周围炎临床观察. 上海针灸杂志 27:26-27.

[439]Huang Zhi-gang. (2008)Clinical Observations on Treatment of Shoulder Periarthritis by Warm Needling. SHANGHAI JOURNAL OF ACUPUNCTURE AND MOXIBUSTION. 27:26-27.

[440] 雷教育,夏春芳,付懿.(2008)经肌电刺激治疗脊髓损伤疗效观察. 上海针灸杂志 27:28-29.

[440]Lei Jiao-yu, Xia Chun-fang, Fu Yi. (2008)Observations on the Therapeutic Effect of Percutaneous Electrostimulation on Spinal Cord Injury. SHANGHAI JOURNAL OF ACUPUNCTURE AND MOXIBUSTION. 27:28-29.

[441] 殷之放.(2008)颈三针为主治疗颈椎病疗效观察. 上海针灸杂志 27:50.

[441]Yin Zhi-fang. (2008)Observation of cervical three needles in treating cervical spondylosis. SHANGHAI JOURNAL OF ACUPUNCTURE AND MOXIBUSTION. 27:50.

[442] 付晓红,张巧玲. (2008)贺氏三通法配合穴位注射治疗偏头痛疗效观察. 上海针灸杂志 27:12-13.

[442]Fu Xiao-hong, Zhang Qiao-ling. (2008)Observations on the Efficacy of He's three-unblocking Therapy plug Point Injection in Treating Migraine. SHANGHAI JOURNAL OF ACUPUNCTURE AND MOXIBUSTION. 27:12-13.

[443] 陈华,傅杰英. (2008)温针治疗囊肿结节型痤疮临床研究. 上海针灸杂志 27:16-18.

[443]Chen Hua, Fu Jie-ying. (2008)Clinical Study of Warming Needling Treatment for Cystic Nodular Ache. SHANGHAI JOURNAL OF ACUPUNCTURE AND MOXIBUSTION. 27:16-18.

[444] 楼利芳,陈旻. (2008)针刺配合红外线治疗宫颈糜烂疗效观察. 上海针灸杂志 27:21-22.

[444]Luo li-fang,Chen Min. (2008)Observations on the Therapeutic Effect of Acupuncture plus Infrared Ray on Cervical Erosion. SHANGHAI JOURNAL OF ACUPUNCTURE AND MOXIBUSTION. 27:21-22.

[445] 穆艳云,李忠仁,程洁,鲍超. (2008)针刺对急性脑梗死患者血清IL-6、IL-8影响的观察. 上海针灸杂志 27:3-5.

[445]Mu Yan-yun, Li Zhong-ren, Cheng Jie, Bao Chao et.al. (2008)Observations on the Influence of Acupuncture on Serum IL-6 and IL-8 in Patients with Acute Cerebral Infarction MU. SHANGHAI JOURNAL OF ACUPUNCTURE AND MOXIBUSTION. 27:3-5.

[446] 朱永志,陈伊,张艳艳. (2008)电针神经干治疗脑梗死偏瘫近期脑电变化观察. 上海针灸杂志 27:6-7.

[446]Zhu Yong-xhi, Chen Yi, Zhang Yan-yan. (2008)Observations on an Early Change in Cerebral Electricity in Electroacupuncture of Nerve Stem for Cerebral Infarction-induced

Hemiplegia. SHANGHAI JOURNAL OF ACUPUNCTURE AND MOXIBUSTION. 27:6-7.

[447] 朱海涛. (2008)穴位埋线治疗慢性萎缩性胃炎疗效观察. 上海针灸杂志 27:11-12.

[447]Zhu Hai-tao. (2008)Observations on the Efficacy of Catgut Embedding at Acupoint in Treating Chronic Atrophic Gastritis. SHANGHAI JOURNAL OF ACUPUNCTURE AND MOXIBUSTION. 27:11-12.

[448] 朱博畅,单永华. (2008)不同针刺方法治疗多发性抽动症临床观察. 上海针灸杂志 27:17-18.

[448]Zhu Bo-chang, Shan Yong-hua. (2008)Clinical Observations on Treatment of Tourette's Syndrome by Different Acupuncture Methods. SHANGHAI JOURNAL OF ACUPUNCTURE AND MOXIBUSTION. 27:17-18.

[449] 宫朝霞,张坤. (2008)针刺配合微波治疗下颌关节功能紊乱疗效观察. 上海针灸杂志 27:42.

[449]Gong Chao-xia, Zhang Kun. (2008)Effect of acupuncture combined with microwave in treatment of temporomandibular joint dysfunction treatment. SHANGHAI JOURNAL OF ACUPUNCTURE AND MOXIBUSTION. 27:42.

[450] 贺君. (2008)俞募配穴埋线治疗慢性萎缩性胃炎临床观察. 上海针灸杂志 27:8-10.

[450]He Jun. (2008)Clinical Observations on Treatment of Chronic Atrophic Gastritis by Catgut Embedding at Combined Shu and Mu Points. SHANGHAI JOURNAL OF ACUPUNCTURE AND MOXIBUSTION. 27:8-10.

[451] 高扬. (2008)针刺配合推拿治疗冈上肌肌腱炎疗效观察. 上海针灸杂志 27:20-21.

[451]Gao Yang. (2008)Observations on the Efficacy of Acupuncture plus Massotherapy in Treating Supraspinatus Tendinitis. SHANGHAI JOURNAL OF ACUPUNCTURE AND MOXIBUSTION. 27:20-21.

[452] 程肖芳,姜进平,彭燕玲,陈宇锋,石林青. (2008)电针加穴位注射治疗腰椎间盘突出症疗效观察. 上海针灸杂志 27:22-24.

[452]Cheng Xiao-fang, Jiang Jin-ping, Peng Yan-ling, Chen Yu-feng, Shi Lin-qing. (2008)Observations on the Efficacy of Electroacupuncture plus Point Injection in Treating Lumbar Intervertebral Disc Protrusion. SHANGHAI JOURNAL OF ACUPUNCTURE AND MOXIBUSTION. 27:22-24.

[453] 叶天申,王庆佳,谢文霞,陈勇,何金彩. (2008)腹针治疗原发性失眠症的随机对照研究. 上海针灸杂志 27:3-5.

[453]Ye Tian-shen, Wang Qing-jia, Xie Wen-xia, Chen Yong. (2008)A Randomized Control Study of Abdominal Acupuncture Treatment for Primary Insomnia. SHANGHAI JOURNAL OF ACUPUNCTURE AND MOXIBUSTION. 27:3-5.

[454] 黄婕,范郁山. (2008)浅刺针法治疗肥胖症临床观察. 上海针灸杂志 27:11-12.

[454]Huang Jie, Fan Yu-shan. (2008)Clinical Observations on Treatment of Obesity by Shallow Needling Method. SHANGHAI JOURNAL OF ACUPUNCTURE AND MOXIBUSTION. 27:11-12.

[455] 许军峰,卞金玲,李金波, 申鹏飞,边丽娜,等. (2008)经筋围刺加电针治疗中风后三角肌萎缩30例. 上海针灸杂志 27:28.

[455]Xu Jun-feng, Bian Jin-ling, Li Jin-bo, Shen Peng-fei, Bian Li-na. (2008)The tendons surrounding needling plus Electroacupuncture for treatment of poststroke deltoid muscle atrophy in 30 cases. SHANGHAI JOURNAL OF ACUPUNCTURE AND MOXIBUSTION. 27:28.

[456] 朱永志,李崖雪,张桂波, 陈英华,李岩,等. (2008)针刺配方对中风患者血浆中GMP-140的影响及临床疗效观察. 上海针灸杂志 27:3-4.

[456]Zhu Yong-zhi, Li Ya-xue, Zhang Gui-bo, Chen Ying-hua, Li Yan et.al. (2008)Influences of Acupuncture Prescriptions on Serum GMP-140 and Their Clinical Effects in Stroke Patients. SHANGHAI JOURNAL OF ACUPUNCTURE AND MOXIBUSTION. 27:3-4.

[457] 顾煜,许敬人. (2008)不同温针灸治疗膝骨关节炎疗效观察. 上海针灸杂志 27:13-14.

[457]Gu Yu, Xu Jing-ren. (2008)Evaluation of the Clinical Efficacies of Different Types of Warm Needling in Treating Genual Osteoarthritis. SHANGHAI JOURNAL OF ACUPUNCTURE AND MOXIBUSTION. 27:13-14.

[458] 周子信,王仙梅. (2008)针刺加骶管注药治疗腰椎间盘突出症疗效观察. 上海针灸杂志 27:17-18.

[458]Zhou Zi-xin, Wang Xian-mei. (2008)Drug injection in the treatment of lumbar disc herniation curative effect observation of acupuncture combined with sacral canal. SHANGHAI JOURNAL OF ACUPUNCTURE AND MOXIBUSTION. 27:17-18.

[459] 孙奎,杨永晖,周忠良,杨骏,沈德凯. (2008)隔附子饼灸治疗肝肾不足型膝骨关节炎的临床观察. 上海针灸杂志 27:9-10.

[459]Sun Kui, Yang Yong-hui, Zhou Zhong-liang, Yang Jun, Shen De-kai. (2008)Clinical Observations on the Treatment of Primary Genual Osteoarthritis of Liver-kidney Depletion Type by Aconite Cakeseparated Moxibustion. SHANGHAI JOURNAL OF ACUPUNCTURE AND MOXIBUSTION. 27:9-10.

[460] 周立华,唐英,卢依平,路玫. (2008)艾灸法治疗艾滋病脾气虚腹泻的临床研究. 上海针灸杂志 27:17-19.

[460]Zhou Li-hua, Tang Ying, Lu Yi-ping, Lu Mei. (2008)Clinical Observations on Moxibustion Treatment of Aids Diarrhea of Spleen Qi Deficiency Type. SHANGHAI JOURNAL OF ACUPUNCTURE AND MOXIBUSTION. 27:17-19.

[461] 朱慎勇. (2008)双针巨刺治疗偏瘫后肩痛疗效观察. 上海针灸杂志 27:26-27.

[461]Zhu Shen-yong. (2008)Observations on the Efficacy of Double-needle Contralateral Meridian Acupuncture in Treating Posthemiplegic Omalgia. SHANGHAI JOURNAL OF ACUPUNCTURE AND MOXIBUSTION. 27:26-27.

[462] 周晓平,陈尚杰,何锦添,李红. (2008)调神通络针刺法治疗脑梗死轻度认知障碍的临床观察. 上海针灸杂志 27:3-4.

[462]Zhou Xiao-ping, Chen Shang-jie, He Jin-tian, Li Hong. (2008)Clinical Observations on Treatment of Post-Cerebral Infarction Mild Cognitive Dysfunction by Mind-Regulating and Meridian-Unblocking Acupuncture. SHANGHAI JOURNAL OF ACUPUNCTURE AND MOXIBUSTION. 27:3-4.

[463] 陈尚杰,符文彬,李红, 樊莉. (2008)不同针刺法对脑梗死无认知障碍患者的CSS及MBI的影响. 上海针灸杂志 27:8-9.

[463]Chen Shang-jie, Fu Wen-bin, Li Hong, Fan Li et.al. (2008)Effects of Different Needling Methods on CSS and MBI in Cerebral Infarction Patients without Cognitive Dysfunction. SHANGHAI JOURNAL OF ACUPUNCTURE AND MOXIBUSTION. 27:8-9.

[464] 郑芙蓉,龚东方. (2008)针刺配合电磁疗治疗乳腺增生病临床观察. 上海针灸杂志 27:13-14.

[464]Zheng Fu-rong, Gong Dong-fang. (2008)Clinical Observations on Treatment of Mastoplasia by Acupuncture plus Electromagnetotherapy. SHANGHAI JOURNAL OF ACUPUNCTURE AND MOXIBUSTION. 27:13-14.

[465] 盛益国. (2008)针刺耳压为主对单纯性肥胖症患者腹部三围的影响. 上海针灸杂志 27:17-18.

[465]Sheng Yi-guo.(2008)Acupuncture of auricular pressure effect on simple obesity in patients with abdominal measurements. SHANGHAI JOURNAL OF ACUPUNCTURE AND MOXIBUSTION. 27:17-18.

[466] 孙舟红,宋丽. (2008)温针疗法治疗花粉症疗效观察. 上海针灸杂志 27:19-20.

[466]Sun Zhou-hong, Song Li. (2008)Observations on the Therapeutic Effect of Warm Needling on Pollinosis. SHANGHAI JOURNAL OF ACUPUNCTURE AND MOXIBUSTION. 27:17-18.

[467] 黄皖生. (2008)腹针牵引推拿并用治疗腰椎间盘突出症疗效观察. 上海针灸杂志 27(6):23-24.

[467]Huang Wan-sheng. (2008)Observations on the Efficacy of Combined Use of Abdominal Acupuncture, Traction and Massotherapy in Treating Lumbar Intervertebral Disc Protrusion. SHANGHAI JOURNAL OF ACUPUNCTURE AND MOXIBUSTION. 27(6):23-24.

[468] 孙钰. (2008)齐刺治疗腰椎间盘突出症疗效观察. 上海针灸杂志 27:25-26.

[468]Sun Yu. (2008)Clinical Observations on Treatment of Lumbar Intervertebral Disc Protrusion by Triple Needling. SHANGHAI JOURNAL OF ACUPUNCTURE AND MOXIBUSTION. 27:25-26.

[469] 汪崇淼,吴耀持,朱伟民, 张峻峰,周景辉. (2008)透刺配合手法治疗肩周炎临床观察. 上海针灸杂志 27:27-28.

[469]Wang Chong-miao, Wu Yao-chi, Zhu Wei-min, Zhang Jun-feng, Zhou Jing-hui. (2008)Clinical Study of Treatment of Shoulder Periarthritis by Point-through-point Acupuncture plus Manipulation. SHANGHAI JOURNAL OF ACUPUNCTURE AND MOXIBUSTION. 27:27-28.

[470] 张翠彦,郭惠杰,袁巍. (2008)温针和激光穴位照射治疗颞颌关节功能紊乱的疗效比较.上海针灸杂志 27:29-30.

[470]Zhang Cui-yan, Guo Hui-jie, Yuan Wei. (2008)Comparison of the Efficacy of Warm Needling with Laser Radiation in Treating Temporomandibular Joint Dysfunction. SHANGHAI JOURNAL OF ACUPUNCTURE AND MOXIBUSTION. 27:29-30.

[471] 肖晓华,李瑞春,朱红霞,帅记焱,徐明芳. (2008)头穴透刺电针治疗缺血性中风临床观察. 上海针灸杂志 27:6-8.

[471]Xiao Xiao-hua, Li Rui-chun, Zhu Hong-xia, Shuai Ji-yan, Xu Ming-fang. (2008)Clinical Study of the Treatment of Ischemic Stroke with Scalp Point-through-point Electro acupuncture. SHANGHAI JOURNAL OF ACUPUNCTURE AND MOXIBUSTION. 27:6-8.

[472] 李汶阳,刘智艳. (2008)不同取穴方法治疗缺血性中风临床观察. 上海针灸杂志 27:9-10.

[472]Li Wen-yang, Liu Zhi-yan. (2008)Clinical Observations on Treatment of Ischemic Stroke by Different Methods of Selecting Points. SHANGHAI JOURNAL OF ACUPUNCTURE AND MOXIBUSTION. 27:9-10.

[473] 张航曼. (2008)温针治疗颞颌关节紊乱病临床观察. 上海针灸杂志 27:26-27.

[473]Zhang Hang-man. (2008)Clinical Observations on Treatment of Temporomandibular Joint Disorder by Warm Needling. SHANGHAI JOURNAL OF ACUPUNCTURE AND MOXIBUSTION. 27:26-27.

[474] 赵利华,韦良玉,刘布谷, 周路,黄均琼,等. (2008)针刺配合温和灸治疗脑动脉硬化症认知功能减退的临床观察. 上海针灸杂志 27:3-4.

[474]Zhao Li-hua, Wei Liang-yu, Liu Bu-gu, Zhou Lu, Huang Jun-qiong et.al. (2008)Clinical Study of Treatment of Cognitive Hypofunction Due to Cerebral Arteriosclerosis by Acupuncture plus Gentle Moxi-bustion. SHANGHAI JOURNAL OF ACUPUNCTURE AND MOXIBUSTION. 27:3-4.

[475] 江钢辉,李湘力. (2008)靳氏舌三针治疗中风运动性失语症疗效观察. 上海针灸杂志 27:5-6.

[475]Jiang Gang-hui, Li Xiang-li. (2008)Observations on the Efficacy of Jin's Tongue Three Needles in Treating Apoplectic Motor Aphasia. SHANGHAI JOURNAL OF ACUPUNCTURE AND MOXIBUSTION. 27:5-6.

[476] 佟帅,刘建桥. (2008)舌针加电针治疗吞咽困难疗效观察. 上海针灸杂志 27:9-10.

[476]Tong Shuai, Liu Jian-qiao. (2008)Observations on the Efficacy of Tongue Acupuncture plus Electroacupuncture in Treating Dysphagia. SHANGHAI JOURNAL OF ACUPUNCTURE AND MOXIBUSTION. 27:9-10.

[477] 杨奕平. (2008)针刺配合中药熏洗治疗慢性肛周湿疹疗效观察. 上海针灸杂志 27:23-24.

[477]Yang Yi-ping. (2008)Observations on the Efficacy of Acupuncture plus Fumigating with Chinese Herbal Medicine in Treating Chronic Perianal Eczema. SHANGHAI JOURNAL OF ACUPUNCTURE AND MOXIBUSTION. 27:23-24.

[478] 王正心,陈光辉,施小成. (2008)针刺加药物注射治疗膝骨关节炎临床观察. 上海针灸杂志 27:25-26.

[478]Wang Zheng-xin, Chen Guang-hui, Shi Xiao-cheng. (2008)Clinical Observations on Treatment of Genual Osteoarthritis by Acupuncture plus Drug Injection. SHANGHAI JOURNAL OF ACUPUNCTURE AND MOXIBUSTION. 27:25-26.

[479]杨志新,卞金玲,许军峰,申鹏飞,熊杰,等. (2008)针刺治疗脑梗死恢复期多中心随机对照研究——中医证候疗效评价的报告. 上海针灸杂志 27:3-6.

[479]Yang Zhi-xin, Bian Jin-ling, Xu Jun-feng, Shen Peng-fei, Xiong Jie et.al. (2008)A Multicenter Randomized Controlled Trial of Acupuncture Treatment for the Convalescent Stage of Cerebral Infarction-Re-port on the Assessment of Therapeutic Effects on Syndrome in Traditional Chinese Medicine. SHANGHAI JOURNAL OF ACUPUNCTURE AND MOXIBUSTION. 27:3-6.

[480] 吴笛,吴艳华,谢永红. (2008)项针配合药物治疗颈性眩晕临床观察. 上海针灸杂志 27(9):23-24.

[480]Wu Di, Wu Yan-hua, Xie Yong-hong. (2008)Clinical Observations on Treatment of Cervical Vertigo with Nape Acupuncture plus Medicine. SHANGHAI JOURNAL OF ACUPUNCTURE AND MOXIBUSTION. 27(9):23-24.

[481] 李共信,张锡三,陈淑彦,王建林. (2008)火针治疗肩关节周围炎临床观察. 上海针灸杂志 27:27-28.

[481]Li Gong-xin, Zhang Xi-san, Chen Shu-yan, Wang Jian-lin. (2008)Clinical Observations on Treatment of Shoulder Periarthritis by Fire Needling. SHANGHAI JOURNAL OF ACUPUNCTURE AND MOXIBUSTION. 27:27-28.

[482] 钟伟泉,侯乐.(2008)针刺落枕穴加牵引治疗落枕临床观察. 上海针灸杂志 27:32-33.

[482]Zhong Wei-quan, Hou Le. (2008)Clinical Observations on Treatment of Stiffneck by Point Laozhen Acupuncture plus Traction. SHANGHAI JOURNAL OF ACUPUNCTURE AND MOXIBUSTION. 27:32-33.

[483] 周长山,孔德清,韩正勇. (2008)苍龟探穴法针刺极泉穴对脑卒中腕-手功能的影响. 上海针灸杂志 27:34.

[483]Zhou Chang-shan, Kong De-qing, Han Zheng-yong. (2008)Green Tortoise Exploring effects of acupuncture extremely fontal acupuncture point on hand function of wrist - stroke. SHANGHAI JOURNAL OF ACUPUNCTURE AND MOXIBUSTION. 27:34.

[484] 马玉琴,张健,金龙涛,王小平,王静,等. (2008)分期循经针刺配合运动疗法治疗中风偏瘫临床观察. 上海针灸杂志 27:3-5.

[484]Ma Yu-qin, Zhang Jian, Jin Long-tao, Wang Xiao-ping, Wang Jing et.al. (2008)Clinical Observations on Treatment of Apoplectic Hemiplegia with Along-meridian Acupuncture by Stages plus Kinesitherapy. SHANGHAI JOURNAL OF ACUPUNCTURE AND MOXIBUSTION. 27:3-5.

[485] 孙远征,徐莺莺. (2008)温针治疗糖尿病周围神经病变临床观察. 上海针灸杂志 27:6-8.

[485]Sun Yuan-zheng, Xu Ying-ying. (2008)Clinical Observations on Treatment of 26 Diabetic Peripheral Neuropathy Patients by Warm Needling. SHANGHAI JOURNAL OF ACUPUNCTURE AND MOXIBUSTION. 27:6-8.

[486] 袁广宇. (2008)电针加康复治疗周围神经损伤临床观察. 上海针灸杂志,,27:9-10.

[486]Yuan Guang-yu. (2008)Clinical Observations on Treatment of Peripheral Nerve Injury by Electroacupuncture plus Rehabilitation. SHANGHAI JOURNAL OF ACUPUNCTURE AND MOXIBUSTION. 27:9-10.

[487] 胡晶晶,张唐法,张红星,孙国杰,刘翼程. (2009)头针对血管性痴呆患者智能量表的临床观察. 上海针灸杂志 28:15-17.

[487]Hu Jing-jing, Zhang Tang-fa, Zhang Hong-xing, Sun Guo-jie, Liu Yi-cheng. (2008)Clinical observations on the Efficacy of Scalp Acupuncture Treatment for Vascular Dementia Due to Cerebral Infarction by Means of Intelligence Scales. SHANGHAI JOURNAL OF ACUPUNCTURE AND MOXIBUSTION. 28:15-17.

[488] 高维滨,刘勇,倪金霞, 朱文增,李晓宁. (2009)项针治疗中风后假性延髓麻痹的临床研究. 上海针灸杂志 28:18-20.

[488]Gao Wei-bin, Liu Yong, Ni Jin-xia, Zhu Wen-zeng, Li Xiao-ning. (2008)Clinical Study of Nape Acupuncture Treatment for Pseudobulbar Paralysis. SHANGHAI JOURNAL OF ACUPUNCTURE AND MOXIBUSTION. 28:18-20.

[489] 李建忠. (2009)透刺与中药并用治疗肿瘤患者顽固性呃逆疗效观察. 上海针灸杂志 28:23-24.

[489]Li Jian-zhong. (2008)Observations on the Therapeutic Effect of Point-through-point Acupuncture plus Chinese Herbal Medicine on Intractable Hiccup. SHANGHAI JOURNAL OF ACUPUNCTURE AND MOXIBUSTION. 28:23-24.

[490] 庄子齐,王敦建. (2009)电针百会、神门对腰椎间盘突出症疼痛的影响. 上海针灸杂志 28:33-35.

[490]Zhuang Zi-ji, Wang Dun-jian. (2008)Effect of Electroacupuncture at Baihui and Shenmen on Pain in Lumbar Intervertebral Disc Herniation. SHANGHAI JOURNAL OF ACUPUNCTURE AND MOXIBUSTION. 28:33-35.

[491] 段慧,左小红. (2009)温排针治疗膝关节内侧副韧带损伤疗效观察. 上海针灸杂志 28:39-41.

[491]Duan Hui, Zuo Xiao-hong. (2008)Observations on the Efficacy of Warning Acupuncture in Treating Medial Collateral Ligament Injury of Knee Joint. SHANGHAI JOURNAL OF ACUPUNCTURE AND MOXIBUSTION. 28:39-41.

[492] 李博文,李琳,张招娣,黄选玮,陈久毅.(2009)黄选玮针刺治疗肩周炎临床观察. 上海针灸杂志 28:42-43.

[492]Li Bo-wen, Li Lin, Zhang Zhao-di, Huang Xuan-wei, Chen Jiu-yi. (2008)Clinical Observations on Treatment of Shoulder Periarthritis with Huang Xuan-wei's Acupuncture. SHANGHAI JOURNAL OF ACUPUNCTURE AND MOXIBUSTION. 28:42-43.

[493] 李艺,夏勇,刘世敏, 具紫勇,史晓岚,等. (2009)电针对围绝经期综合征患者Kupperman评分的效应. 上海针灸杂志 28:559-561.

[493]Li Yi, Xia Yong, Liu Shi-min, Ju Zi-yong, Shi Xiao-lan et.al. (2008)Effect of Electroacupuncture on Kupperman Index Scores in Patients with Perimenopausal Syndrome. SHANGHAI JOURNAL OF ACUPUNCTURE AND MOXIBUSTION. 28:559-561.

[494] 金瑛,王爱君,薛平,谢蔚,陈志珍. (2009)温针治疗脾虚湿阻型单纯性肥胖症临床观察. 上海针灸杂志 28:565-567.

[494]Jin Ying, Wang Ai-jun, Xue Ping, Xie Wei, Chen Zhi-zhen. (2008).Clinical Observations on Warm Needling Treatment for Simple Obesity of Spleen Deficiency and Dampness Encumbrance Type. SHANGHAI JOURNAL OF ACUPUNCTURE AND MOXIBUSTION. 28:565-567.

[495] 陆斌,周俊灵. (2009)盛氏面三针治疗Bell面瘫临床疗效观察. 上海针灸杂志 28:568-571.

[495]Lu Bin, Zhou Jun-ling. (2008)Observations on the Clinical Efficacy of Sheng's Facial Three-needle Therapy for Bell's Palsy. SHANGHAI JOURNAL OF ACUPUNCTURE AND MOXIBUSTION. 28:568-571.

[496] 陈向东,张红,黄克勤. (2009)早期针刺干预对急性面神经炎预后的影响. 上海针灸杂志 28:572-574.

[496]Chen Xiang-dong, Zhang Hong, Huang Ke-qin. (2008)Influence of Early Acupuncture Intervention on the Prognosis of Acute Facial Neuritis. SHANGHAI JOURNAL OF ACUPUNCTURE AND MOXIBUSTION. 28:572-574.

[497] 李小军,柳文丹,胡彩虹. (2009)头针配合运动疗法对偏瘫体感诱发电位的影响. 上海针灸杂志 28:575-576.

[497] Li Xiao-jun, Liu Wen-dan, Hu Cai-hong. (2009)Influence of Concomitant Scalp Acupuncture and Kinetotherapy on Somatosensory Evoked Potential in Hemiplegia Patients. SHANGHAI JOURNAL OF ACUPUNCTURE AND MOXIBUSTION. 28:575-576.

[498] 陈敏, 钱云霞. (2009)针刺治疗带状疱疹后遗神经痛疗效观察. 上海针灸杂志 28(10):585-586.

[498] Chen Min, Qian Yun-xia. (2009)Observations on the Efficacy of Acupuncture in Treating Postherpetic Neuralgia. SHANGHAI JOURNAL OF ACUPUNCTURE AND MOXIBUSTION. 28(10):585-586.

[499] 张学健. (2009)芒针透刺治疗慢性前列腺炎疗效观察. 上海针灸杂志 28:589-590.

[499] Zhang Xue-jian. (2009)Observations on the Efficacy of Point-through-point Acupuncture with Elongate Needles in Treating Chronic Prostatitis. SHANGHAI JOURNAL OF ACUPUNCTURE AND MOXIBUSTION. 28:589-590.

[500] 佟帅,刘建桥. (2009)针刺配合红外偏振光治疗过敏性鼻炎疗效观察. 上海针灸杂志28:639-640.

[500] Tong Shuai, Liu Jian-qiao. (2009)Observations on the Therapeutic Effect of Acupuncture Assisted with Polarized Infrared on Allergic Rhintis. SHANGHAI JOURNAL OF ACUPUNCTURE AND MOXIBUSTION. 28:639-640.

[501] 孙治东,王娟娟. (2009)穴位埋线治疗腰椎间盘突出症疗效观察. 上海针灸杂志28:652-654.

[501] Sun Zhi-dong, Wang Juan-juan. (2009)Observations on the Efficacy of New Medicated Thread Embedding Therapy for Lumbar Intervertebral Disc Herniation. SHANGHAI JOURNAL OF ACUPUNCTURE AND MOXIBUSTION. 28:652-654.

[502] 连方,刘梅,张建伟,张宁,孙振高等. (2009)针刺联合腔内理疗治疗黄素化未破裂卵泡综合征临床观察.上海针灸杂志28:685-688.

[502] Lian Fang, Liu Mei, Zhang Jian-wei, Zhang Ning, Sun Zhen-gao et.al. (2009)Clinical Observations on Combined Treatment of Luteinized Unruptured Follicle Syndrome (LUFS) with Acupuncture and Intracavitary Physiotherapy. SHANGHAI JOURNAL OF ACUPUNCTURE AND MOXIBUSTION. 28:685-688.

[503] 魏红沁,丁春华,张晴,朱晓宏.(2009)艾灸治疗高脂血症临床观察.上海针灸杂志28:698-699.

[503] Wei Hong-qin, Ding Chun-hua, Zhang Qing, Zhu Xiao-hong. (2009)Clinical Observations on Moxibustion Treatment for Hyperlipemia. SHANGHAI JOURNAL OF ACUPUNCTURE AND MOXIBUSTION. 28:698-699.

[504] 潘文宇. (2009)电针加穴位埋线治疗寻常痤疮疗效观察.上海针灸杂志28:703-704.

[504] Pan Wen-yu. (2009)Observations on the Efficacy of Electroacupuncture plus Acupoint Catgut Embedding in Treating Acne Vulgaris. SHANGHAI JOURNAL OF ACUPUNCTURE AND MOXIBUSTION. 28:703-704.

[505] 史江峰. (2009)俞募原配穴针刺治疗中风后便秘临床观察.上海针灸杂志28:709-710.

[505] Shi Jiang-feng. (2009)Clinical Observations on the Treatment of Postapoplectic Constipation by Back-Shu-Front-Mu- Source Point Combination. SHANGHAI JOURNAL OF ACUPUNCTURE AND MOXIBUSTION. 28:709-710.

[506] 王茵萍,仲远明,赵卫梅. (2009)解剖针刺法治疗慢性软组织损伤疗效观察.上海针灸杂志28:719-721.

[506] Wang Yin-ping, Zhong Yuan-ming, Zhao Wei-mei. (2009)Observations on the Efficacy of Anatomic Needling Method in Treating Chronic Soft Tissue Injury. SHANGHAI JOURNAL OF ACUPUNCTURE AND MOXIBUSTION. 28:719-721.

[507] 张峻峰,吴耀持,李石胜. (2009)透刺温针灸治疗膝骨关节炎疗效观察.上海针灸杂志28:722-723.

[507] Zhang Jun-feng, Wu Yao-chi, Li Shi-sheng. (2009)Observations on the Efficacy of Point-through-point Acupuncture and Moxibustion in Treating Genual Osteoarthritis. SHANGHAI JOURNAL OF ACUPUNCTURE AND MOXIBUSTION. 28:722-723.

[508] 林廷樾,黄华超.(2009)刺血加温针灸治疗膝骨关节炎疗效观察.上海针灸杂志28:724-725.

[508] Lin Ting-yue, Huang Hua-chao. (2009)Observations on the Efficacy of Pricking Bloodletting plus Warm Needling in Treating Genual Osteoarthritis. SHANGHAI JOURNAL OF ACUPUNCTURE AND MOXIBUSTION. 28:724-725.

[509] 何乐中.(2009)电针配合耳压治疗糖尿病周围神经病变疗效观察.上海针灸杂志28:100-101.

[509] He Le-zhong. (2009)Observations on the Efficacy of Electroacupuncture plus Auricular Point Plaster Therapy in Treating Diabetic Peripheral Neuropathy. SHANGHAI JOURNAL OF ACUPUNCTURE AND MOXIBUSTION. 28:92-94.

[510] 李勇,符文彬,郭元琦,蒙昌荣.(2009)腹针治疗腰椎间盘突出症临床观察.上海针灸杂志28:92-94.

[510] Li Yong, Fu Wen-bin, Guo Yuan-qi, Meng Ghang-rong. (2009)Clinical Observation on Abdominal Acupuncture Treatment for Lumbar Intervertebral Disc Herniation. SHANGHAI JOURNAL OF ACUPUNCTURE AND MOXIBUSTION. 28:92-94.

[511] 卢洪,彭建明. (2009)针刺加推拿治疗肛肠病术后尿潴留疗效观察.上海针灸杂志28:149-150.

[511] Lu Hong, Peng Jian-ming. (2009)Observations on the Efficacy of Acupuncture Plus Massotherapy in Treating Anorectal Postoperative Uroschesis. SHANGHAI JOURNAL OF ACUPUNCTURE AND MOXIBUSTION. 28:149-150.

[512] 曹莲瑛,沈特立,倪喻斐,张伟.(2009)不同频率的电针配合推拿治疗腰椎间盘突出症疗效观察.上海针灸杂志28:153-155.

[512] Cao Lian-ying, Shen Te-li, Ni Yu-fei, Zhang Wei. (2009)Observations on the Efficacy of Different Frequency Electoacupuncture Plus Massotherapy in Treating Lumbar Intervertebral Disc Herniation. SHANGHAI JOURNAL OF ACUPUNCTURE AND MOXIBUSTION. 28:153-155.

[513] 迟旭,鞠琰莉. (2009)经皮神经电刺激治疗慢传输型便秘疗效观察.上海针灸杂志28:205-206.

[513] Chi Xu, Ju Yan-li. (2009)Transcutaneous Electrical Nerve Stimulator for Treatment of Slow Transit Constipation. SHANGHAI JOURNAL OF ACUPUNCTURE AND MOXIBUSTION. 28:205-206.

[514] 孙善斌,杨骏,张闻东,陈幸生,韩为,等. (2009)辨经刺井法及颞三针治疗血管性痴呆临床观察.上海针灸杂志28:207-209.

[514] Sun Shan-bin, Yang Jun, Zhang Wen-dong, Chen Xing-sheng , Han Wei et.al. (2009)Clinical Observations on Six-meridian Syndrome Differentiation Acupuncture at Well Points and Temporal Three-needle Acupuncture for Treatment of Vascular Dementia. SHANGHAI JOURNAL OF ACUPUNCTURE AND MOXIBUSTION. 28:207-209.

[515] 陈晓军,方针,罗高权,吴凌云.(2009)调和阴阳针刺法治疗急性脑梗死临床观察.上海针灸杂志28:210-212.

[515] Chen Xiao-ju, Fang Zhen, Luo Gao-quan, Wu Ling-yun. (2009)Clinical Observations on Yin-yang Harmonizing Acupuncture Method for Treatment of Acute Cerebral Infarction. SHANGHAI JOURNAL OF ACUPUNCTURE AND MOXIBUSTION. 28:210-212.

[516] 杨城,余宏,吴良慈. (2009)穴位埋线治疗急性面神经麻痹临床观察.上海针灸杂志28:215-216.

[516] Yang Cheng, Yu Hong, Wu Liang-ci. (2009)Clinical Observations on Catgut Embedding at Acupoints for Treatment of Acute Facial Paralysis. SHANGHAI JOURNAL OF ACUPUNCTURE AND MOXIBUSTION. 28:215-216.

[517] 陈丹,王友军,吴荣华,胡幼平.(2009)傍刺治疗神经根型颈椎病临床疗效的量化评价.上海针灸杂志28:219-221.

[517] ChenDan, Wang You-jun, Wu Rong-hua, Hu You-ping. (2009)Quantitative Evaluation of the Clinical Efficacy of Proximal Needling in Treating Cervical Spondylosis of Nerve Root Type. SHANGHAI JOURNAL OF ACUPUNCTURE AND MOXIBUSTION. 28:219-221.

[518] 王东岩,王岩,杨晓东.(2009)不同穴位与肌肉运动点针刺在脑卒中患者腕手功能重建中的对比研究.上海针灸杂志28:253-255.

[518] Wang Dong-yan[1], Wang Yan[2], Yang Xiao-dong. (2009)Comparative Study of Wrist-hand Function Rebuilding Between Acupuncture at Different Acupoints and at Muscular Motor Points in Cerebral Stroke Patients. SHANGHAI JOURNAL OF ACUPUNCTURE AND MOXIBUSTION. 28:253-255.

[519] 孙秋红,张会平,宋桂红,薛桂华.(2009)温针灸治疗慢性腹泻疗效观察.上海针灸杂志28:258-259.

[519] Sun Qiu-hong, Zhang Hui-ping, Song Gui-hong, Xue Gui-hua. (2009)Clinical Observations on Warm Needling for the Treatment of 100 Patients with Chronic Diarrhea. SHANGHAI JOURNAL OF ACUPUNCTURE AND MOXIBUSTION. 28:258-259.

[520] 陆军伟,孙建华.(2009)颈项针为主治疗假性球麻痹疗效观察.上海针灸杂志28:262-265.

[520] Lu Jun-wei, Sun Jian-hua. (2009)Observations on the Therapeutic Effect of Nape Acupuncture as a Main Treatment for Pseudobulbar Palsy. SHANGHAI JOURNAL OF ACUPUNCTURE AND MOXIBUSTION. 28:262-265.

[521] 陈龙安,叶晓品,何永江.(2009)穴位埋线结合针刀治疗顽固性网球肘疗效观察.上海针灸杂志28:266-267.

[521] Chen Long-an,Ye Xiao-pin, He Yong-jiang. (2009)Observations on the Efficacy of a Knife Needle plus Acupoint Catgut-Embedding in Treating Refractory Tennis Elbow. SHANGHAI JOURNAL OF ACUPUNCTURE AND MOXIBUSTION. 28:266-267.

[522] 刘磊,伦新.(2009)头部不同针刺法治疗梗塞性痴呆临床观察.上海针灸杂志28:313-315.

[522] Liu Lei, Lun Xin. (2009)Clinical Observations on Different Scalp Needling Methods for the Treatment of Multiple Infarcted Dementia. SHANGHAI JOURNAL OF ACUPUNCTURE AND MOXIBUSTION. 28:313-315.

[523] 胡幼平,刁骧,尹重,王友军.(2009)关刺加温针灸对肩周炎患者临床疗效的量化评价.上海针灸杂志28:336-338.

[523] Hu You-ping[1], Diao Xiang[2], Yin Chong[3], WANG You-jun. (2009)Quantitative Assessment of the Clinical Efficacy of Joint Needling plus Warm Needling in Treating Shoulder Periarthritis Patients. SHANGHAI JOURNAL OF ACUPUNCTURE AND MOXIBUSTION. 28:336-338.

[524] 朱捷.(2009)针刺配合推拿治疗颈胸段椎小关节错缝的近远期疗效观察.上海针灸杂志28:343-345.

[524] Zhu Jie. (2009)Observations on the Shod-term and Long-term Efficacies of Acupuncture plus Massotherapy in Treating Cervicothoracic Facet Joint Semi-dislocation. SHANGHAI JOURNAL OF ACUPUNCTURE AND MOXIBUSTION. 28:343-345.

[525] 陈宏伟,杨国荣,唐永春.(2009)电针治疗偏头痛疗效观察.上海针灸杂志28:353-354.

[525] Chen Hong-wei, Yang Guo-rong, Tang Yong-chun. (2009)Observation of curative effect of electroacupuncture for treatment of migraine. SHANGHAI JOURNAL OF ACUPUNCTURE AND MOXIBUSTION. 28:353-354.

[526] 李小军,郑斌.(2009)早期头针结合现代康复技术对脑卒中后偏瘫康复疗效观察.上海针灸杂志28:380-382.

[526] Li Xiao-jun, Zheng Bin. (2009)Observations on the Efficacy of Early Scalp Acupuncture plus Modern Rehabilitation Techniques in Treating Postapoplectic Hemiplegia. SHANGHAI JOURNAL OF ACUPUNCTURE AND MOXIBUSTION. 28:380-382.

[527] 李淑萍,倪勇,霍国敏.(2009)不同针法对急性脑梗死患者体感诱发电位的影响.上海针灸杂志28:386-387.

[527] Li Shu-ping, Ni Yong, Huo Guo-min. (2009)Effects of Different Needling Methods on Somatosensory Evoked Potential in Patients with Acute Cerebral Infarction. SHANGHAI JOURNAL OF ACUPUNCTURE AND MOXIBUSTION. 28:386-387.

[528] 陈朋,高汉义,吉秀芹,刘捷,姜梅芳,等.(2009)针刺配合穴位注射治疗特发性周围性面神经麻痹疗效分析.上海针灸杂志28:392-394.

[528] Chen Peng, Gao Han-yi, Ji Xiu-qin, Liu Jie, Jiang Mei-fang et.al. (2009)Analysis of the Efficacy of Acupuncture plus Acupuncture Point Injection in Treating Idiopathic Peripheral Facial Paralysis. SHANGHAI JOURNAL OF ACUPUNCTURE AND MOXIBUSTION. 28:392-394.

[529] 周立武.(2009)燔针劫刺治疗肩峰下滑囊炎临床观察.上海针灸杂志28:406-407.

[529] Zhou Li-wu. (2009)Clinical Observations on Fire Needling Treatment for Subacromial Bursitis. SHANGHAI JOURNAL OF ACUPUNCTURE AND MOXIBUSTION. 28:406-407.

[530] 许军峰.(2009)委中穴不同刺法对中风患者下肢功能障碍的影响.上海针灸杂志28:439-441.

[530] Xu Jun-feng. (2009)Effects of Different Point Weizhong Needling Methods on Lower Limb Dysfunction in Stroke Patients. SHANGHAI JOURNAL OF ACUPUNCTURE AND MOXIBUSTION. 28:439-441.

[531] 曾睿,唐俊良.(2009)雷火灸配合推拿治疗慢性疲劳综合征疗效观察.上海针灸杂志28:445-446.

[531] Zeng Rui, Tang Jun-liang. (2009)Observations on the Efficacy of Thunder-fire Moxibustion plus Massotherapy in Treating Chronic Fatigue Syndrome. SHANGHAI JOURNAL OF ACUPUNCTURE AND MOXIBUSTION. 28:445-446.

[532] 付晓红,张巧玲.(2009)鼻三针配合穴位贴敷治疗变应性鼻炎疗效观察.上海针灸杂志28:452-453.

[532] Fu Xiao-hong, Zhang Qiao-ling. (2009)Observations on the Efficacy of Jin's Nasal Three-point Needling plus Acupoint Application in Treating Allergic Rhinitis. SHANGHAI JOURNAL OF ACUPUNCTURE AND MOXIBUSTION. 28:452-453.

[533] 王晶青,朱洪生.(2009)电针辅助静脉麻醉用于无痛人流的临床观察.上海针灸杂志28:456-457.

[533] Wang Jing-qing, Zhu Hong-sheng. (2009)Clinical Observations on Electroacupuncture-assisted Intravenous Anesthesia for Painless Artificial Abortion. SHANGHAI JOURNAL OF ACUPUNCTURE AND MOXIBUSTION. 28:456-457.

[534] 宣益民,赵媛.(2009)浅刺法治疗急性缺血性中风偏瘫疗效观察.上海针灸杂志28:507-509.

[534] Xuan Yi-min, Zhao Yuan. (2009)Observations on the Efficacy of Shallow Needling Method in Treating Acute Ischemic Stroke Hemiplegia. SHANGHAI JOURNAL OF ACUPUNCTURE AND MOXIBUSTION. 28:507-509.

[535] 张中一,刘茵.(2009)电针干预急性期周围性面瘫临床疗效观察.上海针灸杂志28:517-519.

[535] Zhang Zhong-yi, Liu Yin. (2009)Observations on the Clinical Efficacy of Electroacupuncture Intervention in Treating Acute-stage Peripheral Facial Paralysis. SHANGHAI JOURNAL OF ACUPUNCTURE AND MOXIBUSTION. 28:517-519.

[536] 刘银妮,张红星,黄国付,邹燃,魏巍.(2009)电针夹脊穴配合刺络拔罐治疗带状疱疹疗效观察.上海针灸杂志28:523-525.

[536] Liu Yin-ni, Zhang Hong-xing, Huang Guo-fu, Zhou Ran, Wei Wei. (2009)

Observations on the Efficacy of Electroacupuncture at Huatuojiaji plus Pricking-cupping Bloodletting in Treating Herpes Zoster. SHANGHAI JOURNAL OF ACUPUNCTURE AND MOXIBUSTION. 28:523-525.

[537] 陈纯涛,张颜,黄蜀. (2009)火针治疗肝郁痰凝型扁平疣疗效观察.上海针灸杂志28:526-527.

[537] Chen Chun-tao, Zhuang Yan, Huang Shu. (2009)Observations on the Efficacy of Fire Needling in Treating Verruca Plana of Liver Qi Depression and Phlegm Stasis Type. SHANGHAI JOURNAL OF ACUPUNCTURE AND MOXIBUSTION. 28:526-527.

[538] 彭良. (2010)多针透刺配合梅花针叩刺治疗周围性面神经麻痹疗效观察.上海针灸杂志29:17-18.

[538] Peng Liang. (2010)Observations on the Efficacy of Point-to-point Acupuncture with Several Needles plus Plum-blossom Needle Tapping in Treating Peripheral Facial Paralysis. SHANGHAI JOURNAL OF ACUPUNCTURE AND MOXIBUSTION. 29:17-18.

[539] 聂斌,罗仁瀚,陈秀玲,徐凯.(2010)雷火灸治疗虚寒型胃痛疗效观察.上海针灸杂志29:21-22.

[539] Nie Bin, Luo Ren-han, Chen Xiu-ling, Xu Kai. (2010)Observations On the Efficacy of Thunder-fire Moxibustion in Treating Gastralgia of Deficiency Cold Type. SHANGHAI JOURNAL OF ACUPUNCTURE AND MOXIBUSTION. 29:21-22.

[540] 李运峰. (2010)芒针治疗胃下垂疗效观察.上海针灸杂志29:23-24.

[540] Li Yun-feng. (2010)Observations OB the Efficacy of Elongated Needle Treatment for Gastroptosis. SHANGHAI JOURNAL OF ACUPUNCTURE AND MOXIBUSTION. 29:23-24.

[541] 陈小凯,李旅萍,吴虹,栗刚,蔡红卫.(2010)刺络法对颈椎病细胞间粘附分子-1的影响.上海针灸杂志29:25-27.

[541] Chen Xiao-kai, Li Lu-ping, Wu Hong, Li Gang, Cai Wei-hong. (2010)Study of the Influence of Pricking Bloodletting Method on Intracellular Adhesion Molecule-1 in the Treatment of Cervical Spondylosis. SHANGHAI JOURNAL OF ACUPUNCTURE AND MOXIBUSTION. 229:25-27.

[542] 周平,李国安. (2010)针刺对神经根型颈椎病斜方肌肌张力的影响.上海针灸杂志29:28-30.

[542] Zhou Ping, Li Guo-an. (2010)Clinical Study of the Effect of Acupuncture on Trapezius Muscle Tension in Cervical Spondylotic Radiculopathy. SHANGHAI JOURNAL OF ACUPUNCTURE AND MOXIBUSTION. 29:28-30.

[543] 李莉.(2010)电针与温针治疗强直性脊柱炎临床对照研究.上海针灸杂志29:34-35.

[543] Li Li. (2010)Randomized Clinical Control Study of Electroacupuncture and Warm Needling in Treating Ankylosing Spondylitis. SHANGHAI JOURNAL OF ACUPUNCTURE AND MOXIBUSTION. 29:34-35.

[544] 施曼华.(2010)透刺结合温针治疗肩关节周围炎疗效观察.上海针灸杂志,29:36-37.

[544] Shi Man-hua. (2010)Observations on the Efficacy of Penetration Needling plus Warm Needling in Treating Scapulohumeral Periarthritis. SHANGHAI JOURNAL OF ACUPUNCTURE AND MOXIBUSTION. 29:36-37.

[545] 孟魏魏,蔡秀水. (2010)电针配合健腰操治疗腰椎间盘突出症疗效观察.上海针灸杂志29:40-42.

[545] Meng Wei-wei, Cai Xiu-shui. (2010)Observations on the Efficacy of Electroacupuncture plus Waist-strengthening Exercises in Treating Lumbar Intervertebral Disc Herniation. SHANGHAI JOURNAL OF ACUPUNCTURE AND MOXIBUSTION. 29:40-42.

[546] 周忠良,孙奎,程红亮,刘德春,杨骏. (2010)隔药灸治疗血瘀型膝骨关节炎疗效观察.上海针灸杂志29:45-47.

[546] Zhou Zhong-liang, Sun Kui, Cheng Hong-liang, Hu De-chun, Yang Jun. (2010)Observations on the Therapeutic Effect of Herbal Cake-separated Moxibustion on Knee Osteoarthritis of Blood Stasis Type. SHANGHAI JOURNAL OF ACUPUNCTURE AND MOXIBUSTION. 29:45-47.

[547] 车涛,陈永强,裘敏蕾,孙剑,李岩峰.(2010)电针治疗髌骨软化症疗效观察.上海针灸杂志29:48-49.

[547] Che Tao, Chen Yong-qiang, Qiu Min-lei, Sun Jian, Li Yan-feng. (2010)Observations on the Therapeutic Effect of Electroacupuncture on Chondromalacia Patellae. SHANGHAI JOURNAL OF ACUPUNCTURE AND MOXIBUSTION. 29:48-49.

[548] 金泽,姜珊珊,包大鹏. (2010)电针治疗前列腺增生临床观察.上海针灸杂志29:105-106.

[548] Jin Ze, Jiang Shan-shan, Bao Da-peng. (2010)Clinical Observations on Electroacupuncture Treatment for Prostatic Hyperplasia. SHANGHAI JOURNAL OF ACUPUNCTURE AND MOXIBUSTION. 29:105-106.

[549] 蔡玉梅,陆坚,郑继范,吴毅.(2010)针刺加耳穴贴压对混合痔术后镇痛的临床观察.上海针灸杂志29:107-108.

[549] Cai Yu-mei, Lu Jian, Zheng Ji-fan, Wu Yi. (2010)Clinical Study of the Analgesic Effect of Acupuncture plus Auricular Point Plaster Therapy After Mixed Hemorrhoid Surgery. SHANGHAI JOURNAL OF ACUPUNCTURE AND MOXIBUSTION. 29:107-108.

[550] 申鹏飞,卞金玲,孟志宏,石学敏.(2010)捻转补法针刺人迎穴干预原发性高血压亚急症的效应观察.上海针灸杂志,29:71-73.

[550] Shen Peng-fei, Bian Jin-ling, Meng Zhi-hong, Shi Xue-min. (2010)Observations on the Efficacy of Acupuncture at Point Renying (ST 9) by a Twirling Reinforcement Method in Treating Hypertensive Urgencies in Primary Hypertension Patients. SHANGHAI JOURNAL OF ACUPUNCTURE AND MOXIBUSTION. 29:71-73.

[551] 黄琳娜,安军明,苏同生,王璞,董岚. (2010)头针治疗血管性痴呆随机对照临床研究.上海针灸杂志29:79-82.

[551] Huang Lin-na, An Jun-ming, Su Tong-sheng, Wang Pu, Dong Lan et.al. (2010)Clinical Randomized Controlled Trial of Scalp Acupuncture Treatment for Vascular Dementia. SHANGHAI JOURNAL OF ACUPUNCTURE AND MOXIBUSTION. 29:79-82.

[552] 杜伟. (2010)巨刺加电针疏密波治疗中风偏瘫疗效观察.上海针灸杂志29:86-87.

[552] Du Wei. (2010)Observations on the Efficacy of Contralateral Meridian Needling plus Sparse-dense Wave Electroacupuncture in Treating Stroke Hemiplegia.

SHANGHAI JOURNAL OF ACUPUNCTURE AND MOXIBUSTION. 29:86-87.

[553] 余蓝. (2010)头针为主治疗中风后遗症疗效观察.上海针灸杂志29:88-90.

[553] Yu Lan. (2010)Clinical Observations on the Efficacy of Scalp Acupuncture as a Main Treatment for Apoplectic Sequela. SHANGHAI JOURNAL OF ACUPUNCTURE AND MOXIBUSTION. 29:88-90.

[554] 朱余明,周红,闵屹华,施伶俐.(2010)针刺超前镇痛对开胸手术患者围术期镇痛效果的影响.上海针灸杂志29:620-622.

[554] Zhu Yu-ming, Zhou Hong, Min Yi-hua, Shi Ling-li. (2010)Influence of Advanced Acupuncture Analgesia on Perioperative Analgesic Effect in Thoracotomy Patients. SHANGHAI JOURNAL OF ACUPUNCTURE AND MOXIBUSTION. 29:620-622.

[555] 魏凌霄,许曙,宣益民, 赵媛.(2010)针刺关元穴治疗气郁型月经失调疗效观察.上海针灸杂志29:629-630.

[555] Wei Ling-xiao, Xu Shu, Xuan Yi-min, Zhao Yuan. (2010)Observations on the Efficacy of Hand-twisting Lifting and Thrusting Acupuncture at Point Guanyuan in Treating Menstrual Disorder of Qi Stagnation Type. SHANGHAI JOURNAL OF ACUPUNCTURE AND MOXIBUSTION. 29:629-630.

[556] 段锦绣,彭唯娜,刘志顺, 杨德莉,郭郡,等. (2010)深刺天枢穴改善结肠慢传输型便秘临床观察.上海针灸杂志29:631-633.

[556] Duan Jin-xiu, Peng Wei-na, Liu Zhi-shun, Yang De-li, Guo Jun et.al. (2010)Clinical Observation on the Treatment of Slow Transit Constipation by Deeply Inserting Tianshu Acupoint. SHANGHAI JOURNAL OF ACUPUNCTURE AND MOXIBUSTION. 29:631-633.

[557] 梁谊深,张越,谢胜,王力平,朱初良,等. (2010)穴位埋线配合太极拳治疗便秘型肠易激综合征疗效观察.上海针灸杂志29:634-635.

[557] Liang Yi-shen, Zhang Yue, Xie Sheng, Wang Li-ping, Zhu Chu-liang et.al. (2010)Observations on the Efficacy of Acupoint Catgut Embedding plus Taijiquan in Treating Irritable Bowel Syndrome of Constipation Type. SHANGHAI JOURNAL OF ACUPUNCTURE AND MOXIBUSTION. 29:634-635.

[558] 王为风,陈妙根. (2010)齐刺配合循经辨证治疗腰椎间盘突出症疗效观察.上海针灸杂志29:648-649

[558] Wang Wei-feng, Chen Miao-gen. (2010)Observations on the Efficacy of Triple Needling plus Along-meridian Syndrome Differentiation in Treating Lumbar Intervertebral Disc Herniation. SHANGHAI JOURNAL OF ACUPUNCTURE AND MOXIBUSTION. 29:648-649.

[559] 刘未艾,吴清明,李向荣, 李丹丹,付磊,等. (2010)头电针配合卒中单元综合疗法治疗脑卒中偏瘫疗效观察.上海针灸杂志29:149-151.

[559] Liu Wei-ai, Wu Qing-ming, Li Xiang-rong, Li Dan-dan, Fu Lei et.al. (2010)Observations on the Efficacy of Combined Treatment of Stroke Hemiplegia with Scalp Electroacupuncture and Stroke Unit. SHANGHAI JOURNAL OF ACUPUNCTURE AND MOXIBUSTION. 29:149-151.

[560] 谢冬玲,朱丽芳,刘惠宇, 曾春英.(2010)事件相关电位P300在头皮针治疗康复期脑梗死认知障碍应用.上海针灸杂志29:152-153.

[560] Xie Dong-ling, Zhu Li-fang, Liu Hui-yu, Zeng Chun-ying. (2010)Application of Event-related Potential P300 to Scalp Acupuncture Treatment of Cognitive Disorder in the Convalescent Stage of Cerebral infarction. SHANGHAI JOURNAL OF ACUPUNCTURE AND MOXIBUSTION. 29:152-153.

[561] 何云清,徐静. (2010)七星针叩刺配合拔罐治疗椎动脉型颈椎病疗效观察.上海针灸杂志29:170-171.

[561] He Yun-qing, Xu Jing. (2010)Observations on the Efficacy of Seven-star Needle Tapping plus Cupping in Treating Cervical Spondylotic Vertebral Arteriopathy. SHANGHAI JOURNAL OF ACUPUNCTURE AND MOXIBUSTION. 29:170-171.

[562] 田国平,刘峰. (2010)透刺法配合局部活动治疗肱二头肌长头肌腱鞘炎疗效观察.上海针灸杂志29:174-175.

[562] Tian Guo-pin, Liu Feng. (2010)Observations on the Efficacy of Point-to-point Acupuncture plus Local Movement in Treating Tenosynovitis of Long Head of Biceps Brachii. SHANGHAI JOURNAL OF ACUPUNCTURE AND MOXIBUSTION. 29:174-175.

[563] 李秀彬. (2010)隔物温和灸治疗膝骨关节炎疗效观察.上海针灸杂志29:178-180.

[563] Li Xiu-bin. (2010)Observations on the Therapeutic Effect of Indirect Mild-warm Moxibustion on Knee Osteoarthritis. SHANGHAI JOURNAL OF ACUPUNCTURE AND MOXIBUSTION. 29:178-180.

[564] 李艺,夏勇,刘世敏, 具紫勇,史晓岚,等. (2010)电针对围绝经期综合征患者血清性激素的影响.上海针灸杂志29:199-201.

[564] Li Yi, Xia Yong, Liu Shi-min, Ju Zi-yong, Shi Xiao-lan et.al. (2010)Effect of Electroacupuncture on Serum Sex Hormones in Patients with Perimenopausal Syndrome. SHANGHAI JOURNAL OF ACUPUNCTURE AND MOXIBUSTION. 29:199-201.

[565] 孙占玲,金亚蓓,金慧芳. (2010)耳针治疗围绝经期综合征多中心临床疗效观察.上海针灸杂志29:209-211.

[565] Sun Zhan-ling, Jin Ya-bei, Jin Hui-fang. (2010)A Multicenter Observational Study of the Clinical Efficacy of Ear Acupuncture in Treating Perimenopausal Syndrome. SHANGHAI JOURNAL OF ACUPUNCTURE AND MOXIBUSTION. 29:209-211.

[566] 林华东. (2010)电针配合隔姜灸治疗胃下垂疗效观察.上海针灸杂志,29:221-222.

[566] Lin Hua-dong. (2010)Observations on the Efficacy of Electroacupuncture plus Ginger Moxibustion in Treating Gastroptosis. SHANGHAI JOURNAL OF ACUPUNCTURE AND MOXIBUSTION. 29:221-222.

[567] 洪文,张凌,王丽娟,黄汉传,刘颂豪,等. (2010)激光穴位照射治疗前列腺增生症临床观察.上海针灸杂志29:223-224.

[567] Hong Wen, Zhang Ling, Wang Li-juan, Huang Han-chuan, Liu Song-hao et.al. (2010)Clinical Observations on the Efficacy of Acupoint Laser Radiation in Treating Prostatic Hyperplasia. SHANGHAI JOURNAL OF ACUPUNCTURE AND MOXIBUSTION. 29:223-224.

[568] 口锁堂,吴耀持. (2010)关刺治疗经筋病症临床疗效分析.上海针灸杂志29:237-238.

[568] Kou Suo-tang, Wu Yao-chi. (2010)Analysis of the Clinical Therapeutic Effect of Joint Needling on Muscular System Diseases. SHANGHAI JOURNAL OF ACUPUNCTURE AND MOXIBUSTION. 29:237-238.

[569] 韩淑凯,张宝昌,左永发,问晓玉.(2010)经筋排刺法配合皮肤针治疗脑卒中后上肢痉挛疗效观察.上海针灸杂志29:284-286.

[569] Han Shu-kai, Zhang Bao-chang, Zuo Yong-fa, Wen Xiao-yu. (2010)Observations on the Efficacy of Muscle-region Alignment Needling plus Skin Acupuncture in Treating Post-stroke Upper Limb Spasticity. SHANGHAI JOURNAL OF ACUPUNCTURE AND MOXIBUSTION. 29:284-286.

[570] 杨冲.(2010)温通针法配合透刺治疗顽固性面瘫疗效观察.上海针灸杂志29:287-288.

[570] Yang Chong. (2010)Observations on the Efficacy of Warm Needling plus Penetrative Needling in Treating Refractory Facial Paralysis. SHANGHAI JOURNAL OF ACUPUNCTURE AND MOXIBUSTION. 29:287-288.

[571] 毛爱民. (2010)背俞穴挑治配合针刺治疗冠心病室性心律失常疗效观察.上海针灸杂志29:294-295.

[571] Mao Ai-min. (2010)Observations on the Efficacy of Piercing Back-Shu Points plus Acupuncture in Treating Ventricular Arrhythmia in Coronary Heart Disease. SHANGHAI JOURNAL OF ACUPUNCTURE AND MOXIBUSTION. 29:294-295.

[572] 徐凯,陈秀玲,黄云声. (2010)电针围刺治疗颞下颌关节功能紊乱综合征疗效观察.上海针灸杂志29:303-304.

[572] Xu Kai, Chen Xiu-ling, Hung Yun-sheng. (2010)Observations on the Therapeutic Effect of Surrounding Electroacupuncture on Temporomandibular Joint Disturbance Syndrome. SHANGHAI JOURNAL OF ACUPUNCTURE AND MOXIBUSTION. 29:303-304.

[573] 关鑫,高建东,王琛,余安胜,何立群.（2010)温针联合氯沙坦钾治疗慢性肾炎临床观察.上海针灸杂志,2010,29:347-349.

[573] Guan Xin, Gao Jian-dong, Wang Chen, Yu An-sheng, He Li-qun. (2010)Clinical Observations on the Combined Treatment of Chronic Nephritis with Warm Needling and Losartan Potassium. SHANGHAI JOURNAL OF ACUPUNCTURE AND MOXIBUSTION. 29:347-349.

[574] 孙远征,罗梅,牛雪茹. (2010)针刺募穴预防中风后患者便秘临床观察.上海针灸杂志29:352-353.

[574] Sun Yuan-zheng, Luo Mei, Niu Xue-ru. (2010)Clinical Observations on Acupuncture at Front-Mu Points for Preventing Constipation in Post-stroke Patients. SHANGHAI JOURNAL OF ACUPUNCTURE AND MOXIBUSTION. 29:352-353.

[575] 金泽,王琳晶. (2010)针刺夹脊穴治疗中风偏瘫痉挛状态临床观察.上海针灸杂志29:362-363.

[575] Jin Ze, Wang Lin-jing. (2010)

Clinical Observations on Acupuncture at Huatuojiaji Points for the Treatment of Post-stroke Hemiplegic Spasticity. SHANGHAI JOURNAL OF ACUPUNCTURE AND MOXIBUSTION. 29:362-363.

[576] 倪欢欢,崔晓,胡永善,冯美兰,周翠侠,等. (2010)浮刺结合功能训练治疗肩手综合征疗效观察.上海针灸杂志29367-368.

[576] Ni Huan-huan, Cui Xiao, Hu Yong-shan, Feng Mei-lan, Zhou Cui-xia et.al. (2010)Clinical Observations on the Efficacy of Superficial Needling plus Functional Training in Treating Shoulder-hand Syndrome. SHANGHAI JOURNAL OF ACUPUNCTURE AND MOXIBUSTION. 29367-368.

[577] 王金玲,白田雨,王欣. (2010) 电针配合康复训练治疗脊髓损伤疗效观察.上海针灸杂志29:369-371.

[577] Wang Jin-ling, Bai Tian-yu, Wang Xin. (2010)Observations on the Efficacy of Electroacupuncture plus Rehabilitation Training in Treating Spinal Cord Injury. SHANGHAI JOURNAL OF ACUPUNCTURE AND MOXIBUSTION. 29:369-371.

[578] 曹莲瑛,张伟,沈特立,李艳.(2010)不同频率电针治疗面肌痉挛临床疗效观察.上海针灸杂志29:372-374.

[578] Cao Lian-ying, Zhang Wei, Shen Te-li, Li Yan. (2010)Observations on the Clinical Efficacy of Different Frequency Electroacupuncture in Treating Facial Spasm. SHANGHAI JOURNAL OF ACUPUNCTURE AND MOXIBUSTION. 29:372-374.

[579] 王剑波,王延琳,卢永屹,吴耀持.(2010)温针透刺治疗老年膝骨关节炎疗效分析.上海针灸杂志29:390-392.

[579] Wang Jian-bo, Wang Yan-lin, Lu Yong-yi, Wu Yao-chi. (2010)Analysis of the Therapeutic Effect of Warm Point-to-point Needling on Senile Knee Osteoarthritis. SHANGHAI JOURNAL OF ACUPUNCTURE AND MOXIBUSTION. 29:390-392.

[580] 彭慧渊,何希俊,赵明华. (2010) 电针对脑卒中后抑郁患者日常生活活动能力及生存质量影响.上海针灸杂志29:425-427.

[580] Peng Hui-yuan, He Xi-jun, Zhao Ming-hua. (2010)Effect of Electroacupuncture at Temporal Three Needle Acupoints as Main Treatment on Activities of Daily Living and Quality of Life in Patients with Post-stroke Depression. SHANGHAI JOURNAL OF ACUPUNCTURE AND MOXIBUSTION. 29:425-427.

[581]王伟华,杨沈秋,顾明全. (2010)针刺配合隔物灸治疗中风后尿失禁疗效观察.上海针灸杂志29:433-435.

[581] Wang Wei-hua, Yang Shen-qiu, Gu Ming-quan. (2010)Observations on the Efficacy of Acupuncture plus Indirect Moxibustion in Treating Post-stroke Urinary Incontinence. SHANGHAI JOURNAL OF ACUPUNCTURE AND MOXIBUSTION. 29:433-435.

[582]赵颖.(2010)头体针结合治疗缺血性脑卒中后便秘疗效观察.上海针灸杂志,29:436-438.

[582] Zhao Ying. (2010)Observations on the Efficacy of Combined Scalp and Body Acupuncture in Treating Post Ischemic Stroke Constipation. SHANGHAI JOURNAL OF ACUPUNCTURE AND MOXIBUSTION. 29:436-438.

[583]汪军,孙克兴,吴绪波. (2010)头针治疗对脑瘫儿童粗大运动功能的影响.上海针灸杂志29:442-445.

[583] Wang Jun, Sun Ke-xing, Wu Xu-bo. (2010)Clinical Observations on the Effect of Scalp Acupuncture on Gross Motor Function in Cerebral Palsy Children. SHANGHAI JOURNAL OF ACUPUNCTURE AND MOXIBUSTION. 29:442-445.

[584]覃彪民,谷婷婷.(2010)电针加悬灸百会治疗颈性眩晕疗效观察.上海针灸杂志,29:454-455.

[584] Qin Biao-min, Gu Ting-ting. (2010)Observations on the Efficacy of Electroacupuncture plus Suspended Moxibustion over Baihui in Treating Cervical Vertigo. SHANGHAI JOURNAL OF ACUPUNCTURE AND MOXIBUSTION. 29:454-455.

[585]巫祖强,刘吉昌,粟漩. (2010)针刺配合药物直流电导入治疗三叉神经痛临床观察.上海针灸杂志29:509-510.

[585] Wu Zu-qiang, Liu Ji-chang, Shu Xuan. (2010)Clinical Observations on Acupuncture plus Direct Current Drug Introduction for the Treatment of Trigeminal Neuralgia. SHANGHAI JOURNAL OF ACUPUNCTURE AND MOXIBUSTION. 29:509-510.

[586]任莉赟,马向明. (2010)针刺配合隔姜灸治疗原发性痛经疗效观察.上海针灸杂志29:517-518.

[586] Ren Li-yun, Ma Xiang-ming. (2010)Observations on the Efficacy of Acupuncture plus Ginger Moxibustion in Treating Primary Dysmenorrhea. SHANGHAI JOURNAL OF ACUPUNCTURE AND MOXIBUSTION. 29:517-518.

[587]陈晓洁,汪永坚,严红妹. (2010)穴位温灸法治疗妇科术后尿潴留疗效观察.上海针灸杂志29:519-520.

[587] Chen Xiao-jie, Wang Yong-jian, Yan Hong-mei. (2010)Observations on the Therapeutic Effect of Acupoint Mild Moxibustion on Gynaecologic Postoperative Uroschesis. SHANGHAI JOURNAL OF ACUPUNCTURE AND MOXIBUSTION. 29:519-520.

[588]曾顺军,刘丽,关德斌. (2010)颈椎横突穴位注射治疗神经根型颈椎病疗效观察.上海针灸杂志29:523-524.

[588] Zeng Shun-jun, Liu Li, Guan De-bin. (2010)Analysis of Cervical Vertebral Transverse Process Point Injection for the Treatment of Cervical Spondylotic Radiculopathy. SHANGHAI JOURNAL OF ACUPUNCTURE AND MOXIBUSTION. 29:523-524.

[589]徐世芬,庄礼兴.(2010)电针百会、印堂为主治疗抑郁症疗效观察.上海针灸杂志29:569-572.

[589] Xu Shi-fen, Zhuang Li-xing. (2010)Observations on the Efficacy of Electroacupuncture at Baihui and Yintang as Main Treatment for Depression. SHANGHAI JOURNAL OF ACUPUNCTURE AND MOXIBUSTION. 29:569-572.

[590]张洪,邓鸿. (2010)针刺配合穴位注射治疗地震后广泛性焦虑症疗效观察.上海针灸杂志29:576-577.

[590] Zhang Hong, Deng Hong. (2010)Observations on the Efficacy of Acupuncture plus Acupuncture Point Injection in Treating Post-earthquake Generalized Anxiety Disorder. SHANGHAI JOURNAL OF ACUPUNCTURE AND MOXIBUSTION. 29:576-577.

[591]丁敏,蒋亚秋,林天云. (2010)穴位埋线治疗顽固性面瘫疗效观察.上海针灸杂志29:578-579.

[591] Ding Min, Jiang Ya-qiu, Lin Tian-yun. (2010)Observations on the Efficacy of Acupoint Catgut Embedding in Treating Refractory Facial Paralysis. SHANGHAI JOURNAL OF ACUPUNCTURE AND MOXIBUSTION. 29:578-579.

[592]赵义造.(2010)针刺不同留针时间对面神经麻痹疗效观察.上海针灸杂志29:580-581.

[592] Zhao Yi-zao. (2010)Observations on the Effects of Acupuncture in Different Needle Retention Times in the Treatment of Facial Paralysis. SHANGHAI JOURNAL OF ACUPUNCTURE AND MOXIBUSTION. 29:580-581.

[593]黄文学.(2010)针刺董氏奇穴为主治疗三叉神经痛疗效观察.上海针灸杂志29:582-583.

[593] Huang Wen-xue. (2010)Observations on the Efficacy of Acupuncture at Dong's Extraordinary Points as Main Treatment for Trigeminal Neuralgia. SHANGHAI JOURNAL OF ACUPUNCTURE AND MOXIBUSTION. 29:582-583.

[594]杜海英,俞兴源. (2010)眼轮匝肌排针刺法治疗特发性眼睑痉挛疗效观察.上海针灸杂志29:584-585.

[594] Du Hai-ying, Yu Xing-yuan. (2010)Observations on the Efficacy of Orbicularis Oculi-aligned Acupuncture in Treating Idiopathic Blepharospasm. SHANGHAI JOURNAL OF ACUPUNCTURE AND MOXIBUSTION. 29:584-585.

[595]董有康,姜云武,王春林,郭村晓,李应华.(2010)针刺治疗肝肾不足椎动脉型颈椎病疗效观察.上海针灸杂志29:586-588.

[595] Dong You-kang, Jiang Yun-wu, Wang Chun-lin, Guo Cun-xiao, Li Ying-hua. (2010)Observations on the Efficacy of Acupuncture Based on Point Selection in Treating Cervical Spondylotic Vertebral Arteriopathy with Liver-kidney Deficiency. SHANGHAI JOURNAL OF ACUPUNCTURE AND MOXIBUSTION. 29:586-588.

[596]吴高飞,宣丽华,吴翔. (2010)腹针治疗腰椎间盘突出症疗效观察.上海针灸杂志29:591-592.

[596] Wu Gao-fei, Xuan Li-hua, Wu Xiang. (2010)Observations on the Therapeutic Effect of Abdominal Acupuncture on Lumbar Intervertebral disc Herniation. SHANGHAI JOURNAL OF ACUPUNCTURE AND MOXIBUSTION. 29:591-592.

[597]梁雪英,林震,林光. (1992)隔姜灸治疗阳萎临床观察.针刺研究4:263-265.

[597] Liang Xue-ying, Lin Zhen, Lin Guang. (1992)Clinical observation of ginger partition moxibustion treatment of impotence. Acupuncture Research. 4:263-265.

[598]梁雪英,林震,林光. (1992)隔姜灸对阳萎症的治疗作用.针刺研究4:303.

[598] Liang Xue-ying, Lin Zhen, Lin Guang. (1992)The treatment of moxibustion with ginger on the impotence. Acupuncture Research. 4:303.

[599]周杰芳,靳瑞. (2004)不同电针强度对高血压实证病人血压的影响.针刺研究29:286-295.

[599] Zhou Jie-fang, Jin Rui. (2004)Influence of Different Intensities of Electroacupuncture on Blood Pressure in Excess-syndrome Type Hypertension Patients. Acupunture Research. 29:286-295.

[600]高宏,李雪珍,叶文伟,周柏英,金英杰,等. (2009)粗针皮下透刺神道穴对慢性荨麻疹患者症状积分及血清IgE的影响.针刺研究34:272-275.

[600] Gao Hong, Li Xue-zhen, Ye Wen-wei, Zhou Bai-ying, Jin Ying-jie et.al. (2009)Influence of Penetrative Needling of Shendao(GV 11)on the Symptom Score and Serum IgE Content in Chronic Urticaria Patients. Acupunture Research. 34:272-275.

[601]唐强,张立,魏铁花,王艳,白晶,等.(2009)电鍉丛针治疗脑梗死患者运动功能障碍的随机对照疗效观察.针刺研究34:193-197.

[601] Tang Qiang, Zhang Li, Wei Tie-hua, Wang Yan, Bai Jing et.al. (2009)Randomized controlled observation curative effect of electro spoon needle cluster motion in patients with dysfunction of the treatment of cerebral infarction. Acupunture Research. 34:193-197.

[602]职良喜.(2007)电浮针对原发性痛经镇痛作用的随机对照研究.针刺研究32:342-346.

[602] Zhi Liang-xi. (2007)Randomized Controlled Study on the Analgesic Effect of Superficial Needling plus Electrostimulation of Sanyinjiao (SP 6) for Primary Dysmenorrhea. Acupunture Research. 32:342-346.

[603]唐育民,魏湘华,尹志礼,郝冰.(2001)电针对安氟醚最低肺泡有效浓度的影响.针刺研究26:172.

[603] Tang Yu-min, Wei Xiang-hua, Yin Zhi-li, Hao Bing. (2001)Effect of Electroacupuncture on Minimal Alveolar Concentration of Enflurane. Acupunture Research. 26:172.

[604]倪育飞,李军,王本福,蒋松鹤,陈奕,等.(2009)电针对肠镜检查患者脑电双频指数和β-内啡肽的影响.针刺研究34:339-343.

[604] Ni Yu-fei, Li Jun, Wang Ben-fu, Jiang Song-he, Chen Yi et.al. (2009)Effects of Electroacupuncture on Bispectral Index and Plasma β-endorphin in Patients Undergoing COlonoscopy. Acupunture Research. 34:339-343.

[605]罗华丽,李荣亨.(2007)电针对单纯性肥胖瘦素及脂联素的影响.针刺研究32:264-267.

[605] Luo Hua-li, Li Rong-heng. (2007)Effect of Electroacupuncture on Leptin and Adiponectin in Simple Obesity Patients. Acupunture Research. 32:264-267.

[606]李璇,张红星,黄国付,冯永芳,邹燃,等. (2009)电针夹脊配合围刺治疗带状疱疹疗效的随机对照观察.针刺研究34:125-135.

[606] Li Xuan, Zhang Hong-xing, Huang Guo-fu, Feng Yong-fang, Zou Ran et.al. (2009)Observation on the Therapeutic Effect of Electroacupuncture of Jiaji(EX-B 2)plus Regional En circled Needling for Herpes Zoster. Acupunture Research. 34:125-135.

[607]周媛,周国赢,李少坤,靳建宏.(2010)电针结合背腰部拔罐治疗中风后疲劳64例.针刺研究35:380-383.

[607] Zhou Yuan, Zhou Guo-ying, Li Shao-kun, Jin Jian-hong. (2010)Clinical Observation on the Therapeutic Effect of Electroacupuncture Combined with Cupping on Post-stroke Fatigue. Acupunture Research. 35:380-383.

[608]王成伟,李宁,何洪波,吕建琴,刘志顺.(2010)电针双侧天枢穴对功能性便秘患者自觉症状的影响及疗效满意度评价一项单中心、前瞻性随机对照临床试验.针刺研究35:375-379.

[608] Wang Cheng-wei, Li Ning, He Hong-bo, Lv Jian-qin, Liu Zhi-shun. (2010)Effect of Electroacupuncture of Tianshu(ST 25)on the Rational Symptoms of Functional Constipation Patients and Evaluation on Its Efficacy Satisfaction:A Single-center,Prospective,Practical and Randomized Control Trial. Acupunture Research. 35:375-379.

[609]金亚蓓,孙占玲,金慧芳. (2009)耳穴电针治疗子宫内膜异位症痛经的随机对照研究.针刺研究34:188-192.

[609] Jin Ya-bei, Sun Zhan-ling, Jin Hui-fang. (2009)Randomized Controlled Study on Ear-electroacupuncture Treatment of Endo-metriosis-induced Dysmenorrhea in Patients. Acupunture Research. 34:188-192.

[610]张蔚婷,金真,罗非,张磊,崔国红,等. (2001)经皮穴位电刺激诱发的大脑fMRI信号与镇痛的关系.针刺研究26:193-194.

[610] Zhang Wei-ting, Jin Zhen, Luo Fei, Zhang Lei, Cui Guo -hong et.al. (2001)Correlation between Acupoint-stimulation Evoked Cerebral Functional Magnetic Resonance Signals and Analgesic Effect. Acupunture Research. 6:193-194.

[611]余建明,曲丕盛,范皓,王振,金亚蓓,等. (2010)经皮穴位电刺激在乳腺癌根治手术中镇痛效应的研究.针刺研究35:43-46.

[611] Yu Jian-ming, Qu Pi-sheng, Fan Hao, Wang Zhen, Jin Ya-bei et.al. (2010)Observation on the Analgesic Effect of Transcutaneous Electrical Acupoint Stimulation for Breast Radical Carcinoma Operation. Acupunture Research. 35:43-46.

[612]张宁霞,刘桂珍,孙克兴,郝晋东.(2007)热补针法结合康复训练对小儿脑瘫患儿功能影响的随机对照研究.针刺研究32:260-263.

[612] Zhang Ning-xia, Liu Gui-zhen, Sun Ke-xing, Hao Jin-dong. (2007)Clinical Study on the Treatment of Infant Cerebral Palsy with Warm-reinforcing Needling Combined with Rehabilitation Training. Acupunture Research. 32:260-263.

[613]张仁惠.(1989)三种刺激方法在623例针麻甲状腺手术中的应用.针刺研究1-2:214-215.

[613] Zhang Ren-hui. (1989)Application of three kinds of stimulation methods in 623 cases of thyroid operation under acupuncture anesthesia. Acupunture Research. 1-2:214-215.

[614]焦焰. (1991)四肢扭伤针刺镇痛的观察.针刺研究Z1:253-254.

[614] Jiao yan. (1991)Observation of limb sprain of acupuncture analgesia. Acupunture Research. 1:253-254.

[615]周炜,刘泓,王丽平,许志刚,冯勇伟,等. (2009)头体针与腹针交替应用治疗中风后上肢功能障碍疗效.针刺研究34:128-131.

[615] Zhou Wei, Liu Hong, Wang Li-ping, Xu Zhi-gang, Feng Yong-wei et.al. (2009)Effect of Alternative Administration of Scalp-acupuncture, Body-acupuncture and Abdominal acupuncture on the Upper-limb Dyscinesia in Patients with Stroke. Acupunture Research. 34:128-131.

[616]张宏荣. (2002)穴位注射配合牵引治疗颈椎病疗效观察.针刺研究27:287-290.

[616] Zhang Hong-rong. (2002)Observation on the Therapeutic Effect of Acupoint-injection Combined with Traction for Treatment of Cervical Spondylopathy. Acupunture Research. 27:287-290.

[617]吴家萍. (2010)针刺辨证治疗中风后抑郁症150例临床观察.针刺研究35:303-306.

[617] Wu Jia -ping. (2010)Clinical Observation on Acupuncture Treatment of 150 Cases of Post-stroke Depression According to Syndrom Differentiation. Acupunture Research. 35:303-306.

[618]谌剑飞. (2001)针刺对Ⅱ型糖尿病患者血清生长激素的影响.针刺研究26:310-313.

[618] Shen Jian-fei. (2001)Influence of Acupuncture on Serum Growth Hormone Level in Patients with Type Ⅱ Diabetes. Acupunture Research. 26:310-313.

[619]时杰,王秋,时岩. (2003)针刺对老年性白内障患者房水中的离子元素及免疫系统的影响.针刺研究28:62-65.

[619] Shi Jie, Wang Qiu, Shi Yan. (2003)Effect of Acupuncture on the Changes of Ion Contents of Aqueous Humor and the Immune System in Senile Cataract Patients. Acupunture Research. 28:62-65.

[620]王京京,宋玉静,吴中朝,初笑欧,王巧妹,等.(2009)针刺对慢性疲劳综合征患者疲劳程度影响的随机对照观察.针刺研究34:120-124.

[620] Wang Jing-jing, Song Yu-jing, Wu Zhong-chao, Chu Xiao-ou, Wang Qiao-mei et.al. (2009)Randomized Controlled Clinical Trials of Acupuncture Treatment of Chronic Fatigue Syndrome. Acupunture Research. 34:120-124.

[621]褚芹, 王琳.(2007)针刺对糖尿病肾病血液流变学影响的临床随机对照研究.针刺研究32:335-337.

[621]Chu Qin, Wang Lin.(2007) Effect of Acupuncture on Hemorheology in Patients with Diabetic Nephropathy. Acupuncture Research ＆ Orthopedics 32:335-337.

[622]王祥瑞, 卢中平, 许灿然, [杭燕南](http://202.201.7.11/kcms/detail/search.aspx?dbcode=CJFQ&sfield=au&skey=杭燕南&code=05967811;05966052;05965529;23057203;22835167;), [孙大金](http://202.201.7.11/kcms/detail/search.aspx?dbcode=CJFQ&sfield=au&skey=孙大金&code=05967811;05966052;05965529;23057203;22835167;).(2001)针刺对心脏手术病人的机体保护作用.针刺研究26:173-174.

[622]Wang Xiang-rui, Lu Zhong-ping, Xu Can-ran, Hang Yan-nan, Sun Da-jin.(2001).

The Protective Effects of Electroacupuncture on Patients Undergoing Cardiac Surgery. Acupuncture Research ＆ Orthopedics 26:173-174.

[623]秦必光, 任业川, 张兰英, [白占勇](http://202.201.7.11/kcms/detail/search.aspx?dbcode=CJFQ&sfield=au&skey=白占勇&code=05980555;06494470;06494138;06494658;08795600;08795611;08811157;08811153;), [胡北喜](http://202.201.7.11/kcms/detail/search.aspx?dbcode=CJFQ&sfield=au&skey=胡北喜&code=05980555;06494470;06494138;06494658;08795600;08795611;08811157;08811153;), 等. (2001)针刺复合小剂量硬膜外麻醉胆囊切除临床研究总结.针刺研究26:143-149.

[623]Qin Bi-guang, Ren Ye-chuan, Zhang Lan-ying, Bai Zhan-yong, Hu Bei-xi, et al.(2001) Summary of Clinical Research on Acupuncture Combined with Epidural Anesthesia for Cholecystectomy. Acupuncture Research ＆ Orthopedics 26:143-149.

[624]张兰英, 彭莉, 秦必光. (2001)针刺复合硬膜外麻醉胆囊切除术胆心反射发生率探讨.针刺研究26:156-160.

[624]Zhang Lan-ying, Peng Li, Qin Bi-guang.(2001)Incidence Rate of Gallbladder-cardiac Reflex during Cholecystectomy under Acupuncture Combined with Epidural Administration of Small Dose of Anesthetic. Acupuncture Research ＆ Orthopedics 26:156-160.

[625]李立志, 孙频声.(1991)针刺复合硬膜外麻醉术中效果及后效应.针刺研究Z1:276-277.

[625]Li Li-zhi, Sun Pin-sheng.(1991)Effects of Acupuncture combined with Epidural Block Observed during and after Appendectomy.Acupuncture Research ＆ Orthopedics Z1:276-277.

[626]张安仁, 罗绯, 潘志伟, [周永杰](http://202.201.7.11/kcms/detail/search.aspx?dbcode=CJFQ&sfield=au&skey=周永杰&code=20314499;20169226;20849007;21079224;).(1996)针刺后溪神、门对脑外伤性痴呆的影响.针刺研究21:12-14.

[626]Zhang An-ren, Luo Fei, Pan Zhi-wei, （1996） Zhou Yong-jie.(1996) Influence of Cerebral Traumatic Dementia Treated with Acupuncture at Houxi and shenmen. Acupuncture Research ＆ Orthopedics 21:12-14.

[627]梁绿茵.(2002)针刺加葱盐外敷灸治疗产后尿潴留43例临床观察.针刺研究27:292-294.

[627]Liang Lv-yin.(2002) Clinical Observation on Treatment of 43 Cases of Postpartum Uroschesis with Acupuncture plus Scallion-salt Separated Moxibustion. Acupuncture Research ＆ Orthopedics 27:292-294.

[628]王曙辉, 许明珠, 崔韶阳, [郭元琦](http://202.201.7.11/kcms/detail/search.aspx?dbcode=CJFQ&sfield=au&skey=郭元琦&code=23761375;23761376;10744330;23761377;).(2010)针刺结合刺络放血疗法治疗膝关节骨性关节炎的临床随机对照研究.针刺研究35:129-133.

[628]Wang Shu-hui, Xu Ming-zhu, Cui Shao-yang, Guo Yuan-qi.(2010)Randomized Controlled Clinical Trails on Treatment of Knee Osteoarthritis with Acupuncture Combined with Blood-letting Therapy.Acupuncture Research ＆ Orthopedics 35:129-133.

[629]薛正海. (1998)针刺经穴与全息穴治疗遗尿54例.针刺研究3:229-230.

[629]Xue Zheng-hai.(1998)Treatment of Ennresis by Acupuncture at Acupoints and Holographic Points. Acupuncture Research ＆ Orthopedics 3:229-230.

[630]曹玲, 侯广云, 范文双. (2007)针刺颈部夹脊穴治疗颈源性高血压的疗效分析.针刺研究32:195-198.

[630]Cao Ling, Hou Guang-yun, Fan Wen-shuang.(2007)Analysis on the Therapeutic Effects of Acupuncture of Jiaji (EX-B 2) for Treatment of Cervical Hypertension. Acupuncture Research ＆ Orthopedics 32:195-198.

[631]常庚申, 马克昌.(2001)针刺内麻点超前镇痛在四肢骨科手术中的应用.针刺研究26:181-182.

[631]Chang Geng-shen, Ma Ke-chang.(2001)Application of Needling“Neimadian-point” in Advance to Orthopedic Surgery of Extremities.Acupuncture Research ＆ Orthopedics 26:181-182.

[632]潘锋, 陈建永, 詹程胹. (2006)针刺配合生物反馈治疗出口梗阻型便秘的临床观察.针刺研究31:294-297.

[632]Pan Feng, Chen Jian-yong, Zhan Cheng-er.(2006)Clinical Observation on the Treatment of Outlet Obstruction Type Constipation by Acupuncture Combined with Biofeedback Method. Acupuncture Research ＆ Orthopedics 31:294-297.

[633]刘累耕, 刘岚, 吕鸣, [王超](http://202.201.7.11/kcms/detail/search.aspx?dbcode=CJFQ&sfield=au&skey=王超&code=09833217;08779932;09833216;09833223;).(2001)针刺配合药物治疗格林巴利综合征.针刺研究26:183-184.

[633]Liu Lei-geng, Liu Lan, Lv Ming, Wang Chao.(2001)Combined Treatment of 68 Cases of Multiple Radiculoneuritis. Acupuncture Research ＆ Orthopedics 26:183-184.

[634]睢明河.(2001)针刺手少阳经穴治疗中风后上臂痉挛的临床观察.针刺研究26:131-133.

[634]Sui Ming-he.(2001)Clinical Observation on the Therapeutic Effect of Acupuncture of Hand-Shaoyung Meridian Acupoints for Treatment of Upper-limb Spasm in Stroke Patients.

Acupuncture Research ＆ Orthopedics 26:131-133.

[635]柏亚萍, 傅杰英.(2007)针刺胃经经穴治疗胃肠实热型肥胖的临床观察.针刺研究, 32:128-131.

[635]Bai Ya-ping, Fu Jie-ying.(2007)Clinical Observation on the Regularity of Acupuncture-induced Body-reduction in Excess-heat-type 0besity Patients.Acupuncture Research ＆ Orthopedics 32:128-131.

[636]尹淑英, 魏守顺, 刘晓翎. (2004)针刺与度冷丁对121例输尿管结石绞痛患者镇痛和排石作用的临床分析.针刺研究29:66-68.

[636]Yin Shu-ying, Wei Shou-shun, Liu Xiao-ling.(2004)Clinical Analysis on the Therapeutic Effect of Electroacupuncture for Treatment of 121 Cases of Ureterolith with Colic. Acupuncture Research ＆ Orthopedics 29:66-68.

[637]吴滨, 周荣兴, 陈名金, [屈云](http://202.201.7.11/kcms/detail/search.aspx?dbcode=CJFQ&sfield=au&skey=屈云&code=21435509;07593901;20647477;17637809;07584371;07584902;21214074;07586000;21617689;07593956;), [张永玲](http://202.201.7.11/kcms/detail/search.aspx?dbcode=CJFQ&sfield=au&skey=张永玲&code=21435509;07593901;20647477;17637809;07584371;07584902;21214074;07586000;21617689;07593956;), 等.(1995)针刺治疗对恶性肿瘤患者细胞免疫调节的影响.针刺研究20:67-71.

[637]Wu Bin, Zhou Rong-xing, Chen Ming-jin, Qu Yun, Zhang Yong-ling, et al.(1995) Effect of Acupuncture on the Regulation of Cell-mediated Immunity in the Patients with Malignant Tumors. Acupuncture Research ＆ Orthopedics 20:67-71.

[638]常佩芬. (2005)针刺治疗稳定性心绞痛30例体会.针刺研究30:50-52.

[638]Chang Pei-fen.(2005)My Experience about Acupuncture Treatment of 30 Cases of Stable Angina Pectoris. Acupuncture Research ＆ Orthopedics 30:50-52.

[639]唐胜修, 徐祖豪, 唐萍, [李凤萍](http://202.201.7.11/kcms/detail/search.aspx?dbcode=CJFQ&sfield=au&skey=李凤萍&code=06905305;06900191;22788313;06900192;). (2003)针刺治疗抑郁性神经症及对免疫功能的影响.针刺研究28:270-272.

[639]Tang Sheng-xiu, Xu Zu-hao, Tang Ping, Li Feng-Ping.(2003)Treatment of Depressive Psychosis with Acupuncture and Its Effect Oil Immunological Function. Acupuncture Research ＆ Orthopedics 28:270-272.

[640]李扬帆, 邓柏颖, 粟胜勇, [周恩华](http://202.201.7.11/kcms/detail/search.aspx?dbcode=CJFQ&sfield=au&skey=周恩华&code=06897739;06902547;06903809;06897659;).(2004)针灸对中风后偏瘫肩痛者血脂影响的观察.针刺研究29:289-292.

[640]Li Yang-fan, Deng Bai-ying, Su Sheng-yong, Zhou En-hua.(2004) Effect of Acupuncture and Moxibustion on Blood Lipid Level in Apoplectic Hemiplegia Patient with Omalgia. Acupuncture Research ＆ Orthopedics 29:289-292.

[641]吴家萍, 边朝辉. (2006)针灸和甲氨蝶呤治疗类风湿性关节炎90例临床疗效对比.针刺研究31:235-237.

[641]Wu Jia-ping, Bian Zhao-hui.(2006)Comparison Between Acu-moxibustion and Methotrexate in the Therapeutic Effect in the Treatment of 90 Cases of Rheumatoid Arthritis.Acupuncture Research ＆ Orthopedics 31:235-237.

[642]李艳慧, 杨文辉, 庄礼兴, [郑谅](http://202.201.7.11/kcms/detail/search.aspx?dbcode=CJFQ&sfield=au&skey=郑谅&code=06931332;06931282;06929497;06931319;).(1997)针灸为主治疗血管性痴呆的临床研究.针刺研究:259-262.

[642]Li Yan-hui, Yang Wen-hui, Zhuang Li-xing, Zheng Liang.(1997)Clinical Study on the Vascular Dementiala Treated with Acupuncture and Moxibustion.Acupuncture Research ＆ Orthopedics:259-262.

[643]刘世敏, 吴焕淦, 王拥军, [朱毅](http://202.201.7.11/kcms/detail/search.aspx?dbcode=CJFQ&sfield=au&skey=朱毅&code=08600204;05973964;08631941;09697361;).(2000)针灸治疗不同类型颈椎病的临床研究.针刺研究25:225-227.

[643]Liu Shi-min, Wu Huan-gan, Wang Yong-jun, Zhu Yi.(2000)Clinical Study of Acupuncture and Moxibustion Treatment on Cervical Spondylosis.Acupuncture Research ＆ Orthopedics25:225-227.

[644]雷磊. (1997)针灸治疗冠心病30例临床观察.针刺研究22:184

[644]Lei Lei.(1997)Clinical Study on Effects of Acupuncture and Moxibustion Treatment 30 Cases of Coronary Heart Disease. Acupuncture Research ＆ Orthopedics 22:184

[645]唐育民, 魏湘华, 尹志礼, [郝冰](http://202.201.7.11/kcms/detail/search.aspx?dbcode=CJFQ&sfield=au&skey=郝冰&code=20312375;21363216;21417732;21587754;).(2001)针麻开胸手术超前镇痛作用临床观察.针刺研究26:168-169.

[645]Tang Yu-min, Wei Xiang-hua, Yin Zhi-li, Hao Bing.(2001)Clinical Observation on Preemptive Analgesia of EIectroacupuncture for Patients Undergoing Thoracic Operation.Acupuncture Research ＆ Orthopedics 26:168-169.

[646]孙频声, 李立志, 侍明宏.(1992)针麻与硬膜外麻醉行阑尾切除术效果比较研究.针刺研究2:87-89.

[646]Sun Pin-sheng, Li Li-zhi, Si Ming-hong.(1992)Comparison Between of Acupuncture and Epidural Anesthesia in Appendectomy. Acupuncture Research ＆ Orthopedics 2:87-89.

[647]张毅敏.(2006)针药结合对先天愚型儿童智商和社会适应行为的影响.针刺研究31:166-168.

[647]Zhang Yi-min.(2006)Effect of Acupuncture Combined with Herbal Medicines on Intelligence Quotient and Adaptive Behavior Quotient in Infant Patients with Down’S Syndrome. Acupuncture Research ＆ Orthopedics31:166-168.

[648]吴连方.(2001)针药结合分娩镇痛的临床研究.针刺研究26:179-180.

[648]Wu Lian-fang.(2001)Application of Acupuncture Combined with Medicines for Pain Relief during Labor.Acupuncture Research ＆ Orthopedics 26:179-180.

[649]徐天舒, 钱海华.(2009)针药结合麻醉下手术治疗混合痔40例临床观察.针刺研究34:403-405.

[649]Xu Tian-shu, Qian Hai-hua.(2009) Clinical Observation on the Effect of Acupuncture-assisted Anesthesia for 40 Cases of Mixed Hemorrhoids Surgery.Acupuncture Research ＆ Orthopedics 34:403-405.

[650]徐建钟,刘家英,张永刚,[张丹敏](http://202.201.7.11/kcms/detail/search.aspx?dbcode=CJFQ&sfield=au&skey=张丹敏&code=10042341;10042315;12260699;06371794;21593936;09971143;),[李艳华](http://202.201.7.11/kcms/detail/search.aspx?dbcode=CJFQ&sfield=au&skey=李艳华&code=10042341;10042315;12260699;06371794;21593936;09971143;), 等.(2001)针药结合治疗坐骨神经痛.针刺研究26:187-189.

[650]Xu Jian-zhong, Liu Jia-ying, Zhang Yong-gang, Zhang Dan-min, Li Yan-hua, et al.(2001)Electroacupuncture Combined with Medicines for Treatment of Sciatica. Acupuncture Research ＆ Orthopedics 26:187-189.

[651]李艳慧, 靳瑞. (1994)针刺颞穴为主治疗脑血管意外后遗症的临床研究.针刺研究19:4-7.

[652]Li Yan-hui, Jin Rui.(1994)Clinical Study on the Sequelae of Cerebral Vascular Accident Treated with Temporal-point Acupuncture.Acupuncture Research ＆ Orthopedics 19:4-7.

[652]周建伟, 张凡. (1997)阿是穴四花刺法治疗痛证的临床治疗观察.针刺研究22:103.

[652]Zhou Jian-wei，Zhang Fan.(1997)Clinical Observation on Ashi points Sihua needling Method for Treatment of Acute Pains.Acupuncture Research ＆ Orthopedics 22:103.

[653]罗庆道, 罗建明. (1997)电针背俞穴对急性胃脘痛的镇痛观察.针刺研究22:138.

[653]Luo Qing-dao, Luo Jian-ming.(1997)Clinical Observation on Acute Stomachache Treated by Electro-needle on Back-shu Points. Acupuncture Research ＆ Orthopedics 22:138.

[654]唐辉毅, 梁伟民, 俞莹芳, [潘银英](http://202.201.7.11/kcms/detail/search.aspx?dbcode=CJFQ&sfield=au&skey=潘银英&code=08617438;09688601;05965123;09687645;08640400;08649509;08634327;08639932;05964135;), [徐振邦](http://202.201.7.11/kcms/detail/search.aspx?dbcode=CJFQ&sfield=au&skey=徐振邦&code=08617438;09688601;05965123;09687645;08640400;08649509;08634327;08639932;05964135;), 等.(1997)吗啡和电针对术后病人淋巴细胞增殖反应的影响.针刺研究22:143.

[654]Tang Hui-yi, Liang Wei-min, Yu Ying-fang, Pan Yin-ying, Xu Zhen-bang, et al.(1997)Effect of Epidural Morphine and Electroacupuncture (EA) on Lymphocyte Proliferation of Postoperative Analgesia Patients.Acupuncture Research ＆ Orthopedics 22:143.

[655]刘颖涛, 秦必光, 程希熊, [张兰英](http://202.201.7.11/kcms/detail/search.aspx?dbcode=CJFQ&sfield=au&skey=张兰英&code=05980555;06494470;13986578;08829078;).(1997)针刺复合小剂量硬膜外麻醉胃大部切除术对循环功能的影响.针刺研究22:125-126.

[655]Liu Ying-tao, Qin Bi-guang, Cheng Xi-xiong, Zhang Lan-ying.(1997)Effect of Compound Acupuncture and Small Dose Peridural Anesthesia on Circulatory Function in Subtotal Gastrectomy..Acupuncture Research ＆ Orthopedics 22:125-126.

[656]秦必光, 程希熊, 彭其芳, [张兰英](http://202.201.7.11/kcms/detail/search.aspx?dbcode=CJFQ&sfield=au&skey=张兰英&code=06494138;08829078;08813857;05980555;14850811;08814673;06494470;), [徐永健](http://202.201.7.11/kcms/detail/search.aspx?dbcode=CJFQ&sfield=au&skey=徐永健&code=06494138;08829078;08813857;05980555;14850811;08814673;06494470;).(1997)针刺复合小剂量硬膜外麻醉胃大部切除研究小结.针刺研究Z1:130.

[656]Qin Bi-guang, Cheng Xi-xiong, Peng Qi-fang, Zhang Lan-ying, Xu Yong-jian.(1997)Study on Subtotal Gastrectomy under Compound Acupuncture and Small Dosage Peridural Anesthesia.

Acupuncture Research ＆ Orthopedics Z1:130.

[657]秦必光, 张兰英.(1997)针刺麻醉对硬膜外麻醉阻滞范围的探讨.针刺研究1-2:131-132.

[657]Qin Bi-guang, Zhang Lan-ying.(1997)Study on Effect of Acupuncture Anesthesia on Bloking Range of Peridural Anesthesia.Acupuncture Research ＆ Orthopedics 1-2:131-132.

[658]方向明, 刘维洲, 肖永俭. (1997)艾灸治疗溃疡性结肠炎58例疗效观察.针刺研究22:196.

[658]Fang Xiang-ming, Liu Wei-zhou, Xiao Yong-jian.(1997) The Observation on the Curative Effect of Ulcerative Colitis by Treatment of Moxibustion. Acupuncture Research ＆ Orthopedics 22:196.

[659]樊松龄.(1997)针刺加增温灸治疗腰腿痛疗效分析.针刺研究22:226.

[659]Fan Song-ling.(1997) Forty-two Cases of Stubborn Sciatica Treated by Material-separated Moxibustion.Acupuncture Research ＆ Orthopedics 22:226.

[660]屈桂莲, 庄心良, 徐国辉, [杨柳芬](http://202.201.7.11/kcms/detail/search.aspx?dbcode=CJFQ&sfield=au&skey=杨柳芬&code=09637208;09637983;09636370;08595759;08595577;09638005;05965775;00008445;08561503;), [王珍娣](http://202.201.7.11/kcms/detail/search.aspx?dbcode=CJFQ&sfield=au&skey=王珍娣&code=09637208;09637983;09636370;08595759;08595577;09638005;05965775;00008445;08561503;), 等.(1997)针药复合麻醉用于肾移植术的临床研究.针刺研究22:275-279.

[660]Qu Gui-lian, Zhuang Xin-liang, Xu Guo-hui, Yang Liu-fen, Wang Zhen-di, et al.(1997)

Combination of Acupuncture Analgesia and Eoidural Block in Renal Transplantation.

Acupuncture Research ＆ Orthopedics22:275-279.

[661]李金波.(1998)针刺对急性脑血管病继发肺水肿患者PA-aO2的影响.针刺研究23:281-282.

[661]Li Jin-bo.(1998)Influence of Acupuncture on PA-aO_2_ of Patients with Secondary Pneumonedema after Acute Cerebrovascular Disease. Acupuncture Research ＆ Orthopedics23:281-282.

[662]阮经文, 郑沛仪, 温明, [饶中东](http://202.201.7.11/kcms/detail/search.aspx?dbcode=CJFQ&sfield=au&skey=饶中东&code=10182998;10085492;10085484;10183189;).(1999)针刺对中风早期患者认知功能影响的临床观察.针刺研究24:223-226.

[662]Ruan Jing-wen, Zheng Pei-yi, Wen-ming, Rao Zhong-dong.(1999)Clinical Observation on the Effects of Acupuncture on the Cognition of the Early Stroke Patients. Acupuncture Research ＆ Orthopedics 24:223-226.

[663]王升旭, 赖新生, 李树成, [老锦雄](http://202.201.7.11/kcms/detail/search.aspx?dbcode=CJFQ&sfield=au&skey=老锦雄&code=20239941;05966168;06767306;06766295;).(1999)电针夹脊穴治疗颈椎病的临床研究.针刺研究24:227-230.

[663]Wang Sheng-xu, Lai Xin-sheng, Li Shu-cheng, Lao Jin-xiong.(1999)The Clinical Research of Electric Acupuncture at Jiaji Acupoints to Treat Cervical Spondylosis.Acupuncture Research ＆ Orthopedics 24:227-230.

[664]朱新民.(2000)针刺治疗中心性脉络膜视网膜病变32例的临床观察.针刺研究25:223-224.

[664]Zhu Xin-min.(2000)Clinical Observation on Effect of Acupuncture in Treating 32 Cases of Central Choroido-retinopathy.Acupuncture Research ＆ Orthopedics 25:223-224.

[665]何金柱, 李兰英, 徐伟. (2000)辨证取穴论治前列腺增生症.针刺研究, 25:300-301.

[665]He Jin-zhu, Li Lan-ying, Xu Wei.(2000)Acupuncture Treatment of Prostatic Hyperlasia by Selecting Acupoints according to Syndrome Differentiation.Acupuncture Research ＆ Orthopedic 25:300-301.

[666]尹志礼, 秦必光, 王焱林, [胡北喜](http://202.201.7.11/kcms/detail/search.aspx?dbcode=CJFQ&sfield=au&skey=胡北喜&code=05980555;13986583;21417732;05972650;21363216;06494138;), [唐育民](http://202.201.7.11/kcms/detail/search.aspx?dbcode=CJFQ&sfield=au&skey=唐育民&code=05980555;13986583;21417732;05972650;21363216;06494138;), 等.(2001)针刺复合安氟醚吸入全麻食管癌切除临床研究总结.针刺研究26:38-44.

[666]Yin Zhi-li, Qin Bi-guang, Wang Yan-lin, Hu Bei-xi, Tang Yu-min, et al.(2001)Summary of Clinical Research on Acupuncture Anesthesia in Combination with General Anesthesia of Enflurane Inhalation for Resection of Esophageal Carcinoma.Acupuncture Research ＆ Orthopedics 26:38-44.

[667]余华, 张安仁, 张月娥, [郑闵琴](http://202.201.7.11/kcms/detail/search.aspx?dbcode=CJFQ&sfield=au&skey=郑闵琴&code=05980968;20729118;06493027;08766768;06482749;21045660;20314499;), [王文莉](http://202.201.7.11/kcms/detail/search.aspx?dbcode=CJFQ&sfield=au&skey=王文莉&code=05980968;20729118;06493027;08766768;06482749;21045660;20314499;), 等.(2001)麦粒灸对脾虚泄泻证患者唾液淀粉酶活性及血清SIgA的影响.针刺研究26:63-66.

[667]Yu Hua, Zhang An-ren, Zhang Yue-e, Zheng Min-qin, Wang Wen-li, et al.(2001)Effect off Granule Moxibustion on Saliva Amylase Activity and Serum SIgA in Patients with Spleen Deficiency Type Diarrhea.Acupuncture Research ＆ Orthopedics 26:63-66.

[668]戴文军, 赖新生. (2001)天灸疗法对支气管哮喘患者血浆SP和VIP含量的影响.针刺研究26:134-137.

[668]Dai Wen-jun, Lai Xin-sheng.(2001)The Effect of Medicinal Vesiculation Therapy on Plasma SP

and VIP Contents in Bronehial Asthma Patients.Acupuncture Research ＆ Orthopedics 26:134-137.

[669]唐育民, 秦必光, 胡北喜, [魏湘华](http://202.201.7.11/kcms/detail/search.aspx?dbcode=CJFQ&sfield=au&skey=魏湘华&code=05980555;21417732;21363216;06494138;21587754;), [尹志礼](http://202.201.7.11/kcms/detail/search.aspx?dbcode=CJFQ&sfield=au&skey=尹志礼&code=05980555;21417732;21363216;06494138;21587754;).(2001)应用针刺复合安氟醚全麻食管癌根治术的临床研究.针刺研究26:138-142.

[669]Tang Yu-min, Qin Bi-guang, Hu Bei-xi, Wei Xiang-hua, Yin Zhi-li.(2001)Clinical Study on the Effect of Acupuncture Combined with Enflurane General Anesthesia for Esophagectomy.Acupuncture Research ＆ Orthopedics 26:138-142.

[670]睢明河, 马惠芳, 白杰. (2003)顶颞前斜线齐刺法治疗中风偏瘫的临床观察.针刺研究28:144-146.

[670]Sui Ming-he, Ma Hui-fang, Bai Jie.(2003)Clinical Observation on Acupuncture Treatment of Apoplectic Hemiplegia.Acupuncture Research ＆ Orthopedics 28:144-146.

[671]卢中平, 王祥瑞, 孙大金. (2003)电针刺激对缺血再灌注心肌保护中IL-8的作用机制.针刺研究28:210-213.

[671]Lu Zhong-ping, Wang Xiang-rui, Sun Da-jin.(2003)Involvement of IL-8 in the Protective Effect of Electroacupuncture on Myocardial Ischemia-reperfusion Injury.Acupuncture Research ＆ Orthopedics 28:210-213.

[672]黄伟贞. (2003)针刺对高脂血症患者血脂水平的影响.针刺研究28:218-220.

[672]Huang Wei-zhen.(2003)Effect of Acupuncture on Blood-lipid Level in Patients with Hyperlipemia.Acupuncture Research ＆ Orthopedics 28:218-220.

[673]李永方, 李尚丽, 温娟, [郑利星](http://202.201.7.11/kcms/detail/search.aspx?dbcode=CJFQ&sfield=au&skey=郑利星&code=20595164;20646188;21065544;20821532;20409992;13278192;), [郭秀英](http://202.201.7.11/kcms/detail/search.aspx?dbcode=CJFQ&sfield=au&skey=郭秀英&code=20595164;20646188;21065544;20821532;20409992;13278192;), 等.(2003)电针治疗糖尿病周围神经病变的神经电生理观察.针刺研究, 28:224-229.

[673]Li Yong-fang, Li Shang-li, Wen Juan, Zheng Li-xing, Guo Xiu-ying.et al.(2003)Clinical Observation on Nuroelectrophysiological Changes of Diabetic Peripheral Neuropathy after Electroacupuncture Treatment.Acupuncture Research ＆ Orthopedics 28:224-229.

[674]屈云, 蒋毅, 刘沙鑫, [何成奇](http://202.201.7.11/kcms/detail/search.aspx?dbcode=CJFQ&sfield=au&skey=何成奇&code=19001181;21323903;05975962;21468581;15504332;), [熊淑芳](http://202.201.7.11/kcms/detail/search.aspx?dbcode=CJFQ&sfield=au&skey=熊淑芳&code=19001181;21323903;05975962;21468581;15504332;).(2003)前后交替针刺配合手法治疗中风急性期后下肢偏瘫的临床研究.针刺研究28:267-269.

[674]Qu Yun, Jiang Yi, Liu Sha-xin, He Cheng-qi, Xiong Shu-fang.(2003)Observation on the Therapeutic Effect of Acupuncture and Massage for Treatment of Lower-limb Dysfunction in Stroke Patients.Acupuncture Research ＆ Orthopedics 28:267-269.

[675]江钢辉, 李艳慧, 黄勇, [李健萍](http://202.201.7.11/kcms/detail/search.aspx?dbcode=CJFQ&sfield=au&skey=李健萍&code=06929495;06929497;00156090;06930948;). (2004)头颅磁共振成像定位围针对中风偏瘫患者血液流变学的影响.针刺研究29:63-65.

[675]Jiang Gang-hui, Li Yan-hui, Huang Yong, Li Jian-ping.(2004)Effect of MRI-guided Surrounding Needling on Hemorheology of Stroke Patients. Acupuncture Research ＆ Orthopedics 29:63-65.

[676]伦新, 荣莉, 杨文辉. (2004)头颅CT定位围针对多发梗塞性痴呆患者生存质量的影响.针刺研究29:149-152.

[676]Lun Xin, Rong Li, Yang Wen-hui.(2004)Effect of CT-aided Scalp Surrounding Needling on the Life Quality of Multiple Cerebral Infarction Dementia Patients. Acupuncture Research ＆ Orthopedics 29:149-152.

[677]俞昌德, 吴炳煌, 洪安辉, [白靖宇](http://202.201.7.11/kcms/detail/search.aspx?dbcode=CJFQ&sfield=au&skey=白靖宇&code=06679747;06678607;06678464;06677407;06684843;), [俞宙](http://202.201.7.11/kcms/detail/search.aspx?dbcode=CJFQ&sfield=au&skey=俞宙&code=06679747;06678607;06678464;06677407;06684843;).(2004)头针加药物治疗早期脑梗死患者外周血丙二醇含量变化与神经功能康复的研究.针刺研究29:222-226.

[677]Yu Chang-de, Wu Bing-huang, Hong An-hui, Bai Jing-yu, Yu Zhou.(2004)Changes of Serum MDA Content and Neurological Rehabilitation in Cerebral Infarction Patients Treated with Scalp-acupuncture plus Medication.Acupuncture Research ＆ Orthopedics 29:222-226.

[678]周杰芳, 靳瑞.(2004)不同电针强度对高血压实证病人血压的影响.针刺研究29:286-288.

[678]Zhou Jie-fang, Jin Rui.(2004)Influence on Different Intensities of Electroacupuncture on Blood Pressure in Excess-syndrome Type Hypertension Patients.Acupuncture Research ＆ Orthopedics 29:286-288.

[679]汤国娟. (2004)扬刺温针灸治疗四肢筋痹的临床观察.针刺研究29:293-295.

[679]Tang Guo-juan.(2004)Clinical Observation on the Treatment of “Jinbi”(Muscular Rheumatism) by Centro-square Needling Combined with Moxibustion. Acupuncture Research ＆ Orthopedics 29:293-295.

[680]谢文霞, 王均炉. (2005)经皮穴位电刺激配合全麻行异氟醚控制性降压的可行性研究.针刺研究30:3-8.

[680]Xie Wen-xia, Wang Jun-lu.(2005)Study on the Applicability of Combined Transcutaneous Electrical Acupoint Stimulation and Routine Anesthesia for Cerebral Operation under Isoflurane-Controlled Hypotension.Acupuncture Research ＆ Orthopedics 30:3-8.

[681]邓宁. (2005)透刺法与药物治疗颈性眩晕症疗效对比观察.针刺研究30:48-49.

[681]Deng Ning.(2005)Comparative Study on Clinical Therapeutic Effects of Acupoint-to-acupoint Needling and Medication for Cervical Vertigo.Acupuncture Research ＆ Orthopedics 30:48-49.

[682]任昶, 高永辉. (2005)针刺治疗扁平疣临床观察.针刺研究30: 113-114.

[682]Ren Chang, Gao Yong-hui.(2005)Clinical Observation on Acupuncture of Verruca Plana.Acupuncture Research ＆ Orthopedics 30: 113-114.

[683]陈兴华, 赖新生. (2005)针刺对中风性假性球麻痹患者血浆内皮素及一氧化氮的影响.针刺研究30:171-174.

[683]Chen Xing-hua, Lai Xin-sheng.(2005)Effect of Acupuncture on Plasma Endothelin and NO Levels in Apoplectic Pseudobulbar Paralysis Patients.Acupuncture Research ＆ Orthopedics 30:171-174.

[684]赖新生, 黄泳.(2006)百会、水沟、神门影响血管性痴呆患者认知功能的比较研究.针刺研究31:54-57.

[684]Lai Xin-sheng, Huang Yong.(2006)Comparison of Clinical Effects of Acupuncture of Baihui(cv 20)，Shuigou(GV 26)and Shenmen(HT 7)on the Cognitive Function of Vascular Dementia Patients.Acupuncture Research ＆ Orthopedics 31:54-57.

[685]陈跃来, 岑珏, 侯文光, [高志强](http://202.201.7.11/kcms/detail/search.aspx?dbcode=CJFQ&sfield=au&skey=高志强&code=22992769;22745650;22761116;22711388;23064672;22827233;), [虞先敏](http://202.201.7.11/kcms/detail/search.aspx?dbcode=CJFQ&sfield=au&skey=虞先敏&code=22992769;22745650;22761116;22711388;23064672;22827233;), 等.(2006)不同针刺方法对女性尿道综合征膀胱容量影响的临床观察.针刺研究31:116-118.

[685]Chen Yue-lai, Cen Jue, Hou Wen-guang, Gao Zhi-qiang, Yu Xian-min, et al.(2006)Comparative Study on the Effects of Electroacupuncture plus Hand-acupuncture and Simple Hand-acupuncture On Bladder Volume in Female Urethral Syndrome Patients.Acupuncture Research ＆ Orthopedics 31:116-118.

[686]职良喜. (2006)复方亚甲蓝液有氧穴位注射治疗疱疹后神经痛的临床研究.针刺研究31:119-122.

[686]Zhi Liang-xi.(2006)Clinical Observation on the Therapeutic Effect of Acupoint Injection of Compound Methylthioninum Chloride for Post-herpetic Neuralgia.Acupuncture Research ＆ Orthopedics 31:119-122.

[687]张全明, 余瑞英, 庞坚, [周雅芳](http://202.201.7.11/kcms/detail/search.aspx?dbcode=CJFQ&sfield=au&skey=周雅芳&code=06928968;05972562;10296941;09397479;10296938;09442691;), [周茵](http://202.201.7.11/kcms/detail/search.aspx?dbcode=CJFQ&sfield=au&skey=周茵&code=06928968;05972562;10296941;09397479;10296938;09442691;), 等.(2006)针刺对儿童药物中毒性听力语言障碍脑干听觉诱发电位的影响.针刺研究31:163-165.

[687]Zhang Quan-ming, Yu Rui-ying, Pang Jian, Zhou Ya-fang, Zhou Yin, et al.(2006)Effect of Acupuncture Oil Brainstem Auditory Evoked Potential in Children with Hearing and Language Disorders.Acupuncture Research ＆ Orthopedics 31:163-165.

[688]李虹, 侯中伟, 白玉兰, [谷世喆](http://202.201.7.11/kcms/detail/search.aspx?dbcode=CJFQ&sfield=au&skey=谷世喆&code=06436158;06430415;06434744;06435038;).(2006)头针、体针及头体针结合治疗中风230例的疗效比较.针刺研究31:169-172.

[688]Li Hong, Hou Zhong-wei, Bai Yu-lan, Gu Shi-zhe.(2006)Comparison of the Therapeutic Effects among Scalp-，Body-acupuncture and Scalp-plus Body-acupuncture in the Treatment of 230 Cases of Stroke.Acupuncture Research ＆ Orthopedics 31:169-172.

[689]韩颖, 王宏才, 翟桂荣, [张红卫](http://202.201.7.11/kcms/detail/search.aspx?dbcode=CJFQ&sfield=au&skey=张红卫&code=11663683;06287611;16180033;16773389;16679468;16028382;), [魏立新](http://202.201.7.11/kcms/detail/search.aspx?dbcode=CJFQ&sfield=au&skey=魏立新&code=11663683;06287611;16180033;16773389;16679468;16028382;), 等.(2006)电针少泽穴对乳汁分泌不足产妇催乳作用的疗效观察.针刺研究31:173-175.

[689]Han Ying, Wang Hong-cai, Zhai Gui-rong, Zhang Hong-wei, Wei Li-xin, et al.(2006)Observation on the Therapeutic Effect of Electroacupuncture of Shaoze(SI 1)in the Treatment of Parturients with Hypolactation.Acupuncture Research ＆ Orthopedics 31:173-175.

[690]姚筱梅, 姚树坤, 张瑞星, [常丽丽](http://202.201.7.11/kcms/detail/search.aspx?dbcode=CJFQ&sfield=au&skey=常丽丽&code=07161013;07164944;05976562;07164206;). (2006)针刺对功能性消化不良患者内脏敏感性的影响.针刺研究31:228-231.

[690]Yao Xiao-mei, Yao Shu-kun, Zhang Rui-xing, Chang Li-li, (2006)Effect of Electroacupuncture Stimulation on Visceral Sensitivity in Patients with Functional Dyspepsia.Acupuncture Research ＆ Orthopedics 31:228-231.

[691]许云祥, 张家维. (2006)电针对糖尿病性认知功能障碍患者学习记忆的影响.针刺研究31:232-234.

[691]Xu Yun-xiang, Zhang Jia-wei.(2006)Influence of Electroacupuncture on Learning and Memory Ability in Diabetic Cognitive Dysfunction Patients.Acupuncture Research ＆ Orthopedics 31:232-234.

[692]符文彬, 樊莉, 朱晓平, [何青](http://202.201.7.11/kcms/detail/search.aspx?dbcode=CJFQ&sfield=au&skey=何青&code=06830208;07503237;06839439;06819358;00019613;10746497;06931332;06844072;06844720;), [王玲](http://202.201.7.11/kcms/detail/search.aspx?dbcode=CJFQ&sfield=au&skey=王玲&code=06830208;07503237;06839439;06819358;00019613;10746497;06931332;06844072;06844720;), 等. (2006)针刺调肝法治疗抑郁性神经症的临床研究.针刺研究31:355-358.

[692]Fu Wen-bin, Fan Li, Zhu Xiao-ping, He Qing, Wang Ling, et al.(2006)Clinical Research on Acupuncture Treatment of Depressive Neurosis by Using Liver-function-regulating Method.Acupuncture Research ＆ Orthopedics 31:355-358.

[693]李砚青.(2006)针刺合并综合疗法治疗假性延髓麻痹54例疗效观察.针刺研究31:359-361.

[693]Li Yan-qing.(2006)Observation on the Therapeutic Effect of Combined Acupuncture Therapy on Dysphagia from Pseudobulbar Palsy in 54 Cases of Stroke Patients.Acupuncture Research ＆ Orthopedics 31:359-361.

[694]奚玉凤, 邹婷, 艾宙, 等. (2006)清热利湿祛瘀针刺法治疗急性痛风性关节炎的临床分析.针刺研究31:362-364.

[694]Xi Yu-feng, Zou Ting, Ai Zhou, et al.(2006)Clinical Observation on Acupuncture Treatment of Acute Gouty Arthritis in Terms of Clearing Heat，Eliminating Dampness and Stasis.Acupuncture Research ＆ Orthopedics 31:362-364.

[695]何永昌. (2006)电针加DAJ艾灸仪治疗跟痛症临床观察.针刺研究31:368.

[695]He Yong-chang.(2006)Clinical Observation on Electroacupuncture and DAJ Moxibustion Apparatus for Treatment of calcanodynia. Acupuncture Research ＆ Orthopedics 31:368.

[696][张文彭](http://202.201.7.11/kcms/detail/search.aspx?dbcode=CJFQ&sfield=au&skey=张文彭&code=20765347;11602794;11561915;06634804;06637577;06634805;), [Е.В.Владимирский](http://202.201.7.11/kcms/detail/search.aspx?dbcode=CJFQ&sfield=au&skey=Е.В.Владимирский&code=20765347;11602794;11561915;06634804;06637577;06634805;), [А.В.Туев](http://202.201.7.11/kcms/detail/search.aspx?dbcode=CJFQ&sfield=au&skey=А.В.Туев&code=20765347;11602794;11561915;06634804;06637577;06634805;), [В.Л.Кучерский](http://202.201.7.11/kcms/detail/search.aspx?dbcode=CJFQ&sfield=au&skey=В.Л.Кучерский&code=20765347;11602794;11561915;06634804;06637577;06634805;), [Е.В.Семеиных](http://202.201.7.11/kcms/detail/search.aspx?dbcode=CJFQ&sfield=au&skey=Е.В.Семеиных&code=20765347;11602794;11561915;06634804;06637577;06634805;), [Е.Г.Паршакова](http://202.201.7.11/kcms/detail/search.aspx?dbcode=CJFQ&sfield=au&skey=Е.Г.Паршакова&code=20765347;11602794;11561915;06634804;06637577;06634805;) , 等. (2007)宣肺健脾益肾针刺法对不同程度支气管哮喘患者心率变异性与肺功能的影响.针刺研究32:42-48.

[696] Zhang Wen-peng, [Е.В.Владимирский](http://202.201.7.11/kcms/detail/search.aspx?dbcode=CJFQ&sfield=au&skey=Е.В.Владимирский&code=20765347;11602794;11561915;06634804;06637577;06634805;), [А.В.Туев](http://202.201.7.11/kcms/detail/search.aspx?dbcode=CJFQ&sfield=au&skey=А.В.Туев&code=20765347;11602794;11561915;06634804;06637577;06634805;), [В.Л.Кучерский](http://202.201.7.11/kcms/detail/search.aspx?dbcode=CJFQ&sfield=au&skey=В.Л.Кучерский&code=20765347;11602794;11561915;06634804;06637577;06634805;), [Е.В.Семеиных](http://202.201.7.11/kcms/detail/search.aspx?dbcode=CJFQ&sfield=au&skey=Е.В.Семеиных&code=20765347;11602794;11561915;06634804;06637577;06634805;), [Е.Г.Паршакова](http://202.201.7.11/kcms/detail/search.aspx?dbcode=CJFQ&sfield=au&skey=Е.Г.Паршакова&code=20765347;11602794;11561915;06634804;06637577;06634805;) , et al.(2007).Effects of Acupuncture on the Pulmonary Function and Heart Rate Variability in Different State of Bronchial Asthma.Acupuncture Research ＆ Orthopedics 32:42-48.

[697]陈锋, 吴松, 张艳. (2007)穴位埋线对单纯性肥胖症患者肿瘤坏死因子-α和胰岛素抵抗的影响.针刺研究32: 49-52.

[697]Chen Feng, Wu Song, Zhang Yan.(2007)Effect of Acupoint Catgut Embedding on TNF-a and Insulin Resistance in Simple Obesity Patients.acupuncture Research ＆ Orthopedics 32: 49-52.

[698]周立武. (2007)恢刺治疗颈源性头痛临床观察.针刺研究32: 67.

[698]Zhou Li-wu.(2007)Clinical Observation on Hui puncture of Cervicogenic Headache.acupuncture Research ＆ Orthopedics 32: 67.

[699]王新宇. (2007)针刺对广泛性子宫切除术后膀胱功能影响的研究.针刺研究, 32: 132-135.

[699]Wang Xin-yu.(2007)Effect of Acupuncture on Bladder Function in Patients with Radical Hysterectomy.acupuncture Research ＆ Orthopedics 32: 132-135.

[700]李月梅, 庄礼兴, 赖新生, [江钢辉](http://202.201.7.11/kcms/detail/search.aspx?dbcode=CJFQ&sfield=au&skey=江钢辉&code=06926018;06929045;06930948;05966168;).(2007)电针对常年性变应性鼻炎患者血浆血管活性肠肽和P物质的影响.针刺研究32:136-138.

[700]Li Yue-mei, Zhuang Li-xing, Lai Xin-sheng, Jiang Gang-hui.(2007)Effects of Electroacupuncture on Plasma Vasoactive Intestinal Peptide and Substance P in Perennial Allergic Rhinitis Patients.acupuncture Research ＆ Orthopedics 32:136-138.

[701]张慧敏. (2007)头穴丛刺结合言语康复治疗脑卒中运动性失语临床研究.针刺研究32: 190-194.

[701]Zhuang Hui-min.(2007)Clinical Treatment of Apoplectic Aphemia with Multi-needle Puncture of Scalp-points in Combination with Visual-Listening-Speech Training. acupuncture Research ＆ Orthopedics 32: 190-194.

[702]白桦, 于澎, 于淼. (2007)电针对干燥综合征患者性激素水平影响的研究.针刺研究32: 203-206.

[702]Bai Hua, Yu Peng, Yu miao.(2007)Effect of Electroacupuncture on Sex Hormone Levels in Patients with Sjögren's syndrome.acupuncture Research ＆ Orthopedics 32: 203-206.

[703]秦正玉, 胡玲, 夏晓红, [李梦](http://202.201.7.11/kcms/detail/search.aspx?dbcode=CJFQ&sfield=au&skey=李梦&code=06215095;06206644;06210683;06206711;06210061;), [吴子建](http://202.201.7.11/kcms/detail/search.aspx?dbcode=CJFQ&sfield=au&skey=吴子建&code=06215095;06206644;06210683;06206711;06210061;).(2007)电针三阴交对围绝经期综合征患者生殖内分泌影响的随机对照研究.针刺研究32: 255-259.

[703]Qin Zheng-yu, Hu Ling, Xia Xiao-hong, Li Meng, Wu Zi-jian.(2007)Effects of Electroacupuncture of Sanyinjiao(SP 6)on Genito-endocrine in Patients with Perimenopausal Syndrome.acupuncture Research ＆ Orthopedics 32: 255-259.

[704]褚芹, 王琳, 刘国真. (2007)针刺对糖尿病肾病血液流变学影响的临床随机对照研究.针刺研究32: 335-337.

[704]Chu Qin, Wang Lin, Liu Guo-zhen.(2007)Effect of Acupuncture on Hemorheology in Patients with Diabetic Nephropathy.acupuncture Research ＆ Orthopedics 32: 335-337.

[705]何扬子, 韩冰, 郑仕富, [王丽娜](http://202.201.7.11/kcms/detail/search.aspx?dbcode=CJFQ&sfield=au&skey=王丽娜&code=07776462;07776691;07744335;07744220;07744256;07743793;10627790;), [陈卓铭](http://202.201.7.11/kcms/detail/search.aspx?dbcode=CJFQ&sfield=au&skey=陈卓铭&code=07776462;07776691;07744335;07744220;07744256;07743793;10627790;), 等. (2007)不同留针时间针刺对缺血性中风患者血液流变学的影响.针刺研究32: 338-341.

[705]He Yang-zi, Han Bing, Zheng Shi-fu, Wang Li-na, Chen Zhuo-ming, et al.(2007).Effect of Different Acupuncture Needle-retaining Time On Hemorheology in Ischemic Stroke Patients.acupuncture Research ＆ Orthopedics 32: 338-341.

[706]王均炉, 任秋生, 沈财成, [谢文霞](http://202.201.7.11/kcms/detail/search.aspx?dbcode=CJFQ&sfield=au&skey=谢文霞&code=10513465;10513500;08995057;08972956;10513470;10129097;), [郑瑞献](http://202.201.7.11/kcms/detail/search.aspx?dbcode=CJFQ&sfield=au&skey=郑瑞献&code=10513465;10513500;08995057;08972956;10513470;10129097;), 等.(2008)经皮穴位电刺激对开颅术围术期脑损伤相关因子的影响.针刺研究33: 26-30.

[706]Wang Jun-lu, Ren Qiu-sheng, Shen Cai-cheng, Xie Wen-xia, Zheng Rui-xian, et al.(2008)Effect of Transcutaneous Acupoint Electrical Stimulation on Blood Bioactive Compounds Involving Cerebral lnjury During Craniotomy.acupuncture Research ＆ Orthopedics 33: 26-30.

[707]刘智斌, 牛文民, 杨晓航, [牛晓梅](http://202.201.7.11/kcms/detail/search.aspx?dbcode=CJFQ&sfield=au&skey=牛晓梅&code=09514708;15108997;15112003;14239477;). (2008)嗅三针治疗血管性痴呆的随机对照研究.针刺研究 33: 131-134.

[707]Liu Zhi-bin, Niu Wen-min, Yang Xiao-hang, Niu Xiao-mei.(2008)Clinical Investigation on Electroacupuncture Treatment of Vascular Dementia with“Xiusanzhen”.acupuncture Research ＆ Orthopedics 33: 131-134.

[708]刘喜德, 张金禄, 郑汉光, [刘风云](http://202.201.7.11/kcms/detail/search.aspx?dbcode=CJFQ&sfield=au&skey=刘风云&code=10589669;10303244;09449477;10589671;10303221;), [陈滢](http://202.201.7.11/kcms/detail/search.aspx?dbcode=CJFQ&sfield=au&skey=陈滢&code=10589669;10303244;09449477;10589671;10303221;).(2008)蜂针疗法治疗类风湿关节炎的临床随机对照研究.针刺研究33: 197-200.

[708]Liu Xi-de, Zhang Jin-lu, Zheng Han-guang, Liu Feng-yun, Chen Ying.(2008)Clinical Randomized Study of Bee-sting Therapy for Rheumatoid Arthritis.acupuncture Research ＆ Orthopedics 33: 197-200.

[709]肖晓玲, 刘志顺.(2008)不同时间电针治疗失眠疗效评价.针刺研究33: 201-204.

[709]Xiao Xiao-ling, Liu Zhi-shun.(2008)Comparison of Therapeutic Effects of Electroacupuncture Treatment of Insomnia at Different Time.acupuncture Research＆Orthopedics 33: 201-204.

[710]夏晓红, 胡玲, 秦正玉, [周军](http://202.201.7.11/kcms/detail/search.aspx?dbcode=CJFQ&sfield=au&skey=周军&code=06210683;06206644;06210061;17608445;06212346;06216482;07065639;23041634;), [李梦](http://202.201.7.11/kcms/detail/search.aspx?dbcode=CJFQ&sfield=au&skey=李梦&code=06210683;06206644;06210061;17608445;06212346;06216482;07065639;23041634;), 等.(2008）电针三阴交治疗围绝经期综合征多中心随机对照研究.针刺研究33: 262-266.

[710]Xia Xiao-hong, Hu Ling, Qin Zheng-yu, Zhou Jun, Li Meng, et al.(2008)Multicentral Randomized Controlled Clinical Trials about Treatment of Perimenopausal Syndrome with Electroacupuncture of Sanyinjiao(SP 6).acupuncture Research ＆Orthopedics 33:262-266.

[711] 楚佳梅, 包烨华, 邹超.(2008)头穴久留针对血管性痴呆病人认知和生活能力及P300的影响.针刺研究33:334-338.

[711] Chu Jia-mei, Bao Ye-hua, Zou Chao, (2008)Effect of Long-time Retention of Scalp Needle on the Abilities of Cognition。Daily Living Activity and P300 in Vascular Dementia Patients.acupuncture Research ＆Orthopedics 33:334-338.

[712] 刘长征, 雷波, 郑剑峰.(2008)围刺结合刺络拔罐治疗聚合性痤疮的随机对照研究.针刺研究33:406-408.

[712]Liu Chang-zheng, Lei Bo, Zheng Jian-feng.(2008)Randomized Control Study on the Treatment of 26 Cases of Acne Conglobata with Encircling Acupuncture Combined with Venesection and Cupping.acupuncture Research ＆Orthopedics 33:406-408.

[713] 杨继军, 孙立虹, 余延芬, [葛建军](http://202.201.7.11/kcms/detail/search.aspx?dbcode=CJFQ&sfield=au&skey=葛建军&code=07164352;07159708;07155419;07159321;13982061;15519439;), [李新华](http://202.201.7.11/kcms/detail/search.aspx?dbcode=CJFQ&sfield=au&skey=李新华&code=07164352;07159708;07155419;07159321;13982061;15519439;), 等.(2008)隔物灸对寒湿凝滞型原发性痛经患者内皮素和一氧化氮含量的影响.针刺研究33:409-412.

[713]Yang Ji-jun, Sun Li-hong, Yu Yan-fen, Ge Jian-jun, Li Xin-hua, et al.(2008)Influence of Ginger-Partitioned Moxibustion on Serum NO and Plasma Endothelin-1 Contents in Patients with Primary Dysmenorrhea of Cold—damp Stagnation Type.acupuncture Research ＆Orthopedics 33:409-412.

[714] 孙立虹, 葛建军, 杨继军, [佘延芬](http://202.201.7.11/kcms/detail/search.aspx?dbcode=CJFQ&sfield=au&skey=佘延芬&code=07159708;07159321;07164352;07155419;07152290;13982061;17600349;07138208;14245144;07126786;20782666;07135656;07135749;), [李文丽](http://202.201.7.11/kcms/detail/search.aspx?dbcode=CJFQ&sfield=au&skey=李文丽&code=07159708;07159321;07164352;07155419;07152290;13982061;17600349;07138208;14245144;07126786;20782666;07135656;07135749;), 等. (2009)隔物灸治疗寒湿凝滞型原发性痛经的随机对照临床研究.针刺研究34:398-402.

[714]Sun Li-hong, Ge Jian-jun, Yang Ji-jun, Yu Yan-fen, Li Wen-li, et al.(2009)Randomized Controlled Clinical Study on Ginger-partitioned Moxibustion for Patients with Cold-damp Stagnation Type Primary Dysmenorrhea.acupuncture Research ＆Orthopedics 34:398-402.

[715] 张宁霞, 刘桂珍, 黄太权, [李蔚江](http://202.201.7.11/kcms/detail/search.aspx?dbcode=CJFQ&sfield=au&skey=李蔚江&code=22953485;22819042;23007903;23856776;23856777;23856778;23856779;23042789;), [罗家其](http://202.201.7.11/kcms/detail/search.aspx?dbcode=CJFQ&sfield=au&skey=罗家其&code=22953485;22819042;23007903;23856776;23856777;23856778;23856779;23042789;), 等. (2009)热补针法结合康复训练对缺血性脑卒中偏瘫患者早期运动功能的影响.针刺研究34: 406-409.

[715]Zhang Ning-xia, Liu Gui-zhen, Huang Tai-quan, Li Wei-jiang, Luo Jia-qi, et al.(2009)Effects of Warming-reinforcing acupuncture combined with rehabilitation training on the early motor Function of Hemiparalysis Patients caused by ischemic Brain Stroke.acupuncture Research ＆Orthopedics 34:406-409.

[716] 李常法, 贾春生, 李晓峰, [石晶](http://202.201.7.11/kcms/detail/search.aspx?dbcode=CJFQ&sfield=au&skey=石晶&code=07158594;07154941;11018684;07155427;24110589;15555542;), [豆真真](http://202.201.7.11/kcms/detail/search.aspx?dbcode=CJFQ&sfield=au&skey=豆真真&code=07158594;07154941;11018684;07155427;24110589;15555542;), 等.(2010)耳针沿皮透刺配合体针对急性期脑梗死患者肌力和神经功能恢复的影响.针刺研究35:56-60.

[716]Li Chang-fa, Jia Chun-sheng, Li Xiao-feng, Shi Jing, Dou Jing-jing, et al.(2010)Effect of Penetrative Needling of Otopoints Combined with Body Acupuncture on Limb Myodynamia and Neurofunction in Patients with Acute Cerebral Infarction.acupuncture Research ＆Orthopedics35:56-60.

[717] 马文珠, 周培娟, 张怡, [袁英](http://202.201.7.11/kcms/detail/search.aspx?dbcode=CJFQ&sfield=au&skey=袁英&code=05967573;06437407;06437350;06438827;24624362;06367554;08245094;), [吴颖](http://202.201.7.11/kcms/detail/search.aspx?dbcode=CJFQ&sfield=au&skey=吴颖&code=05967573;06437407;06437350;06438827;24624362;06367554;08245094;), 等.(2010)电针三阴交对产程影响的临床观察.针刺研究35:217-221.

[717]Ma Wen-zhu, Zhou Pei-juan, Zhang Yi, Yuan Ying, Wu Ying, et al.(2010)Clinical Observation on the Effect of Electroacupuncture of Sanyinjiao(SP 6) on Labor.acupuncture Research ＆Orthopedics 35:217-221.

[718] 朱英, 陈日兰, 苗芙蕊, [姬乐](http://202.201.7.11/kcms/detail/search.aspx?dbcode=CJFQ&sfield=au&skey=姬乐&code=22948000;22991175;23415141;22400846;).(2010)隔药灸结合电针治疗寒湿型膝骨性关节炎的疗效观察.针刺研究35:293-297.

[718]Zhu Ying, Chen Ri-lan, Miao Fu-rui, Ji Le.(2010)Clinical Observation on the Therapeutic Effect of Drugs-paste Separated Moxibustion Combined with Electroacupuncture for Knee Osteoarthritis Patients of Cold-damp Type.acupuncture Research ＆Orthopedics 35:293-297.

[719] 赖毛华, 马红霞, 姚红, [刘华](http://202.201.7.11/kcms/detail/search.aspx?dbcode=CJFQ&sfield=au&skey=刘华&code=22687576;06924267;06924368;06924231;23991138;23416766;07357325;), [宋兴华](http://202.201.7.11/kcms/detail/search.aspx?dbcode=CJFQ&sfield=au&skey=宋兴华&code=22687576;06924267;06924368;06924231;23991138;23416766;07357325;), 等. (2010)腹针对肥胖型多囊卵巢综合征患者内分泌及糖脂代谢的影响.针刺研究35:298-302.

[719]Lai Mao-hua, Ma Hong-xia, Yao Hong, Liu Hua, Song Xing-hua, et al.(2010)Effect of Adominal Acupuncture Therapy on the Endocrine and Metabolism in Obesity-type Polycystic Ovarian Syndrome Patients.acupuncture Research ＆Orthopedics 35:298-302.

[720] 梅春林.(1996).27例坐骨神经痛的穴位辨证治疗.针灸临床杂志12:42.

[720]Mei Chun-lin.(1996).Treating 27 Cases of Sciatica with Acupoint and dialectical Treatment.acupuncture Research ＆Orthopedics 12:42.

[721] 刘凤波, 付美香.(2005)G6805加神灯治疗面肌痉挛102例临床观察.针灸临床杂志21:45.

[721]Liu Feng-bo, Fu Mei-xiang.(2005)Clinical Observation on G6805 and TDP of hemifacial spasm in 102 Cases of Patients.Journal of Clinical Acupuncture and Moxibustion＆Orthopedics 21:45.

[722] 杨兆民, 刘农虞.(1996)SMY-10型电脑随机脉冲针疗仪治疗痛证的临床观察.针灸临床杂志12:42.

[722]Yang Zhao-min, Liu Nong-yu.(1996)Clinical Observation on SMY-10 Type Computer Random pulse therapeutic device for Treatment of Acute Pains.Journal of Clinical Acupuncture and Moxibustion＆Orthopedics 12:42.

[723] 李绍军, 谯智泉, 喻凤文, 等. (2010)阿是穴臭氧注射联合推拿治疗粘连期肩周炎临床疗效观察针灸.针灸临床杂志26:32-34.

[723]Li Shao-jun, Qiao Zhi-quan, Yu Feng-wen, et al.(2010)Clinical Observation on the Therapeutic Effect of O₃Ashi-points injection Combined with Massage Therapies in Treating the Periarthritis of Shoulder in Adherence Term.Journal of Clinical Acupuncture and Moxibustion＆Orthopedics 26:32-34.

[724] 吴咚咚, 李岩.(2010)八脉交会穴配合耳穴治疗心脾两虚型慢性疲劳综合征针灸.针灸临床杂志26:31-33.

[724]Wu Dong-dong, Li Yan.(2010)The Treatment of Both Heart and Spleen Deficiency Type of Chronic Fatigue Syndrome by Eight Confluent Points with Ear Acupuncture.Journal of Clinical Acupuncture and Moxibustion＆Orthopedics 26:31-33.

[725] 张俊, 张德基.(1996)邦迪辣椒痛可贴与针刺拔罐结合治疗坐骨神经痛针灸.针灸临床杂志12:32.

[725]Zhang Jun, Zhang De-ji.(1996)Bangdi Chilli Plaster Combined with Acupuncture and Cupping in Treating the sciatica.Journal of Clinical Acupuncture and Moxibusti＆Orthopedics 12:32.

[726] 哈力甫, 陈跃来, 申鹏飞, [陈国美](http://202.201.7.11/kcms/detail/search.aspx?dbcode=CJFQ&sfield=au&skey=陈国美&code=23000786;22992769;10243262;22714462;23035309;), [葛林宝](http://202.201.7.11/kcms/detail/search.aspx?dbcode=CJFQ&sfield=au&skey=葛林宝&code=23000786;22992769;10243262;22714462;23035309;).(2004)膀胱募穴与合穴对尿道动力学影响的临床研究.针灸临床杂志20:51.

[726]Ha Li-fu, Chen Yue-lai, Shen Peng-fei, Chen Guo-mei.Ge Lin-bao.(2004)Clinical Research on Impact on urethral dynamic with Bladder Ming Mu points and the sea points.Journal of Clinical Acupuncture and Moxibustion＆Orthopedics 20:51.

[727] 薛青理. (2010)臂丛阻滞、推拿手法松解结合穴位注射治疗肩周炎疗效观察.针灸临床杂志26:15-17.

[727]Xue Qing-li.(2010)Observation on Therapeutic Effect of the Brachial Plexus Blocking Therapy and the Massage Technique Therapy and the Meridians Injection Therapy Combined to Treat Periarthritis.Journal of Clinical Acupuncture and Moxibustion＆Orthopedics 26:15-17.

[728] 金承香, 金书, 夏东斌, [黄泳](http://202.201.7.11/kcms/detail/search.aspx?dbcode=CJFQ&sfield=au&skey=黄泳&code=20622618;25147594;22905520;05977183;22021093;23741476;), [肖慧玲](http://202.201.7.11/kcms/detail/search.aspx?dbcode=CJFQ&sfield=au&skey=肖慧玲&code=20622618;25147594;22905520;05977183;22021093;23741476;), 等.(2010)薄氏腹穴皮下浅刺配合落枕穴针刺治疗落枕.针灸临床杂志26:45-47.

Jin Cheng-xiang, Jin-shu, Xia Dong-bin, Huang Yong, Xiao Hui-ling, et al.(2010)A Clinical Observation on Treating Stiff Neck by the Combination of Superficial Needling at Bo’s Abdominal Acupoints and Laozhen Point.Journal of Clinical Acupuncture and Moxibustion＆Orthopedics 26:45-47.

[728][729] 皮敏, 饶晓丹, 曹雪梅. (2007)不同刺法对急性脑梗塞患者CRP水平影响的临床研究针灸. 针灸临床杂志23:1-4.

PI Min, Rao Xiao-dan, Cao Xue-mei.(2007)Study on the Effects of Different Acupuncture Therapies on Serum C- reactive protein level in patients with acute cerebral infarction.

Journal of Clinical Acupuncture and Moxibustion＆Orthopedics 23:1-4.

[730] 宋立中, 张泉玲, 李艳梅. (2007)辰时、酉时电针对脑血栓患者TXB_2、PGF_(1a)的影响.针灸临床杂志23:25-28.

[730] Song Li-zhong, Zhang Quan-ling, Li Yan-mei.(2007)Impact on TXB_2 and FGF_(1a) in Patients with cerebral thrombosis Treated with Electro-acupuncture at Chenshi 08:00 and Electro-acupuncture at Youshi 18:00.Journal of Clinical Acupuncture and Moxibustion＆Orthopedics 23:25-28.

[731] 马广昊, 顾群.(2010)粗针通督疗法治疗寻常性痤疮43例针灸.针灸临床杂志26:9-11.

[731]Ma Guang-hao, Gu Qun.(2010)Therapeutic Effect of Rough Needle on Du Longitude about 43 Cases Acne Vulgaris.Journal of Clinical Acupuncture and Moxibustio＆Orthopedics26:9-11.

[732] 丁放.(2007)单纯灸法治疗类风湿关节炎针灸.针灸临床杂志23:40-41.

[732]Ding Fang.(2007)Clinical Observation on Rheumatoid Arthritis Treated Purely by Moxibustion.Journal of Clinical Acupuncture and Moxibustio＆Orthopedics 23:40-41.

[733] 王宇, 曹华, 邢艳丽.(2010)电鍉丛针结合认知训练治疗轻度认知功能障碍针灸.针灸临床杂志26:32-33.

[733]Wang Yu, Cao Hua, Xing Yan-li.(2010)Observation of Effect of Cluster Needing of Electro一‘di’Acupuncture Combination of Cognitive Training in Acupuncture Treatment of Mild Cognitive Impairment.Journal of Clinical Acupuncture and Moxibustio＆Orthopedics 23:40-41.

[734] 钟丽霞, 袁宜勤. (2008)电针、牵引配合项肌静力练习治疗颈型颈椎病的临床观察针灸.针灸临床杂志24:22-23.

[734]Electro-acupuncture, Neck Traction and Nucha Muscle’s Isometric Contraction for Treatment of Cervieum-type Cervical.Journal of Clinical Acupuncture and Moxibustio＆Orthopedics 24:22-23.

[735] 陶群, 陆惠新.(2007)电针拔罐刺血治疗腰突症相关腹主动脉钙化疗效观察针灸.针灸临床杂志23:46.

[735]Tao Qun, Lu Hui-xin.(2007)Clinical Observation on the Therapeutic Effect of Electro-acupuncture and Cupping combined with Bloodletting in Treating Lumbar Disc Herniation Correlates Abdominal Aortic Calcification. Journal of Clinical Acupuncture and Moxibustio＆Orthopedics 23:46.

[736] 石育才.(2008)电针“扳机点”法治疗三叉神经痛.针灸临床杂志24:32-33.

[736] Shi Yu-cai.(2008)Trigeminal Neuralgia Treated by Electro-acupuncture trigger points.Journal of Clinical Acupuncture and Moxibustio＆Orthopedics 24:32-33.

[737] 蔡国伟, 梁书忠.(1996)电针膀胱丛刺激点治疗小儿遗尿20例疗效观察.针灸临床杂志12:12.

[737]Cai Guo-wei, Liang Shu-zhong.(1996)Clinical Observation on the Therapeutic Effect of Electro-acupuncture Urinary Bladder Clump of stimulus in Treating Children Enuresis in 20 Cases of Patients.Journal of Clinical Acupuncture and Moxibustio＆Orthopedics 12:12.

[738] 佀雪平, 丛惠芳, 王晓滨, 等. (2006)电针刺激合谷穴预防麻醉诱导期心血管抑制临床观察针灸.针灸临床杂志 3:4-7.

[738]Si Xue-ping, Cong Hui-fang, Wang Xiao-bin, et al.(2006)The Clinical Observation on the Electric Needle Stimulating to prevent the Cardiovascular Depression in the Process of Vein Anesthesia Induction.Journal of Clinical Acupuncture and Moxibustio＆Orthopedics 3:4-7.

[739] 蔡国伟, 朱达义.(1996)电针刺激神经干配合悬灸对单纯性面神经炎疗效观察针灸. 针灸临床杂志12:65.

[739]Cai Guo-wei, Zhu Da-yi.(1996)Clinical Observation on the Therapeutic Effect of Electroacupuncture Irritates neural stem Combined with Suspended Moxibustion in Treating Simple Facial Paralysis.Journal of Clinical Acupuncture and Moxibustio＆Orthopedics 12:65.

[740] 牟淑兰, 石铁英.(2010)电针对腹部术后胃瘫综合征的干预治疗针灸.针灸临床杂志26:27-28.

[740]Mou Shu-lan, Shi Tie-ying.(2010)Electro-acupuncture Intervention Treatment of Abdominal

Postsurgical Gastroparesis Syndrome.Journal of Clinical Acupuncture and Moxibustio＆Orthopedics 26:27-28.

[741] 时金华, 孙有谦, 高寅秋.(2008)电针干预全麻气管插管应激反应的多中心临床研究针灸.针灸临床杂志24:1-3.

[741]Shi Jin-hua, Sun You-qian, Gao Yan-qiu.(2008)Multicenter Studies of Clinical Curative Effect of Electroacupuncture Interferes the Stress of Trachea Cannula in General Anesthesia.Journal of Clinical Acupuncture and Moxibustio＆Orthopedics 24:1-3.

[742] 谢凯. (2002)电针加TDP治疗腰椎间盘突出症临床观察 .针灸临床杂志18:32.

[742]Xie Kai.(2002)Clinical Observation on Prolapse of Lumbar Intervertebral Disc Treated with Electric Acupuncture and TDP.Journal of Clinical Acupuncture and Moxibustio＆Orthopedics 18:32.

[743] 陈桢艳, 张红星, 张唐法, [刘翼程](http://202.201.7.11/kcms/detail/search.aspx?dbcode=CJFQ&sfield=au&skey=刘翼程&code=09022000;16644158;15605069;15858810;09022031;09046429;), [代义](http://202.201.7.11/kcms/detail/search.aspx?dbcode=CJFQ&sfield=au&skey=代义&code=09022000;16644158;15605069;15858810;09022031;09046429;), 等.(2008)电针加耳穴贴压治疗单纯性肥胖症疗效观察.针灸临床杂志24:3-5.

[743]Chen Zhen-yan, Zhang Hong-xing, Zhang Tang-fa, Liu Yi-cheng, Dai Yi, et al.(2008)The Observation on the Efficacy of Acupuncture Treatment on Pure Adiposity.Journal of Clinical Acupuncture and Moxibustio＆Orthopedics 24:3-5.

[744] 李萍.(2009)电针配合拔罐治疗腰椎间盘突出症疗效观察.针灸临床杂志25:31-32.

[744]Li Ping.(2009)Observation of Electric Acupuncture Combined with Ventouse on Lubar Intervertebral Disc Protrusion.Journal of Clinical Acupuncture and Moxibustio＆Orthopedics 25:31-32.

[745] 刘志良. (2002)电针配合硬脊膜外注药治疗腰椎间盘突出症的临床观察.针灸临床杂志18:47-48.

[745]Liu Zhi-liang.(2002)Clinical Observation on Prolapse of Lumbar Intervertebral Disc Treated with Electric Acupuncture and Extradural Injection of Spinal Cord.Journal of Clinical Acupuncture and Moxibustion 18:47-48.

[746] 杨俊生, 衣蕾, 朱云清.(2007)电针为主治疗顽固性呃逆47例临床观察.针灸临床杂志23:24-25.

[746]Yang Jun-sheng, Yi Lei, Zhu Yun-qing.(2007)Observation on clinical therapeutic effect of electric acupuncture on refractoriness hiccough.Journal of Clinical Acupuncture and Moxibustion 23:24-25.

[747] 刁永红, 韩秀华, 马华, [梁万增](http://202.201.7.11/kcms/detail/search.aspx?dbcode=CJFQ&sfield=au&skey=梁万增&code=09044149;08284703;08340795;09044200;08299328;08284737;), [吕月美](http://202.201.7.11/kcms/detail/search.aspx?dbcode=CJFQ&sfield=au&skey=吕月美&code=09044149;08284703;08340795;09044200;08299328;08284737;), 等.(2010)电针治疗胆石症的临床观察.针灸临床杂志26:36-37.

[747] Diao Yong-hong, Han Xiu-hua, Ma Hua, Liang Wan-zeng, Lv Yue-mei , et al.(2010)Clinical Observation on Electro-acupuncture for Treatment of Cholelithiasis.Journal of Clinical Acupucnture and Moxibustion 26:36-37.

[748] 张彩侠, 孙远征.(2010)电针治疗慢性酒精中毒周围神经病的临床观察.针灸临床杂志26:38-39.

[748]Zhang Cai-xia, Sun Yuan-zheng.(2010)Clinical Observation on Electric acupuncture for Treatment of Chronic Alcoholism Peripheral

Neuropathy.Journal of Clinical Acupucnture and Moxibustion 26:38-39.

[749] 黄晓媛, 李崖雪, 孙申田.(2007)电针治疗尿道综合征25例临床研究.针灸临床杂志23:42-43.

[749]Huang Xiao-yuan , Li Ya-xue, Sun Sheng-tian.(2007)Clinical study on Electric acupuncture for Treating 25 cases of [urethral syndrome](http://dict.cnki.net/dict_result.aspx?searchword=尿道综合征&tjType=sentence&style=&t=urethral+syndrome).Journal of Clinical Acupucnture and Moxibustion 23:42-43.

[750] 谢菊英, 李志宏, 罗红缨, [李启秀](http://202.201.7.11/kcms/detail/search.aspx?dbcode=CJFQ&sfield=au&skey=李启秀&code=10235800;09209957;10605912;).(2007)电针治疗痛风性急性关节炎临床疗效观察.针灸临床杂志23:23-24.

[750]Xie Ju-ying, Li Zhi-hong, Luo Hong-ying, Li Qi-xiu.(2007)Therapeutic observation on the acute goute arthritis mainly treated with electric acupuncture.Journal of Clinical Acupucnture and Moxibustion 23:23-24.

[751] 吴建丽, 高维滨.(2010)电针治疗膝关节骨性关节炎临床观察.针灸临床杂志26:38-39.

[751]Wu Jian-li, Gao Wei-bin.(2010)Clinical Observation on Electric acupuncture for Treatment of Knee Osteoarthritis.Journal of Clinical Acupucnture and Moxibustion 26:38-39.

[752] 刘未艾, 吴清明, 李向荣, [谭涛](http://202.201.7.11/kcms/detail/search.aspx?dbcode=CJFQ&sfield=au&skey=谭涛&code=10975007;11185497;11061286;11052618;11126291;17512826;), [石学慧](http://202.201.7.11/kcms/detail/search.aspx?dbcode=CJFQ&sfield=au&skey=石学慧&code=10975007;11185497;11061286;11052618;11126291;17512826;), 等. (2008)电针治疗中风后便秘的疗效观察.针灸临床杂志24:17-18.

[752]Liu Wei-ai, Wu Qing-ming, Li Xiang-rong, Tan Tao, Shi Xue-hui, et al.(2008)Observation on therapeutic effect of Electric acupuncture on Constipation after apoplexy.Journal of Clinical Acupucnture and Moxibustion 24:17-18.

[753] 白桦, 于澎.(2010)电针治疗灼口综合征的临床疗效观察.针灸临床杂志26:28-29.

[753] Bai Hua, Yu Peng.(2010)Therapeutic observation on  [burning mouth syndrome](http://dict.cnki.net/javascript:showjdsw('showjd_0','j_0')) treated with electric acupuncture.Journal of Clinical Acupucnture and Moxibustion 26:28-29

[754] 王宽宇, 陈静, 孙晓龙, [张石军](http://202.201.7.11/kcms/detail/search.aspx?dbcode=CJFQ&sfield=au&skey=张石军&code=07354093;07356638;07362832;13963934;).(2007)电针足三里、上巨虚对腹部术后胃肠功能的影响.针灸临床杂志23:19-20.

[754]Wang Yu-kuan, Chen Jing, Sun Xiao-long, Zhang Shi-jun.(2007)The effects of manipulation skills treating gastroenteric disorders by electro-acupuncturing at "Zusanli" (ST 36) and "Shangjuxu" (ST 37) points.Journal of Clinical Acupucnture and Moxibustion 23:19-20

[755] 韩鹏, 胡新颖.(2010)调神养心针法治疗抑郁症的临床研究.针灸临床杂志, 26:20-21.

[755]Han Peng, Hu Xin-ying.(2010)Clinical study on “adjusted gods and nurshing heart [acupuncture method](http://dict.cnki.net/javascript:showjdsw('jd_t','j_'))”for treatment of [depression](http://dict.cnki.net/javascript:showjdsw('jd_t','j_')).Journal of Clinical Acupucnture and Moxibustion 26:20-21

[756] 王琼芬, 李飞, 李玲.(2008)非药物综合疗法治疗神经根型颈椎病的随机对照观察.针灸临床杂志.24:1-4.

[756]Wang Qiong-fen, Li Fei, Li Ling.(2008)Randomized controlled observation of treating the Cervical Spondylotic Radiculopauthy with the non-medical comprehensive therapy.Journal of Clinical Acupucnture and Moxibustion 24:1-4

[757] 欧阳颀, 魏智钧, 张春梅, [周巍](http://202.201.7.11/kcms/detail/search.aspx?dbcode=CJFQ&sfield=au&skey=周巍&code=20181107;20253720;20738004;20804649;).(2010)分期动静针刺治疗周围性面瘫临床研究.针灸临床杂志26:1-4.

[757] Ou Yang-qi, Wei Zhi-jun, Zhang Chun-mei, Zhou Wei.(2010)Effects of Staging Dynamic-Static Acupuncture on Patients with Peripheral Facial Paralysis.Journal of Clinical Acupucnture and Moxibustion 26:1-4

[758] 马玉琴, 金龙涛, 张健, [王小平](http://202.201.7.11/kcms/detail/search.aspx?dbcode=CJFQ&sfield=au&skey=王小平&code=07840215;), [王静](http://202.201.7.11/kcms/detail/search.aspx?dbcode=CJFQ&sfield=au&skey=王静&code=07840215;).(2008)分期循经针刺法对中风患者运动功能的影响研究.针灸临床杂志24:5-6.

[758] Ma Yu-qin, Jin Long-tao, Zhang Jian, Wang Xiao-pin, Wang Jing.(2008)Study on the effect of treatment for kinetic function of stroke patients by acupuncturing at different points of different stages.Journal of Clinical Acupucnture and Moxibustion 24:5-6.

[759] 熊同学. (2007)腹针治疗颈椎病疗效观察.针灸临床杂志23:14-15.

[759]Xiong Tong-xue.(2007)Therapeutic observation on [cervical spondylosis](http://dict.cnki.net/dict_result.aspx?searchword=颈椎病&tjType=sentence&style=&t=cervical+spondylosis) treated with [abdominal acupuncture](http://dict.cnki.net/dict_result.aspx?searchword=腹针&tjType=sentence&style=&t=abdominal+acupuncture).Journal of Clinical Acupucnture and Moxibustion 23:14-15.

[761] 盛益国,丁家祥.(2007)光电耳压针刺治疗单纯性肥胖临床研究.针灸临床杂志23:9-10.

[761]Sheng Yi-guo, Ding Jia-xiang.(2007) Clinical observation of simple obesity treated by light-electro acupuncture.Journal of Clinical Acupucnture and Moxibustion. 23:9-10.

[761] 盛益国, 丁家祥.(2007)光电耳压针刺治疗单纯性肥胖临床研究.针灸临床杂志23:9-10.

[761]Sheng Yi-guo, Ding Jia-xiang.(2007)Clinical observation of simple obesity treated by light-electro acupuncture.Journal of Clinical Acupucnture and Moxibustion 23:9-10.

[762] 李巍, 谭洛, 于波, [李普海](http://202.201.7.11/kcms/detail/search.aspx?dbcode=CJFQ&sfield=au&skey=李普海&code=09110584;08391268;09110612;08347461;08391048;08347486;), [陈健](http://202.201.7.11/kcms/detail/search.aspx?dbcode=CJFQ&sfield=au&skey=陈健&code=09110584;08391268;09110612;08347461;08391048;08347486;), 等.(2008)氦—氖激光穴位针刺治疗高脂血症临床研究.针灸临床杂志24:3-5.

[762]Li Wei, Tan Luo, Yu Bo, Li Pu-hai, Chen Jian, et al.(2008)Acupuncture on acupoints with helium- neon laser treating high blood- fat symptom.Journal of Clinical Acupucnture and Moxibustion 24:3-5.

[763] 王东岩, 董旭, 冯丽媛.(2010)合谷刺加电针对中风患者腕手功能重建的研究.针灸临床杂志26:16-17.

[763]Wang Dong-yan, Dong Xu, Fen Li-yuan.(2010)Study on [functional reconstruction](http://dict.cnki.net/dict_result.aspx?searchword=功能重建&tjType=sentence&style=&t=functional+reconstruction) of [wrist](http://dict.cnki.net/dict_result.aspx?searchword=腕&tjType=sentence&style=&t=wrist) and [hand](http://dict.cnki.net/dict_result.aspx?searchword=手&tjType=sentence&style=&t=hand) in Patients of apoplexy treated with [muscular needling](http://dict.cnki.net/dict_result.aspx?searchword=合谷刺&tjType=sentence&style=&t=muscular+needling).Journal of Clinical Acupucnture and Moxibustion 26:16-17.

[764] 杨立侠, 杨铭.(2009)合谷刺治疗肩周炎42例.针灸临床杂志25:32-33.

[764]Yang Li-xia, Yang Ming.(2009)Acupuncture Hegu point treatment with 48 cases of periarthritis of shoulder.Journal of Clinical Acupucnture and Moxibustion.25:32-33.

[765] 周贤华.(2004)火针治疗肱骨外上髁炎31例疗效观察.针灸临床杂志20:28-29.

[765] Zhou Xian-hua.(2004)Therapeutic observation on external humeral epicondylitis treated with fire needle.Journal of Clinical Acupucnture and Moxibustion 20:28-29

[766] 孙远征, 王春英, 黄梓平.(2007)降逆止咳汤配合针灸治疗胃食管反流性咳嗽30例疗效观察.针灸临床杂志23:21-22.

[766]Sun Yuan-zheng, Wang Chun-ying, Huang Zi-ping.(2007)Therapeutic observation on gastroesophageal reflux induced cough treated with acupucnture combined with “jiang ni zhi ke tang”.Journal of Clinical Acupucnture and Moxibustion 23:21-22.

[767] 苏秀贞, 郭芸倩.(2010)经络环皮部挑治法治疗小儿脑瘫的疗效观察.针灸临床杂志26:30-31.

[767]Su Xiu-zhen, Guo Yun-qian.(2010)The Observation of the Curative Effect on Babies' Cerebral Palsy by Pricking Therapy on Meridian Circle in Skin.Journal of Clinical Acupucnture and Moxibustion 2:30-31.

[768] 孙敬青, 张琳.(2010)老十针”为主治疗功能性消化不良临床观察.针灸临床杂志26:9-11.

[768]Sun Jing-qing, Zhang Lin.(2010)Clinical Observation of Functional Dyspepsia Treated with Acupuncture" The Old 10 - Needles"Journal of Clinical Acupucnture and Moxibustion 2:30-31.

[769] 成旭辉.(2010)龙虎交战针法配合温针灸治疗腰椎间盘突出症.针灸临床杂志26:38-39.

[769]Cheng Xu-hui.(2010)The Treatment of Lumbar Disc Herniation by Using Dragon and Tiger Fighting Acupuncture Combined with Warm Acupuncture.Journal of Clinical Acupucnture and Moxibustion 26:38-39.

[770] 王曙辉, 杨丽霞, 魏林林, [崔星](http://202.201.7.11/kcms/detail/search.aspx?dbcode=CJFQ&sfield=au&skey=崔星&code=10780847;11202211;06806581;15881292;23687888;), [彭志华](http://202.201.7.11/kcms/detail/search.aspx?dbcode=CJFQ&sfield=au&skey=彭志华&code=10780847;11202211;06806581;15881292;23687888;).(2010)捏脊结合针刺治疗糖尿病胃轻瘫35例.针灸临床杂志26:4-6.

[770]Wang Shu-hui, Yang Li-xia, Wei Lin-lin, Cui Xing, Peng Zhi-hua.(2010)Observation of Therapeutic Effect of Chiropractic Combing with Acupuncture on 35 Cases with Diabetic Gastroparesis.Journal of Clinical Acupucnture and Moxibustion 26:4-6.

[771] 张艳红, 魏凤阁.(2007)全程冷敷加巨刺治疗急性踝外侧韧带损伤35例.针灸临床杂志23: 18-19.

[771]Zhang Yan-hong, Wei Feng-ge.(2007)Thirty - five acute ankle joint lateral ligament injury cases cured by cold- pack through whole therapeutic course plusing opposite acupuncture.Journal of Clinical Acupucnture and Moxibustion 23:18-19.

[772] 廖钰, 张君幸, 冯雪芳, 等.(2009)雀啄灸法治疗膝骨性关节炎的临床研究.针灸临床杂志25:1-3.

[772] Liao Yu, Zhang Jun-xing, Feng Xue-fang.(2009)Clinical research on birdpecking moxibustion treatment with knee osteoarthritis.Journal of Clinical Acupucnture and Moxibustion 25:1-3.

[773] 何天有.(2010)“三位一体”针法治疗中风后遗症疗效观察.针灸临床杂志26:34-36.

[773] He Tian-you.(2010)The Clinical Observation on Treatment by "Trinity-acupuncture" in Sequela of Cerebral Apoplexy.Journal of Clinical Acupucnture and Moxibustion 26:34-36.

[774] 徐彦龙, 何天有.(2007)“三阴穴”配合“阴三穴”治疗慢性前列腺炎临床疗效观察.针灸临床杂志23:12-13.

[774]Xu Yan-long, He Tian-you.(2007)Therapeutic observation on [chronic prostatitis](http://dict.cnki.net/dict_result.aspx?searchword=慢性前列腺炎&tjType=sentence&style=&t=chronic+prostatitis) treated with “san yin xue”combined with “san yang xue”.Journal of Clinical Acupucnture and Moxibustion 23:12-13.

[775] 林桂君, F. Camará. (2007)神阙穴隔盐灸治疗儿童遗尿的临床观察.针灸临床杂志23:55-56.

[775]Lin Gui-jun, F. Camará.(2007)Clinical observations on the treatment of children's enuresis by acupuncture plus salt- partitioned moxibustion at Shenque.Journal of Clinical Acupucnture and Moxibustion 23:55-56.

[776] 杨军雄, 向开维. (2010)双手同究刺法的临床研.针灸临床杂志26:25-27.

[776]Yang Jun-xiong, Xiang Kai-wei.(2010)Clinical Study of Hand with the Thorns Law.Journal of Clinical Acupucnture and Moxibustion 26:25-27.

[777] 黄华超. (2010)速刺加药熨治疗急性期面瘫60例.针灸临床杂志26:57-58.

[777]Huang Hua-chao, (2010)The Treatment of 60 Cases of Acute Facial Paralysis with Speed Acupuncture Combine with Drug Ironing.Journal of Clinical Acupucnture and Moxibustion 26:57-58.

[778] 罗小林, 龚享文, 吴洪皓.(2010)天灸疗法对慢性支气管炎患者生命质量影响.针灸临床杂志26:48-50.

[778] Luo Xiao-lin, Gong Xiang-wen, Wu Hong-hao.(2010)The Effect on the Quality of Life through the Tian Moxibustion Treatment.Journal of Clinical Acupucnture and Moxibustion 26:48-50.

[779] 黄柳和, 孔令深, 练汉健, 罗健.(2002)挑罐法治疗退行性脊椎炎的临床研究.针灸临床杂志18:6-8.

[779]Huang Liu-he, kong Ling-sheng, Lian Han-jian, Luo Jian.(2002)Clinical observations on the treatment of [retrograde osteoarthritis](http://dict.cnki.net/dict_result.aspx?searchword=退行性脊椎炎&tjType=sentence&style=&t=retrograde+osteoarthritis) by tiao guan fa.Journal of Clinical Acupucnture and Moxibustion 18:6-8.

[780] 粟漩, 赖新生.(2010)通督调神法电针治疗脑卒中后吞咽障碍疗效观察.针灸临床杂志26:3-6.

[780]Su Xuan, Lai Xin-sheng.(2010)The Clinical Study on" Tongdutiaoshen"(An Acapuncture Treatment)for Treatment of Dysphagia after Stroke.Journal of Clinical Acupucnture and Moxibustion 26:3-6.

[781] 刘丽, 于学平, 李晓陵, [许宇飞](http://202.201.7.11/kcms/detail/search.aspx?dbcode=CJFQ&sfield=au&skey=许宇飞&code=07362807;07354127;07362797;07354116;07354089;24044760;), [王丰](http://202.201.7.11/kcms/detail/search.aspx?dbcode=CJFQ&sfield=au&skey=王丰&code=07362807;07354127;07362797;07354116;07354089;24044760;), 等.(2010)头部电针治疗抽动-秽语综合征临床疗效观察.针灸临床杂志26:21-22.

[781]Liu Li, Yu Xue-ping, Li Xiao-ling, Xu Yu-fei, Wang Feng, et al, (2010)Clinical observations on the treatment of [gilles de la tourette syndrome](http://dict.cnki.net/dict_result.aspx?searchword=抽动-秽语综合征&tjType=sentence&style=&t=gilles+de+la+tourette+syndrome) by the head on ischemic apop-lexy .Journal of Clinical Acupucnture and Moxibustion 26:21-22.

[782] 曹华, 王宇, 邢艳丽. (2010)头穴丛刺结合认知训练对轻度认知功能障碍的影响.针灸临床杂志26:4-6.

[782]Cao Hua, Wang Yu, Xing Yan-li.(2010)Cluster Needling of Scalp Point Therapy Combined with Cognitive Training on the Impact of Mild Cognitive Impairment.Journal of Clinical Acupucnture and Moxibustion 26:4-6.

[783] 孙善贤, 蔡国锋, 杜晓蕾, 邹伟.(2010)头穴配合三阳经腧穴针刺治疗脑梗死后偏瘫.针灸临床杂志26:48-49.

[783] Sun Shan-xian, Cai Guo-feng, Du Xiao-lei, Zhou Wei.(2010)[Scalp point](http://dict.cnki.net/dict_result.aspx?searchword=头穴&tjType=sentence&style=&t=scalp+point) combined with acupucnture of three yang meridians treat [hemiplegia](http://dict.cnki.net/dict_result.aspx?searchword=偏瘫&tjType=sentence&style=&t=hemiplegia) after [cerebral infarction](http://dict.cnki.net/dict_result.aspx?searchword=脑梗死&tjType=sentence&style=&t=cerebral+infarction).Journal of Clinical Acupucnture and Moxibustion 26:48-49.

[784] 夏阳, 朱天忠, 宋亚光, [杨林](http://202.201.7.11/kcms/detail/search.aspx?dbcode=CJFQ&sfield=au&skey=杨林&code=05982906;08784685;08784680;06428188;07842660;08117067;08784682;08784674;), [冯祯钰](http://202.201.7.11/kcms/detail/search.aspx?dbcode=CJFQ&sfield=au&skey=冯祯钰&code=05982906;08784685;08784680;06428188;07842660;08117067;08784682;08784674;), 等.(2004)头针结合CT定位治疗中风100例临床观察.针灸临床杂志20:4-6.

[784] Xiao Yang, Zhu Tian-zhong, Song Ya-guang, Yang Lin, Feng Zhen-yu, et al, (2004)Observation on therapeutic effect of 100 cases of apoplexy by scalp acupuncture and CT localization.Journal of Clinical Acupucnture and Moxibustion 20:4-6.

[785] 肖震心, (2010)头针结合体针治疗小脑性共济失调疗效观察.针灸临床杂志26:12-13.

[785] Xiao Zhen-xin.(2010)Observations on the Therapeutic Effect of Scalp-plus Body-acupuncture on Cerebellar Ataxia.Journal of Clinical Acupucnture and Moxibustion 26:12-13.

[786] 贺必梅, 李万瑶, 李国才, [秦凯](http://202.201.7.11/kcms/detail/search.aspx?dbcode=CJFQ&sfield=au&skey=秦凯&code=11458674;06923088;06835255;11317550;10726261;), [谢晓澜](http://202.201.7.11/kcms/detail/search.aspx?dbcode=CJFQ&sfield=au&skey=谢晓澜&code=11458674;06923088;06835255;11317550;10726261;), 等.(2006)头针麻醉对肠癌患者术后炎症反应的影响.针灸临床杂志22:5-7

[786]He Bi-mei, Li Wan-yao, Li Guo-cai, Qin Kai, Xie Xiao-lan, et al.(2006)Effects of head- acupuncture on post- operation inflammtory reaction of the intestine cancer patients.Journal of Clinical Acupucnture and Moxibustion 22:5-7.

[787] 李红艳, 沙丽丽, 李立峰.(2010)透刺对脑卒中后认知功能障碍干预效应的临床研究.针灸临床杂志26:19-20.

[787]Li Hong-yan, Sha Li-li, Li Li-feng.(2010)Clinical study on the [interventional effect](http://dict.cnki.net/dict_result.aspx?searchword=干预效应&tjType=sentence&style=&t=interventional+effect) of [cognitive impairment](http://dict.cnki.net/dict_result.aspx?searchword=认知功能障碍&tjType=sentence&style=&t=cognitive+impairment) after [stroke](http://dict.cnki.net/dict_result.aspx?searchword=脑卒中&tjType=sentence&style=&t=stroke) treated by [penetration needling](http://dict.cnki.net/dict_result.aspx?searchword=透刺&tjType=sentence&style=&t=penetration+needling) .Journal of Clinical Acupucnture and Moxibustion 26:19-20.

[788] 杨云玲.(1999)推拿配合He-Ne激光治疗周围性面瘫.针灸临床杂志15:12-13.

[788]Yang Yun-ling.(1999)[Massage](http://dict.cnki.net/dict_result.aspx?searchword=推拿&tjType=sentence&style=&t=massage) combined with [he-ne laser](http://dict.cnki.net/dict_result.aspx?searchword=He-Ne激光&tjType=sentence&style=&t=he-ne+laser) treat [peripheral facial paralysis](http://dict.cnki.net/dict_result.aspx?searchword=周围性面瘫&tjType=sentence&style=&t=peripheral+facial+paralysis).Journal of Clinical Acupucnture and Moxibustion 15;12-13.

[789] 张宏涛, 方晓丽.(2010)温通针法治疗颈肩臂综合征临床疗效观察.针灸临床杂志26:40-42.

[789]Zhang Hong-tao, Fang Xiao-li.(2010)Observations on the Clinical Efficacy of Warming-promotion Method of Acupuncture in Treating Neck and Shoulder-arm Syndrome.Journal of Clinical Acupucnture and Moxibustion 26:40-42.

[790] 徐凯, 刘永鑫, 刁鉴伟.(2010)温针阿是穴治疗跟痛症.针灸临床杂志26:39-41.

[790]Xu Kai, Liu Yong-xin, Diao Jian-wei.(2010)Clinical Research on Treating the Heel Pain with Warm Needling on Ashi-points.Journal of Clinical Acupucnture and Moxibustion 26:39-41.

[791] 王为凤, 陈妙根.(2010)温针灸分期治疗周围性面瘫临床观察.针灸临床杂志26:36-39.

[791] Wang Wei-feng, Chen Miao-gen.(2010)The Clinical Obversation Of Warm Acupuncture Treatment Applied To Peripheral Facial Paralysis By Stage.Journal of Clinical Acupucnture and Moxibustion 26:36-39.

[792] 周丽华, 章裕华, 李志豪.(2010)温针灸结合功能锻炼治疗肩周炎疗效观察.针灸临床杂志26:45-46.

[792]Zhou Li-hua, Zhang Yu-hua, Li Zhi-hao.(2010)Observations on the Efficacy of Warming Acupuncture combined with exercise of function in Treating scapulohumeral periarthritis.Journal of Clinical Acupucnture and Moxibustion 26:45-46.

[793] 姜忠华, (2004)温针灸为主治疗中老年骨性关节炎临床观察.针灸临床杂志20:40-41.

[793]Jiang Zhong-hua.(2004)Clinical observation of senium osteoarthritis treated with warm needle mainly.Journal of Clinical Acupucnture and Moxibustion 26:40-41.

[794] 陈兴华.(1998)温针灸治疗肩周炎76例疗效观察.针灸临床杂志14:11-12.

[794]Chen Xing-hua.(1998)Observations on the Therapeutic Effect of 76 cases of [periarthritis](http://dict.cnki.net/dict_result.aspx?searchword=肩周炎&tjType=sentence&style=&t=periarthritis) treated with warm needle. Journal of Clinical Acupucnture and Moxibustion 14:11-12.

[795] 张丽华.(2007)温针灸治疗周围性面瘫38例疗效观察.针灸临床杂志23:39.

[795]Zhang Li-hua.(2007)Observations on the Therapeutic Effect of 38 cases of Peripheral Facial Paralysis treated with warm needle. Journal of Clinical Acupucnture and Moxibustion 23:39.

[796] 戴祥珍.(1996)温针与TDP治疗颞下颌关节紊乱综合征94例疗效观察.针灸临床杂志12:47.

[796]Dai Xiang-Zhen.(1996)Observations on the Therapeutic Effect of 94 cases of [temporomandibular joint disturbance syndrome](http://dict.cnki.net/javascript:showjdsw('showjd_0','j_0')) treated with warm needle and TDP. Journal of Clinical Acupucnture and Moxibustion 12:47.

[797] 刘亚平, 刘树山, 马秀琴.(2007)消脂汤配合针灸治疗脂肪肝90例的临床观察.针灸临床杂志23:9-10.

[797] Liu Ya-ping, Liu Shu-shan, Ma Xiu-qin.(2007)Clinical observation of fatty liver treated with warm needle combined with Jiejiu Xiaozhi Decoction.Journal of Clinical Acupucnture and Moxibustion 23:9-10.

[798] 李迎真, 吕静, 韩纪琴, [安军明](http://202.201.7.11/kcms/detail/search.aspx?dbcode=CJFQ&sfield=au&skey=安军明&code=24733247;24733248;24733249;21196919;).(2010)小针刀配合手法治疗颈源性眩晕的疗效观察.针灸临床杂志26:26-27.

[798]Li Ying-zhen, Lv Jing, Han Ji-qin, An Jun-ming.(2010)Observations on the Therapeutic Effect of [dizziness caused from cervical](http://dict.cnki.net/dict_result.aspx?searchword=颈源性眩晕&tjType=sentence&style=&t=dizziness+caused+from+cervical) treated with small Needle Knife and Manipulation . Journal of Clinical Acupucnture and Moxibustion 26:26-27.

[799] 吴志刚, 左天伟.(2004)新设穴为主药物注入治疗椎动脉型颈椎病临床观察.针灸临床杂志20:29-30.

[799] Wu Zhi-gang, Zuo Tian-wei.(2004)Clinical observation of [cervical spondylosis of vertebral artery type](http://dict.cnki.net/javascript:showjdsw('showjd_0','j_0')) treated with drug-infusing therapy.Journal of Clinical Acupucnture and Moxibustion 20:29-30.

[800] 华云辉, 陈协辉, 张燕, 雷跃. (2010)醒脑开窍法联合靳三针对中风后遗症的临床疗效体会. 针灸临床杂志26:17-19.

[800]Hua Yun-hui, Chen Xie-hui, Zhang Yan, Lei Yue.(2010)Observations on the Clinical Efficacy of alternate resuscitating conbined with [jin three needles](http://dict.cnki.net/dict_result.aspx?searchword=靳三针&tjType=sentence&style=&t=jin+three+needles) in Treating [apoplectic sequela](http://dict.cnki.net/dict_result.aspx?searchword=中风后遗症&tjType=sentence&style=&t=apoplectic+sequela) .Journal of Clinical Acupucnture and Moxibustion 26:17-19.

[801]张静, 李玉竹, 庄礼兴. (2006)穴位埋线疗法为主治疗全身强直—阵挛型癫痫90例.针灸临床杂志22:8-10.

[801]Zhang Jing, Li Yu-zhu, Zhang Li-xing.(2006)Observation on therapeutic affect of 90 tonic - clonic epilepsy patients treated by catgut implantation therapy.Journal of Clinical Acupucnture and Moxibustion 22:8-10.

[801]闫继红. (2001)穴位埋线治疗单纯性肥胖症疗效观察.针灸临床杂志23:58-59.

[801]Yan Ji-hong.(2001)Therapeutic effects of simple corpulence treated with catgut implantation.Journal of Clinical Acupucnture and Moxibustion 23:58-59..

[803]罗卫平, 郭结妍, 黄红缨. (2010)穴位透刺配合隔姜灸治疗顽固性面瘫.针灸临床杂志26(05):12-13.

[803]Luo Wei-ping, Guo Jie-yan, Huang Hong-yin.(2010)Clinical Study on Acupuncture Combined with Ginger-separated Moxibustion for Treatment Obistinate Peripheral Facial Paralysis.Journal of Clinical Acupucnture and Moxibustion 26(05):12-13.

[804]刘俐, 杨斌, 刘明清, 黄启嵩. (2007)穴位注射加针刺、TDP照射治疗颞下颌关节功能紊乱.针灸临床杂志23:61-62.

[804]Liu Li, Yang Bin, Liu Ming-qing, Huang Qi-song.(2007)acupuncture and point injection and TDP [irradiation](http://dict.cnki.net/javascript:showjdsw('jd_t','j_')) treat [temporomandibular joint disturbance](http://dict.cnki.net/dict_result.aspx?searchword=颞下颌关节功能紊乱&tjType=sentence&style=&t=temporomandibular+joint+disturbance).Journal of Clinical Acupucnture and Moxibustion 23:61-62.

[805]孟培燕 , 吴绪荣. (2007)穴位注射加针灸推拿治疗椎动脉型颈椎病.针灸临床杂志23:30-31.

[805]Meng Pei-yan, Wu Xu-rong.(2007)acupuncture and point injection and [acupuncture](http://dict.cnki.net/dict_result.aspx?searchword=针灸&tjType=sentence&style=&t=acupuncture) and [manipulation](http://dict.cnki.net/dict_result.aspx?searchword=推拿&tjType=sentence&style=&t=manipulation) treat [cervical spondylosis of vertebral artery type](http://dict.cnki.net/dict_result.aspx?searchword=椎动脉型颈椎病&tjType=sentence&style=&t=cervical+spondylosis+of+vertebral+artery+type).Journal of Clinical Acupucnture and Moxibustion 23:30-31.

[806]陈珊. (2010)穴位注射治疗膝关节顽固性冷痛.针灸临床杂志26:13.

[806]Chen Shan.(2010))acupuncture and point injection treats [crymodynia](http://dict.cnki.net/dict_result.aspx?searchword=冷痛&tjType=sentence&style=&t=crymodynia) of [knee joint](http://dict.cnki.net/dict_result.aspx?searchword=膝关节&tjType=sentence&style=&t=knee+joint).Journal of Clinical Acupucnture and Moxibustion 26:13.

[807]金瑛, 聂俊, 汪军华, [王爱君](http://202.201.7.11/kcms/detail/search.aspx?dbcode=CJFQ&sfield=au&skey=%CD%F5%B0%AE%BE%FD&code=09437076;10298537;11451155;11516194;11701382;), [徐媛媛](http://202.201.7.11/kcms/detail/search.aspx?dbcode=CJFQ&sfield=au&skey=%D0%EC%E6%C2%E6%C2&code=09437076;10298537;11451155;11516194;11701382;). (2006)压灸百会结合刺络放血治疗脑损伤后综合征38例总结.针灸临床杂志22:43-44.

[807]Jin Ying, Nie Jun, Wang Jun-hua, Wang Ai-jun, Xu Yuan-yuan.(2006)A report on 38 cases of [post cerebral injury syndrome](http://dict.cnki.net/dict_result.aspx?searchword=脑损伤后综合征&tjType=sentence&style=&t=post+cerebral+injury+syndrome) treated with Compressive Moxibustion on Baihui and [blood-letting puncture](http://dict.cnki.net/dict_result.aspx?searchword=刺络放血&tjType=sentence&style=&t=blood-letting+puncture).Journal of Clinical Acupucnture and Moxibustion 22:43-44.

[808]周庆翀, 刘署鹏, 范郁山. (2010)沿皮浅刺法治疗小儿多动症41例.针灸临床杂志26:38-39.

[808]Zhou Qing-xu, Liu Shu-peng, Fan Yu-shan.(2010)41 cases of Infantile Attention-deficit Hyperactivity Disorder treated with a methed that Along the skin acupuncture.Journal of Clinical Acupucnture and Moxibustion 26:38-39.

[809]王鹏琴, 李敬林 , 王健. (2007)眼针对急性脑梗死患者神经功能缺损及血清C-反应蛋白水平的影响.针灸临床杂志23:23-24.

[809]Wang Peng-qin, Li Jing-lin, Wang Jian.(2007)Influences of eye needles in acute brain stem dead patient of nerve damage-function and in blood serum C-response protein level.Journal of Clinical Acupucnture and Moxibustion 23:23-24.

[810]冯卫星, 闫咏梅. (2010)眼针对脑梗塞患者神经功能缺损程度和Barthel指数的影响.针灸临床杂志26:7-8.

[810]Feng Wei-xing, Yan Yong-mei.(2010)The Effect of Eye-Acupuncture on the Degree of Neurological Impairment and the Barthel Index of Cerebral Infarction Patients.Journal of Clinical Acupucnture and Moxibustion 26:7-8.

[811]吴笛. (1999)眼针配头针加电治疗脑血管意外后尿失禁40例.针灸临床杂志15:33-34

[811]Wu Di.(1999)40 cases of urinary incontinence due to cerebrovascular accident treated with eye acupuncture combined with [scalp acupuncture](http://dict.cnki.net/dict_result.aspx?searchword=头针&tjType=sentence&style=&t=scalp+acupuncture).Journal of Clinical Acupucnture and Moxibustion 15:33-34.

[812]陈仁年 , 陈永斌. (2010)抑制法针刺阿是穴为主治疗腰肌劳损.针灸临床杂志26:13-15.

[812]Chen Ren-nian, Chen Yong-bin.(2010)[Inhibition method](http://dict.cnki.net/dict_result.aspx?searchword=抑制法&tjType=sentence&style=&t=inhibition+method) and Ashi Point of Needle Splinter treat [lumbar muscle strain](http://dict.cnki.net/dict_result.aspx?searchword=腰肌劳损&tjType=sentence&style=&t=lumbar+muscle+strain).Journal of Clinical Acupucnture and Moxibustion.26:13-15.

[813]胡忠根, 张全爱, 朱雄心, 等. (2008)银质针治疗腰突症手术后遗症临床观察.针灸临床杂志24:4-5.

[813]Hu Zhong-ying, Zhang Quan-ai, Zhu Xiong-xin, et al.(2008)clinical randomized control observation on the sequelae of lumbar disc herniation operation treated by silver needles.Journal of Clinical Acupucnture and Moxibustion 24:4-5.

[814]张勇, 朱永志, 贺兴欢. (2010)荥合配穴加常规取穴治疗面神经炎疗效观察.针灸临床杂志26:15-16.

[814]Zhang Yong, Zhu Yong-zhi, He Xing-huan.(2010)An Observation of the Clinical Effect of Facial Neuritis Treated with Brook-sea Point Combined with Conventional Acupuncture.Journal of Clinical Acupucnture and Moxibustion 26:15-16.

[815]宋兴武. (2009)涌泉穴对针刺结合牵引治疗腰椎间盘突出症增效作用的临床观察.针灸临床杂志25:31-33.

[815]Sun Xing-wu.(2009))Clinical observation on the [synergism](http://dict.cnki.net/dict_result.aspx?searchword=增效作用&tjType=sentence&style=&t=synergism) of [lumbar disc herniation](http://dict.cnki.net/dict_result.aspx?searchword=腰椎间盘突出症&tjType=sentence&style=&t=lumbar+disc+herniation) treated by acupuncture combined with [traction treatment](http://dict.cnki.net/dict_result.aspx?searchword=牵引治疗&tjType=sentence&style=&t=traction+treatment).Journal of Clinical Acupucnture and Moxibustion 25:31-33.

[816]赵立刚, 马莉, 李亚杰, [程为平](http://search.cnki.com.cn/Search.aspx?q=author:程为平), [吴茜](http://search.cnki.com.cn/Search.aspx?q=author:吴茜). (2007)针刺百会、大椎治疗老年性痴呆的疗效观察.针灸临床杂志 23:42-43.

[816]Zhao Li-gang, Ma Li, Li Ya-jie, ChengWei-ping, Wu Qian.(2007))An observation of the Clinical Effect of [senile dementia](http://dict.cnki.net/dict_result.aspx?searchword=老年性痴呆&tjType=sentence&style=&t=senile+dementia) Treated with acupunture of baihui、dazhui.Journal of Clinical Acupucnture and Moxibustion 23:42-43.

[817]乔颖欣, 程为平. (2007)针刺百会”、神门”治疗抑郁症的临床研究.针灸临床杂志23:52-54.

[817]Qiao Ying-xin, Chen Wei-ping.(2007)Observations on the Clinical Efficacy of [depression](http://dict.cnki.net/dict_result.aspx?searchword=抑郁症&tjType=sentence&style=&t=depression) treated with acupunture of baihui、dazhui .Journal of Clinical Acupucnture and Moxibustion 23:52-54.

[818]王亚南, 郑祖艳. (2010)针刺背俞穴为主治疗焦虑性神经症的临床疗效研究.针灸临床杂志26:13-14.

[818]Wang Ya-nan, Zheng Zu-yan.(2010)The Clinical Curative Effect Research of Acupuncture on Back-shu Points Primarily to Treat the Anxious Neurosis.Journal of Clinical Acupucnture and Moxibustion 26:13-14.

[819]杨火祥. (2009)针刺触激脊神经根术在腰椎间盘突出症中的应用.针灸临床杂志25:18-19.

[819]Yang Huo-xiang.(2009)Application of needling-touching spinal nerve root methods in lumbar intervertebral disc protrusion (LIDP).Journal of Clinical Acupucnture and Moxibustion 25:18-19.

[820]徐凡, 白秀荣 , 冉春风. (1995)针刺华佗夹脊穴治疗脊髓性截瘫临床研究.针灸临床杂志11:13-14.

[820]Xu Fan, Bai Xiu-rong, Ran Chun-feng.(1995)The Clinical Curative Effect Research of [spinal paraplegia](http://dict.cnki.net/dict_result.aspx?searchword=脊髓性截瘫&tjType=sentence&style=&t=spinal+paraplegia) treated with acupunture of Hua Tuo Jia Ji.Journal of Clinical Acupucnture and Moxibustion 11:13-14.

[821]李永义, 杨松堤, 于学平, [黄玉晶](http://search.cnki.com.cn/Search.aspx?q=author:黄玉晶). (2010)针刺箕门、气海穴治疗神经源性尿潴留疗效观察.针灸临床杂志26:31-32.

[821]Li Yong-yi, Yang Sun-ti, Yu Xue-ping, Huang Yu-jing.(2010)Observations on the Efficacy of [neurogenic](http://dict.cnki.net/dict_result.aspx?searchword=神经源性&tjType=sentence&style=&t=neurogenic) [urinary retention](http://dict.cnki.net/dict_result.aspx?searchword=尿潴留&tjType=sentence&style=&t=urinary+retention) treated with acupunture of [qihai](http://dict.cnki.net/dict_result.aspx?searchword=气海穴&tjType=sentence&style=&t=qihai(r6)) and [point sp](http://dict.cnki.net/dict_result.aspx?searchword=箕门穴&tjType=sentence&style=&t=point+sp+11) .Journal of Clinical Acupucnture and Moxibustion 26:31-32.

[822]孙景胜, 陈先风, 马永军. (1993)针刺加穴位注射治疗类风湿性关节炎126例临床观察.针灸临床杂志9:21-22.

[822]Sun Jing-sheng, Chen Xian-feng, Ma Yong-jun.(1993))An observation of the Clinical Effect of 126 cases of [rheumatoid arthritis](http://dict.cnki.net/dict_result.aspx?searchword=类风湿性关节炎&tjType=sentence&style=&t=rheumatoid+arthritis) Treated with acupunture combined with point injection .Journal of Clinical Acupucnture and Moxibustion 9:21-22.

[823]白震民, 王可, 崔海. (2010)针刺结合砭石治疗青少年假性近视的疗效观察.针灸临床杂志26:23-24.

[823]Bai Zheng-ming, Wang Ke, Cui Hai.(2010)The Clinical Observation of Treating Teenagers' Pseudomyopia by Acupuncture Combined with Stone Needle.Journal of Clinical Acupucnture and Moxibustion 26:23-24.

[824]覃彪民, 老锦雄. (2006)针刺结合关节松动术治疗肩周炎的临床观察.针灸临床杂志22:14-15.

[824]Qin Biao-ming, Lao Jin-xiong.(2006)Clinical observation of acupuncture combined with joints loosening therapy for treatment of scapulonhumeral periarthritis.Journal of Clinical Acupucnture and Moxibustion.22:14-15.

[825]李国辉, 陈惠, 何宇峰, [谭吉林](http://www.cqvip.com/Main/Search.aspx?w=谭吉林), [苏秀群](http://www.cqvip.com/Main/Search.aspx?w=苏秀群), 等. (2006)针刺结合减重支持系统训练对脑卒中患者平衡功能的影响.针灸临床杂志22:11-13.

[825]Li Guo-hui, Chen hui, He Yu-feng, Tan Ji-lin, Su Xiu-qun, et al.(2006)Investigation on the effect of needling combined with partial body weight support system (PBWS) training on the balance function of stroke patients.Journal of Clinical Acupucnture and Moxibustion 22:11-13.

[826]梁谊深, 谢胜, 冯金娟. (2010)针刺结合穴位注射治疗功能性便秘.针灸临床杂志26:29-30.

[826]Liang Yi-sheng, Xie Sheng, Feng Jin-juan.(2010)Acupuncture and point injection treats [functional constipation](http://dict.cnki.net/dict_result.aspx?searchword=功能性便秘&tjType=sentence&style=&t=functional+constipation).Journal of Clinical Acupucnture and Moxibustion 26:29-30.

[827]纪艳华, 王霆. (2009)针刺结合中药熏洗治疗肛肠病术后疼痛.针灸临床杂志25:14-15.

[827]Ji Yan-hua, Wang Ting.(2009)Treating Anorectal Pain after Surgery by Acupuncture Combined with Chinese Medicine Steaming.Journal of Clinical Acupucnture and Moxibustion 25:14-15.

[828]曹玲 , 王顺. (2007)针刺颈部夹脊穴对颈源性高血压患者血浆内皮素影响的观察.针灸临床杂志23:9-10.

[828]Cao Lin, Wang Shun.(2007)Effect of acupuncture with cervical-jiaji pointson endothelin in the patient of cervical hypertension.Journal of Clinical Acupucnture and Moxibustion 23:9-10.

[829]张小峰. (2007)针刺联合推拿治疗颈性眩晕188例.针灸临床杂志23:8-9.

[829]Zhang Xiao-feng.(2007)Treatment of cervical vertigo with manipulations combined with acupuncture.Journal of Clinical Acupucnture and Moxibustion 23:8-9.

[830]王峰. (2009)针刺量化治疗腰椎间盘突出症60例临床研究.针灸临床杂志25:6-7.

[830]Wang Febg.(2009)An observation of the clinical effect of 60 cases of [lumbar disc herniation](http://dict.cnki.net/dict_result.aspx?searchword=腰椎间盘突出症&tjType=sentence&style=&t=lumbar+disc+herniation) Treated with [quantizat](http://dict.cnki.net/dict_result.aspx?searchword=量化&tjType=sentence&style=&t=quantization)ed [acupuncture](http://dict.cnki.net/dict_result.aspx?searchword=针刺&tjType=sentence&style=&t=acupuncture).Journal of Clinical Acupucnture and Moxibustion 25:6-7.

[831]刘丕弘, 张丹琦, 张素冰, 封雪, 赵晶, 等. (2010)针刺麻醉复合颈丛神经阻滞用于甲状腺手术的临床观察.针灸临床杂志26:28-29.

[831]Liu Pi-hong, Zhang Dan-qi, Zhang Su-bing, Feng Xue, Zhao Jing, et al.(2010)An observation of the clinical effect of acupuncture anesthesia used for[thyroid surgery](http://dict.cnki.net/dict_result.aspx?searchword=甲状腺手术&tjType=sentence&style=&t=thyroid+surgery) .Journal of Clinical Acupucnture and Moxibustion 26:28-29.

[832]史永奋, 王丹华. (2005)针刺内关公孙为主治疗术后胃瘫综合征疗效观察.针灸临床杂志21:44-45.

[832]Shi Yong-fen, Wang Dan-hua.(2005)An observation of the clinical effect of [postsurgical gastroparesis synodrom](http://dict.cnki.net/dict_result.aspx?searchword=术后胃瘫综合征&tjType=sentence&style=&t=postsurgical+gastroparesis+synodrom) treated with acupuncture Neiguan(PC 6) and Gongsun(SP 4) .Journal of Clinical Acupucnture and Moxibustion 21:44-45.

[833]曾学清, 滕东时, 杨涛, [何小花](http://search.cnki.com.cn/Search.aspx?q=author:何小花), [隋晓亮](http://search.cnki.com.cn/Search.aspx?q=author:隋晓亮), 等. (2005)针刺配合康复训练治疗脑梗塞后构音障碍30例.针灸临床杂志21:9-10.

[833]Ceng Xue-qing, Teng Dong-shi, Yang Tao, He Xiao-hua, Sui Xiao-liang, et al.(2005)[Acupuncture](http://dict.cnki.net/dict_result.aspx?searchword=针刺&tjType=sentence&style=&t=acupuncture) combined with [rehabilitation training](http://dict.cnki.net/dict_result.aspx?searchword=康复训练&tjType=sentence&style=&t=rehabilitation+training) treat [dysarthria](http://dict.cnki.net/dict_result.aspx?searchword=构音障碍&tjType=sentence&style=&t=dysarthria) after [cerebral infarction](http://dict.cnki.net/dict_result.aspx?searchword=脑梗塞&tjType=sentence&style=&t=cerebral+infarction).Journal of Clinical Acupucnture and Moxibustion 21:9-10.

[834]朱崇田, 石娜. (2009)针刺配合康复治疗脑损伤后吞咽障碍.针灸临床杂志25:12-13.

[834]Zhu Chong-tian, Shi Na.(2009)[Acupuncture](http://dict.cnki.net/dict_result.aspx?searchword=针刺&tjType=sentence&style=&t=acupuncture) combined with [rehabilitation training](http://dict.cnki.net/dict_result.aspx?searchword=康复训练&tjType=sentence&style=&t=rehabilitation+training) treat [dysphagia](http://dict.cnki.net/dict_result.aspx?searchword=吞咽障碍&tjType=sentence&style=&t=dysphagia) after [brain injury](http://dict.cnki.net/dict_result.aspx?searchword=脑损伤&tjType=sentence&style=&t=brain+injury).Journal of Clinical Acupucnture and Moxibustion 25:12-13.

[835]王栋 , 林宪军. (2010)针刺配合脐疗法治疗慢性疲劳综合征.针灸临床杂志26:29-30.

[835]Wang Dong, Lin Xian-jun.(2010)[Acupuncture](http://dict.cnki.net/dict_result.aspx?searchword=针刺&tjType=sentence&style=&t=acupuncture) combined with chinese medicine on umbilicus treat [chronic fatigue syndrome](http://dict.cnki.net/dict_result.aspx?searchword=慢性疲劳综合征&tjType=sentence&style=&t=chronic+fatigue+syndrome).Journal of Clinical Acupucnture and Moxibustion 26:29-30.

[836]范娥. (2009)针刺配合牵引治疗腰椎间盘突出症.针灸临床杂志25:17-18.

[836]Fan E.(2009)Observation on Therapeutic Effect of Acupuncture Combined with Inverted Traction in Lumbar Intervertebral Disc Herniation.Journal of Clinical Acupucnture and Moxibustion 25:17-18.

[837]梁慧. (2010)针刺配合穴位贴敷治疗中风后肩手综合征30例.针灸临床杂志26:15-16.

[837]Liang Hui.(2010)[Acupuncture](http://dict.cnki.net/dict_result.aspx?searchword=针刺&tjType=sentence&style=&t=acupuncture) combined with [point-application](http://dict.cnki.net/dict_result.aspx?searchword=穴位贴敷&tjType=sentence&style=&t=point-application) treat 30 cases of [shoulder-hand syndrome](http://dict.cnki.net/dict_result.aspx?searchword=肩手综合征&tjType=sentence&style=&t=shoulder-hand+syndrome) .Journal of Clinical Acupucnture and Moxibustion 26:15-16.

[838]叶莉, 朱士涛. (1997)针刺配合穴位注射治疗三叉神经痛30例.针灸临床杂志13:21-22.

[838]Ye Li, Zhu Shi-tao.(1997)Acupuncture Point Injection with 30 Cases of Treatment of Trigeminal Neuralgia.Journal of Clinical Acupucnture and Moxibustion 13:21-22.

[839]杨璀健, 郑利群. (2010)针刺配合药物离子导入治疗急性面神经炎.针灸临床杂志26:14-16.

[839]Yang Cui-jian, Zheng Li-qun.(2010)[Acupuncture](http://dict.cnki.net/dict_result.aspx?searchword=针刺&tjType=sentence&style=&t=acupuncture) combined with [drug-iontophoresis](http://dict.cnki.net/dict_result.aspx?searchword=药物离子导入&tjType=sentence&style=&t=drug-iontophoresis) treat [acute face neuritis](http://dict.cnki.net/dict_result.aspx?searchword=急性面神经炎&tjType=sentence&style=&t=acute+face+neuritis) .Journal of Clinical Acupucnture and Moxibustion 26:14-16.

[840]孙远征, 杨密, 王薇. (2010)针刺配合药物熏蒸法治疗神经根型颈椎病.针灸临床杂志26:25-27.

[840]Sun Yuan-zheng, Yang Mi, Wang Wei.(2010)The Treatment of Nerve Root Cervical Spondylosis by Acupuncture and Chinese Medicine Fumigation.Journal of Clinical Acupucnture and Moxibustion 26:25-27.

[841]尹伦辉. (2005)针刺配合正脊推拿及音乐疏导治疗慢性疲劳综合征的对照观察.针灸临床杂志 08:9-10.

[841]Yi Lun-hui.(2005)Comparative observation on therapeutic effects on acupuncture combined with massage and music for treatment of chronic fatigue syndrome.Journal of Clinical Acupucnture and Moxibustion 08:9-10.

[842]周淑英, 余雅璇, 范洪力. (1996)针刺配穴注胎盘组织液治疗痤疮50例疗效对比观察.针灸临床杂志12:33-34.

[842]Zhou Shu-ying, Yu Ya-xuan, Fan Hong-li.(1996)Comparative observation on therapeutic effects on acupuncture combined with point injection of [placenta](http://dict.cnki.net/dict_result.aspx?searchword=胎盘&tjType=sentence&style=&t=placenta) [tissue fluid](http://dict.cnki.net/dict_result.aspx?searchword=组织液&tjType=sentence&style=&t=tissue+fluid) for treatment of [acne](http://dict.cnki.net/dict_result.aspx?searchword=痤疮&tjType=sentence&style=&t=acne).Journal of Clinical Acupucnture and Moxibustion 12:33-34.

[843]尹建平, 金远林, 王海燕. (2008)针刺四神聪治疗颈性眩晕40例.针灸临床杂志24:26-27.

[843] Yi Jian-ping, Jin Yuan-lin, Wang Hai-yan.(2008) Acupuncture Sishencong(EX-HN) with 40 Cases of Treatment of [Cervical Vertigo](http://dict.cnki.net/dict_result.aspx?searchword=颈性眩晕&tjType=sentence&style=&t=cervical+vertigo) .Journal of Clinical Acupucnture and Moxibustion 24:26-27.

[844]周忠亮. (2009)针刺推拿治疗膝关节骨性关节炎疗效观察.针灸临床杂志25:15-16.

[844]Zhou Zhong-liang.(2009)Clinical Observation on Knee Osteoarthritis Treated by Acupuncture Combined with Massage.Journal of Clinical Acupucnture and Moxibustion 25:15-16.

[845]王冰梅, 马建, 马莉. (2010)针刺为主治疗糖尿病周围神经病变34例.针灸临床杂志26:17-18.

[845]Wang Bin-mei, Ma Jian, Ma Li.(2010)A Clinical Analysis of the Curative Effect of Acupuncture onDiabetes Complicated by Around Neuropathological Changes.Journal of Clinical Acupucnture and Moxibustion 26:17-18.

[846]程坤, 颜红, 段可杰. (2008)针刺为主综合治疗焦虑症32例疗效观察.针灸临床杂志24:29-30.

[846]Cheng Kun, Yan Hong, Duan Ke-jie.(2008)32 cases of anxiety syndwme treated by needling mainly synthesisly.Journal of Clinical Acupucnture and Moxibustion 24:29-30.

[847]吴军. (2009)针刺为主综合治疗面神经麻痹30例.针灸临床杂志25:18-19.

[847]Wu Jun.(2009)30 cases of [facial paralysis](http://dict.cnki.net/dict_result.aspx?searchword=面神经麻痹&tjType=sentence&style=&t=facial+paralysis) treated by needling mainly synthesisly.Journal of Clinical Acupucnture and Moxibustion 25:18-19.

[848]唐晓文. (2009)针刺穴位加贴敷法治疗乳腺增生病疗效观察.针灸临床杂志25:5-7.

[848]Tang Xiao-wen.(2009)Observation of Effectiveness of Acupuncture Point and Sticking Therapy on Hyperplasia of Mammary Glands.Journal of Clinical Acupucnture and Moxibustion 25:5-7.

[849]李和平, 关春燕. (2008)针刺优选法结合标准分期与康复治疗脑卒中偏瘫.针灸临床杂志24:3-5.

[849]Li He-ping, Guan Chun-yan.(2008)Clinical observation on rehabilitation therapy effects of acupoint careful selection methods combined with standard staging sys-tem on apoplectic hemiplegia patients.Journal of Clinical Acupucnture and Moxibustion 24:3-5.

[850]芦冬梅. (1993)针刺与静滴催产素治疗产妇继发性宫缩乏力疗效对照观察.针灸临床杂志9:24-25.

[850]Lu Dong-mei.(1993)Comparative observation on therapeutic effects on acupuncture combined with [intravenously guttae oxytocin](http://dict.cnki.net/dict_result.aspx?searchword=静滴催产素&tjType=sentence&style=&t=intravenously+guttae+oxytocin) for treatment of deutetopathy uterine contraction hypodynamia.Journal of Clinical Acupucnture and Moxibustion 9:24-25.

[851]伍志浩, 闫继红. (2007)针刺与药物并用治疗肩周炎疗效观察.针灸临床杂志23:27-28.

[851]Wu Zhi-hao, Yan Ji-hong.(2007)Observation of Effectiveness of Acupuncture and Drugs on [Scapulohumeral Periarthritis](http://dict.cnki.net/dict_result.aspx?searchword=肩周炎&tjType=sentence&style=&t=scapulohumeral+periarthritis).Journal of Clinical Acupucnture and Moxibustion 23:27-28.

[852]李岩, 赵桂君, 陈英华, 沙丽丽, 魏冰, 等. (2010)针刺治疗代谢综合征50例.针灸临床杂志26:18-20.

[852]Li Yan, Zhao Gui-jun, Chen Ying-hua, Sha Li-li, Wei Bing, et al.(2010)32 cases of [metabolic](http://dict.cnki.net/dict_result.aspx?searchword=代谢综合征&tjType=sentence&style=&t=metabolic+syndrome)  syndwme treated by needling.Journal of Clinical Acupucnture and Moxibustion 26:18-20.

[853]苏云海, 麻红. (2007)针刺治疗短暂性脑缺血发作的临床观察.针灸临床杂志23:14-15.

[853]Su Yun-hai, Ma Hong.(2007)Clinical observation on transient ischemic attack treated by acupuncture.Journal of Clinical Acupucnture and Moxibustion 23:14-15.

[854]张晓彤, 强宝全. (1998)针刺治疗多发脑梗塞导致老年痴呆74例临床观察.针灸临床杂志14:7-8.

[854]Zhang Xiao-tong, Qiang Bao-quan.(1998)Clinical observation on 74 cases of multiple cerebral infarction dementai treated by acupuncture.Journal of Clinical Acupucnture and Moxibustion 14:7-8.

[855]叶运英. (2008)针刺治疗功能失调性子宫出血的疗效观察.针灸临床杂志24:14-15.

[855]Ye Yun-ying, (2008)Observation on the effect of acupuncture in treating parafunction metrorrhagia.Journal of Clinical Acupucnture and Moxibustion 24:14-15.

[856]李冬梅, 白鹏, 赵吉平. (2009)针刺治疗急性变态反应性接触性皮炎28例.针灸临床杂志25:3-5.

[856]Li Dong-mei, Bai Peng, Zhao Ji-ping.(2009)28 cases patients of acute allergic contact dermatitis treated by acupuncture.Journal of Clinical Acupucnture and Moxibustion 25:3-5.

[857]吴绪平, 罗惠平, 黄伟, [吴洪阳](http://202.201.7.11/kcms/detail/search.aspx?dbcode=CJFQ&sfield=au&skey=%CE%E2%BA%E9%D1%F4&code=07410448;22768674;22930478;22891316;), [王述菊](http://202.201.7.11/kcms/detail/search.aspx?dbcode=CJFQ&sfield=au&skey=%CD%F5%CA%F6%BE%D5&code=07410448;22768674;22930478;22891316;). (2008)针刺治疗急性脑梗死临床观察.针灸临床杂志24:1-2.

[857]Wu Xu-ping, Luo Hui-ping, Huang Wei, Wu Hong-yang, Wang Shu-ju.(2008)The clinical observation of acute cerebral infarction patients by acupuncture treatment.Journal of Clinical Acupucnture and Moxibustion 24:1-2.

[858]陈啸峰. (2009)针刺治疗急性期周围性面瘫55例.针灸临床杂志25:37-38.

Cheng Xiao-feng.(2009)Acupuncture treats 55 cases of peripheral facial paralysis.Journal of Clinical Acupucnture and Moxibustion 25:37-38.

[858][859]史朝霞. (2006)针刺治疗假性球麻痹48例疗效观察.针灸临床杂志22:11-12.

Shi Zhao-xia.(2006)Observation on therapeutic effect of acupuncture for treatment of pseudobulbar palsy.Journal of Clinical Acupucnture and Moxibustion 22:11-12.

[860]董妙先. (2007)针刺治疗偏头痛50例临床观察.针灸临床杂志23:5-6.

[860]Dong Miao-xian.(2007)The clinical observation of 50 cases of [migraine](http://dict.cnki.net/dict_result.aspx?searchword=偏头痛&tjType=sentence&style=&t=migraine) by acupuncture treatment.Journal of Clinical Acupucnture and Moxibustion 23:5-6.

[861] 韩鹏, 胡新颖. (2010)针刺治疗偏头痛的疗效及对TCD影响的临床研究. 针灸临床杂志, 2010, 26:22-23.

[861]Han Peng, Hu Xin-ying.(2010)The clinical study of [migraine](http://dict.cnki.net/dict_result.aspx?searchword=偏头痛&tjType=sentence&style=&t=migraine) by acupuncture treatment and the influence on TCD.Journal of Clinical Acupucnture and Moxibustion, 2010, 23:5-6.

[862] 王谅, 谢媛. (2006)针刺治疗青春期精神分裂症的临床研究. 针灸临床杂志, 2006, 22 :12-14.

[862]Wang Liang, Xie Yuan.(2006)Clinical study on the hebetic schizophrenia by acupuncture.Journal of Clinical Acupucnture and Moxibustion , 2010, 22:12-14.

[863] 罗高国, 郭新侠, 陈改娟. (2010)针刺治疗球麻痹所致言语困难30例. 针灸临床杂志, 2010, 26:11-12.

[863]Luo Gao-guo, Guo Xin-xia, Chen Gai-juan.(2010)Acupuncture treats 30 cases of [dyslalia](http://dict.cnki.net/dict_result.aspx?searchword=言语困难&tjType=sentence&style=&t=dyslalia) caused by [bulbar paralysis](http://dict.cnki.net/dict_result.aspx?searchword=球麻痹&tjType=sentence&style=&t=bulbar+paralysis).Journal of Clinical Acupucnture and Moxibustion , 2010, 26:11-12.

[864] 王欣. (2008)针刺治疗失眠50例. 针灸临床杂志, 2008, 24:14-15.

[864]Wang Xing.(2008)Acupuncture treating insomnia.Journal of Clinical Acupucnture and Moxibustion, 2008.24:14-15.

[865] 罗仁瀚, 徐凯, 周杰. (2008)针刺治疗失眠症的临床研究. 针灸临床杂志, 2008, 24:5-6.

[865]Luo Ren-han, Xu Kai, Zhou Jie.(2008)Clinical study of acupuncture treating patients treated with insomnia.Journal of Clinical Acupucnture and Moxibustion , 24:5-6.

[866] 任建军. (2007)针刺治疗糖尿病周围神经病变30例. 针灸临床杂志, 2007, 23 :26-27.

[866]Ren Jian-jun.(2007)30 cases of [diabetic peripheral neuropathy](http://dict.cnki.net/dict_result.aspx?searchword=糖尿病周围神经病变&tjType=sentence&style=&t=diabetic+peripheral+neuropathy) treated with acupuncture.Journal of Clinical Acupucnture and Moxibustion , 23:26-27.

[867] 强宝全. (2009)针刺治疗糖尿病周围神经病变40例. 针灸临床杂志, 2009, 25:25-26.

[867] Qiang Bao-quan.(2009)04 cases of [diabetic peripheral neuropathy](http://dict.cnki.net/dict_result.aspx?searchword=糖尿病周围神经病变&tjType=sentence&style=&t=diabetic+peripheral+neuropathy) treated with acupuncture.Journal of Clinical Acupucnture and Moxibustion , 25:25-26.

[868] 蒋宝龙, 孙远征. (2008)针刺治疗特发性震颤临床疗效观察. 针灸临床杂志, 2008, 24:22-23.

[868]Jiang Bao-long, Sun Yuan-zheng.(2008)Observation on therapeutic effect of acupuncture for treatment of [essential tremor](http://dict.cnki.net/dict_result.aspx?searchword=特发性震颤&tjType=sentence&style=&t=essential+tremor).Journal of Clinical Acupucnture and Moxibustion, 24:22-23.

[869] 陈燕坤. (2007)针刺治疗膝关节骨性关节炎304例疗效对照. 针灸临床杂志, 2007, 23:19-20.

[869] Chen Yan-kun. (2007)Effective comparison of 304 cases of knee osteoarthritis treated by acupuncture , JOURNAL OF CLINICAL ACUPUNCTURE AND MOXIBUSTION, 23:19-20.

[870] 贺军. (2002)针刺治疗腰椎间盘突出症临床观察. 针灸临床杂志, 2002, 18:11-12.

[870] . He jun (2002).Clinical Observation of Acupuncture Treating Prolapse of Lumbar Intervertebral Disc JOURNAL OF CLINICAL ACUPUNCTURE AND MOXIBUSTION, 18:11-12.

[871] 徐凤鸣, 王奇, 刘晓磊. (2009)针刺治疗抑郁症的临床观察. 针灸临床杂志, 2009, 25:27-28.

[871] Xu Feng-ming.Wang Qi Liu Xiao-lei (2009)Clinical Observation on Treating Dysthymia Disorders by Acupuncture. JOURNAL OF CLINICAL ACUPUNCTURE AND MOXIBUSTION, 25:27-28.

[872] 付蕾. (2010)针刺治疗原发性痛经50例. 针灸临床杂志, 2010, 26:16-17.

[872] Fu Lei. (2010)The Clinical Observation of the Acupuncture Treatment for Primary Dysmenorrheal 50 Cases.JOURNAL OF CLINICAL ACUPUNCTURE AND MOXIBUSTION, 26:16-17.

[873] 曹春梅. (2005)针刀、药、超短波结合治疗肱骨外上髁炎疗效观察. 针灸临床杂志, 2005, 21:29-30.

[873] Cao Chun-mei. (2005)Observations on the effect of the small needle knife and medicine and ultrashort therapy on inflammation with the humerus epicondylus lateralis , Cases. JOURNAL OF CLINICAL ACUPUNCTURE AND MOXIBUSTION, 21:29-30.

[874] 桂清民. (2010) 针刀闭合松解术配合手法治疗神经根型颈椎病临床研究. 针灸临床杂志, 2010, 26:29-31

[874] Gui Qing-min. (2010) Clinical Study of the Needle Scalpel Close Loosening Therapy Combined with Manual Therapy in the Treatment of the Radicular Cervical Spondylosis.JOURNAL OF CLINICAL ACUPUNCTURE AND MOXIBUSTION, 26:29-31

[875] 於芸. (2010)针刀配合手法治疗颈性眩晕的临床观察. 针灸临床杂志, 2010, 26:50-51.

[875] Yu Yun. (2010).Clinical observations on Cervical certigo Treated by Acupunture therapy. JOURNAL OF CLINICAL ACUPUNCTURE AND MOXIBUSTION, 26:50-51.

[876] 陈美仁, 李强. (2009)针刀治疗面肌痉挛20例. 针灸临床杂志, 2009, 25:34-35.

[876] Chen Mei-ren.Li Qiang. (2009) Clinical observation on 20 cases of hemifacial spasm treated with Acupuncture therapy. JOURNAL OF CLINICAL ACUPUNCTURE AND MOXIBUSTION , 2009, 25:34-35.

[877] 柳爱红. (2009)针罐并用治疗荨麻疹临床疗效观察. 针灸临床杂志, 2009, 25:16.

[877] Liu Ai-hong. (2009)Clinical Observation on Urticaria Treated by Acupuncture and Cupping Therapy. JOURNAL OF CLINICAL ACUPUNCTURE AND MOXIBUSTION, 2009, 25:16.

[878] 谢文霞, 潘进钱, 叶天申. (2004) 针灸对脑外伤患者血清中SOD、MDA、LPO水平变化的研究. 针灸临床杂志, 2004, 20:14-16

[878] Xie Wen-xia Qian Jin-qian Ye Tian-kun. (2004) The study of investigation acupuncture and moxibustion in effect the blood serum level of SOD, MDA, LPO in acute cerebral trauma patiens JOURNAL OF CLINICAL ACUPUNCTURE AND MOXIBUSTION, 2004, 20:14-16

[879] 徐佳. (2005) 针灸耳压治疗肥胖症215例临床观察. 针灸临床杂志, 2005, 04, 13-14+11

[879] Xu Jia. (2005) JOURNAL OF CLINICAL ACUPUNCTURE AND MOXIBUSTION, 04, 13-14+11 Clinical observation on 215 cases of obesity treated with acupuncture and Auricular point sticking, 2005, 04, 13-14+11

[880] 潘良德. (2004)针灸加短波治疗周围性面瘫38例临床分析. 针灸临床杂志, 2004, 20:26-27.

[880] Pan Liang-de. (2004)Clinical analysis of 38 patients with peripheral facial paralysis treated with acupuncture and short wave JOURNAL OF CLINICAL ACUPUNCTURE AND MOXIBUSTION , 2004, 20:26-27.

[881] 黄龙模. (2008)针灸加穴位注射治疗中风后遗症50例. 针灸临床杂志, 2008, 24:51-52

[881] Huang Long-mo. (2008)Acupuncture treatment combined with acupoints injecting on the sequelae of stroke. JOURNAL OF CLINICAL ACUPUNCTURE AND MOXIBUSTION., 2008, 24:51-52

[882] 徐佳. (2005)针灸加穴注治疗慢性盆腔炎疗效观察. 针灸临床杂志, 2008, 21:16-17.

[882] Xu Jia. (2005)Observation on the therapeutic effect of acupuncture and point- injection on chronic pelvic inflammatory disease .JOURNAL OF CLINICAL ACUPUNCTURE AND MOXIBUSTION, 2008, 21:16-17.

[883] 姜晓岩, 任凯, 赵希武. (2008)针灸结合西酞普兰治疗抑郁症34例. 针灸临床杂志, 2008, 24:18-19.

[883] Jiang Xiao-yan.Ren Kai.Zhao Xi-wu (2008)Clinical research on 68 treatment of depression with citalopram and electric acupuncture .JOURNAL OF CLINICAL ACUPUNCTURE AND MOXIBUSTION, 2008, 24:18-19.

[884] 王宗信. (2007)针灸结合中药川芎茶调散治疗偏头痛45例临床体会. 针灸临床杂志, 2007, 24:22-23

[884] Wang Zong-xin. (2007)Clinical experience on 45 treatment of migraine treated with acupuncture combined with TCM Chuan Xiong Cha. JOURNAL OF CLINICAL ACUPUNCTURE AND MOXIBUSTION, 2007, 24:22-23

[885] 郭崇秋. (2008)针灸埋线疗法治疗第三腰椎横突综合征110例. 针灸临床杂志, 2008, 24:13-14

[885] Guo Chong-qiu. (2008)Clinical research on 110 treatment of Third lumbar transverse process syndrome treated with Acupuncture embedding therapy . JOURNAL OF CLINICAL ACUPUNCTURE AND MOXIBUSTION, 2008, 24:13-14.

[886] 王启芳, 李新民. (2004)针灸配合刮痧治疗肩周炎104例疗效观察. 针灸临床杂志, 2004, 20:7-8

[886] .Wang Qi-fang Li Xin-min (2004)Effect observation of scapulohumeral periarthritis treated with acupuncture and cutaneous scraping. JOURNAL OF CLINICAL ACUPUNCTURE AND MOXIBUSTION, 2004, 20:7-8

[887] 吴立雄, 王玲, 陶加平, 汪栋材. (2008)针灸任脉穴治疗糖尿病胃轻瘫35例临床观察. 针灸临床杂志, 2008, 24:40-42

[887] Wu Li-xiong Wang Lin Tao Jia-ping Wang Dong-cai, .(2008)The effect of the treatment of DGP by needling acupoints in the Ren meridian. JOURNAL OF CLINICAL ACUPUNCTURE AND MOXIBUSTION., 2008, 24:40-42

[888]王学红. (2008)针灸推拿治疗肩周炎疗效比较. 针灸临床杂志, 2008, 24:27-28

[888] Wang Xue-hong. (2008)Comparison of effects on Frozen treated by Acupuncture and Massage, URNAL OF CLINICAL ACUPUNCTURE AND MOXIBUSTION, 2008, 24:27-28

[889] 罗树华. (2007)针灸治疗单纯性肥胖60例临床观察. 针灸临床杂志, 2007, 23:17-18.

[889] Luo Shu-hua . (2007) Clinical observation of 60 cases of simplicial adiposity treated with acupuncture.JOURNAL OF CLINICAL ACUPUNCTURE AND MOXIBUSTION , 2007, 23:17-18.

[890] 王孝平, 刘永敏, 郝颖. (2006)针灸治疗放化疗后顽固性呃逆35例. 针灸临床杂志, 2临床杂志20:13-14.

[890] Wang Xiao-ping, Liu Yong-mmin, Hao Ying. (2006) Clinical research on 35 treatment of Intractable hiccups after chemotherapy treated with Acupuncture, JOURNAL OF CLINICAL ACUPUNCTURE AND MOXIBUSTION, 20:13-14.

[891] 曾晓智, 黎仲, 彭庆, 麦少英. (2004)针灸治疗功能性子宫出血126例的随机对照观察. 针灸临床杂志20:13-14.

[891]Zeng Xiao-zhi. Li Zhong. Peng Qin. Mai Shao-ying. (2004) Acupuncture treatment of dysfunctional uterine bleeding observed 126 cases of randomized controlled. JOURNAL OF CLINICAL ACUPUNCTURE AND MOXIBUSTION 20:13-14.

[892] 高淑红. (2004)针灸治疗类风湿性关节炎疗效观察. 针灸临床杂志, 2004, 20:8-9.

[892] Gao Shu-hong. (2004)The clinical effect observation of rheumatoid arthritis treated with acupuncutre and moxibustion . JOURNAL OF CLINICAL ACUPUNCTURE AND MOXIBUSTION , 2004, 20:8-9.

[893]钟宾谟. (2005) 针灸治疗面神经炎的临床观察. 针灸临床杂志, 2005, 21:23-24.

[893] Zhong Bin-mo. (2005) Clinical observation of Facial paralysis treated by Acupuncture, . JOURNAL OF CLINICAL ACUPUNCTURE AND MOXIBUSTION , 2005, 21:23-24.

[894] 李显辉, 时海鹰, 杨晓宁, 张齐昌, 李敏, 等. (1998)针灸治疗糖尿病周围神经病变的临床观察. 针灸临床杂志, 1998, 14:16-17.

[894] Li Xian-hui, Shi Hai-ying, Yang Xiao-ning, Zhang Qi-chang Li Min, et al. (1998)Clinical observation of Diabetic peripheral neuropathy treated by Acupuncture, JOURNAL OF CLINICAL ACUPUNCTURE AND MOXIBUSTION , 1998, 14:16-17.

[895] 于金栋, 赵援非. (2007)针灸治疗药物依赖性头痛疗效观察. 针灸临床杂志, 2007, 23:27

[895] Yu Jin-dong Zhao Yuan-fei . (2007)Observations on treating the headache of drug - dependence by acup - mox ther , 2007JOURNAL OF CLINICAL ACUPUNCTURE AND MOXIBUSTION , 2007, 23:27

[896] 宁晓军. (2000)针挑治疗慢性萎缩性胃炎的临床观察. 针灸临床杂志, 2000, 16:35-37.

[896] Ning Xiao-jun. (2000) Clinical observation of Chronic atrophic gastritis treated by Acupuncture, JOURNAL OF CLINICAL ACUPUNCTURE AND MOXIBUSTION , 2000, 16:35-37.

[897] 李彦梅, 杨金山, 李晓光. (2002)针推并用治疗椎动脉型颈椎病的临床观察. 针灸临床杂志, 2002, 18:4-5

[897] Li Yan-mei Yang Jin-shan Li Xiao-guang (2002)Clinical observation of acupuncture and massage treating vertebral cervical spondylopathy . JOURNAL OF CLINICAL ACUPUNCTURE AND MOXIBUSTION , 2002, 18:4-5

[898] 陈传江. (2006)针推结合治疗腰椎间盘突出症的临床观察. 针灸临床杂志, 2006, 22:26-27.

[898] Chen Chuan-jiang. (2006)Clinical observation of acupuncture and massage treating vertebral cervical spondylopathy, JOURNAL OF CLINICAL ACUPUNCTURE AND MOXIBUSTION , 2006, 22:26-27.

[899]谭武, 何泽多, 陈志斌, 等. (2008)针推同步疗法为主治疗肩周炎疗效观察. 针灸临床杂志, 2008, 24:20-21.

[899] Tan Wu , HeZe-duo, et al. (2008)The clinical effect observation of Acupuncture therapy-based synchronization treating Frozen , JOURNAL OF CLINICAL ACUPUNCTURE AND MOXIBUSTION, 2008, 24:20-21.

[900] 黄晶. (2003)针药并用治疗急性缺血性脑血管病的临床观察. 针灸临床杂志, 2003, 19:14.

[900] Huang Jing. (2003)Clinical observation of acute ischemic cerebrovascular disease treated by acupuncture and drug. JOURNAL OF CLINICAL ACUPUNCTURE AND MOXIBUSTION , 2003, 19:14.

[901] 孙河, 樊晓瑞. (2010) 针药并用治疗视网膜色素变性临床研究. 针灸临床杂志, 2010, 26:20-22.

[901] Sun He, Pan Xiao-rui. (2010) , Clinical Study on Optic Atrophy Caused by Retinitis Pigmentosa Treated by Acupunture Combined with Chinese Medicine. JOURNAL OF CLINICAL ACUPUNCTURE AND MOXIBUSTION , 2010, 6:20-22.

[902] 肖海涛, 李景义. (2006)针药并用治疗小儿遗尿症40例. 针灸临床杂志, 2006, 22:20-21

[902] Xiao Hai-tao, Li Jing-yi. (2006)Clinical research on 40 treatment of Small two enuresis treated by Acupunture Combined with Chinese Medicine, JOURNAL OF CLINICAL ACUPUNCTURE AND MOXIBUSTION , 2006, 22:20-21

[903] 蔡国锋, 张殿全, 李旗, 孙波, 庄哲, 等. (2010)针药并用治疗原发性三叉神经痛47例. 针灸临床杂志, 2010, 26:6-10.

[903] Cai Guofeng, Zhang Dian-quan, Li Qi, Sun Bo, Zhunag Zhe, et al. (2010)Acupuncture Combined and Chinese Medicine to Treat 47 Cases of Primary Trigeminal Neuralgia. JOURNAL OF CLINICAL ACUPUNCTURE AND MOXIBUSTION , 2010, 26:6-10.

[904] 李景义, 李桂芬, 魏秀兰. (2004)针药并用治面神经炎120例对照观察. 针灸临床杂志, 2004, :18-19.

[904] Li Jing-yi, Li Gui-fang , Wei Xiu-lan. (2004) 2Clinical controlled observation of facial inflammation treated with acupuncture and medicine:120 cases reported, JOURNAL OF CLINICAL ACUPUNCTURE AND MOXIBUSTION , 2004, :18-19.

[905] 徐桂冬, 童延华, 黄富强, 童利民, 许云帆. (2006)针药结合对冠心病心绞痛患者生存质量影响. 针灸临床杂志, 2006, 22:16-17.

[905] Xu Gui-dong , Dong Yan-hua , Huang Fu-qiang , Dong Li-min , Xu Yun-fan. (2006)The impact on quality of Life in Patients with angina pectoris treated by Acupunture Combined with Chinese Medicine JOURNAL OF CLINICAL ACUPUNCTURE AND MOXIBUSTION , 2006, 22:16-17.

[906]徐维, 陈麟, 张冬梅, 杨广印. (2007) 针药结合治疗带状疱疹疗效观察. 针灸临床杂志, 2007, 23:18-19

[906] Xu Wei, Chen Lin, Zhang Dong-mei, Yang Guang-yin. (2007) The clinical effect observation of Shingles treated by Acupunture Combined with Chinese Medicine , JOURNAL OF CLINICAL ACUPUNCTURE AND MOXIBUSTION , 2007, 23:18-19

[907] 杨清杰, 杨改丽. (2008)针药结合治疗胆石症的临床观察. 针灸临床杂志, 2008, 24:23-24.

[907] Yang Qing-jie, Yang Gai-li. (2008) Clinical observation of cholelithiasis treated by needling companiea with medicine, JOURNAL OF CLINICAL ACUPUNCTURE AND MOXIBUSTION , 2008, 24:23-24.

[908] 赵晖. (2007)针药结合治疗急性痛风性关节炎60例. 针灸临床杂志, 2007, 23:26-27.

[908] Zhao Hui. (2007)Clinical study on acute goute arthritis treated by acupuncture combined with Chinese medicine. JOURNAL OF CLINICAL ACUPUNCTURE AND MOXIBUSTION , 2007, 23:26-27.

[909] 赵立明. (2004)针药结合治疗甲状腺机能亢进性心脏病155例. 针灸临床杂志, 2004, 20:32-33.

[909] Zhao Li-ming. (2004) Acupuncture Combined and Chinese Medicine to Treat 155 Cases of Hyperthyroid heart disease.JOURNAL OF CLINICAL ACUPUNCTURE AND MOXIBUSTION , 2004, 20:32-33.

[910] 叶淑兰. (2007)针药结合治疗老年顽固性失眠58例. 针灸临床杂志, 2007, 23:16-17.

[910] Ye Shu-lan . (2007)Acupuncture Combined and Chinese Medicine to Treat 58 Cases of Elderly intractable insomnia, JOURNAL OF CLINICAL ACUPUNCTURE AND MOXIBUSTION , 2007, 23:16-17.

[911] 林源, 姚志芳, 陈旭军. (2007)针药结合治疗慢性浅表性胃炎. 针灸临床杂志, 2007, 23:23-24.

[911] Lin Yuan, Yao Zhi-jun, Chen Xu-jun. (2007)Combination of acupucnture and medicine in treating chronic superficial nephritis. JOURNAL OF CLINICAL ACUPUNCTURE AND MOXIBUSTION , 2007, 23:23-24.

[912]刘建萌. (2006)针药结合治疗面肌痉挛25例临床疗效观察. 针灸临床杂志, 2006, 22:13-14.

[912] Liu Jian-meng. (2006)The clinical effect observation of 25 treatment on hemifacial spasm by Acupunture Combined with Chinese Medicine JOURNAL OF CLINICAL ACUPUNCTURE AND MOXIBUSTION , 2006, 22:13-14.

[913] 曲齐生, 杨娜, 逄金彩. (2009)针药结合治疗面神经炎30例. 针灸临床杂志, 2009, 25:10-11.

[913] Qu Qi-sheng, Yang Na, Peng Jin-cai. (2009)Acupuncture Combined and Chinese Medicine to Treat 30 Cases of Facial paralysis, JOURNAL OF CLINICAL ACUPUNCTURE AND MOXIBUSTION , 2009, 25:10-11.

[914] 陶然, 孙远征. (2010)针药结合治疗脑梗死的临床观察. 针灸临床杂志, 2010, 26:27-28.

[914] Tao Ran, Sun Yuan-zheng. (2010)Observation on Effect of Cerebral Infarction treated by Acupuncture Combined with Naodesheng, JOURNAL OF CLINICAL ACUPUNCTURE AND MOXIBUSTION , 2010, 26:27-28.

[915] 孙远征, 范秋玉. (2010)针药结合治疗皮质下动脉硬化性白质脑病的临床观察. 针灸临床杂志, 2010, 26:18-20.

[915] Sun Yuan-zheng, Fan Qiu-yu. (2010) Acupuncture Treatment of Subcortical Arteriosclerotic Leukoencephalopathy Clinical Observation. JOURNAL OF CLINICAL ACUPUNCTURE AND MOXIBUSTION , 2010, 26:18-20.

[916] 左政, 姜云武, 李左芹. (2009)针药结合治疗强直性脊柱炎的临床疗效观察. 针灸临床杂志, 2009, 25:9-10.

[916] Zuo Zheng, Jiang Yun-wu, Li Zuo-qing. (2009)Clinical effect observation of Ankylosing Spondylitis treated by Acupunture Combined with Chinese Medicine JOURNAL OF CLINICAL ACUPUNCTURE AND MOXIBUSTION , 2009, 25:9-10.

[917] 郑杰. (2009)针药结合治疗糖尿病视网膜病变45例. 针灸临床杂志, 2009, 25:31-32.

[917] Zheng Jie . (2009)Acupuncture Combined and Chinese Medicine to Treat 45 Cases of Diabetic retinopathy, JOURNAL OF CLINICAL ACUPUNCTURE AND MOXIBUSTION, 2009, 25:31-32.

[918] 吴芙蓉. (2010)针药结合治疗血管性痴呆. 针灸临床杂志, 2010, 26:50-52.

[918] Wu Fu-rong (2010)Acupunture Combined with Chinese Medicine treat Vascular dementia.JOURNAL OF CLINICAL ACUPUNCTURE AND MOXIBUSTION, 2010, 26:50-52.

[919] 杨叁平, 胡建镇, 刘晓辉. (2005)针药结合治疗腰椎间盘突出症121例. 针灸临床杂志, 2005, 21:19-20.

[919] Yang Can-ping, Hu Jian-zhen, Liu Xiao-hui. (2005)Acupuncture Combined and Chinese Medicine to Treat 121 Cases of Lumbar disc herniation. JOURNAL OF CLINICAL ACUPUNCTURE AND MOXIBUSTION, 2005, 21:19-20.

[920] 王欣君, 王玲玲, 乔慧芬, 李建兵. (2008)针药结合治疗抑郁症睡眠障碍临床疗效观察. 针灸临床杂志, 2008, 24:1-2.

[920] Wang Xin-jun, Wang Ling-ling, Qiao Hui-fen, Li Jian-bing. (2008)Observing clinical efficacy and characteristics with acupuncture and medicine in treating depression sleep disorders. JOURNAL OF CLINICAL ACUPUNCTURE AND MOXIBUSTION, 2008, 24:1-2.

[921] 韩志君, 李蕾, 赵惠, 等. (2007)针药结合治疗原发性三叉神经痛30例临床观察. 针灸临床杂志, 2007, 23:17-18.

[921] Han Zhi-jun, Li Lei, Zhao Hui, et al. (2007)Clinical observation on 30 cases of primary trigeminal neuralgia treated by combination of acupuncture and medication.JOURNAL OF CLINICAL ACUPUNCTURE AND MOXIBUSTION, 2007, 23:17-18.

[922] 祝丰奎, 赵军, 丁勇. (2010)针药结合治疗中风后抑郁的临床疗效观察. 针灸临床杂志, 2010, 26:10-11

[922] Zhu Jun-kui, Zhao Jun, Ding Yong. (2010)Clinical observation on 30 cases of primary trigeminal neuralgia treated by combination of acupuncture and medication.JOURNAL OF CLINICAL ACUPUNCTURE AND MOXIBUSTION, 2010, 26:10-11

[923] 路学荣, 胡雪艳, 曲静. (2006)针药结合治疗椎-基底动脉供血不足的临床观察. 针灸临床杂志, 2006, 22:25-26.

[923] Lu Xue-rong, Hu Yan-jing, Qu Jing. (2006) Clinical observation on treatment of vertebro- basilar artery insufficinecy with acupuncture and Chinese medicine.JOURNAL OF CLINICAL ACUPUNCTURE AND MOXIBUSTION, 2006, 22:25-26.

[924] 张瑞杰, 周亚杰. (2007)针药治疗糖尿病50例疗效观察. 针灸临床杂志, 2007, 23:34-35.

[924] Zhang Rui-jie, Zhou Ya-jie. (2007)Clinical effect observation on of 50 treatment of Diabetes treated by Acupunture Combined with Chinese Medicine JOURNAL OF CLINICAL ACUPUNCTURE AND MOXIBUSTION, 2007, 23:34-35.

[925] 叶清景. (2006)针熨结合治疗神经根型颈椎病临床观察. 针灸临床杂志, 2006, 22:14-15.

[925] Ye Qing-jing. (2006) Clinical effect observation of Nerve root type cervical spondylosis treated by Acupunture Combined with Chinese Medicine.JOURNAL OF CLINICAL ACUPUNCTURE AND MOXIBUSTION, 2006, 22:14-15.

[926] 晁建国. (2005)中医联合疗法治疗增生性膝关节炎96例. 针灸临床杂志, 2005, 21:23-24.

[926] Chao Jian-guo. (2005) United TCM Methods Treating Hyperplastic Knee Arthritis.JOURNAL OF CLINICAL ACUPUNCTURE AND MOXIBUSTION, 2005, 21:23-24.

[927] 唐晓军, 刘波. (2005)滋阴补肾、宁心安神法针刺治疗2型糖尿病心脏植物神经病变40例. 针灸临床杂志, 2005, 21:29-30.

[927] Rang Xiao-jun, Liu Bo. (2005)Clinical observation on cardiac autonomic neuropathy in diabetes 2 with nourish kidney- yin and tranquilize mind acupuncture therapy.JOURNAL OF CLINICAL ACUPUNCTURE AND MOXIBUSTION, 2005, 21:29-30.

[928]余蕾, 张春婷, 曹雪梅. (2005)子宫穴温针灸为主治疗子宫肌瘤32例临床观察. 针灸临床杂志, 2005, 21:45.

[928] Yu Lei, Zhang Chun-ting, Cao Xue-mei. (2005).Clinical observation on 32 treatment of Uterine fibroids treated by Uterine warm acupuncture points-based .JOURNAL OF CLINICAL ACUPUNCTURE AND MOXIBUSTION, 2005, 21:45.

[929] 魏瑞仙, 葛宝和, 巴艳东. (2009)子母补泻法针刺治疗瘀血阻络型冠心病即刻效应的观察. 针灸临床杂志, 2009, 25:9-12.

[929] Wei Rui-xian, Ge Bao-he, Ba Yan-dong. (2009)Observation on Yuxuezuluo Coronary Artery Disease Immediate Effect Treated by Zimubuxiefa Acupuncture.JOURNAL OF CLINICAL ACUPUNCTURE AND MOXIBUSTION, 2009, 25:9-12.

[930] 牟宪慧. (2008)综合康复治疗脑卒中后偏瘫肩的疗效研究. 针灸临床杂志, 2008, 24:15-16.

[930] Mu Xian-hui . (2008)Efficacy studies in Comprehensive rehabilitation treating Post-stroke hemiplegic shoulder.JOURNAL OF CLINICAL ACUPUNCTURE AND MOXIBUSTION, 2008, 24:15-16.

[931] 彭慧渊, 何希俊, 杨璀健. (2010)足三里穴位注射弥可保针治疗贝尔麻痹36例. 针灸临床杂志, 2010, 26:52-54.

[931] Peng Hui-yuan, He Xi-jun, Yang Cui-jian. (2010)Effect Observing of Methycobal Zusanli Point Injection on Treating Bell Palsy 36 Cases.JOURNAL OF CLINICAL ACUPUNCTURE AND MOXIBUSTION, 2010, 26:52-54.

[932]周逸平, 王月兰, 方志斌. (1997)心经经脉与相应脏腑相关的研究针刺对冠心病患者血液5-HT、NT和TXB2、6Ket0-PGF1α含量的影响及相关的研究. 针灸临床杂志, 1997, 13:20-24.

[932]Zhou Yi-ping. Wang Yue-lan. Fang Zhi-bin. (1997) Reseach for patients with coronary heart blood 5-HT, and related research NT and TXB2, 6Ket0-PGF1α content Electroacupuncture, Wang Yuelan, Fang Zhibin (1997) Heart Meridian associated with the corresponding organs of acupuncture.JOURNAL OF CLINICAL ACUPUNCTURE AND MOXIBUSTION, 1997, 13:20-24.

[933] 刘敏, 苏同生, 毕宇峰. (1997)电针戒毒对海洛因成瘾的脱瘾效能观察. 针灸临床杂志, 1997, 13(4、5):40-42.

[933] Liu Min , Su Tong-sheng, Bi Yu-feng. (1997)EA drug withdrawal efficacy observation of heroin addiction, JOURNAL OF CLINICAL ACUPUNCTURE AND MOXIBUSTION, 1997, 13(4、5):40-42.

[934]解玉庆, 赵泉林, 王景波, [谢晓彬](http://search.cnki.com.cn/Search.aspx?q=author:谢晓彬), [秦颖](http://search.cnki.com.cn/Search.aspx?q=author:秦颖), 等. (1997)声电鍉针治疗对甲亢病人血压影响的观察. 针灸临床杂志, 1997, 13(4、5):36-37.

Xie Yi-qin. Zhao Quan-lin. Wang Jin-bo. Xie Xiao-bin. Qin-yin, et al.(1997)Acoustic-electric mention acupuncture affects blood pressure in patients with hyperthyroidism on the observation.JOURNAL OF CLINICAL ACUPUNCTURE AND MOXIBUSTION, 1997, 13(4、5):36-37

[935] 李春梅, 吴琛涛, 谷玉亭. (1997)针灸与推拿结合治疗腰椎间盘突出症63例临床观察. 针灸临床杂志, 1997, 13(4、5):30.

[935] Li Chun-mei, Wu Shen-tao, Gu Yu-ting . (1997) combination of acupuncture and Massage. JOURNAL OF CLINICAL ACUPUNCTURE AND MOXIBUSTION, 1997, 13(4、5):30.

[936] 钟奇, 张缙. (1997)通经接气针刺手法治疗急性脑梗塞的临床观察. 针灸临床杂志, 1997, 13:23-25.

[936] Zhong Qi, Zhang Jin. (1997)Clinical observation on 63 cases of Lumbar disc protrusion treated bynical observation on treatment of acute cerebral infarction treated by passing through coherent acupuncture techniques , JOURNAL OF CLINICAL ACUPUNCTURE AND MOXIBUSTION, 1997, 13:23-25.

[937] 冯湘, 王亚莉. (1997)针刺预防人工流产综合征的临床观察. 针灸临床杂志, 1997, 13:28.

[937] Feng Xiang, Wang Ya-li. (1997)Clinical observation on defense of Abortion syndrome treated by Acupuncture.JOURNAL OF CLINICAL ACUPUNCTURE AND MOXIBUSTION, 1997, 3:28.

[938] 殷克敬, 杜元灏, 王端辉. (1997)针刺治疗中风急性期机理研究. 针灸临床杂志, 1997, 13:16-19.

[938] Yin Ke-jing, Du Yuan-ying, Wang Duan-hui. (1997)Mechanism of acupuncture treatment of acute stroke. JOURNAL OF CLINICAL ACUPUNCTURE AND MOXIBUSTION, 1997, 13:16-19.

[939] 张平, 金雯. (1997)腰椎退行性骨关节病针灸证治体会. 针灸临床杂志, 1997, 13:43

[939] Zhang Ping, Jin Wen. (1997) Acupuncture Treatment Experience in Lumbar degenerative joint disease . JOURNAL OF CLINICAL ACUPUNCTURE AND MOXIBUSTION, 1997, 13:43

[940] 王如杰, 刘磊. (1999)补阴蹻泻阳蹻治疗不寐40例观察. 针灸临床杂志, 1999, 15:22-24.

[940] Wang Ru-jie, Liu Lei. (1999)Clinical observation on treatment of Insomnia treated by Qiao Qiao diarrhea yin yang. JOURNAL OF CLINICAL ACUPUNCTURE AND MOXIBUSTION, 1999, 5:22-24.

[941] 杨颖, 王照浩. (1999)头针治疗闭合性颅脑外伤的临床观察. 针灸临床杂志, 1999, 15:29-31.

[941] Yang Ying, Wang Zhao-hao. (1999)Clinical observation on treatment of Closed head injury treated by Closed scalp. JOURNAL OF CLINICAL ACUPUNCTURE AND MOXIBUSTION, 1999, 15:29-31.

[942] 童利民, 瞿群威, 张道敬, [王大军](http://search.cnki.com.cn/Search.aspx?q=author:王大军), [李江明](http://search.cnki.com.cn/Search.aspx?q=author:李江明), 等. (1999)电针加颈动脉注药对脑梗塞患者甲皱微循环的影响及疗效观察. 1999, 15:48-50.

[942] Tong Li-min, Qu Qun-wei, Zhang Dao-jing. Wang Da-jun, Li Jiang-ming, et al. (1999)The impact and efficacy observation of electroacupuncture plus carotid injection for patients with cerebral infarction nail fold microcirculation. 1999, 15:48-50.

[943] 李淑荣, 邹艳红, 桑鹏, [吕昊哲](http://search.cnki.com.cn/Search.aspx?q=author:吕昊哲), [杨敏](http://search.cnki.com.cn/Search.aspx?q=author:杨敏), 等. (1999)针刺配合运动疗法对56例中风偏瘫患者肢体运动功能障碍的疗效研究. 1999, 15:14-15.

[943] Li Shu-rong, Zou Yan-hong, Sang Peng, Lv Hao-zhe, Yang Min, et al. (1999)Research on the efficacy of acupuncture combined with exercise therapy for 56 cases of stroke patients with hemiplegia limb movement dysfunction.JOURNAL OF CLINICAL ACUPUNCTURE AND MOXIBUSTION, 1999, 15:14-15.

[944] 刘静. (2000)颞三针法加体针治疗脑梗塞恢复期60例临床观察. 针灸临床杂志, 2000, 16:29-30.

[944] Liu Ji. (2000)Clinical observation on 63 cases of Infarction convalescence after treating by Temporal three acupuncture and body acupuncture. JOURNAL OF CLINICAL ACUPUNCTURE AND MOXIBUSTION, 2000, 6:29-30.

[945] 常华, 陈延州, 高清云. (2000)针刺麻醉在老年人体外震波碎石中的应用. 针灸临床杂志, 2000, 16:6-8

[945] Chang Hua, Chen Yan-zhou, Gao Qing-yun. (2000)The use of Acupuncture anesthesia in extracorporeal shock wave lithotripsy in the elderly. JOURNAL OF CLINICAL ACUPUNCTURE AND MOXIBUSTION, 2000, 16:6-8

[946] 蔡德锋. (2000)温针为主综合治疗膝关节积水150例. 针灸临床杂志, 2000, 16:6-8.

[946] Cai De-feng. (2000) 150 cases of Acupuncture treatment of knee warm water comprehensive.JOURNAL OF CLINICAL ACUPUNCTURE AND MOXIBUSTION , 2000, 16:6-8.

[947] 王如杰. (2001)针刺治疗血管性痴呆42例临床分析. 针灸临床杂志, 2001, 17:8.

[947] Wang Ru-jie. (2001)Clinical analysis of 42 cases in vascular dementia treated by acupuncture. JOURNAL OF CLINICAL ACUPUNCTURE AND MOXIBUSTION, 2001, 17:8.

[948] 张庆力. (2001)针刺推拿治疗腰椎间盘突出症. 针灸临床杂志, 2001, 17:5.

[948] Zhang Qing-li. (2001)Acupuncture and massage treatment of lumbar disc herniation.JOURNAL OF CLINICAL ACUPUNCTURE AND MOXIBUSTION , 2001, 17:5.

[949] 刘凤菠, 付美香. (2001) G6805+TDP治疗乳腺增生病128例的临床观察. 针灸临床杂志, 2001, 17:34.

[949] Liu Feng-bo, Fu Mei-xiang. (2001). Clinical research on 128 treatment of cyclomastopathy.JOURNAL OF CLINICAL ACUPUNCTURE AND MOXIBUSTION, 2001, 17:34.

[950] 唐强, 郭艳芹, 田晓彦, 倪金霞, 张慧敏, 等. (2001)头穴丛刺长留针法治疗急性脑梗死的临床观察. 针灸临床杂志, 2001, 17:57.

[950] Tang Qiang. Guo Yang-qin. Tian Xiao-yan. Ni Jin-xia. Zhang Hui-ming, et al.(2006) Clinical Observation of Acute Cerebral Infarction Treated with Cluster Needles on Scalp-point by Retaining Long Time. JOURNAL OF CLINICAL ACUPUNCTURE AND MOXIBUSTION, 2001 , 17:57.

[951] 谢来芬, 沈玉杰. (2001)风湿仙丹结合穴位埋线对类风湿性关节炎患者血浆中cAMP、cGMP含量的影响. 针灸临床杂志, 2001, 17:51-52.

[951]Xie Lai-fen. Shen Yu-jie.(2001) Rheumatoid elixir combines catgut embedding rheumatoid arthritis patients on plasma cAMP, cGMP content. JOURNAL OF CLINICAL ACUPUNCTURE AND MOXIBUSTION, 2001, 17:51-52.

[952] 林矛. (2002)穴注治疗原发性三叉神经痛103例疗效观察. 针灸临床杂志, 2002, 18:37.

[952]Lin Mao.(2002) Observation on Curative Effectiveness of 103 Patients with Primary Trigeminal Neuralgia Treated with Injection on Acupoint. JOURNAL OF CLINICAL ACUPUNCTURE AND MOXIBUSTION, 2002, 18:37.

[953] 田丰伟, 路瑜, 李宁. (2002) “管灸”疗法治疗周围性面瘫的临床疗效观察. 针灸临床杂志, 2002, 18:50-52.

[953]Tian Fen-wei. Lu Yu. Li Ling.(2002) Clinical Observation "pipe moxibustion" therapy for treatment of peripheral facial paralysis. JOURNAL OF CLINICAL ACUPUNCTURE AND MOXIBUSTION, 2002, 18:50-52.

[954] 余霜泉. (2002)齐刺与常规针法治疗颈椎病临床疗效对比观察. 针灸临床杂志, 2002, 18:35.

[954]Yu Shuang-quan.(2002) Clinical observation of Needling acupuncture with conventional treatment of cervical spondylosis . JOURNAL OF CLINICAL ACUPUNCTURE AND MOXIBUSTION, 2002, 18:35.

[955] 袁淑华, 徐洁, 张岩. (2002) 电针配合汤药治疗血管性痴呆的疗效观察. 针灸临床杂志, 2002, 18:40-41.

[955]Yuan Shu-hua. Xu Jie. Zhang Yan.(2002)Observation of curative effectiveness of vascular dementia treated with electronic acupuncture and herb broth. JOURNAL OF CLINICAL ACUPUNCTURE AND MOXIBUSTION, 2002, 18:40-41.

[956] 李华明. (2002)腰椎间盘突出症150例临床观察. 针灸临床杂志, 2002, 18:4-5.

[956] Li Hua-ming.(2002).Clinical observation of lumbar disc herniation 150 cases. JOURNAL OF CLINICAL ACUPUNCTURE AND MOXIBUSTION, 2002, 18:4-5.

[957] 黄烈弥. (2002)电针拔罐与低中频脉冲电疗法治疗不同类型颈椎病的疗效观察. 针灸临床杂志, 2002, 18:34-35.

[957]Huang Lie-mi.(2002) Efficacy of acupuncture and cupping therapy for low-IF pulse of different types of cervical spondylosis. JOURNAL OF CLINICAL ACUPUNCTURE AND MOXIBUSTION, 2002, 18:34-35.

[958] 刘继洪, 施兰, 潘丽玲. (2002)多功能针耳穴治疗寻常性痤疮的临床观察. 针灸临床杂志, 2002, 18:7-8.

[958]Liu Shi-hong. Shi Lan. Pan Li-ling(2002) Clinical observation of acne vulgaris versatile ear acupuncture treatment. JOURNAL OF CLINICAL ACUPUNCTURE AND MOXIBUSTION, 2002, 18:7-8.

[959] 王伟华, 金泽, 孙远证, [李书霖](http://social.wanfangdata.com.cn/Locate.ashx?ArticleId=zjlczz200301027&Name=李书霖). (2003)电鍉针治疗慢性前列腺炎对尿代动力学的临床研究. 针灸临床杂志, 2003, 19:31-32.

[959] Wang Wei-hua. Jin Ze. Sun Yuan-zhneg. Li Shu-lin.(2003)Clinical study of urine pharmacokinetics of electric 鍉 treatment of chronic prostatitis.JOURNAL OF CLINICAL ACUPUNCTURE AND MOXIBUSTION, 2002, 19:31-32.

[960] 陈小凯, 吴虹. (2003)针刺晕听区治疗颈性眩晕80例疗效观察. 针灸临床杂志, 2003, 19:12-13.

[960]Chen Xiao-kai. Wu Hong.(2003) 80 cases of cervical vertigo efficacy of acupuncture treatment dizzy listening area. JOURNAL OF CLINICAL ACUPUNCTURE AND MOXIBUSTION, 2003, 19:12-13.

[961] 李圣平, 王小梅. (2003)全息胚电针氧疗治疗面神经炎临床观察. 针灸临床杂志, 2003, 19:30.

[961] Li Shen -ping. Wang Xiao-mei.(2003)ECIWO EA oxygen therapy facial neuritis clinical observation. JOURNAL OF CLINICAL ACUPUNCTURE AND MOXIBUSTION, 2003, 19:30.

[962] 吴昊. (2003)体针结合回旋灸治疗带状疱疹临床观察. 针灸临床杂志, 2003, 19:55-56.

[962] Wu Hao.(2003) Roundabout body acupuncture moxibustion treatment of herpes zoster. JOURNAL OF CLINICAL ACUPUNCTURE AND MOXIBUSTION, 2003, 19:55-56.

[963] 金孟梓, 陈加强, 陈先威, 陈德沛. (2003)小针刀为主治疗第三腰椎横突综合症临床研究. 针灸临床杂志, 2003, 19: 21-22.

[963] Jin Meng-xin. Chen Jia-qiang. Chen Xian-wei. Chen De-pei.(2003) Small knife dominated the third lumbar transverse process syndrome therapy clinical research. JOURNAL OF CLINICAL ACUPUNCTURE AND MOXIBUSTION, 2003, 19: 21-22.

[964] 吴亿中. (2003)齐刺、温针灸治疗肱骨外上髁炎74例临床观察. 针灸临床杂志, 2003, 19:33-34.

[964]Wu Yi-zhong.(2003)Clinical observation on the treatment of 74 cases of external humeral epicondylitis with triple needling plus warmed-needle acupuncture. JOURNAL OF CLINICAL ACUPUNCTURE AND MOXIBUSTION, 2003, 19:33-34.

[965] 崔新坤. (2003)头皮针加推拿治疗中风偏瘫56例疗效观察. 针灸临床杂志, 2003, 19:43-44.

[965]Cui Xin-kun.(2003) Scalp acupuncture and massage therapy efficacy 56 cases of stroke hemiplegia.JOURNAL OF CLINICAL ACUPUNCTURE AND MOXIBUSTION, 2003, 19:43-44.

[966] 江勇, 王敏华, 王敏, 华启海, 李震宇, 等. (2003)针刺推拿治疗紧张性头痛临床研究. 针灸临床杂志, 2003, 19:7-8.

[966]Jiang Yong. Wang Min-hua. Wagn Min. Hua Qi-hai. Li Zhen-yu.(2003) Clinical study on the treatment for tension headache by acupuncture and Chinese massage. JOURNAL OF CLINICAL ACUPUNCTURE AND MOXIBUSTION, 2003, 19:7-8.

[967] 宿中笑, 郑丽丽. (2003)针刺治疗黄褐斑的临床疗效观察. 针灸临床杂志, 2003, 19:9-10.

[967]Su Zhong-xiao. Zheng Li-li.(2003) Clinical observations on curative effect of cholasma by the acupuncture. JOURNAL OF CLINICAL ACUPUNCTURE AND MOXIBUSTION, 2003, 19:9-10

[968] 谢凯. (2003)电针配合牵引治疗腰椎间盘突出症临床观察. 针灸临床杂志, 2003, 19:29.

[968]Xie Kai (2003)Clinical observation on treating LDH by EA plus traction. JOURNAL OF CLINICAL ACUPUNCTURE AND MOXIBUSTION, 2003, 19:29.

[969] 金伟, 杨金山. (2003)颈项针配合牵引理疗治疗神经根型颈椎病的临床观察. 针灸临床杂志, 2003, 19:49-50.

[969]Jin Wei. Yang Jin-shan.(2003)Clinical Acupuncture and neck traction therapy treatment of nerve root type cervical spondylosis.JOURNAL OF CLINICAL ACUPUNCTURE AND MOXIBUSTION, 2003, 19:49-50.

[970] 潘文宇, 李艳慧, 宋金带. (2003)针刺加火针治疗椎动脉型颈椎病的疗效观察. 针灸临床杂志, 2003, 19:10-11.

[970]Pan Wen-yun. Li Yan-hui. Song Jin-dai(2003) Efficacy of acupuncture and acupuncture fire cervical vertebral artery disease. JOURNAL OF CLINICAL ACUPUNCTURE AND MOXIBUSTION, 2003, 19:10-11.

[971] 周一敏, 冷钰玲. (2003)电针治疗宫颈癌术后尿潴留40例. 针灸临床杂志, 2003, 19:23-24.

[971]Zhou Yi min. Leng Yu-ling.(2003)Acupuncture treatment of cervical cancer 40 cases of postoperative urinary retention..JOURNAL OF CLINICAL ACUPUNCTURE AND MOXIBUSTION, 2003, 19:23-24.

[972] 吴耀. (2003) “气至病所”针法治疗臀上皮神经损伤的疗效观察. 针灸临床杂志, 2003, 19:30-31.

[972]Wu Yao.(2003) Observation on therapeutic effect of contusion of superior clunial nerve treated with acupuncture of Qi extending affected parts method. JOURNAL OF CLINICAL ACUPUNCTURE AND MOXIBUSTION, 2003, 19:30-31.

[973] 周忠亮. (2003)少阳三针刺法治疗偏头痛42例观察. 针灸临床杂志, 2003, 19:33.

[973]Zhou Zhong-liang.(2003)Shaoyang three acupuncture treatment 42 patients with migraine.JOURNAL OF CLINICAL ACUPUNCTURE AND MOXIBUSTION, 2003, 19:33.

[974] 孙华. (2003)温针灸在术后胃瘫治疗中的作用. 针灸临床杂志, 2003, 19:38-40.

[974]Sun Hua.(2003) Action of needle warming through moxibustion in treatment of postoperative stomach palsy. JOURNAL OF CLINICAL ACUPUNCTURE AND MOXIBUSTION, 2003, 19:38-40.

[975] 徐斯伟, 张载义, 胡蔚琼. (2003)艾炷灸加针刺治疗顽固性周围性面瘫的临床观察. 针灸临床杂志, 2003, 19:40-41.

[975] Xu Si-wei. Zhang Zai-yi. Hu Wei-qiong.(2003) Moxa moxibustion acupuncture treatment of intractable clinical observation of peripheral facial paralysis. JOURNAL OF CLINICAL ACUPUNCTURE AND MOXIBUSTION, 2003, 19:40-41.

[976] 王玲. (2003)针灸缪刺法治疗面肌痉挛临床观察. 针灸临床杂志, 2003, 19:17-18.

[976]Wang Ling.(2003) Acupuncture Needling Therapy Clinical hemifacial spasm.JOURNAL OF CLINICAL ACUPUNCTURE AND MOXIBUSTION, 2003, 19:17-18.

[977] 冯淑兰, 何新芳. (2003)颞三针治疗无先兆型偏头痛临床疗效观察. 针灸临床杂志, 2003, 19:23-24.

[977]Feng Shu-fen. He Xin-fang.(2003)Clinical observation of migraine by the temploral three acupoints. JOURNAL OF CLINICAL ACUPUNCTURE AND MOXIBUSTION, 2003, 19:23-24.

[978] 严伟, 殷建权. (2003)体针头皮针结合治疗老年性震颤的疗效观察. 针灸临床杂志, 2003, 19:34.

[978] Yan Wei. Ying Jian-quan.(2003)Efficacy of scalp acupuncture combined with body acupuncture treatment of senile tremor.JOURNAL OF CLINICAL ACUPUNCTURE AND MOXIBUSTION, 2003, 19:34.

[979] 庞勇. (2003)益肾调督针法对脑梗塞血管活性物质的影响. 针灸临床杂志, 2003, 19:50-52.

[979]Pang Yong. (2003)Governor acupuncture affects kidney tune of cerebral vasoactive substances. JOURNAL OF CLINICAL ACUPUNCTURE AND MOXIBUSTION, 2003, 19:50-52.

[980] 娄玉方. (2003)针刺缺盆穴为主治疗神经根型颈椎病的疗效观察. 针灸临床杂志, 2003, 19:56.

[980]Lou Yu-fang.(2003) Quepen efficacy of acupuncture points for treatment of nerve root type cervical spondylosis. JOURNAL OF CLINICAL ACUPUNCTURE AND MOXIBUSTION, 2003, 19:56.

[981] 蔡曰新, 蔡勋. (2003)针刺加中药熏蒸治疗急性腰肌扭伤98例. 针灸临床杂志, 2003, 19:43.

[981] Cai Yue-xin. Cai Xun.(2003) Acupuncture and Chinese medicine fumigation treatment of acute lumbar sprain 98 cases.JOURNAL OF CLINICAL ACUPUNCTURE AND MOXIBUSTION, 2003 , 19:43.

[982] 刘传瑞, 邱泽法, 张洪清. (2003)电针与金针促通术治中风肢体运动功能障碍临床观察. 针灸临床杂志, 2003, 19:46-47.

[982]Liu Chuan-rui. Qiu Ze-fa. Zhang Hong-qing.(2003)EA and treatment of stroke patients lilies facilitation motor dysfunction clinical observation. JOURNAL OF CLINICAL ACUPUNCTURE AND MOXIBUSTION, 2003, 19:46-47.

[983] 陈小凯, 吴虹. (2003)巨刺治疗膝骨关节炎81例疗效观察. 针灸临床杂志, 2003, 19:53.

[983] Chen Xiao-kai. Wu Hong.(2003) Needling treatment of knee osteoarthritis 81 cases were observed. JOURNAL OF CLINICAL ACUPUNCTURE AND MOXIBUSTION, 2003 , 19:53.

[984] 谢感共, 谭琳蓥, 卢献群, 赵彩娇. (2003) 灵龟八法治疗原发性痛经临床研究. 针灸临床杂志, 2003, 19:59-60.

[984] Xie Gan-gong. Tan Lin-rong. Lu Xian-qun. Zhao Cai-qiao.(2003)Clinical study on primary dysmenorrheal treated with eight methods of intelligentturtle. JOURNAL OF CLINICAL ACUPUNCTURE AND MOXIBUSTION, 2003, 19:59-60.

[985] 于晓曦. (2003)电针治疗血管性痴呆46例临床观察. 针灸临床杂志, 2003, 19:27-28.

[985]Yu Xiao-xi.(2003)Clinical observation of vascular dementia treated with electrotherapy:46 cases reported. JOURNAL OF CLINICAL ACUPUNCTURE AND MOXIBUSTION, 2003, 19:27-28.

[986] 毕焕洲, 李延, 秦玉文, 李书霖, 徐行军. (2003)蟾蜂栓合电针治疗前列腺增生(BPH)的临床观察. 针灸临床杂志, 2003, 19:37.

[986] Bi Huan-zhou. Li Yan. Qin Yu-wen. Li Shu-wen.(2003)Bee toad tied together EA treatment of benign prostatic hyperplasia (BPH) clinical observation.. JOURNAL OF CLINICAL ACUPUNCTURE AND MOXIBUSTION, 2003, 19:37.

[987] 刘原龙, 林华日. (2003)灯芯灸联合胸腺肽治疗抗HBe阳性慢性乙型肝炎疗效观察. 针灸临床杂志, 2003, 19:39-40.

[987]Liu Yuan-long. Lin Hua-ri.(2003)Thymosin wick moxibustion therapy anti-HBe positive chronic hepatitis B Efficacy. JOURNAL OF CLINICAL ACUPUNCTURE AND MOXIBUSTION, 2003, 19:39-40.

[988] 张宏图. (2003)针刺治疗血管性头痛的临床研究. 针灸临床杂志, 2003, 19:8-9.

[988]Zhang Hong-tu.(2003) The clinical study on vascular headache treated with acupuncture. JOURNAL OF CLINICAL ACUPUNCTURE AND MOXIBUSTION, 2003, 19:8-9.

[989] 左晓峰. (2003)针刺配合推拿治疗椎动脉型颈椎病临床观察. 针灸临床杂志, 2003, 19:10-12.

[989] Zuo Xiao-feng.(2003)Clinical Study of Treatment of Cervical Spondylotic Vertebral Arteriopathy by Acupuncture plus Massotherapy. JOURNAL OF CLINICAL ACUPUNCTURE AND MOXIBUSTION, 2003, 19:10-12.

[990] 吴立红, 王小平. (2003)温针配合膝关节运动疗法治疗膝痹75例临床观察. 针灸临床杂志, 2003, 19:42-43.

[990]Wu Li-hong. Wang Xiao-ping.(2003)Clinical observation of treatment of 75 cases of paralysis knee knee warm Acupuncture and exercise therapy. JOURNAL OF CLINICAL ACUPUNCTURE AND MOXIBUSTION, 2003, 19:42-43.

[991] 金明月, 李政, 刘松雨, 马红鹤, 李锦瑞, 等. (2003)早期针刺康复治疗中风病425例疗效观察. 针灸临床杂志, 2003, 19:10-11.

[991] Jin Ming-yue. Li Zheng. Liu Song-yu. Ma Hong-he. Li Jin-rui, et al.(2003)Clinical effect observation of stroke treated with early acupuncture rehabilitation treatment:425 cases reported. JOURNAL OF CLINICAL ACUPUNCTURE AND MOXIBUSTION, 2003, 19:10-11.

[992] 沈克艰, 杨海鸥. (2003)腰骶部针刺治疗盆腔炎的疗效观察. 针灸临床杂志, 2003, 19:17-18.

[992]Shen Ke-jian. Yang Hai-ou.(2003) Clinical effect observation of pelvic inflammation treated with acupuncture at Lumbosacral point. JOURNAL OF CLINICAL ACUPUNCTURE AND MOXIBUSTION, 2003, 19:17-18.

[993] 薛爱国, 李秀霞, 黄雪梅. (2003) 针刺与药物治疗不伴先兆偏头痛的临床疗效对比研究. 针灸临床杂志, 2003, 19:23-24.

[993]Xue Ai-guo, Li Xiu-xia, Huang Xue-mei.(2003) Comparative study on clinical therapeutic effect of acupuncture and medicine on non-prmonitory migraine. JOURNAL OF CLINICAL ACUPUNCTURE AND MOXIBUSTION, 2003, 19:23-24.

[994] 李月梅. (2003) 电针治疗常年性变应性鼻炎的临床疗效观察. 针灸临床杂志, 2003, 19:16-17.

[994]LiYue-mei.(2003) Observation on clinical therapeutic effect of electroacupuncture on perennial allergic rhinitis JOURNAL OF CLINICAL ACUPUNCTURE AND MOXIBUSTION, 2003, 19:16-17.

## [995] 李常法, 赵藏朵, 刘彦华. (2003)三针四穴为主治疗面瘫50例疗效观察. 针灸临床杂志, 2003, 19:27-28.

[995] LiChang-fa, Zhao Cang-duo, Liu Yan-hua.(2003) Three-pin four points for treatment of facial paralysis Observation of 50 Cases. JOURNAL OF CLINICAL ACUPUNCTURE AND MOXIBUSTION, 2003, 19:27-28.

[996] 陈学农, 周清毅, 赵开祝, 杨庭辉. (2003)穴位埋线治疗陈旧性面瘫158例的临床研究. 针灸临床杂志, 2003, 19:35-36.

[996]Chen Xue-nong, Zhou Qing-yi, Zhao Kai-zhu, Yang Ting-hui.(2003) Clinicai investigate on catgut-embedding for treatment of dated facial neuritis in 158 cases. JOURNAL OF CLINICAL ACUPUNCTURE AND MOXIBUSTION, 2003, 19:35-36.

[997] 聂志华, 钟志伦. (2004)针刺结合血栓通治疗原发性坐骨神经痛46例. 针灸临床杂志, 2004, 20:19-20.

## [997] Nie Zhi-hua, Zhong Zhi-long.(2004) 46 cases with primordial sciatica treated with acupuncture and Xueshuantong. JOURNAL OF CLINICAL ACUPUNCTURE AND MOXIBUSTION, 2004 , 20:19-20.

## [998] 包大鹏, 孙远征. (2004)皮下埋针治疗寻常痤疮的临床研究. 针灸临床杂志, 2004, 20:31-32.

[998] Bao Da-peng, Sun Yuan-zheng(2004) Acne Vulgaris Clinical Research) subcutaneous buried acupuncture JOURNAL OF CLINICAL ACUPUNCTURE AND MOXIBUSTION, 2004, 20:31-32.

[999] 张晓军, 郑美华, 吴燕璟. (2004)针刺治疗功能性消化不良46例. 针灸临床杂志, 2004, 20:25-26.

[999] Zhang Xiao-jun, Zheng Mei-hua, WU Yan-jing.(2004) Acupuncture treatment of functional dyspepsia 46 cases JOURNAL OF CLINICAL ACUPUNCTURE AND MOXIBUSTION, 2004, 20:25-26.

[1000] 黄秋贤, 朴献玉, 王若梅. (2004)电针与高压氧结合治疗突发性耳聋48例临床观察. 针灸临床杂志, 2004, 20:36-37.

[1000] Huang Qiu-xian, Piao Xian-yu, Wang Ruo-mei.(2004)Sudden deafness 48 cases of clinical observation of electroacupuncture combined with hyperbaric oxygen therapy. JOURNAL OF CLINICAL ACUPUNCTURE AND MOXIBUSTION, 2004, 20:36-37.

[1001] 史永奋, 王丹华, 张海缨. (2004)运用《黄帝内经》傍针刺法治疗肩周炎疗效观察, 针灸临床杂志, 2004, 20:40-41.

[1001]Shi Yong-fen, Wang Dan-hua, Zhang Hai-ying.(2004) Therapeutic effect of proximal needling on periarthritis of shoulder in HuangdiNeijing. JOURNAL OF CLINICAL ACUPUNCTURE AND MOXIBUSTION, 2004, 20:40-41.

[1002] 潘田成. (2004)透刺结合丹栀逍遥散治疗失眠症临床对比研究. 针灸临床杂志, 2004, 20:43-44.

[1002]Pan Tian-cheng.(2004) Penetration combined DZXS Clinical comparative study of the treatment of insomnia . JOURNAL OF CLINICAL ACUPUNCTURE AND MOXIBUSTION, 2004, 20:43-44.

[1003] 程子刚. (2004)电针治疗中非地区肩、膝痛78例体会. 针灸临床杂志, 2004, 20:24.

[1003] Cheng Zi-gang.(2004)Electro-acupuncture Central Africa shoulder, knee 78 cases of Experience. JOURNAL OF CLINICAL ACUPUNCTURE AND MOXIBUSTION, 2004, 20:24.

[1004] 田丰玮, 苟春雁, 李宁. (2004)分期平衡针刺法治疗中风偏瘫的临床研究. 针灸临床杂志, 2004, 20:34-35.

[1004] Tian Feng-wei, Gou Chun-yan, Li Ning.(2004) Clinical research of stroke hemiplepia treated by the different stage balance acupuncture project.JOURNAL OF CLINICAL ACUPUNCTURE AND MOXIBUSTION, 2004, 20:34-35.

[1005] 赵树源. (2004)针刺疗法治疗中风偏瘫的临床研究. 针灸临床杂志, 2004, 20:10-11.

[1005] Zhao Shun-yuan.(2004) Clinical studies of acupuncture treatment for stroke hemiplegia.JOURNAL OF CLINICAL ACUPUNCTURE AND MOXIBUSTION, 2004.20:10-11.

[1006] 李宝栋, 白晶, 高维滨. (2004) 电针治疗隐性脊柱裂所致遗尿的临床研究. 针灸临床杂志, 2004, 20:49-50.

[1006]Li Bao-dong, Bai Jing, Gao Wei-bin.(2004) Clinical studies of acupuncture treatment induced enuresis spina bifida . JOURNAL OF CLINICAL ACUPUNCTURE AND MOXIBUSTION, 2004, 20:49-50

[1007] 翟铁军, 罗恩丽, 郭士杰. (2004) 针刺抗抑郁治疗对卒中后抑郁康复疗效的影响. 针灸临床杂志, 2004, 20:5-7.

[1007]Zhai Tie-jun, Luo En-li, Guo Shi-jie.(2004) Effect of acupuncture on the rehabilitation of depression after cerebral apoplexy.JOURNAL OF CLINICAL ACUPUNCTURE AND MOXIBUSTION, 2004, 20:5-7

[1008] 朱文增, 郭加利, 倪金霞, 鲍春玲, 东贵荣. (2004)头穴透刺结合康复治疗急性脑出血的临床研究. 针灸临床杂志 20:34-35.

[1008] Zhu Wen-zeng, Guo Jia-li, Ni Jia-xia, Bao Chun-ling, Dong Gui-rong.(2004) Clinical studies scalp penetration acupuncture combined with rehabilitation treatment of acute cerebral hemorrhage JOURNAL OF CLINICAL ACUPUNCTURE AND MOXIBUSTION 20:34-35

[1009] 杨焱, 陈红涛. (2004)头针治疗帕金森病30例的临床观察. 针灸临床杂志, 2004, 20:36.

[1009]Yang Yan, Chen Hong-tao.(2004)Clinical observation on the treatment of parkinson’s disease by scalp.JOURNAL OF CLINICAL ACUPUNCTURE AND MOXIBUSTION, 2004, 20:36

[1010] 旷秋和. (2004)隔蒜灸治疗神经性皮炎临床疗效观察. 针灸临床杂志, 2004, 20:41-42.

[1010]Kuang Qiu-he.(2004) Clinical efficacy neurodermatitis garlic separated moxibustion JOURNAL OF CLINICAL ACUPUNCTURE AND MOXIBUSTION, 2004, 20:41-42.

[1011] 谢感共, 郑政, 卢献群, 赵彩娇. (2004)灵龟八法治疗原发性高血压病即时效应观察. 针灸临床杂志, 2004, 20:36-38.

[1011]Xie Gan-gong, Zheng zheng, Lu Xian-qun, Zhao Cai-jiao.(2004) Lingguibafa observe the treatment of primary hypertension immediate effects of Clinical Acupuncture JOURNAL OF CLINICAL ACUPUNCTURE AND MOXIBUSTION, 2004, 20:36-38.

[1012] 王顺, 周振坤, 胡丙成, 蔡玉颖. (2004)面瘫胶囊Ⅱ号与针刺治疗面神经炎的对比研究. 针灸临床杂志, 2004, 20:8-9.

[1012]Wang Shun, Zhou Zhen-kun, Hu Bing-cheng, Cai Yu-ying.(2004) The controlled study of Facial nerve inflammation treated Miantan capsule Ⅱand acupuncture. JOURNAL OF CLINICAL ACUPUNCTURE AND MOXIBUSTION, 2004, 20:8-9.

[1013] 罗卫平, 黄红缨, 谭吉林, [赖莹莹](http://search.cnki.com.cn/Search.aspx?q=author:赖莹莹), [黄晓煌](http://search.cnki.com.cn/Search.aspx?q=author:黄晓煌), 等. (2004)针刺配合吞咽训练治疗脑卒中并发吞咽障碍30例疗效观察. 针灸临床杂志, 2004, 20:12-13.

[1013] Lu Wei-ping, Hong Hong-ying, Tan Xing-lin, Lai Ying-ying, Huang Xiao-huang, et al.(2004) Therapeutic effect of acupuncture combined with deglutition training on deglutition disorders complicated by apoplexy. JOURNAL OF CLINICAL ACUPUNCTURE AND MOXIBUSTION, 2004, 20:12-13.

[1014] 卢泽强. (2004)头皮针配合艾灸治疗椎动脉型颈椎病50例临床体会. 针灸临床杂志, 2004, 20:19-20.

[1014] Lu Ze-qiang.(2004) With moxibustion treatment of vertebral artery type of cervical spondylosis 50 cases of scalp acupuncture Clinical Experience JOURNAL OF CLINICAL ACUPUNCTURE AND MOXIBUSTION, 2004, 20:19-20.

[1015] 黄列英, 曾缓, 黄东博. (2004)针、药、远红外线照射治疗面瘫80例临床疗效分析. 针灸临床杂志, 2004, 20:27-28.

[1015] Huang Lie-ying, Zeng Huan, , Huang Dong-bo.(2004) Needles, medicine, far-infrared radiation treatment of facial paralysis clinical efficacy of 80 cases JOURNAL OF CLINICAL ACUPUNCTURE AND MOXIBUSTION, 2004, 20:27-28.

[1016] 李应昆, 刘军, 唐章全, [李晨](http://www.cqvip.com/Main/Search.aspx?w=李晨). (2004)智能型模拟光电药灸仪治疗痹症的随机对照研究. 针灸临床杂志, 2004, 20:39-41.

[1016] Li Ying-kun, Liu Jun, Tang Zhang-quan, Li Chen.(2004) Clinical effect of artificial instrument of simulating photoelectric moxibustion with Chinese medicines in treating Bi syndrome.JOURNAL OF CLINICAL ACUPUNCTURE AND MOXIBUSTION, 2004, 20:39-41.

[1017] 李芳莉. (2004)熏脐灸治疗女性更年期综合征疗效观察. 针灸临床杂志, 2004, 20:41-42.

[1017]Li Fang-li.(2004) Efficacy of women smoked umbilical moxibustion treatment of menopausal syndrome JOURNAL OF CLINICAL ACUPUNCTURE AND MOXIBUSTION, 2004, 20:41-42.

[1018] 舒丽伟, 王禹. (2004)耳针配合体针治疗坐骨神经痛. 针灸临床杂志, 2004, 20:47-48.

[1018]Shu Li-wei, Wang Yu, (2004) Auricular acupuncture with body acupuncture treatment of sciatica JOURNAL OF CLINICAL ACUPUNCTURE AND MOXIBUSTION, 2004, 20:47-48

[1019] 朱文罡, 刘光英, 任连芳. (2004)腹针治疗腰椎间盘突出症的临床研究. 针灸临床杂志, 2004, 20:23.

[1019]Zhu Wen-gang, Liu Guang-ying, Ren Lian-fang.(2004) Clinical Observation on Abdominal Acupuncture Treatment for Lumbar Intervertebral Disc Herniation.JOURNAL OF CLINICAL ACUPUNCTURE AND MOXIBUSTION, 2004, 20:23.

[1020] 李可欣, 张岩. (2004)头穴交叉刺治疗脑梗塞120例的疗效观察. 针灸临床杂志, 2004, 20:38-40.

[1020]Li Ke-xin, Zhang Yan.(2004) Scalp cross thorn treatment efficacy 120 cases of cerebral infarction JOURNAL OF CLINICAL ACUPUNCTURE AND MOXIBUSTION, 2004, 20:38-40.

[1021] 李华明. (2004)早期针刺加康复法对急性脑血管意外患者偏瘫运动功能的影响. 针灸临床杂志, 2004, 20:8-10.

[1021]Li Hua-ming.(2004) Effect of Early Rehabilitation Act of acupuncture and in patients with acute cerebral vascular accident hemiplegia motor function JOURNAL OF CLINICAL ACUPUNCTURE AND MOXIBUSTION, 2004, 20:8-10.

[1022] 李华宏, 鲍秀山, 吴同芝. (2004)电针配合运动治疗中风后遗症60例临床观察. 针灸临床杂志, 2004, 20:28.

[1022] Li Hua-hong, Bao Xiu-shan, Wu Tong-zhi.(2004) Clinical observation of 60 cases with apoplectic sequel treated with electro-acupuncture combined with movement.JOURNAL OF CLINICAL ACUPUNCTURE AND MOXIBUSTION, 2004, 20:28.

[1023] 张晖, 李继英. (2004)通脑利尿针刺法治疗卒中后排尿障碍的临床观察. 针灸临床杂志, 2004, 20:31-33.

[1023] Zhang Hui, Li Ji-ying.(2004) Clinical observation of acupuncture treating dysfunction of micturition after cerebral apoplexy with themethod of Tong nao li niao.JOURNAL OF CLINICAL ACUPUNCTURE AND MOXIBUSTION, 2004, 20:31-33.

[1024] 孙琦. (2004)耳尖放血结合耳穴贴压法治疗麦粒肿80例. 针灸临床杂志, 2004, 20:34-35.

[1024]Sun Qi.(2004) Apex bloodletting combined treatment of auricular pressure sty 80 cases JOURNAL OF CLINICAL ACUPUNCTURE AND MOXIBUSTION.2004, 20:34-35.

[1025] 潘峰, 叶田, 刘岚. (2004)针刺加手法整复治疗脊柱小关节紊乱肋间神经痛98例. 针灸临床杂志, 2004, 20:9-10.

[1025]Pan Feng, Ye Tian, Liu Lan.(2004) Acupuncture and manipulative treatment of spinal facet joint disorders intercostal neuralgia 98 cases JOURNAL OF CLINICAL ACUPUNCTURE AND MOXIBUSTION, 2004, 20:9-10.

[1026] 潘峰, 叶田, 刘岚. (2004)针刺加艾灸治疗小儿遗尿症56例. 针灸临床杂志, 2004, 20:26.

[1026] Pan Feng, Ye Tian, Liu Lan.(2004)31 patiens with Struma of thyroid treated with acupuncture.JOURNAL OF CLINICAL ACUPUNCTURE AND MOXIBUSTIO, 2004, 20:26.

[1027] 李佩芳, 曹奕, 王二争. (2004)刺络放血对Ⅱ型糖尿病周围神经病变和血液流变学的影响. 针灸临床杂志, 2004, 20:38-40.

[1027]Li Pei-fang, Cao Yi, Wang Er-zheng.(2004) Bloodletting impact on type Ⅱ diabetic peripheral neuropathy and hemorheology JOURNAL OF CLINICAL ACUPUNCTURE AND MOXIBUSTION, 2004, 20:38-40.

[1028] 林志苇, 黎健, 高丽萍, 张夏玲. (2004)肾俞埋线对绝经后骨质疏松症临床疼痛的疗效观察. 针灸临床杂志, 2004, 20:7-8.

[1028] Lin Zhi-wei, Li Jian, Gao Li-ping, Zhang Xia-ling.(2004) Shenshu burying postmenopausal osteoporosis clinical efficacy of pain JOURNAL OF CLINICAL ACUPUNCTURE AND MOXIBUSTION, 2004, 20:7-8.

[1029] 岳玉烈. (2005)中药药物熏蒸结合温针治疗痹证. 针灸临床杂志, 2005, 21:28-30.

[1029]Yue Yu-lie.(2005) Chinese medicines combined with warm acupuncture Arthralgia fumigationJOURNAL OF CLINICAL ACUPUNCTURE AND MOXIBUSTION, 2005, 21:28-30.

[1030] 李峰厚, 葛纪, 蒋晓林. (2005)针刺加超短波治疗腰椎间盘突出症的临床观察. 针灸临床杂志, 2005, 21:34-35.

[1030] Li Feng-hou, Ge Ji, Jiang Xiao-Lin(2005) Clinical observation of lumbar disc herniation treatment with acupuncture and ultrashort JOURNAL OF CLINICAL ACUPUNCTURE AND MOXIBUSTION., 21:34-35.

[1031] 段跃武, 孙雪江, 朱美华. (2005) “醒脑开窍”针刺法治疗急性中风病80例临床研究. 针灸临床杂志, 2005, 21:44-45.

[1031]Duan Yue-wu, Sun Xue-jiang, Zhu Mei-hua.(2005) Clinical study of acute stroke treatment 80 cases CROO "acupuncture JOURNAL OF CLINICAL ACUPUNCTURE AND MOXIBUSTION, 2005, 21:44-45.

[1032] 蒋贵东, 王海莉. (2005)挑刺四缝穴为主治疗小儿疳积疗效观察. 针灸临床杂志, 2005, 21:54-55.

[1032]Jiang Gui-dong, Wang Hai-li.(2005) Prick Sifeng children Malnutrition Treatment Efficacy JOURNAL OF CLINICAL ACUPUNCTURE AND MOXIBUSTION, 2005, 21:54-55.

[1033] 岳玉烈. (2005)针药治疗Ⅱ型糖尿病临床观察. 针灸临床杂志, 2005, 21:16-17.

[1033]Yue Yu-lie.(2005)the clinical observation of diabetes treated with acupuncture and TCM.JOURNAL OF CLINICAL ACUPUNCTURE AND MOXIBUSTION, 2005, 21:16-17.

[1034] 易志龙, 陈伟, 陈春梅, 姜兴鹏. (2005)颞三针加腹针疗法治疗中风后遗症50例疗效观察. 针灸临床杂志, 2005, 21:18-19.

[1034] Yi zhi-long, Chen Wei, Chen Chun-mei, Jiang Xing-peng.(2005) Efficacy of 50 cases of stroke sequelae temporal three-pin plus abdominal acupuncture therapy JOURNAL OF CLINICAL ACUPUNCTURE AND MOXIBUSTION, 2005, 21:18-19.

[1035] 周光辉. (2005)针刺治疗脑卒中患者吞咽障碍的疗效观察. 针灸临床杂志, 2005, 21:32-33.

[1035]Zhou Guang-hui.(2005) Acupuncture for dysphagia stroke patients Efficacy JOURNAL OF CLINICAL ACUPUNCTURE AND MOXIBUSTION, 2005, 21:32-33.

[1036] 左晓峰, 范志慧. (2005)电针配合中药熏蒸治疗增生性膝关节炎65例. 针灸临床杂志, 2005, 21:42-43.

[1036]Zuo Xiao-feng, Fang Zhi-hui.(2005) Electric Acupuncture and Chinese medicine fumigation treatment of hypertrophic osteoarthritis of the knee 65 cases. Journal of Clinical Acupuncture.JOURNAL OF CLINICAL ACUPUNCTURE AND MOXIBUSTION, 2005, 21:42-43.

[1037] 王重新, 宋秋珍. (2005)梅花针叩刺背俞穴治疗慢性疲劳综合症34例疗效观察. 针灸临床杂志, 2005, 21:52-53.

[1037]Wang Chong-xin, Song Qiu-zhen.(2005) Plum-blossom needle treatment of chronic fatigue syndrome, back-shu 34 cases of efficacy.JOURNAL OF CLINICAL ACUPUNCTURE AND MOXIBUSTION, 2005, 21:52-53.

[1038] 张树源, 鲍春玲, 东贵荣. (2005)头穴透刺治疗急性高血压性脑出血神经功能缺损的临床研究. 针灸临床杂志, 2005, 21:12-13.

[1038]Zhang Shu-yuan, Bao Chun-ling, Dong Gui-rong.(2005) Scalp penetration acupuncture clinical study of acute intracerebral hemorrhage treatment of neurological deficits. Clinical AcupunctureJOURNAL OF CLINICAL ACUPUNCTURE AND MOXIBUSTION, 2005, 21:12-13.

[1039] 朱利. (2005)针刺加中药治疗坐骨神经痛的临床观察. 针灸临床杂志, 2005, 21:25.

[1039]Zhu Li.(2005) Acupuncture and traditional Chinese medicine treatment of sciatica Clinical Journal of Clinical Acupuncture JOURNAL OF CLINICAL ACUPUNCTURE AND MOXIBUSTION, 2005, 21:25.

[1040] 周文丽, 考军, 于长青, [马小业](http://search.cnki.com.cn/Search.aspx?q=author:马小业) . (2005)艾灸足三里治疗顺铂所致迟发性呕吐的临床观察. 针灸临床杂志, 2005, 21:47-48.

[1040]Zhou Wen-li, Kao Jun, Yu Chang-qing, Ma Xia-ye(2005) Moxibustion foot clinical observation in the treatment of cisplatin-induced three delayed vomiting. Journal of Clinical Acupuncture, JOURNAL OF CLINICAL ACUPUNCTURE AND MOXIBUSTION, 2005, 21:47-48.

[1041] 程爱萍, 张波, 陈日新. (2005)穴位注射治疗背肌筋膜炎35例临床疗效观察. 针灸临床杂志, 2005, 21:16-17.

[1041]Cheng Ai-ping, Zhang Bo, Chen Ri-xin(2005 Point injection therapy back muscles fasciitis Clinical efficacy 35 casesJOURNAL OF CLINICAL ACUPUNCTURE AND MOXIBUSTION, 2005, 21:16-17.

[1042] 唐平平, 王樟连, 陈利芳. (2005)辰戌两时针刺治疗失眠的疗效对比. 针灸临床杂志, 2005, 21:32-33.

[1042] Wang Zhanglian, Chen Lifang. (2005) when two Chen Xu. Comparison of the efficacy of acupuncture treatment of insomnia Tangping Ping, JOURNAL OF CLINICAL ACUPUNCTURE AND MOXIBUSTION, 2005, 21:32-33.

[1043] 洪建云. (2005)深部夹脊刺治疗腰椎间盘突出症的疗效观察. 针灸临床杂志, 2005, 21:33-34.

[1043]Hong Jian-yun.(2005)Jiaji deep stab efficacy for treatment of lumbar disc herniation

JOURNAL OF CLINICAL ACUPUNCTURE AND MOXIBUSTION, 2005, 21:33-34.

[1044] 谢建谋, 路月香. (2005)叉三针加穴位注射治疗三叉神经痛35例. 针灸临床杂志, 2005, 21:37-38.

[1044]Xie Jian-mo. Lu Yue-xiang.(2005) Fork three acupuncture and Point Injection treatment of trigeminal neuralgia. JOURNAL OF CLINICAL ACUPUNCTURE AND MOXIBUSTION., 2005, 21:37-38.

[1045] 顾旭东, 傅建明, 姚云海. (2005) 电针八髎穴为主治疗脊髓损伤排尿障碍64例疗效观察. 针灸临床杂志, 2005, 21:47-48.

[1045]Gu Xu-dong. Fu Jian.ming. Yao Yun-hia.(2005). EA Baliao points for treatment of spinal cord injury voiding dysfunction JOURNAL OF CLINICAL ACUPUNCTURE AND MOXIBUSTION. 21:47-48.

[1046] 张铁英, 孙琳, 黄梅颖. (2005)辨经取穴治疗神经根型颈椎病360例疗效观察. 针灸临床杂志, 2005, 21:18-19.

[1046] Zhang Tie-ying. Sun Lin. Huang Mei-yin. (2005) Identified by the acupoints treatment of nerve root type cervical spondylosis JOURNAL OF CLINICAL ACUPUNCTURE AND MOXIBUSTION, 2005, 21:18-19.

[1047] 莫睿, 刘波. (2005)健脾和胃、理气通降法针刺治疗糖尿病胃轻瘫的临床观察. 针灸临床杂志, , 21:20-21.

[1047]Mo Rui. Liu Bo.(2005) Spleen and stomach, qi through clinical observation of acupuncture treatment reduced Diabetic Gastroparesis. JOURNAL OF CLINICAL ACUPUNCTURE AND MOXIBUSTION, , 21:20-21.

[1048] 詹程胹, 汪芳俊. (2005)针刺治疗慢性功能性便秘的临床观察. 针灸临床杂志, 2005, 21:24-25.

[1048]Zhang Chen-mian, Wang Fang-jun. (2005) Clinical observation of acupuncture treatment of chronic functional constipation JOURNAL OF CLINICAL ACUPUNCTURE AND MOXIBUSTION, 2005, 21:24-25.

[1049] 李晓宁, 姚素媛, 李晓伟, 倪金霞, 盛国滨. (2005)电针治疗无抑制性神经源性膀胱120例临床研究. 针灸临床杂志, 2005, 21:40-41.

[1049]Li Xiao-ning, Yao Su-yuan, Li Xiao-wei, Ni Jin-xia, Sheng Guo-bin.(2005) Clinical study of 120 cases with repressed neuron bladder treated with electro- acupuncture JOURNAL OF CLINICAL ACUPUNCTURE AND MOXIBUSTION, 2005, 21:40-41.

[1050] 王敏, 王敏华, 梁冰. (2005) 电针治疗对脑梗死偏瘫急性期运动功能的影响. 针灸临床杂志, 21:43-44.

[1050] Wang Ming, Wang-Ming hua, Liang-bin.(2005) Acupuncture treatment without inhibiting neurogenic bladder JOURNAL OF CLINICAL ACUPUNCTURE AND MOXIBUSTION, 21:43-44.

[1051] 张吉玉, 程绍鲁. (2005)透刺配合穴注治疗顽固性面瘫40例疗效观察. 针灸临床杂志, , 21:49-50.

[1051]Zhang Ji-yu, Chen Shao-lu.(2005) Through Acupuncture point injection with the treatment of intractable facial paralysis Cases JOURNAL OF CLINICAL ACUPUNCTURE AND MOXIBUSTION, , 21:49-50.

[1052] 陈晓军, 吴凌云, 张家维. (2005)温针灸治疗缺血性中风56例疗效观察. 针灸临床杂志, , 21:55-56.

[1052]Chen Xiao-jun, Wu Ling-yun, Zhang Jia-wei.(2005) 56 cases of ischemic stroke warm acupuncture treatment efficacy.JOURNAL OF CLINICAL ACUPUNCTURE AND MOXIBUSTION, , 21:55-56.

[1053] 赵时碧, 张丽, 赵颜利, [陈建国](http://search.cnki.com.cn/Search.aspx?q=author:陈建国), [汤君彦](http://search.cnki.com.cn/Search.aspx?q=author:汤君彦), 等. (2005)赵氏雷火灸治疗常年性变应性鼻炎的临床研究. 针灸临床杂志, 2005, 21:19-21.

[1053] Zhao Shi-bi, Zhang Li, Zhao Yan-li, Zhao Jian-guo, Tang Jun-yan, et al, (2005 )Clinical studies Zhao thunderbolt moxibustion treatment of perennial allergic rhinitis.JOURNAL OF CLINICAL ACUPUNCTURE AND MOXIBUSTION, 2005, 21:19-21.

[1054] 肖平, 孙远征, 侯慧先. (2005)督脉埋针治疗女性黄褐斑的临床研究. 针灸临床杂志, 2005, 21:21-22.

[1054]Xiao Ping, Sun Yuan-zheng, Hou Hui-xain.(2005).Clinical studies of female chloasma Du buried acupuncture JOURNAL OF CLINICAL ACUPUNCTURE AND MOXIBUSTION, 2005, 21:21-22.

[1055] 程继君, 费建中, 吴红瑛. (2005)醒脑开窍针刺配合康复训练治疗闭合性颅脑损伤术后73例. 针灸临床杂志, 2005, 21:38-39.

[1055] Chen Ji-jun, Fei Jian-zhong, Wu Hong-ying.(2005)XNKQ acupuncture treatment of 73 cases with rehabilitation training after closed head injury.JOURNAL OF CLINICAL ACUPUNCTURE AND MOXIBUSTION, 2005, 21:38-39.

[1056] 吉学群, 王春梅, 刘占芬, 张智龙. (2005)针刺崇骨穴治疗假球麻痹的临床研究. 针灸临床杂志, 2005, 21:40-41.

[1056] Ji Xue-qun, Wang Chun-mei, Liu Zhan-fen, Zhang Zhi-long(2005)Clinical studies of acupuncture point in the treatment of bone fixing Chong paralysis.. JOURNAL OF CLINICAL ACUPUNCTURE AND MOXIBUSTION, 2005, 21:40-41.

[1057] 刘彬, 苏凤, 燕建新, [董石春](http://search.cnki.com.cn/Search.aspx?q=author:董石春) . (2005) 恒磁场并针刺治疗面神经麻痹的效果观察. 针灸临床杂志, 2005, 21:4-5.

[1057]Liu Bin, Su Feng, Yang Jian-xin, Dong Shi-chun(2005) Constant magnetic field and the acupuncture treatment effect observed facial paralysis. JOURNAL OF CLINICAL ACUPUNCTURE AND MOXIBUSTION, 2005, 21:4-5.

[1058] 常英. (2005)针灸配合推拿治疗腰椎间盘突出症148例. 针灸临床杂志, 2005, 21:18.

[1058]Chang Ying(2005) Acupuncture and massage therapy with 148 cases of lumbar disc herniation. JOURNAL OF CLINICAL ACUPUNCTURE AND MOXIBUSTION, 2005, 21:18.

[1059] 林国华, 李丽霞, 李艳慧, [张汉梁](http://search.cnki.com.cn/Search.aspx?q=author:张汉梁) . (2005)头体针结合治疗缺血性中风失语症的临床观察. 针灸临床杂志, 2005, 21:25-26.

[1059]Lin Guo-hua, Li Li-xia, Li Yan-hui, Zhang Han-liang(2005) Clinical observation of head and body acupuncture treatment of ischemic stroke aphasia..JOURNAL OF CLINICAL ACUPUNCTURE AND MOXIBUSTION, 2005, 21:25-26.

[1060] 刘建萌. (2005)浮针治疗颈椎病的临床观察. 针灸临床杂志, 2005, 21:31-32.

[1060] Liu Lian-meng(2005) Clinical observation of cervical spondylosis float needle therapy. JOURNAL OF CLINICAL ACUPUNCTURE AND MOXIBUSTION, 2005, 21:31-32.

[1061] 洪建云. (2005)化脓灸治疗支气管哮喘临床研究. 针灸临床杂志, 2005, 21:35-36.

[1061]Hong Jian-yun(2005) Clinical studies Huanongjiu treatment of bronchial asthma. JOURNAL OF CLINICAL ACUPUNCTURE AND MOXIBUSTION, 2005, 21:35-36.

[1062] 李勇, 李滋平, 符文彬. (2005)舌针疗法治疗中风后吞咽障碍的临床研究. 针灸临床杂志, 2005, 21:7-8.

[1062]Li Yong, Li Zi-ping, Fu Wen-bing(2005). Clinical study of post-stroke dysphagia tongue acupuncture therapy. JOURNAL OF CLINICAL ACUPUNCTURE AND MOXIBUSTION, 2005, 21:7-8.

[1063] 陈伟, 姜兴鹏. (2005)腹针配合外敷骨增散治疗退行性膝关节炎疗效观察. 针灸临床杂志, 2005, 21:16.

[1063]Chen Wei, Jiang Xing-peng(2005) Abdominal Acupuncture topical bone degenerative osteoarthritis of the knee by casual treatment efficacy.JOURNAL OF CLINICAL ACUPUNCTURE AND MOXIBUSTION, 2005, 21:16.

[1064] 鲍春龄, 张丽荣, 东贵荣. (2005)头穴透刺对急性高血压性脑出血患者血浆内皮素、神经元特异性烯醇化酶的影响. 2005, 21:21-22.

[1064] Bao Chun-lin, Zhang Li-rong, Dong Gui-rong(2005) Scalp penetration acupuncture for patients with acute intracerebral hemorrhage affecting plasma endothelin nerve, neuron-specific enolase. JOURNAL OF CLINICAL ACUPUNCTURE AND MOXIBUSTION, 2005, 21:21-22.

[1065] 侯路成, 李黄彤. (2005)梅花针配合埋线治疗顽固性面瘫42例疗效观察. 针灸临床杂志, 2005, 21:29.

[1065] Hou Lu-cheng, Li Huang-tong(2005) Plum Acupuncture and burying treatment of intractable facial paralysis observation of 42 cases. JOURNAL OF CLINICAL ACUPUNCTURE AND MOXIBUSTION, 2005, 21:29.

[1066] 耿瑞荣. (2005)电针治疗周围性面神经麻痹139例疗效观察. 针灸临床杂志, 2005, 21:36-37.

[1066] Geng Rui-rong(2005) EA 139 cases of peripheral facial paralysis treatment efficacy. JOURNAL OF CLINICAL ACUPUNCTURE AND MOXIBUSTION, 2005, 21:36-37.

[1067] 沈克艰, 吴梅珍. (2005)伏天铺灸对慢性盆腔炎患者近期补体的影响. 针灸临床杂志, 2005, 21:50-51.

[1067]Shen Ke-jian, Wu Mei-zhen(2005)Dog days of summer shop Moxibustion on chronic pelvic inflammatory complement recent patients. JOURNAL OF CLINICAL ACUPUNCTURE AND MOXIBUSTION, 2005, 21:50-51.

[1068] 李新红, 周君, 李先果, 郑丽芳, 王泽涛. (2005)针刺夹脊穴治疗中风偏瘫痉挛状态35例临床观察. 针灸临床杂志, 2005, 21:7-8.

[1068]Li Xin-hong, Zhuo Jun, Li Xian-guo, Zhen Li-fen, Wang Ze-tao(2005). Clinical observation of 35 cases of hemiplegia spasticity Jiaji acupuncture treatment. JOURNAL OF CLINICAL ACUPUNCTURE AND MOXIBUSTION., 21:7-8.

[1069] 王新玲, 逯俭, 宋连会. (2005)温针治疗腰椎间盘突出症临床疗效观察. 针灸临床杂志, 2005, 21:11-12.

[1069]Wang Xin=ling, Lu Jian, Song Lian-hui(2005) Clinical efficacy of acupuncture treatment of lumbar disc herniation warm. JOURNAL OF CLINICAL ACUPUNCTURE AND MOXIBUSTION, 2005, 21:11-12.

[1070] 廖华薇. (2005)经颅多谱勒检测大脑中动脉血流观察头针治疗脑动脉硬化的疗效. 针灸临床杂志, 2005, 21:15-16.

[1070] Liao Hua-wei(2005) Cerebral arteriosclerosis efficacy of transcranial Doppler detection of middle cerebral artery blood flow was observed scalp treatment. JOURNAL OF CLINICAL ACUPUNCTURE AND MOXIBUSTION, 2005, 21:15-16.

[1071] 鲍春龄, 宋春华, 张淑源, [刘瑛琦](http://search.cnki.com.cn/Search.aspx?q=author:刘瑛琦), [张丽荣](http://search.cnki.com.cn/Search.aspx?q=author:张丽荣). (2005)针刺曲池穴治疗慢性荨麻疹56例. 针灸临床杂志, 2005, 21:45-46.

[1071] Bao Chun-lin, Song Chun-hua, Zhang Shu-yuan, Liu Ying-qi, Zhang Li-rong(2005)Quchi acupuncture treatment of chronic urticaria. JOURNAL OF CLINICAL ACUPUNCTURE AND MOXIBUSTION, 2005, 21:45-46.

[1072] 赵宏, 刘志顺, 杨涛. (2005)电针太阳穴治疗椎基底动脉供血不足临床观察. 针灸临床杂志, 2005, 21:8-9.

[1072]Zhao Hong, Liu Zhi-shun, Yang Tao(2005) EA temples treatment of vertebrobasilar insufficiency clinical observation. JOURNAL OF CLINICAL ACUPUNCTURE AND MOXIBUSTION, 2005, 21:8-9.

[1073] 黄俏敏, 陈克正. (2005)针药配合拔罐治疗周围性面神经麻痹临床观察. 针灸临床杂志, 2005, 21:6-7.

[1073] Huang Qiao-ming, Chen Ke-zheng(2005) Acupuncture and cupping therapy clinical observation of peripheral facial paralysis. JOURNAL OF CLINICAL ACUPUNCTURE AND MOXIBUSTION, 2005, 21:6-7.

[1074] 卢静, 贾天贵, 何刚, [汤业军](http://search.cnki.com.cn/Search.aspx?q=author:汤业军), [刘全让](http://search.cnki.com.cn/Search.aspx?q=author:刘全让). (2005)杵针疗法治疗脑动脉粥样硬化70例. 针灸临床杂志, 2005, 21:29-31.

[1074]Lu Jing, Jia Tian-gui, He Gang, Tang Ye-jun, Liu Quan-rang(2005) Pestle acupuncture treatment of cerebral atherosclerosis 70 cases. JOURNAL OF CLINICAL ACUPUNCTURE AND MOXIBUSTION, 2005, 21:29-31.

[1075] 严付红. (2006)壮医药线点灸治疗脾虚泄泻42例. 针灸临床杂志, 2006, 22:43.

[1075]Yan Fu-hong(2006)Zhuang medicine thread moxibustion treatment of 42 cases of diarrhea spleen. JOURNAL OF CLINICAL ACUPUNCTURE AND MOXIBUSTION, 2006, 22:43.

[1096] 周鸿飞, 黄春元, 鞠庆波, 田迎春. (2006)眼针与头针对脑梗塞进行分期治疗的临床观察. 针灸临床杂志, 2006, 22:3-5.

[1096]Zhou Hong-fei, Huang Chun-yuan, Ju Qing-bo, Tian Yin-chun.(2006) Clinical eye pin and head for the treatment of cerebral infarction staging. JOURNAL OF CHANGCHUN COLLEGE OF TRADITIONAL CHINESE MEDICINE , 2006, 22:3-5.

[1097] 庞军, 雷龙鸣, 陈家兴, [黄锦军](http://search.cnki.com.cn/Search.aspx?q=author:黄锦军), [黄永](http://search.cnki.com.cn/Search.aspx?q=author:黄永), 等. (2006)推拿配合电针治疗腰椎间盘突出症临床观察. 针灸临床杂志, 2006, 22:43-45.

[1097]Pang Jun, Lei Long-ming, Chen Jia-xin, Huang Jin-jun, Huang Yong, et al.(2006).Clinical observation of Massage with acupuncture treatment of lumbar disc herniation .JOURNAL OF CHANGCHUN COLLEGE OF TRADITIONAL CHINESE MEDICINE , 2006, 22:43-45.

[1098] 李艳梅, 宋立中, 陈卫. (2006)温针灸配合中药治疗输卵管不通不孕症研究. 针灸临床杂志, 2006, 22:6-8.

[1098]Li Yan-mei, Song Li-zhong, Chen Wai.(2006)Clinical study on oviduct blocking treated with acupuncture and moxibustion and drinking "Tong guan tang".JOURNAL OF CHANGCHUN COLLEGE OF TRADITIONAL CHINESE MEDICINE, 2006, 22:6-8.

[1099] 旷秋和. (2006)火针治疗膝骨性关节炎50例疗效观察. 针灸临床杂志, 2006, 22:19-20.

[1099]Kuang Qiu-he.(2006)Fire needle treatment of knee osteoarthritis Observation of 50 Cases. JOURNAL OF CHANGCHUN COLLEGE OF TRADITIONAL CHINESE MEDICINE , 2006, 22:19-20.

[1100] 李光海, 耿寅卯, 张素娟, 马占学，胡银虎. (2006)头针配合药物治疗抑郁症的疗效观察. 针灸临床杂志22:27-28.

[1100] Li Guang-hai, Geng Yan-mou., Zhang Su-juan, Ma Zhang-xue, Hu Yin-hu.(2006)Clinical observation on depression treated by Scalp combined with western medicine. JOURNAL OF CHANGCHUN COLLEGE OF TRADITIONAL CHINESE MEDICINE 22:27-28.

[1101] 王凯. (2006)电针配合中药治疗失眠症的临床观察. 针灸临床杂志22:34-35.

[1101] Wang Kai.(2006)Clinical observation on electro- acupuncture combined with herbs therapy for insomnia.JOURNAL OF CHANGCHUN COLLEGE OF TRADITIONAL CHINESE MEDICINE 22:34-35.

[1102] 徐莺莺, 孙远征. (2006)腰背部透穴法治疗经前期综合征的疗效观察. 针灸临床杂志22:37-38.

[1102]Xu Ying-ying, Sun Yuan-zheng.(2006)Observation on the effect of point penetration method and medication in treatment of premenstrual syndrome. JOURNAL OF CHANGCHUN COLLEGE OF TRADITIONAL CHINESE MEDICINE 22:37-38.

[1103] 何永昌. (2006)阿是穴压灸法治疗拇指屈肌腱鞘炎疗效观察. 针灸临床杂志22:41-42.

[1103] He Yong-chang.(2006) Clinical observation on point Eshi moxibustion - pressing treatment of tenosynovitis in thumb.JOURNAL OF CHANGCHUN COLLEGE OF TRADITIONAL CHINESE MEDICINE 22:41-42.

[1104] 杨丹红, 陈华德, (2006)方针针刺配合药物对帕金森病康复治疗的疗效观察. 针灸临床杂志22:16-17.

[1104]Yang Dan-hua, Chen Hua-de.(2006) Efficacy of acupuncture combined with the principle of the Parkinson's disease drug rehabilitation.JOURNAL OF CHANGCHUN COLLEGE OF TRADITIONAL CHINESE MEDICINE 22:16-17.

[1105] 杨续艳, 高维滨. (2006)电针治疗脑卒中后尿失禁的临床观察. 针灸临床杂志22:33-34.

[1105]Yang Xu-yan, Gao Wei-bin.(2006)Clinical observation of acupuncture treatment of urinary incontinence after stroke.JOURNAL OF CHANGCHUN COLLEGE OF TRADITIONAL CHINESE MEDICINE 22:33-34.

[1106] 李子勇, 老锦雄. (2006)面部挂针加刺络拔罐治疗痤疮52例的疗效观察. 针灸临床杂志22:11-12.

[1106]Li Zi-yong, Lao Jin-xiong.(2006) Observation On The Therapeutic Effects of 52 cases of Acne Treated By the Combination of Facial Acupucnture and Pricking. JOURNAL OF CHANGCHUN COLLEGE OF TRADITIONAL CHINESE MEDICINE 22:11-12.

[1107] 曾红文, 聂斌. (2006)电针、穴位埋线合耳压综合治疗单纯性肥胖症的临床观察. 针灸临床杂志22:13-14.

[1107]Zeng Hong-wen, Nie Bin.(2006)The Clinical Observation on Electric Acupuncture, Catgut implanation at Acupolnts and Auricular Point sticking for Treatment of Simple Obsesity.JOURNAL OF CHANGCHUN COLLEGE OF TRADITIONAL CHINESE MEDICINE 22:13-14.

[1108] 李丽霞, 林国华, 张汉梁, 刘醒如. (2006)电针治疗腰椎间盘突出症的疗效观察. 针灸临床杂志22:36-37.

[1108]Li Li-xia, Lin Guo-hua, Zhang Han-liang, Liu Xing-ru.(2006)The clinical observation of herniation of internertebral disk treated by the electric acupucnture. JOURNAL OF CHANGCHUN COLLEGE OF TRADITIONAL CHINESE MEDICINE 22:36-37.

[1109] 范桂滨. (2006)针刺导气法治疗急性腰扭伤临床观察. 针灸临床杂志22:43.

[1109] Fan Gui-bin.(2006) Clinical observation of acute lumbar sprain acupuncture Treatment of Gas. JOURNAL OF CHANGCHUN COLLEGE OF TRADITIONAL CHINESE MEDICINE 22:43.

[1110] 罗和平, 王邦博. (2006)分组取穴、交替针刺并电针治疗面神经炎的临床观察. 针灸临床杂志22:10-11.

[1110] Luo He-ping, Wang Bang-bo.(2006)Clinical observation on needling and electroneedling alternate two groups of acupoints on facial neuritis. JOURNAL OF CHANGCHUN COLLEGE OF TRADITIONAL CHINESE MEDICINE 22:10-11.

[1111] 林忆平, 张京晶. (2006)挂针为主配合拔罐治疗顽固性面肌痉挛. 针灸临床杂志22:25-26.

[1111]Lin Yi-ping, Zhang Jin-jin.(2006)The observation of mainly using GUA ZHEN and cupping to treat hemifacial spams. JOURNAL OF CHANGCHUN COLLEGE OF TRADITIONAL CHINESE MEDICINE 22:25-26.

[1112] 罗平, 张淑忆. (2006)醒脑开窍针法治疗卒中后肢体功能障碍78例临床研究. 针灸临床杂志22:41-42.

[1112] Luo Ping, Zhang Shu-yi, (2006)Limb dysfunction after treatment, 78 cases of clinical research in stroke activating Acupuncture. JOURNAL OF CHANGCHUN COLLEGE OF TRADITIONAL CHINESE MEDICINE 22:41-42.

[1113] 黄国付, 张红星, 张唐法. (2006)电针夹脊穴对腰椎间盘突出症患者血浆一氧化氮及自由基代谢的影响. 针灸临床杂志22:5-7.

[1113] Huang Guo-fu, Zhang Hong-xing, Zhang Tang-fa. (2006)Influence of electroacupuncture at Jiaji acupoint on the levels of NO, SOD and MDA in patients with lumbar disc herniation. JOURNAL OF CHANGCHUN COLLEGE OF TRADITIONAL CHINESE MEDICINE 22:5-7.

[1114] 姜桂美, 贾超. (2006)夹脊穴不同取穴与针刺方法治疗腰椎间盘突出症的临床对比观察. 针灸临床杂志22:8-9.

[1114] Jiang Gui-mei, Jia Chao.(2006) Clinical observation Jiaji different acupoints and acupuncture treatment of lumbar disc herniation.. JOURNAL OF CHANGCHUN COLLEGE OF TRADITIONAL CHINESE MEDICINE 22:8-9.

[1115] 李雪岩, 孙忠人. (2006)分期针刺治疗脑梗死的临床疗效对比研究. 针灸临床杂志22:10-11.

[1115]Li Xue-yan, Sun Zhong-ren. (2006)Clinically controlled study on phased needling method for treatment of cerebral infarction. JOURNAL OF CHANGCHUN COLLEGE OF TRADITIONAL CHINESE MEDICINE 22:10-11.

[1116] 陈丽萍, 申永涛, 刘娟. (2006)项针配合康复训练治疗急性脑卒中后吞咽障碍的疗效观察. 针灸临床杂志22:15-16.

[1116] Sun Li -ping, Shen Yong-tao, Liu Juan.(2006)The clinical observation of treatment on acute cerebral apopley aglatition by neck acupuncture adds conditioning. JOURNAL OF CHANGCHUN COLLEGE OF TRADITIONAL CHINESE MEDICINE 22:15-16.

[1117] 白鹏, 王影, 赵吉平, 孙河，包大鹏. (2006)电针与单纯针刺治疗视网膜色素变性的临床对比研究. 针灸临床杂志22:17-19.

## [1117] Bai peng, Wang Ying, Zhao Ji-ping, Sun he, Bao Da-peng.(2006) The comparable clinical study on the treatment of retinitis pigmentosa with electro-acupuncture and pure acupuncture.[**JOURNAL OF CLINICAL ACUPUNCTURE AND MOXIBUSTION**](http://c.g.wanfangdata.com.cn/Periodical-zjlczz.aspx) 22:17-19.

[1118] 曾学清, 滕东时, 杨涛, 何小花. (2006)醒脑开窍针刺法配合现代促通技术改善脑卒中患者肢体功能障碍的临床研究. 针灸临床杂志22:20-21.

[1118] Zue Xue-qing, Teng Dong-shi, Yang Tao, He Xiao-hua(2006)Clinical study on acupuncture method of inducing resuscitation with modern facilitation technique on imprvoe Limb dysfunction of cerebral apoplexy patients.[JOURNAL OF CLINICAL ACUPUNCTURE AND MOXIBUSTION](http://c.g.wanfangdata.com.cn/Periodical-zjlczz.aspx) 22:20-21.

[1119] 姚玉芳, 吴成长, 孟云凤. (2006)耳针加中药治疗寻常痤疮30例临床疗效观察. 针灸临床杂志22:23-24.

[1119]Yao Yu-fang, Wu Cheng-zhang, Meng Yun-feng.(2006)Clinical observation of 30 case on auricular acupuncture with Chinese Medicine on treating common acne.[JOURNAL OF CLINICAL ACUPUNCTURE AND MOXIBUSTION](http://c.g.wanfangdata.com.cn/Periodical-zjlczz.aspx) 22:23-24.

[1120] 高维滨, 刘勇, 倪金霞, 朱文增 李晓宁. (2006)项针治疗假性延髓麻痹的临床疗效再评价. 针灸临床杂志22:8-10.

## [1120]Gao Wei-bing, Liu Yong, Ni Jin-xia, Zhu Wen-zeng. Li Xiao-ning.(2006)The evaluation of the clinical efficacy on the neekacupuncture treatment of pseudabulbar paralysis.[**JOURNAL OF CLINICAL ACUPUNCTURE AND MOXIBUSTION**](http://c.g.wanfangdata.com.cn/Periodical-zjlczz.aspx) 22:8-10.

[1121] 徐波克, 朱冬梅, 孙申田. (2007)经颅重复针刺对60例面神经损伤修复的临床研究. 针灸临床杂志23:13-14.

## [1121] Xu Bo-ke, Zhu Dong-mei, Sun Shen-tian.(2007)The clinical study of recovery of injured facial never by trans-skull repeated acupuncture different point of head

[JOURNAL OF CLINICAL ACUPUNCTURE AND MOXIBUSTION](http://c.g.wanfangdata.com.cn/Periodical-zjlczz.aspx) 23:13-14.

[1122] 龙晖. (2007)手针治疗足跟痛症102例疗效观察. 针灸临床杂志23:21-22.

[1122] Long Hui(2007)The curative effect observation on hand needle on treating heel pains.[JOURNAL OF CLINICAL ACUPUNCTURE AND MOXIBUSTION](http://c.g.wanfangdata.com.cn/Periodical-zjlczz.aspx) 23:21-22.

[1123] 廖小七, 唐华, 肖鹏. (2007)电针中髎穴为主治疗膀胱过度活动症35例. 针灸临床杂志23:35-36.

[1123] Liao Xiao-qi, Tang Huan, Xiao Peng.(2007)35 case on electroacupuncture Ciliao acupoint on treating overactive bladder.JOURNAL OF CLINICAL ACUPUNCTURE AND MOXIBUSTION 23:35-36.

[1124] 卢静, 王瑛. (2007)针刺配合刺血拔罐治疗腰椎间盘突出症63例. 针灸临床杂志23:16-17.

## [1124]Lu Jing, Wang Ying.(2007)Clinical observations of curative effect of lumbar disc prolapse by acupuncture combined with punctural cupping.[**JOURNAL OF CLINICAL ACUPUNCTURE AND MOXIBUSTION**](http://c.g.wanfangdata.com.cn/Periodical-zjlczz.aspx) 23:16-17.

[1125] 赵辉, 谭乐成, 王艳. (2007)针刺配合超短波治疗过敏性鼻炎临床观察. 针灸临床杂志23:20-21.

## [1125]Zhao Hui, Yan Le-cheng, Wang Yang.(2007) Clinical observations on therapeutic effect of acupuncture coordinates with ultra short waves therapy on anaphylactic rhinitis [**JOURNAL OF CLINICAL ACUPUNCTURE AND MOXIBUSTION**](http://c.g.wanfangdata.com.cn/Periodical-zjlczz.aspx) 23:20-21.

[1126] 赵一宇, 刘红玲. (2007)头针配合康复手法治疗偏瘫肩关节半脱位. 针灸临床杂志23:20-21.

[1126] Zhao Yi-yun, Liu Hong-ling.(2007)Scalp acupuncture combined with rehabilitation technique on treating hemiplegic shoulder joint subluxation. JOURNAL OF CLINICAL ACUPUNCTURE AND MOXIBUSTION 23:20-21.

[1127] 杜翠云, 李妍. (2007)针刺日月穴治疗慢性胆囊炎的临床观察. 针灸临床杂志23:35-36.

## [1127]Du Cui-yun, Li Yan.(2007) Clinical observations on the effect of point Riyue acupuncture on the improvement of chronic cholecystitis's symptoms

[JOURNAL OF CLINICAL ACUPUNCTURE AND MOXIBUSTION](http://c.g.wanfangdata.com.cn/Periodical-zjlczz.aspx) 23;35-36.

[1128] 张红星, 张唐法, 周利, 万文俊 黄国付. (2007)电针丰隆穴治疗高脂血症临床研究. 针灸临床杂志23:7-8.

[1128]Zhang Hong-xing, Zhang Tang-fa, Zhou Li. Wan Wen-jun. Huang Guo-fu.(2007)Clinical research of electroacupuncture at Fenglong point on treating hyperlipidemia. JOURNAL OF CLINICAL ACUPUNCTURE AND MOXIBUSTION 23:7-8.

[1129] 庞国军, 王可博. (2007)醒脑开窍针法治疗中风恢复期疗效观察. 针灸临床杂志23:19-20.

## [1129]Pang Guo-jun, Wang Ke-bo.(2007)Observations on therapeutic effect of 'XNKQ' acupuncture on the stages of stroke recovery. [**JOURNAL OF CLINICAL ACUPUNCTURE AND MOXIBUSTION**](http://c.g.wanfangdata.com.cn/Periodical-zjlczz.aspx) 23:19-20.

[1130] 石育才. (2007)电针治疗带状疱疹后遗神经痛31例. 针灸临床杂志23:27-28.

## [1130]Shi Yun-cai.(2007) Electro-acupuncture for treatment of zonal - herpetic neuralgia [**JOURNAL OF CLINICAL ACUPUNCTURE AND MOXIBUSTION**](http://c.g.wanfangdata.com.cn/Periodical-zjlczz.aspx) 23:27-28.

[1131] 廖军芳. (2007)点刺金津玉液穴治疗中风后运动性失语症52例. 针灸临床杂志23:35-36.

[1131]Liao Jun-fang.(2007) 52 case of prinkig on Jin Yuye point on treating motor aphasia after stroke. JOURNAL OF CLINICAL ACUPUNCTURE AND MOXIBUSTION.23:35-36.

[1132] 朱秀平, 朱美群, 黄少姬. (2007)穴位贴敷配合穴位埋线治疗慢性浅表性胃炎的临床疗效观察. 针灸临床杂志23:1-3.

## [1132] Zhu Xiu-ping, Zhu Mei-qun, Huang Shao-ji.(2007)Clinical observation on the therapeutic effect of chronic superfacial gastricism treated by acupoint sticking with catgut ligation. [**JOURNAL OF CLINICAL ACUPUNCTURE AND MOXIBUSTION**](http://c.g.wanfangdata.com.cn/Periodical-zjlczz.aspx). 23:1-3.

[1133] 孙远征, 王春英. (2007)针刺结合脑超声对脑梗死恢复期疗效观察. 针灸临床杂志23:21-22.

## [1133]Sun Yuan-zheng, Wang Chun-ying.(2007)Observations on the Therapeutic effect of the convalescence of cerebral ultrasonic treated by combining acupuncture and cerebral ultrasonic. [**JOURNAL OF CLINICAL ACUPUNCTURE AND MOXIBUSTION**](http://c.g.wanfangdata.com.cn/Periodical-zjlczz.aspx) 23:21-22.

[1134] 朱爱军. (2007)大灸百会、大椎刺血治疗椎动脉型颈椎病. 针灸临床杂志23:29-30.

[1134]Zhu Ai-jun, (2007) Moxibustion on Baihui point and Dazhui blood pricking on treating cervical spondylosis of vertebral artery type.JOURNAL OF CLINICAL ACUPUNCTURE AND MOXIBUSTION 23:29-30.

[1135] 苏云海, 蔡岩松. (2007)透穴针刺治疗动眼神经麻痹的临床观察. 针灸临床杂志23:31.

[1135]Su Yun-hai, Cai Yan-song.(2007) Clinical observation of acupoint penetration acupuncture treatment on treating oculomotor nerve palsy. JOURNAL OF CLINICAL ACUPUNCTURE AND MOXIBUSTION 23:31.

[1136] 丁德光, 罗惠平, 焦扬. (2008)长时间留针治疗失眠的临床研究. 针灸临床杂志, 24:10-11.

[1136] Ding De-guang, Luo Hui-ping, Jiao Yang.(2008) Clinical research on long time retaining needle on treating insomnia.JOURNAL OF CLINICAL ACUPUNCTURE AND MOXIBUSTION 24:10-11.

[1137] 徐凯. (2008)针刺疗法治疗小儿脑瘫的疗效观察. 针灸临床杂志24:11-12.

[1137]Xu Kai.(2008)Observation on therapeutic effect of acupuncture therapy for treatment of children with cerebral palsy. JOURNAL OF CLINICAL ACUPUNCTURE AND MOXIBUSTION 24:11-12.

[1138] 沈王明. (2008)扶突穴为主治疗脑卒中假性球麻痹. 针灸临床杂志24:17-18.

[1138] Shen Wang-ming.(2008)Treatment of stroke pseudobulbar paralysis which mainly futu acupoint.JOURNAL OF CLINICAL ACUPUNCTURE AND MOXIBUSTION 24:17-18.

[1139] 周仲瑜. (2008)齐刺法治疗梨状肌损伤综合症疗效观察. 针灸临床杂志24:22.

[1139] Zhou Zhong-yu(2008)Curative effect observe on puncturing on treating Piriformis injury syndrome. JOURNAL OF CLINICAL ACUPUNCTURE AND MOXIBUSTION 24：22.

[1140] 李春华, 伍萍. (2008)电针结合中药治疗带状疱疹后遗神经痛35例. 针灸临床杂志24:29-30.

[1140]Li Chun-hua, Wu Ping.(2008) 35 cases of traditional Chinese medicine in the treatment of postherpetic neuralgia with Acupuncture. JOURNAL OF CLINICAL ACUPUNCTURE AND MOXIBUSTION 24:29-30.

[1141] 胡幼平, 卢松, 胥林波 . (2008)电针丰隆、阴陵泉治疗原发性高脂血症临床疗效研究.针灸临床杂志24: 6-7.

[1141]Hu You-ping, Lu Song, Xu Lin-bo.(2008)Effect research of cupping hong leong point, Yin ling springs point on treating Primary hyperlipidemia.[JOURNAL OF CLINICAL ACUPUNCTURE AND MOXIBUSTION](http://c.g.wanfangdata.com.cn/Periodical-zjlczz.aspx) 24:6-7.

[1142] 徐维, 陈麟, 孙昱. (2008)电针配合走罐治疗脑梗塞偏瘫临床观察.针灸临床杂志24: 12-13.

## [1142]Xu Wei, Chen lin, Sun Yu.(2008)Clinical observation of electric needle combied with cupping treatment on cerebral infarction hemipleqia.[**JOURNAL OF CLINICAL ACUPUNCTURE AND MOXIBUSTION**](http://c.g.wanfangdata.com.cn/Periodical-zjlczz.aspx) 24:12-13.

[1143] 林旭明, 吴清明, 杨智慧. (2008)针刺加独活寄生汤治疗腰椎间盘突出症43例.针灸临床杂志 24: 15-16.

[1143] Xun Xu-ming, Wu Qing-ming, Yang Zhi-hui.(2008)43 case of acupuncture with parasitic warm soup on treating lumbar intervertebral disc herniation.JOURNAL OF CLINICAL ACUPUNCTURE AND MOXIBUSTION 24:15-16.

[1144] 朱德宇. (2008)醒脑开窍针刺法治疗中风病的临床观察.针灸临床杂志 24: 24.

## [1144] Zhu De-yun .(2008)Clinical observation of waking - up acupuncture treatment on stroke. [**JOURNAL OF CLINICAL ACUPUNCTURE AND MOXIBUSTION**](http://c.g.wanfangdata.com.cn/Periodical-zjlczz.aspx) 24:24

[1145] 张辉. (2008)推拿配合深刺阿是穴治疗急性腰扭伤63例.针灸临床杂志 24: 25-26.

[1145] Zhang Hui.(2008) 63 case of massage combined with deep needling Ashi points on treating acute lumbar sprain. JOURNAL OF CLINICAL ACUPUNCTURE AND MOXIBUSTION

24:25-26

[1146] 黄红缨. (2008)中药隔姜灸治疗支气管哮喘48例.针灸临床杂志 24: 33-34.

[1146] Huang Hong-ying.(2008)48 cases of traditional Chinese medicine moxibustion with ginger on treating bronchial asthma. JOURNAL OF CLINICAL ACUPUNCTURE AND MOXIBUSTION 24:33-34.

[1147]段美志, 李向荣. (2008)醒脑开窍法并夹脊刺治疗中风后偏瘫痉挛状态.针灸临床杂志 24: 7-9.

## [1147]Duan Mei-zhi, Li Xiang-rong.(2008)Clinical observation on curative effect of acupuncture therapy of waking - up combied with cliping the ridge for curing spasticity of hemiparalysis after stroke. [**JOURNAL OF CLINICAL ACUPUNCTURE AND MOXIBUSTION**](http://c.g.wanfangdata.com.cn/Periodical-zjlczz.aspx) 24:7-9.

[1148] 曹强. (2008)头电针配合吞咽功能训练治疗脑卒中后吞咽障碍60例.针灸临床杂志 24: 12-13.

[1148] Cao Qiang.(2008)60 case of scalp acupuncture in combination with deglutition training on treating swallowing disorder after stroke.JOURNAL OF CLINICAL ACUPUNCTURE AND MOXIBUSTION 24:12-13.

[1149] 姜桂美, 吴思平, 贾超. (2008)不同刺激量针刺拮抗肌与主动肌治疗脑卒中后痉挛性偏瘫的临床疗效观察.针灸临床杂志24: 1-3.

[1149]Jiang Gui-mei, Wu Si-ping, Jia Chao.(2008) Clinical efficacy observe of different stimulating amount of acupuncture antagonistic muscle and active muscle on treating spastic hemiplegia after stroke. JOURNAL OF CLINICAL ACUPUNCTURE AND MOXIBUSTION 24:1-3.

[1150] 李景君, 陈晨, 杜边军. (2008)针药结合治疗冠心病心绞痛35例.针灸临床杂志 24: 15-16.

## [1150]Li Jing-jun, Chen chen, Du bian-jun.(2008) 35 patients with angina pectoris treated with acupuncture combined with medicine. [**JOURNAL OF CLINICAL ACUPUNCTURE AND MOXIBUSTION**](http://c.g.wanfangdata.com.cn/Periodical-zjlczz.aspx) 24:15-16.

[1151] 李士杰, 王玉华, 宫丽莉. (2008)针刺配合醋离子导入、刮痧治疗神经根型颈椎病.针灸临床杂志 24: 15-16.

## [1151] Li Shi-jie, Wang Yu-hua, Gong Li-li.(2008）The effect observation of cervical spondylosis of nerve - rooted type treated with acupuncture plus vinegar ion induction and Guasha along channels. [**JOURNAL OF CLINICAL ACUPUNCTURE AND MOXIBUSTION**](http://c.g.wanfangdata.com.cn/Periodical-zjlczz.aspx) 24：15-16.

## [1152] 孙远征 , 薛建琴. (2008)针刺配合脑得生治疗血管性痴呆的临床观察.针灸临床杂志 24: 7-8.

## [1152]Sun Yuan-zheng, Xun Jian-qin.（2008)Clinical observation on acupuncture combined with Naodesheng in the treatment of vascular dementia. [**JOURNAL OF CLINICAL ACUPUNCTURE AND MOXIBUSTION**](http://c.g.wanfangdata.com.cn/Periodical-zjlczz.aspx) 24:7-8.

[1153] 赵嘉勇. (2008)腰部夹脊电针治疗腰椎间盘突出症.针灸临床杂志 24: 10-11.

[1153]Zhao Jia-rong.(2008) The waist with ridge cupping on treating Lumbar disc disease .JOURNAL OF CLINICAL ACUPUNCTURE AND MOXIBUSTION 24:10-11.

[1154] 王海峰 , 戴启斌. (2008)项针疗法治疗颈性头痛的疗效观察.针灸临床杂志 24: 1-3.

[1154]Wang Hai-feng, Dai Qi-bin.(2008) Curative effect observation of acupuncture therapy on treating cervical headache.JOURNAL OF CLINICAL ACUPUNCTURE AND MOXIBUSTION 24:1-3.

[1155] 边晓东. (2008)温针、中药熏洗对脑卒中患者步行能力的影响.针灸临床杂志 24: 13-14.

[1155] Bian Xiao-dong.(2008) The effect of acupuncture, Chinese herbal fumigation and washing to walking ability of stroke patients.JOURNAL OF CLINICAL ACUPUNCTURE AND MOXIBUSTION 24:13-14.

[1156] 陈秋明, 余伯亮 , 宾淑芬. (2008)温针灸内外膝眼穴为主治疗膝骨性关节炎.针灸临床杂志 24: 9-10.

[1156]Chen Qiu-ming, Yu Bo-liang, Bin Shu-fen.（2008） Acupuncture and moxibustion on eye acupuncture point inside and outside the knee on treating osteoarthritis of knee joint. JOURNAL OF CLINICAL ACUPUNCTURE AND MOXIBUSTION 24: 9-10.

[1157] 李卫东. (2008)蜂针结合中药外敷治疗急性痛风性关节炎疗效观察.针灸临床杂志 24: 20-21.

[1157]Li Wei-dong.(2008) Curative effect observation of bee needle combined with external application of Chinese Medicine on treating acute gouty arthritis. JOURNAL OF CLINICAL ACUPUNCTURE AND MOXIBUSTION 24: 20-21.

[1158] 郎伯旭 , 罗建昌. (2008)齐刺配合定点斜扳法为主治疗第3腰椎横突综合症120例.针灸临床杂志 24: 32-33.

[1158]Lang Bo-xu, Luo Jian-chang.(2008)120 case of triple needling combined with fixed oblique pulling method on treating the third lumbar transverse process syndrome. JOURNAL OF CLINICAL ACUPUNCTURE AND MOXIBUSTION 24:32-33.

[1159] 曹雪梅, 于海波, 刘远声. (2008)针刺任脉经穴为主治疗脑梗塞临床观察.针灸临床杂志 24: 4-5.

[1159] Cao Xue-mei, Yu Hai-bo, Liu Yuan-shen.(2008)Clinical observation of acupuncture at Ren meridian on treating cerebral infarction. JOURNAL OF CLINICAL ACUPUNCTURE AND MOXIBUSTION.24:4-5.

[1160] 李佩芳, 於先贵, 王涛. (2008)刺督调神法治疗血管性痴呆的临床研究.针灸临床杂志 24: 8-9.

[1160]Li Pei-fang, Yu Xian-gui, Wang Tao.(2008)Clinical research of thorn overseers adjustable divine on treating vascular dementia. JOURNAL OF CLINICAL ACUPUNCTURE AND MOXIBUSTION.24:8-9.

[1161] 龚燕, 朱国祥, 曾友华. (2008)平衡针刺法治疗中风后上肢高痉挛状态疗效观察.针灸临床杂志 24: 15-17.

## [1161]Gong Yan, Zhu Guo-xiang. Zeng You-hua.(2008) linical observation of recuperative effect of balancing acupuncture in treating high muscular tension of up limb in apoplectic hemiplegia. [**JOURNAL OF CLINICAL ACUPUNCTURE AND MOXIBUSTION**](http://c.g.wanfangdata.com.cn/Periodical-zjlczz.aspx) 24:15-17.

[1162] 温萍, 吴岫芙 , 刘满芬. (2008)针刺配合背部梅花针叩刺拔罐治疗乳腺增生病41例.针灸临床杂志 24: 19-20.

## [1162]Wen Ping, Wu Xiu-fu, Liu Man-fen.(2008) 41 cases of the treatments for galactophore hyperplasia by acupuncture and clubs needle therapy and cupping on the back. [**JOURNAL OF CLINICAL ACUPUNCTURE AND MOXIBUSTION**](http://c.g.wanfangdata.com.cn/Periodical-zjlczz.aspx) 24:19-20.

## [1163] 李滋平. (2008)舌针为主治疗血管性痴呆临床观察.针灸临床杂志 24: 29-30.

## [1163]Li Zi-ping.(2008)The clinical effect of tougue acupuncture for treatment of vascular dementia(VD). [**JOURNAL OF CLINICAL ACUPUNCTURE AND MOXIBUSTION**](http://c.g.wanfangdata.com.cn/Periodical-zjlczz.aspx) 24:29-30.

[1164] 李建中 , 李建华. (2008)电针配合中药治疗呃逆的临床观察.针灸临床杂志 24: 31-32.

[1164]Li Jian-zhong, Li Jian-hua.(2008) Clinical observation of electric acupuncture with Chinese medicine cupping on treating hiccups . JOURNAL OF CLINICAL ACUPUNCTURE AND MOXIBUSTION 24: 31-32.

[1165] 冯晶, 肖咏, 马廉. (2008)项针对脑卒中所致假性延髓麻痹患者血清VEGF和TGF-β1的影响.针灸临床杂志 24: 46-47.

[1165] Feng Jing, Xiao Yong, Ma Lian.(2008)Effect of nape needle on treating stroke induced pseudobulbar palsy patients serum VEGF and TGF- beta 1. JOURNAL OF CLINICAL ACUPUNCTURE AND MOXIBUSTION 24: 46-47.

[1166] 樊玲, 薛斌, 赵菁菁. (2008)近端拮抗肌群取穴法与传统取穴法对下肢痉挛性偏瘫的疗效观察.针灸临床杂志24: 6-8.

[1166]Fan Ling, Xue Bin, Zhao Jing-jing.(2008) The curative effect observation of the proximal end of the antagonistic muscles of acupoint selection method and the traditional method of acupoint on treating lower limb spastic hemiplegia. JOURNAL OF CLINICAL ACUPUNCTURE AND MOXIBUSTION 24: 6-8.

[1167] 杨晓鸿. (2008)反阿是穴治疗膝关节侧副韧带损伤疗效观察.针灸临床杂志 24: 8-10.

## [1167]Yang Xiao-hong.(2008) Observation on therapeutic effects of fan-ashi acupoint treating on kneejoint ligamenta collateralia damage.[**JOURNAL OF CLINICAL ACUPUNCTURE AND MOXIBUSTION**](http://c.g.wanfangdata.com.cn/Periodical-zjlczz.aspx) 24:8-10.

[1168] 陈美仁. (2008)电针配合刮痧治疗乳腺小叶增生30例.针灸临床杂志 24: 17-18.

## [1167]Chen Mei-ren（2008) Clinical observation on 30 accrementition of lobuli mammae patients treated by electroacupuncture and scraping. [**JOURNAL OF CLINICAL ACUPUNCTURE AND MOXIBUSTION**](http://c.g.wanfangdata.com.cn/Periodical-zjlczz.aspx) 24:17-18

[1169] 蔡耿辉, 汪流, 林秀瑶. (2008)推拿配合浅针治疗偏头痛的疗效分析.针灸临床杂志 24: 25-27.

[1169] Cai Geng-hui, Wang Liu, Lin Xiu-yao.(2008)Curative effect analysis of massage combined with shallow puncture on treating migraine. JOURNAL OF CLINICAL ACUPUNCTURE AND MOXIBUSTION 24: 25-27.

[1170] 曹辰虹, 廉玉麟. (2008)运动针法治疗中风后上肢肌肉痉挛的临床观察.针灸临床杂志 24: 33-34.

## [1170]Cao Chen-hong, Lian Yu-lin.(2008) Observations on the curative effect of motor acupuncture on upper limb spasm after stroke. [**JOURNAL OF CLINICAL ACUPUNCTURE AND MOXIBUSTION**](http://c.g.wanfangdata.com.cn/Periodical-zjlczz.aspx) 24:33-34.

[1171] 徐博佳, 孙申田. (2008)头针治疗对不同年龄组Bell麻痹患者神经功能恢复影响的临床研究.针灸临床杂志 24: 6-7.

[1171]Xu Bo-jia. Sun Shen-tian.(2008)Clinical research of scalp acupuncture on treating bell nerve paralysis patients which in different ages functional recovery effect. JOURNAL OF CLINICAL ACUPUNCTURE AND MOXIBUSTION 24: 6-7.

[1172] 余志辉, 胡建芳. (2008)早期针灸加康复治疗对急性缺血中风患者肢体功能的影响.针灸临床杂志 24: 8-11.

## [1172] Yu Zhi-hui, Hu Jian-fang.(2008) Forepart acupuncture and moxibastion with healing treatment influencing body function of acute ischemia stroke patients. [**JOURNAL OF CLINICAL ACUPUNCTURE AND MOXIBUSTION**](http://c.g.wanfangdata.com.cn/Periodical-zjlczz.aspx) 24:8-11.

[1173] 唐强, 陈慧杰, 李雪静. (2009)头针结合高电位治疗脑卒中后肩痛的临床观察.针灸临床杂志 25: 19-20.

## [1173]Tang Qiang, Chen Hui-jie, Li Xue-jing.(2009) Clinical observation of scalp acupuncture combination of high - potential treatment on post - stroke shoulder pain. [**JOURNAL OF CLINICAL ACUPUNCTURE AND MOXIBUSTION**](http://c.g.wanfangdata.com.cn/Periodical-zjlczz.aspx)25:19-20.

[1174] 李岩, 刘睿, 王颖 . (2009)调神理气针刺法治疗中风后抑郁症36例[J].针灸临床杂志 25: 22-23.

## [1174] Li Yan. Liu Rui. Wang Ying.(2009)Opsonic needling therapy treating 36 cases of depression after stroke. [**JOURNAL OF CLINICAL ACUPUNCTURE AND MOXIBUSTION**](http://c.g.wanfangdata.com.cn/Periodical-zjlczz.aspx) 25;22-23.

[1175] 陈建军. (2009)电针加刺络拔罐治疗肩周炎疗效观察.针灸临床杂志25: 27-28.

## [1175]Chen Jian-jun.(2009). Therepeutie effect observation on electro - acupuncture, venesection and ventouse treatment on shoulder pefiarthritis. [**JOURNAL OF CLINICAL ACUPUNCTURE AND MOXIBUSTION**](http://c.g.wanfangdata.com.cn/Periodical-zjlczz.aspx) 25:27-28.

[1176] 张宁, 高敏. (2009)电体针与传统针刺对卒中后足下垂的疗效对比观察.针灸临床杂志 25: 29-30.

## [1176] Zhang Ning, Gao Min.(2009)Efficacy comparison observation of electro- needle and traditional needle treating foot drop after apoplexy.[**JOURNAL OF CLINICAL ACUPUNCTURE AND MOXIBUSTION**](http://c.g.wanfangdata.com.cn/Periodical-zjlczz.aspx) 25:29-30.

[1177] 谢菊英, 王灵. (2009)电针华佗夹脊穴治疗小儿脑瘫肌痉挛的临床观察.针灸临床杂志 25: 31-32.

## [1177] Xie Ju-ying, Wang Ling.(2009) Clinical observation of electro- acupuncture Huatuo Jiaji points treatment with cerebral palsy myospasm. [**JOURNAL OF CLINICAL ACUPUNCTURE AND MOXIBUSTION**](http://c.g.wanfangdata.com.cn/Periodical-zjlczz.aspx) 25:31-32.

[1178] 迟旭, 鞠琰莉. (2009)经皮神经电刺激治疗慢传输型便秘的疗效观察.针灸临床杂志 25: 32-33.

## [1178]Chi Xu, Ju Yan-li.(2009) Observation on clinical therapeutic effect of transcutaneous electric nerve stimulation in slow transit constipation. JOURNAL OF CLINICAL ACUPUNCTURE AND MOXIBUSTION 25-32-33.

[1179] 李鹏飞. (2009)深刺腰夹脊穴配合骶管注射治疗腰椎间盘突出症的临床体会.针灸临床杂志 25: 20-21.

[1179]Li Peng-fei.(2009) Clinical experience of Jiaji points combined with deep needling lumbar sacral canal injection on treating lumbar intervertebral disc herniation. JOURNAL OF CLINICAL ACUPUNCTURE AND MOXIBUSTION 25: 20-21.

[1180] 宋维健. (2009)巨刺与刺络放血法治疗脑卒中后丘脑痛29例.针灸临床杂志 25: 34-35.

[1180] Song Wei-jian.(2009) 29 case of great acupuncture and pricking blood therapy method on treating thalamic pain after stroke.JOURNAL OF CLINICAL ACUPUNCTURE AND MOXIBUSTION 25: 34-35.

[1181] 欧阳颀, 魏智钧, 张春梅. (2009)分期动静针刺对脑卒中早期康复的增强作用[J].针灸临床杂志 25: 3-5.

## [1181] Ou Yang-qi, Wei Zhi-jun, Zhang Chun-mei.(2009)Effects of Staging Dynamic-static Acupuncture on Acute Stroke Patients Received Early Rehabilitation. [**JOURNAL OF CLINICAL ACUPUNCTURE AND MOXIBUSTION**](http://c.g.wanfangdata.com.cn/Periodical-zjlczz.aspx) 25:3-5

[1182] 曲齐生, 于相丽, 逄金彩. (2009)针刺、穴位贴敷配合药物治疗单纯型慢性支气管炎.针灸临床杂志 25: 8-9.

## [1182] Qu Qi-sheng, Yu Xiang-li, Pang Jin-cai.(2009) Effect Observation on Treating 40 cases with Simple Chronic Bronchitis by Acupun cture, Acupoint Application Compounded with Levofloxacin.[**JOURNAL OF CLINICAL ACUPUNCTURE AND MOXIBUSTION**](http://c.g.wanfangdata.com.cn/Periodical-zjlczz.aspx) 25:8-9

[1183] 朱建红. (2009)针刺、TDP理疗结合拔罐治疗慢性盆腔炎42例.针灸临床杂志 25: 12-13.

[1183] Zhu Jian-hong.(2009) Chronic Pelvic Inflammatory Disease Treated by Acupuncture, TDP Combined with Cupping. JOURNAL OF CLINICAL ACUPUNCTURE AND MOXIBUSTION 25: 12-13.

[1184] 赵鑫, 唐强, 王艳. (2009)综合治疗对脑梗死患者下肢功能的影响.针灸临床杂志 25: 1-3.

[1184] Zhao Xin, Tang Qiang, Wang Yan.(2009)Effect of comprehensive treatment on lower limb function in patients with cerebral infarction. JOURNAL OF CLINICAL ACUPUNCTURE AND MOXIBUSTION 25: 1-3.

[1185] 衣华强. (2009)头皮针结合体针治疗神经根型颈椎病30例.针灸临床杂志 25: 18-19.

[1185]Yi Hua-qiang.(2009) Nerve Root Type Cervical Spondylosis 30 Cases Treated by Scalp Acupuncture Combined with Body Acupuncture.JOURNAL OF CLINICAL ACUPUNCTURE AND MOXIBUSTION 25: 18-19.

[1186] 赵树英, 刘亚平. (2009)针药合用治疗脂肪肝30例.针灸临床杂志 25: 22-23.

[1186] Zhao Shu-ying, Liu Ya-ping.(2009) 30 case on the combined use of acupuncture and medicine on treating fatty liver. JOURNAL OF CLINICAL ACUPUNCTURE AND MOXIBUSTION 25: 22-23

[1187] 刘丽莉. (2009)针刺对冠心病合并抑郁症心电图影响的研究.针灸临床杂志 25: 23-24.

[1187] Liu Li-li.(2009) Reseach of acupuncture to effect of ECG in Coronary Heart Disease Complicating Depression. JOURNAL OF CLINICAL ACUPUNCTURE AND MOXIBUSTION 25: 23-24.

[1188] 王勇. (2009)温针灸结合头针治疗膝骨性关节炎疗效观察.针灸临床杂志 25: 34-35.

[1188]Wang Yong.(2009) The curative effect observation of warm acupuncture and scalp acupuncture on treating osteoarthritis of knee joint. JOURNAL OF CLINICAL ACUPUNCTURE AND MOXIBUSTION 25: 34-35.

[1189] 李滋平, 闫晓燕. (2009)穴位埋线为主治疗高血压病的临床研究.针灸临床杂志 25: 3-5.

[1189] Li Zi-ping, Yan Xiao-yan, (2009) Clinical Effect of Catgut-embedding into the Points for Treatment of Essential Hypertension Electroacupuncturing.JOURNAL OF CLINICAL ACUPUNCTURE AND MOXIBUSTION 25: 3-5.

[1190] 梅晨健, 马莉, 徐惠荣. (2009)针刺夹脊穴治疗帕金森病的临床疗效观察[J].针灸临床杂志 25: 13-14.

## [1190]Yang, Chen-jian, Ma li, Xu Hui-rong.(2009)Clinical Observation on Parkinson Treated by Acupuncturing on Jiaji Piont. [**JOURNAL OF CLINICAL ACUPUNCTURE AND MOXIBUSTION**](http://c.g.wanfangdata.com.cn/Periodical-zjlczz.aspx) 25: 13-14.

[1191] 颜慧, 杨玉光. (2009)针刺、按摩配合舒适护理用于镇痛、催产的临床观察.针灸临床杂志25: 21-22.

[1191]Yan Hui, Yang Yu-guang.(2009) Clinical observation of acupuncture, massage with comfortable nursing on analgesia, oxytocin. JOURNAL OF CLINICAL ACUPUNCTURE AND MOXIBUSTION 25: 21-22.

[1192] 黄乃好, 马洪辉, 郑春雪. (2009)电针配合TDP照射治疗肩周炎52例.针灸临床杂志 25: 29-30.

[1192]Huang Nai-hao, Ma Hong-hui, Zheng Chun-xue.(2009)Scapulohumeral Periarthritis 52 Cases Treated by Electro-acupuncture Combined with TDP Irradiation. JOURNAL OF CLINICAL ACUPUNCTURE AND MOXIBUSTION 25: 29-30.

[1193] 叶志英. (2009)膏肓穴七星针扣刺加拔罐综合疗法治疗乳痈.针灸临床杂志 25: 31.

[1193]Ye Zhi-ying.(2009)Gaohuang acupuncture point seven star needle buckle needling and cupping combined therapy on treating acute mastitis.JOURNAL OF CLINICAL ACUPUNCTURE AND MOXIBUSTION 25: 31.

[1194] 谈天明. (2009)齐刺肌肉起止点治疗梨状肌综合症36例.针灸临床杂志25: 34-35.

[1194]Tan Tian-ming.(2009) 36 case of muscle enthesitis tripleneedling on treating piriformis syndrome. JOURNAL OF CLINICAL ACUPUNCTURE AND MOXIBUSTION 25: 34-35.

[1195] 吕士琦. (2009)平推加温和灸预防慢性支气管炎复发100例.针灸临床杂志 25: 11-13.

[1195]Lv Shi-qi.(2009) 100 case of pushing and warming moxibustion on preventing recurrence of chronic bronchitis. JOURNAL OF CLINICAL ACUPUNCTURE AND MOXIBUSTION 25: 11-13.

[1196] 葛成慧, 忽浩杰, 朱广旗. (2009)针刺督脉穴为主对急性脑梗死患者血清CRP的影响.针灸临床杂志 25: 1-3.

[1196]Ge Chen-hui, Hu Hao-jie, Zhu Guang-qi.(2009）Effection of acupuncture du meridian acupuncture point on levels of serum CRP in patients with acute cerebral infarction . JOURNAL OF CLINICAL ACUPUNCTURE AND MOXIBUSTION 25: 1-3.

[1197] 王雪峰, 李艳 , 戴文晋. (2009)针刺配合中频治疗仪治疗脑血管意外后尿失禁67例.针灸临床杂志 25: 15-16.

[1197]Wang Xue-feng, Li Yan, Dai Wen-jin.(2009)Effect of Acupuncturing Du Meridian as A Main Therapy on Serum C-reaction protein in the Patient of Acute Cerebral Infarction. JOURNAL OF CLINICAL ACUPUNCTURE AND MOXIBUSTION 25: 15-16.

[1198] 梁树艺. (2009)电针头部腧穴治疗失眠症疗效观察.针灸临床杂志 25: 24-25.

[1198] Liang Shu-yi.(2009) The curative effect observation of the head acupoints electroacupuncture on treating insomnia. JOURNAL OF CLINICAL ACUPUNCTURE AND MOXIBUSTION 25: 24-25.

[1199] 王野, 邹伟久. (2009)久留针强捻转治疗原发性三叉神经痛.针灸临床杂志 25: 28-29.

[1199]Wang Ye, Zou Wei-jiu.(2009) Stay strong twirling needle on treating primary trigeminal neuralgia. JOURNAL OF CLINICAL ACUPUNCTURE AND MOXIBUSTION 25: 28-29.

[1200] 聂斌, 徐凯, 杨顺益. (2009)通阳醒脑针刺法治疗中风恢复期的临床研究.针灸临床杂志 25: 4-5.

[1200] Nie Bin, Xu Kuai, Yang Shun-yi.(2009)Clinical Research of Apoplexy Convalescence Treatment by Tongyangxingnao Acupuncturing 25: 4-5.

[1201] 胡智海, 王毅, 沈鸿斌. (2009)针刺气海”与三阴交对胰岛素抵抗型多囊卵巢综合征内分泌调整作用的对比研究.针灸临床杂志 25: 1-2.

[1201]Hu Zhi-hai, Wang Yi, Shen Hong-bin.(2009)Study on Impact of Inserting Qihai and Sanyinjiao Point on the Endocrine Changes in Insulin Resisting PCOS .JOURNAL OF CLINICAL ACUPUNCTURE AND MOXIBUSTION 25: 1-2.

[1202] 宋云杰, 侯宝昌, 史云鹤, 刘鹏. (2009)针刺结合穴位注射治疗面神经炎临床观察.针灸临床杂志 25: 13-14.

[1202]Song Yun-jie, Hou Bao-chang, Shi Yun-he.(2009)Clinical Observation on Treating Facial Paralysis by Acupuncture Combined with Acupoint Injection. JOURNAL OF CLINICAL ACUPUNCTURE AND MOXIBUSTION 25: 13-14.

[1203] 杜梦玄. (2009)益气扶正针灸法治疗中风后遗症50例.针灸临床杂志25: 17-18.

[1203]Du Meng-xuan.(2009)Treating Sequelae of Stroke 50 Cases by Yiqifuzheng Acupuncture. [JOURNAL OF CLINICAL ACUPUNCTURE AND MOXIBUSTION](http://c.g.wanfangdata.com.cn/Periodical-zjlczz.aspx) 25: 17-18.

[1204] 倪姗姗, 高旭超, 刘立安. (2009)电针治疗面瘫伴发糖尿病的随机对照研究.针灸临床杂志 25: 27-28.

[1204]Ni Shan-shan, Gao Xu-chao, Liu Li-an.(2009)Randomized Controlled Trial of Comprehensive Treatment of Facial Paralysis with Diabetes Mellitus with Electroacupuncture Chiefly. [JOURNAL OF CLINICAL ACUPUNCTURE AND MOXIBUSTION](http://c.g.wanfangdata.com.cn/Periodical-zjlczz.aspx) 25: 27-28.

[1205] 陈洪沛, 陈佳, 杨运宽, 路永红 向丹黎. (2009)不同针刺方法治疗带状疱疹后遗神经痛的临床观察.针灸临床杂志 25: 31-33.

[1205]Chen Hong-pei, Chen jia, Yang Yun-kuan, Lu Yong-hong, Xiang Dan-li.(2009)Clinical observation of different methods of acupuncture on treating postherpetic neuralgia.[JOURNAL OF CLINICAL ACUPUNCTURE AND MOXIBUSTION](http://c.g.wanfangdata.com.cn/Periodical-zjlczz.aspx) 25: 31-33.

[1206] 谭小华, 刘海涛, 王锂艳, 韦东. (2009)药线点灸配合围刺通电治疗带状疱疹后遗神经痛30例.针灸临床杂志 25: 33-34.

[1206] Tan Xiao-hua, Liu Hai-tao, Wang Li-yang, Wei dong.(2009)30 case of medicated thread moxibustion combined with surround needling electricity on treating Postherpetic neuralgia. [JOURNAL OF CLINICAL ACUPUNCTURE AND MOXIBUSTION](http://c.g.wanfangdata.com.cn/Periodical-zjlczz.aspx) 25: 33-34.

[1207] 刘伟华, 赵嫦莹, 伦新, 余瑾. (2009)眠三针”治疗抑郁障碍相关性失眠30例.针灸临床杂志 25: 5-6.

[1207] Liu Wei-hua, Zhao Chang-ying, Lun Xin, Yu Jin.(2009)Clinical Research on Depression-related Insomnia Treating with Sleep 3-needle Point. [JOURNAL OF CLINICAL ACUPUNCTURE AND MOXIBUSTION](http://c.g.wanfangdata.com.cn/Periodical-zjlczz.aspx) 25: 5-6.

[1208] 庄珣, 陆彦青, 庄礼兴. (2009)火针腰夹脊穴为主治疗腰椎间盘突出症30例.针灸临床杂志 25: 1-3.

## [1208]Zhuang Xun, Lu Yan-qing, Zhuang Li-xing.(2009)The Clinical Observation on Treatment of Fire-needling Lumbar Jiaji Points in Patients with Lumbar Intervertebral Disc protrusion. [**JOURNAL OF CLINICAL ACUPUNCTURE AND MOXIBUSTION**](http://c.g.wanfangdata.com.cn/Periodical-zjlczz.aspx). 25: 1-3.

[1209] 范景海, 蒋丽萍, 李云平, 岳桂贤 张晓丽. (2009)针刺配合音乐疗法治疗偏头痛临床观察.针灸临床杂志25: 14.

[1209]Fang Jin-hai, Jiang Li-ping, Li Yun-ping, Yue Gui-xian, Zhang Xiao-li.(2009)Clinical observation of acupuncture combined with music therapy on treating migraine.JOURNAL OF CLINICAL ACUPUNCTURE AND MOXIBUSTION 25: 14.

[1210] 罗仁瀚, 陈秀玲 , 徐凯雷. (2009)雷火灸治疗单纯性肥胖症30例.针灸临床杂志 25: 30-32.

[1210]Luo Ren-han, Chen Xiu-ling, Xu Kai-lei.(2009)Treating Simple Obesity 30 Cases by Leihuo Moxibustion. [JOURNAL OF CLINICAL ACUPUNCTURE AND MOXIBUSTION](http://c.g.wanfangdata.com.cn/Periodical-zjlczz.aspx) 25: 30-32.

[1211] 姜京明, 廖恒, 陈秘密, 叶禹 杨武. (2009)针刺加穴位注射治疗神经根型颈椎病的临床疗效观察.针灸临床杂志 25: 6-7.

[1211]Jiang Jin-ming, Liao He, Chen Mi-mi, Ye Yu. Yang Wu.(2009) Clinical Study on Nerve-root Cervical Spondylosis with Acupuncture and Acupoint Injection Therapy. [JOURNAL OF CLINICAL ACUPUNCTURE AND MOXIBUSTION](http://c.g.wanfangdata.com.cn/Periodical-zjlczz.aspx) 25: 6-7.

[1212] 孙国栋, 师彬, 吴清波. (2009)三维针刀”治疗神经根型颈椎病的临床研究.针灸临床杂志 25: 10-12.

[1212]Sun Guo-dong, Shi Bin, Wu Qing-bo.(2009)Clinical Research on Never Root Kind of Cervical Spondylosis Cured by Three-dimensional Needle-knife. [JOURNAL OF CLINICAL ACUPUNCTURE AND MOXIBUSTION](http://c.g.wanfangdata.com.cn/Periodical-zjlczz.aspx) 25: 10-12.

[1213] 杜健民, 黄泳 , 陈俊琦. (2009)传统薄氏腹针配合大面积电磁波辐射治疗神经根型颈椎病.针灸临床杂志 25: 1-3.

[1213]Du Jian-ming, Huang Yong, Chen Jun-qi.(2009) Treating Nerve Root Cervical Spondylosis by Bo's Abdominal Acupuncture Combined with Large-scale Electromagnetic Radi-ation.[JOURNAL OF CLINICAL ACUPUNCTURE AND MOXIBUSTION](http://c.g.wanfangdata.com.cn/Periodical-zjlczz.aspx) 25: 1-3.

[1214] 宋云杰, 史云鹤, 侯宝昌, 刘鹏. (2009)针药结合治疗更年期不寐的临床观察.针灸临床杂志 25: 21-22.

[1214] Song Yun-jie, Shi Yun-he, Hou Bao-chan, Liu peng.(2009）Clinical observation of combination of acupuncture and medicine on treating menopausal insomnia. JOURNAL OF CLINICAL ACUPUNCTURE AND MOXIBUSTION 25: 21-22.

[1215] 王伟华, 杨沈秋, 李晶. (2009)头穴透刺结合康复技术对脑卒中运动性失语语言功能的影响.针灸临床杂志 25: 30-31.

[1215]Wang Wei-hua, Yang Shen-qiu, Li Jin.(2009)Effects of Joined Puncture on Head Points Combined with Rehabilitation Technique on Motor Aphasia. [JOURNAL OF CLINICAL ACUPUNCTURE AND MOXIBUSTION](http://c.g.wanfangdata.com.cn/Periodical-zjlczz.aspx) 25: 30-31.

[1216] 武平, 梁繁荣, 杨玲, 罗伦，李安宏，等. (2009)针刺对脑卒中后偏瘫肢体痉挛患者神经功能缺损的影响.针灸临床杂志 25: 8-10.

[1216]Wu Ping, Liang Fang-rong, Yang Ling, Luo-Lun, Li An-hong, et al.(2009) Effect of Acupuncture for Neurological Functional Deficit in the Spasticity Patients after Stroke. [JOURNAL OF CLINICAL ACUPUNCTURE AND MOXIBUSTION](http://c.g.wanfangdata.com.cn/Periodical-zjlczz.aspx) 25: 8-10.

[1217] 李子勇, 卢佩斯. (2009)针刺加穴位埋线治疗单纯性肥胖症的临床对照研究.针灸临床杂志25: 1-3.

[1217] Li Zi-yong, Lu Pei-si.(2009) Clinical Comparative Study on Acupuncture Combined with Acupoint Catgut Embedding for Treatment of Simple Obesity. [JOURNAL OF CLINICAL ACUPUNCTURE AND MOXIBUSTION](http://c.g.wanfangdata.com.cn/Periodical-zjlczz.aspx) 25: 1-3.

[1218] 何泽多, 谭武, 陈志斌. (2009)推拿结合夹脊电针治疗腰椎间盘突出症对照观察.针灸临床杂志 25: 21-23.

[1218]He Zhe-duo, Tan Wu, Chen Zhi-bin.(2009)Control observation of combined with massage Jiaji electropuncture on treating lumbar intervertebral disc herniation. JOURNAL OF CLINICAL ACUPUNCTURE AND MOXIBUSTION 25: 21-23.

[1219] 李湘力, 蔡敬宙, 江钢辉. (2009)舌三针治疗中风失语症30例.针灸临床杂志 25: 6-8.

[1219] Li Xiang-li, Cai Jin-yu, Jiang Gang-hui.(2009)Observation on Clinical Effect of Tongue Triple Acupuncture in the Treatment of Aphasia after Stroke. [JOURNAL OF CLINICAL ACUPUNCTURE AND MOXIBUSTION](http://c.g.wanfangdata.com.cn/Periodical-zjlczz.aspx) 25: 6-8.

[1220] 白桦, 于澎. (2009)电针治疗干燥综合征的临床观察及对免疫功能影响的研究.针灸临床杂志25: 9-11.

[1220]Bai Hua, Yu Peng.(2009) The Study of Electroacupuncture on the Clinical Observation and the Influence of the Immunity Function. [JOURNAL OF CLINICAL ACUPUNCTURE AND MOXIBUSTION](http://c.g.wanfangdata.com.cn/Periodical-zjlczz.aspx) 25: 9-11.

[1221] 刘兴勤. (2009)温针灸为主治疗周围性面瘫50例.针灸临床杂志 25: 10.

[1221]Liu Xin-qin.(2009)Treating Peripheral Facial Paralysis 50 Cases by Needle Warming Moxibustion. [JOURNAL OF CLINICAL ACUPUNCTURE AND MOXIBUSTION](http://c.g.wanfangdata.com.cn/Periodical-zjlczz.aspx) 25: 10.

[1222] 孙健, 贾真, 董嘉怡, 谢长才，徐振华，等. (2009)腹针配合艾灸治疗腰椎间盘突出症临床研究.针灸临床杂志25: 1-2.

[1222] Sun Jian, Jia Zhen, Dong Jia-yi, Xie Chang-cai, Xu Zhen-hua, et al.(2009)Clinical Study on Treating Lumbar Interveterbral Disc Protrusion by Abdomen Needle Combined with Moxibustion. [JOURNAL OF CLINICAL ACUPUNCTURE AND MOXIBUSTION](http://c.g.wanfangdata.com.cn/Periodical-zjlczz.aspx) 25: 1-2.

[1223] 史术峰. (2009)项针结合吞咽康复训练治疗脑卒中后吞咽困难.针灸临床杂志25: 18-19.

[1223] Shi Shu-feng.（2009）Nape needle combined with rehabilitation training on treating dysphagia after stroke. JOURNAL OF CLINICAL ACUPUNCTURE AND MOXIBUSTION 25: 18-19.

[1224] 陈佐龙, 韩春, 王广武. (2009)电针治疗膀胱过度活动症24例.针灸临床杂志 25: 22.

[1224]Chen Zuo-long, Han Chun, Wang Guang-wu.(2009) 24 case of electroacupuncture on treating overactive bladder. JOURNAL OF CLINICAL ACUPUNCTURE AND MOXIBUSTION 25: 22.

[1225] 王晶, 万志杰. (2009)梅花针联合肤疾宁治疗慢性湿疹疗效观察.针灸临床杂志 25: 23-24.

[1225] Wang Jin, Wang Zhi-jie.(2009)Clinical Curative Effect of the Dermal Needles and Triamcinolone Acetonide and Neomycin Paste on Chronic Eczema. [JOURNAL OF CLINICAL ACUPUNCTURE AND MOXIBUSTION](http://c.g.wanfangdata.com.cn/Periodical-zjlczz.aspx) 25: 23-24.

[1226] 赵桂君, 李岩, 陈伊, 王玲姝. (2009)头穴透刺治疗急性脑出血患者的体感诱发电位观察.针灸临床杂志 25: 26-27.

[1226]Zhao Gui-jun , Li Yan, Chen Yi, Wang Ling-zhu.(2009) Observation on somatosensory evoked potential of The scalp point penetration on treating In patients with acute cerebral hemorrhage. JOURNAL OF CLINICAL ACUPUNCTURE AND MOXIBUSTION 25: 26-27.

[1227] 陈啸峰. (2009)电针治疗单纯性肥胖及对血清瘦素和胰岛素影响的研究.针灸临床杂志 25: 3-5.

[1227] Chen Xiao-feng.(2009) Study on serum leptin and insulin influence of electroacupuncture on treating simple obesity. JOURNAL OF CLINICAL ACUPUNCTURE AND MOXIBUSTION 25: 3-5.

[1228] 刘晓磊, 徐凤鸣. (2009)针刺配合康复治疗偏瘫平衡功能障碍40例.针灸临床杂志 25: 8-9.

[1228] Liu Xiao-lei, Xu Fen-ming.(2009) 40 case of acupuncture combined with rehabilitation on treating the balance function of hemiplegia. JOURNAL OF CLINICAL ACUPUNCTURE AND MOXIBUSTION 25: 8-9.

[1229] 旷甫国 , 陈利华. (2009)温针灸结合药物治疗腰椎间盘突出症术后腰痛.针灸临床杂志 25: 39-40.

[1229] Kuang Pu-guo, Chen Li-hua.(2009) Treating Lumber-back Pain of after Operation for Prolapse of Lumber Intervertebral Disc by Warm Needle Moxibustion Combined with Chinese herbs. [JOURNAL OF CLINICAL ACUPUNCTURE AND MOXIBUSTION](http://c.g.wanfangdata.com.cn/Periodical-zjlczz.aspx) 25: 39-40.

[1230] 常国良. (2010)不同针灸方法治疗慢性腹泻的疗效分析.针灸临床杂志 : 12-13.

[1230]Chang Guo-liang.(2010)Curative effect analysis of different acupuncture methods on treating chronic diarrhea.JOURNAL OF CLINICAL ACUPUNCTURE AND MOXIBUSTION:12-13

[1231] 陈少宗, 卜彦青, 翟华普, 杨凯，高树中. (2010)针刺单穴、多穴对原发性痛经患者即时止痛作用规律的初步观察.针灸临床杂志 1: 1-3.

[1231]Chen Shao-zong, Bu Yan-qing, Zhai Hua-pu, Yang Kai, Gao Shu-zhong.(2010)Comparison on Immediate Analgesic Effect in Patients with Primary Dysmenorrhea between Acupuncture of Single Point and Several Points. [JOURNAL OF CLINICAL ACUPUNCTURE AND MOXIBUSTION](http://c.g.wanfangdata.com.cn/Periodical-zjlczz.aspx) 1: 1-3.

[1232] 周忠亮. (2010)少阳三针治疗颈性眩晕疗效观察.针灸临床杂志 26: 24-25.

## [1232]Zhou Zhong-liang.(2010) Clinical Observation on Treating Cervical Vertigo by Shaoyangsanzhen. [**JOURNAL OF CLINICAL ACUPUNCTURE AND MOXIBUSTION**](http://c.g.wanfangdata.com.cn/Periodical-zjlczz.aspx) 26: 24-25.

[1233] 吴昊, 李芳莉. (2010)磁极针治疗Ⅱ型糖尿病末梢神经病变30例.针灸临床杂志 1: 25-26.

[1233] Wu Hao, Li Fang-li.(2010)30 case on magnetic needle on treating Type II diabetes peripheral neuropathy. JOURNAL OF CLINICAL ACUPUNCTURE AND MOXIBUSTION 1: 25-26.

[1234] 吴长岩, 贾乐红, 吕志军. (2010)温针灸对冠心病心绞痛血脂、血流变的影响.针灸临床杂志 1: 36-38.

[1234] Wu Chang-Yan, Jia Le-Hong, Lv Zhi-jun.(2010)Influence of acupuncture and moxibustion on treating angina pectoris of coronary heart disease of blood lipid, blood rheology. JOURNAL OF CLINICAL ACUPUNCTURE AND MOXIBUSTION 1: 36-38.

[1235] 李巍, 谭洛, 苗林艳, 李普海, 陈建. (2010)电针肺俞穴对支气管哮喘患者(急性发作期):临床症状与肺功能的影响.针灸临床杂志 26:4-8.

[1235] Li Wei, Tan Luo, Miao Lin-yan, Li Pu-hai, Chen Jian.(2010)Effect of Electro-acupuncture of BL13 on Symptoms and Pulmonary Function in Asthma. [JOURNAL OF CLINICAL ACUPUNCTURE AND MOXIBUSTION](http://c.g.wanfangdata.com.cn/Periodical-zjlczz.aspx) 26:4-8.

[1236] 卫彦, 寇吉友, 陈军针. (2010)刺人迎穴对高血压患者血清中血管活性物质影响的研究.针灸临床杂志 2: 12-14.

[1236]Wai Liao, Ko Ji-you, Chen Jun-zhen.(2010)Study on Vaso-active Substance in Serum of Essential Hypertensive by the Treatment of Acupuncturing Renying Point. [JOURNAL OF CLINICAL ACUPUNCTURE AND MOXIBUSTION](http://c.g.wanfangdata.com.cn/Periodical-zjlczz.aspx) 2: 12-14.

[1237] 王琳, 吴艺玲, 曲本琦. (2010)不同穴位组合治疗子宫切除术后尿潴留的疗效观察.针灸临床杂志 2:15-16.

[1237]Wang Lin, Wu Yi-ling, Qu Ben-qi.(2010)Clinical Observation of Acupuncture with Different Points Combination on Post-hysterectomy Uroschesis. [JOURNAL OF CLINICAL ACUPUNCTURE AND MOXIBUSTION](http://c.g.wanfangdata.com.cn/Periodical-zjlczz.aspx) 2:15-16.

[1238] 阮志忠, 陆瑾, 李静. (2010)针刺配合拿、捏风池穴治疗失眠症36例.针灸临床杂志 2: 19-20.

[1238]Ruan Zhi-zhong, Lu Jin, Li-jing.(2010)Acupuncture combined with Helena, pinch Fengchi treatment of insomnia 36 cases.JOURNAL OF CLINICAL ACUPUNCTURE AND MOXIBUSTION.2: 19-20.

[1239] 於芸. (2010)刃针治疗颈源性头痛的疗效观察.针灸临床杂志 .2: 32-33.

[1239]Yu Yun.(2010) Effect observation of cervical headache treated with Edge Needles.JOURNAL OF CLINICAL ACUPUNCTURE AND MOXIBUSTION. 2: 32-33.

[1240] 秦玉革. (2010)温针脊神经为主治疗腰椎间盘突出症临床观察.针灸临床杂志2: 35-37

[1240] Qin Yu-ge.(2010）Observation on the Therapeutic Effect of Warming needle Moxibustion Nervus Spinalis on Prolapse of Lumbar Intervertebral Disc. [JOURNAL OF CLINICAL ACUPUNCTURE AND MOXIBUSTION](http://c.g.wanfangdata.com.cn/Periodical-zjlczz.aspx) 2: 35-37

[1241] 韩淑凯, 张宝昌, 左永发, 董进友,马会军. (2010)表里两经并刺法治疗脑卒中后上肢痉挛56例.针灸临床杂志.26: 37-40.

[1241] Han Shu-kai, Zhang Bao-chang, Zuo Yong-fa, Dong Jin-you, Ma Hui-jun.(2010) acupuncture therapy of exteriorinterior related meridian points on upper limb spasticity in the patient of poststroke:56 cases observation. Journal of Clinical Acupuncture and Moxibustion.

26:37-40.

[1242] 宿健,于学平. (2010)针刺治疗感音神经性耳聋.针灸临床杂志 26:40-41.

[1242] Su Jian, Yu Xue-ping.(2010)Acupuncture for sensorineural hearing loss. Journal of Clinical Acupuncture and Moxibustion.26:40-41.

[1243] 邹雅琴, 吴思平, 刘映芬. (2010)针刺配合激光治疗腰椎间盘突出症的临床观察.针灸临床杂志 26: 13-16.

[1243] Zou Ya-Qin, Wu Si-ping, Liu Ying-fang.(2010)Clinical Observation on the Therapeutic Effect of Acupuncture and Superlizer Therapy for Lumbar Disc Herniation. Journal of Clinical Acupuncture and Moxibustion.26:13-16.

[1244] 李虹霖 ,孙远征. (2010)头穴透刺对运动性疲劳影响的临床研究.针灸临床杂志26:16-18.

[1244] Li Hong-lin, Sun Yuan-zheng.(2010)Clinical Research on the Effect of Scalp Penetration Acupuncture Treating Exercise- induced Fatigue. Journal of Clinical Acupuncture and Moxibustion.26:16-18.

[1245] 张茜, 张伟, 孙媛. (2010)笑气吸入与电针刺激在无痛分娩中的临床对比观察.针灸临床杂志 26: 23-25.

[1245] Zhang Xi, Zhang Wei, Sun Yuan .(2010)A compare study on the clinical efect of ease pain using transcutaneous electrical acupoint stimulation (TEAS) with Nitrous oxide (N2O) inhalation during indolence delivery. Journal of Clinical Acupuncture and Moxibustion.26:23-25.

[1246] 谢红亮, 陈尚杰, 曹雪梅,黄石钊,刘永锋. (2010)隔蒜灸配合穴位注射治疗过敏性鼻炎.针灸临床杂志26: 26-27.

## [1246] Xie Hong-liang, Chen Shang-jie, Cao xue-mei, Huang Shi-zhao, Liu Yong-feng(2010). Clinical Study on Treatment of Allergic Rhinitis by Indirect Garlic Moxibustion Coupled with Point Injection. Journal of Clinical Acupuncture and Moxibustion.26:26-27.

[1247] 宋鹏. (2010)局部围刺结合背俞穴治疗黄褐斑临床观察.针灸临床杂志 4: 29-30.

[1247] Song Peng. (2010)Clinical Observation of Sround Acupuncture with Back-shu Points on treatment of chloasma. Journal of Clinical Acupuncture and Moxibustion.4:29-30.

[1248] 汪泽栋, 杨炳同，杨续艳.(2010)电针治疗慢性功能性腹泻的临床疗效观察.针灸临床杂志 26: 33-35.

[1248] Wang Ze-dong, Yang Bing-tong, Yang Xu-yan.(2010)Observation on Chronic Functional Diarrhea Treated by Electro- acupuncture. Journal of Clinical Acupuncture and Moxibustion.

26:33-35.

[1249] 刘双岭, 陈伊, 姜海霞,高维滨. (2010)夹脊电针治疗神经根型颈椎病30例.针灸临床杂志. 26: 39-40.

[1249] Liu Shuang-ling , Chen Yi, Jiang Hai-xia, Gao Wei-bin. (2010)Jia ji electropuncturection for radiculo-spondylopathy:30 cases observation. Journal of Clinical Acupuncture and Moxibustion. 26:39-40.

[1250] 顾勤. (2010)针药结合治疗复发性口腔溃疡疗效观察.针灸临床杂志 5: 28-29.

[1250] Gu Qin. (2010)Clinical effect of Acupuncture Combined with Medicine in the treatment of recurrent oral ulcer.Journal of Clinical Acupuncture and Moxibustion. 5:28-29.

[1251] 曹国元, 杨金龙, 杨青宇, [陈铮华](http://www.cnki.net/KCMS/detail/search.aspx?dbcode=CJFQ&sfield=au&skey=陈铮华&code=15785918;24437368;24357237;24437369;). (2010)激能电摩疗法治疗面瘫经验.针灸临床杂志 5: 56-57.

[1251] Cao Guo-yuan, Yang Jin-ling, Yang Qing-yu,Chen Zheng-hua.(2010)the clinical experience of electrical stimulation for [facioplegia](../../../../D:/Dict/5.4.43.3217/resultui/app:ds:facioplegia).Journal of Clinical Acupuncture and Moxibustion. 5: 56-57.

[1252] 叶成鹄, 姜翼. (1989)艾灸治疗带状疱疹的临床观察.针灸学报 4:41.

[1252] Ye Cheng-gu , Jiang Yi. (1989)Clinical Observation on the Therapeutic Effect of Bandlike Herpes with Moxibustion. Zhen Jiu Za Zhi. 4:41.

[1253] 许荣正. (1989)不同针刺手法治疗胸腹疾病的疗效比较.针灸学报 3:26-27.

[1253] Xu Rong-zheng. (1989)Comparative study on the effects of marketers acupuncture manipulation method on chest and abdomen diseases.3:26-27.

[1254] 申倬彬. (1989)不同针法效应的对比观察.针灸学报

[1254] Shen Zhuo-bin. (1989)Comparative study on the effects of different acupuncture therapy.

Zhen Jiu Za Zhi. 4:48.

[1255] 郭秀玲, 汪秀娟 ,左立云点刺放血拔罐法治疗乳痈的疗效观察.针灸学报2:34-35.

[1255] Guo Xiu-ling，Wang Xiu-juan , Zuo Li-yun.. (1992)Observation on blood letting puncturing and cupping in the treatment of Mastitis. Zhen Jiu Za Zhi. 2:34-35.

[1256] 谢成禄. (1989)电针、TDP治疗痹证的对比观察.针灸学报1:8-9.

[1256] Xie Chen-glu. (1989)The Paired Observation of Clinical Effect of Electroacupuncture and TDP Therapentic Equipment on Arthromyodynia. Zhen Jiu Za Zhi. 1:8-9.

[1257] 包向阳, 于致顺, 葛凤新, [宋乃德](http://www.cnki.net/KCMS/detail/search.aspx?dbcode=CJFQ&sfield=au&skey=宋乃德&code=07356285;14575945;07356201;14905019;14379242;)，[张立秋](http://www.cnki.net/KCMS/detail/search.aspx?dbcode=CJFQ&sfield=au&skey=张立秋&code=07356285;14575945;07356201;14905019;14379242;). 头穴治疗偏瘫的不同疗程、刺激量与疗效的关系.针灸学报

[1257] Bao Xiang-yang, Yu Zhi-shun, Ge Feng-xin, Song Nai-de, Zhang Li-qiu. (1992) Scalp Acupuncture for Treatment of hemiparalysis patients with different courses and quantity of a cupuncture Stimulu. Zhen Jiu Za Zhi. 4:16-19.

[1258] 郝志清, 马学兰,张红. (1988)头针治疗低能儿87例疗效观察.针灸学报

[1258] Hao Zhi-qing , Ma Xue-lan, Zhang Hong. (1988)Clinical observation of Scalp Acupuncture Therapy for 87 cases with feebleminded children.Zhen Jiu Za Zhi. 1:22-23.

[1259] 祁锡玉, 赵燕茹. (1992)头针治疗室上速7例临床观察.针灸学报 6:34.

[1259] Qi Xi-yu, Zhao Yan-ru. (1992)Clinical observation of Scalp Acupuncture Therapy for 7 Cases with Supraventricular tachycardia. Zhen Jiu Za Zhi. 6:34.

[1260] 石宪, 于致顺, 孙申田. (1989)透刺传统头穴与运动区治疗中风偏瘫的比较.针灸学报.2:14-15.

[1260] Shi Xian, Yu Zhi-shun, Sun Shen-tian. (1989)Clinical Observation of Scalp Acupuncture on Apoplectic Hemiplegia. Zhen Jiu Za Zhi. 2:14-15.

[1261] 来心平. (1989)穴位药物注射与针刺治疗腕伸肌腱鞘炎疗效比较.针灸学报2:32-33.

[1261] Lai Xin-ping. (1989)Study of Different Effects between Medication Injection on Sites Plus Acupoints and acupuncture treatment with tenosynovitis of wrist extensors. Zhen Jiu Za Zhi. 2:14-15.

[1262] 吴绪平, 王亚文, 裴廷辅. (1989)一次针刺补泻百会对大学生脑血流图的影响.针灸学报4:47.

[1262] Wu Xu-ping , Wang ya-wen , Pei Ting-fu. (1989)Clinical Observation of Reinforcing and Reducing Methods of acupuncture in Baihui (GV20) for Rheoencephalography of College Students.Zhen Jiu Za Zhi. 4:47.

[1263] 孔德清, 刘孝友. (1992)针刺、药物分阶段综合治疗贝尔氏面瘫82例疗效观察.针灸学报4:12.

[1263] Kong De-qing , Liu Xiao-you.(1992)Clinical observation of acupuncture and drug treatment with Phased-in comprehensive therapy for Bell Palsy:82cases .Zhen Jiu Za Zhi. 4:12.

[1264] 裴廷辅, 刘轩, 朱旭村,[马淑英](http://www.cnki.net/KCMS/detail/search.aspx?dbcode=CJFQ&sfield=au&skey=马淑英&code=21608202;06595547;07350227;21563274;21451578;21352868;)，[张丽华](http://www.cnki.net/KCMS/detail/search.aspx?dbcode=CJFQ&sfield=au&skey=张丽华&code=21608202;06595547;07350227;21563274;21451578;21352868;)，等. (1989)针刺补泻百会对育龄妇女卵泡早期血浆中FSH、LH含量的影响.针灸学报 4:51.

[1264] Pei Ting-fu, Liu Xuan, Zhu Xu-cun, Ma Shu-ying , Zhang Li-hua, et al. (1989)Clinical Observation of Reinforcing and Reducing Methods of acupuncture in Baihui (GV20) for the plasma levels of FSH, LH in fertile woman.Zhen Jiu Za Zhi. 4:51.

[1265] 董建萍, 裴廷辅, 姚晓琳,[马淑英](http://www.cnki.net/KCMS/detail/search.aspx?dbcode=CJFQ&sfield=au&skey=马淑英&code=21608202;06595547;07350227;21563274;21451578;21352868;)，[张丽华](http://www.cnki.net/KCMS/detail/search.aspx?dbcode=CJFQ&sfield=au&skey=张丽华&code=21608202;06595547;07350227;21563274;21451578;21352868;)，等. (1989)针刺补泻百会对育龄妇女卵泡早期血浆中血栓素和前列环素的影响. 针灸学报 4:46.

[1265]Dong Jian-ping, Pei Ting-fu, Yao Xiao-lin , Ma Shu-ying , Zhang Li-hua, et al. (1989)Clinical Observation of Reinforcing and Reducing Methods of acupuncture in Baihui (GV20) for the plasma levels of thromboxane (TXA2) and prostacyclin(PGI2) in fertile woman.Zhen Jiu Za Zhi. 4:46.

[1266] 康慧, 裴廷辅, 张晓彬, [王秀菊](http://www.cnki.net/KCMS/detail/search.aspx?dbcode=CJFQ&sfield=au&skey=王秀菊&code=17624857;05968944;21381186;21449803;21352868;21289047;)，[董建萍](http://www.cnki.net/KCMS/detail/search.aspx?dbcode=CJFQ&sfield=au&skey=董建萍&code=17624857;05968944;21381186;21449803;21352868;21289047;)，等. (1989)针刺补泻百会穴对学生短时记忆的影响.针灸学报.4:45-46.

[1266] Kang Hui , Pei Ting-fu, Zhang Xiao-bin, Wang Xiu-ju,Dong Jian-ping, et al. (1989)Observation of Reinforcing and Reducing Methods of acupuncture in Baihui (GV20) for students`short-term memory.Zhen Jiu Za Zhi. 4:45-46.

[1267] 张晓彬, 裴廷辅, 董建萍, [李玫玫](http://www.cnki.net/KCMS/detail/search.aspx?dbcode=CJFQ&sfield=au&skey=李玫玫&code=21524252;23003675;21349017;21449803;20715460;14866847;21352868;21365817;17624857;)，[孟丽丽](http://www.cnki.net/KCMS/detail/search.aspx?dbcode=CJFQ&sfield=au&skey=孟丽丽&code=21524252;23003675;21349017;21449803;20715460;14866847;21352868;21365817;17624857;)，等. (1989)针刺对近视眼病人视力影响的临床实验观察.针灸学报 4:38-39.

[1267] Zhang Xiao-bin, Pei Ting-fu, Dong Jian-ping, Li Mei-mei, Meng li-li, et al. (1989)Acupuncture for Myopia: Empirical observation.Zhen Jiu Za Zhi. 4:38-39.

[1268] 冀萍, 马仁海. (1989)针刺配合TDP治疗腰痛的临床观察.针灸学报 4:25.

[1268] Ji Ping, Ma Ren-hai .(1989)Clinical observation of Electroacupuncture and TDP Therapentic Equipment for lumbago.Zhen Jiu Za Zhi. 4:25.

[1269] 龙文君 ,张全明. (1991)针刺治疗急性腰扭伤238例临床对照观察.针灸学报 1:25-26.

[1269] Long Wen-jun, Zhang Quan-ming. (1991)Acupuncture for acute lumbar sprain: 238 cases observation.Zhen Jiu Za Zhi. 1:25-26.

[1270] 李会仲. (1992)针刺治疗慢性头痛168例疗效分析.针灸学报06:2-3.

[1270] Li Hui-zhong. (1992)Acupuncture for chronic headaches: 168 cases analysis. Zhen Jiu Za Zhi.06:2-3.

[1271] 原存信, 邢江淮 ,闫怀士. (1991)针灸治疗痛症的临床研究.针灸学报4:14-16.

[1271] Yuan Cun-xin, Xing Jiang-huai, Yan Huai-shi . (1991)Clinical research of Acupuncture for Pain. Zhen Jiu Za Zhi.4:14-16.

[1272] 陈何竟, 董小丽. (2008)Amie方案治疗痉挛型脑瘫患儿运动功能障碍随机对照研究.中国针灸11:797-800.

[1272] Chen He-jing, Dong Xiao-li . (2008) Randomized controlled study on AMIE methods for treatment of movement disorder in the child of convulsive cerebral palsy. Chinese Acupuncture & Moxibustion. 11:797-800.

[1273] 李艳慧, 江钢辉, 潘文宇, [陈秀芳](http://www.cnki.net/KCMS/detail/search.aspx?dbcode=CJFQ&sfield=au&skey=陈秀芳&code=06930879;06930948;00156090;06929495;06929497;06926422;14386738;)， [黄勇](http://www.cnki.net/KCMS/detail/search.aspx?dbcode=CJFQ&sfield=au&skey=黄勇&code=06930879;06930948;00156090;06929495;06929497;06926422;14386738;)，等. (2000)MRI定位围针治疗中风偏瘫的临床观察.中国针灸2:73-74.

[1273] Li Yan-hui , Jiang Gang-hui, Pan Wen-yu , Chen Xiu-fang , Huang Yong, et al. (2000)Clinical Observation on Encircling Needling for Treatment of Hemiplegia due to Apoplexy and Its Comparason with MRI. Chinese Acupuncture & Moxibustion. 2:73-74.

[1274] 张云卿. (1995)阿是穴注射治疗梨状肌综合征50例疗效观察.中国针灸 S1:23-24.

[1274] Zhang Yun-qin. (1995)Observation on acupoint injection therapy to treating 50 cases of piriformis syndrome.Chinese Acupuncture & Moxibustion. S1:23-24.

[1275] 陈国廉, 肖国民. 郑修丽. (2008)背部排罐治疗慢性疲劳综合征疗效观察.中国针灸6:405-407.

[1275] Chen Guo-lian, Xiao Guo-min, Zheng Xiu-li. (2008)Observation on therapeutic effect of multiple cupping at back-shu points on chronic fatigue syndrome. Chinese Acupuncture & Moxibustion. 6:405-407.

[1276] 张晓哲, 王茹敏, 钱军. (2009)不同疗法治疗突发性耳聋疗效观察.中国针灸7:525-528.

[1276] Zhang Xiao-zhe, Wang Ru-min, Qia Jun . (2009)Obaservation on therapeutic effects of different treatments for sudden deafness. Chinese Acupuncture & Moxibustion. 7:525-528.

[1277] 刘智斌,杨晓航. (2006)不同手法针刺足三里穴对人PBMC的STAT5信号转导途径的作用.中国针灸2:120-122.

[1277] Liu Zhi-bin ,Yang Xiao-hang. (2006)Effects of different manipulation methods of acupuncture at Zusanli (ST 36) on signal transduction pathway of STAT5 in human PBMC. Chinese Acupuncture & Moxibustion. 2:120-122.

[1278] 雷龙鸣, 吴林, 胡跃强, [罗本华](http://www.cnki.net/KCMS/detail/search.aspx?dbcode=CJFQ&sfield=au&skey=罗本华&code=23267028;06898592;06903048;06904241;23267029;06897987;23267030;14953628;)，[黄锦军](http://www.cnki.net/KCMS/detail/search.aspx?dbcode=CJFQ&sfield=au&skey=黄锦军&code=23267028;06898592;06903048;06904241;23267029;06897987;23267030;14953628;)，等. (2009)不同针刺方案对脑梗死偏瘫患者康复及病灶侧大脑中动脉平均血流速度的影响.中国针灸29:517-520.

[1278]Lei Long-ming, Wu Lin,Hu Yue-qiang , Luo Ben-hua, Huang Jin-jun, et al. (2009)Effects of different acupuncture treatment on mean blood flow velocity of middle cerebral artery on the affected side and rehabilitation of hemiparalysis caused by cerebral infarction. Chinese Acupuncture & Moxibustion. 29:517-520.

[1279] 张英. (1994)不同针灸疗法影响高血压病患者甲襞微循环的比较研究.中国针灸4:148-151.

[1279] Zhang Ying. (1994)Comparative study on the effects of different acupuncture therapy for Nailfold Microcirculation. Chinese Acupuncture & Moxibustion. 4:148-151.

[1280] 张学丽, 刘颖, 杨丽鹍，王兰. (2009)常规针刺加飞腾八法治疗黄褐斑疗效观察.中国针灸 29:455-458.

[1280] Zhang Xue-li, Liu ying, Yang Li-kun, Wang Lan. (2009)Clinical observation on effects of routine acupuncture combined with Feiteng Bafa for chloasma. Chinese Acupuncture & Moxibustion. 29:455-458.

[1281] 陈文,杨艾堂,戴美堂,付琴丽. (2009)持续牵引下电针治疗腰椎间盘突出症疗效观察.中国针灸29：967-969.

[1281] Chen Wen, Yang Ai-tang, Dai Mei-tang, Fu Qin-li. (2009)Observation on therapeutic effect of electroacupuncture under continuous traction for treatment of lumbar disc herniation. Chinese Acupuncture & Moxibustion. 29:967-969.

[1282] 张艳, 陈锋,吴松. (2007)臭氧穴位注射治疗下腰痛临床观察.中国针灸2:115-116

[1282] Zhang Yan, Chen Feng , Wu Song. (2007)Clinical observation on O3 acupoint injection for treatment of iow back pain. Chinese Acupuncture & Moxibustion. 2:115-116

[1283] 胡幼平, 李鸿, 尹重, [王友军](http://www.cnki.net/KCMS/detail/search.aspx?dbcode=CJFQ&sfield=au&skey=王友军&code=06503443;22519987;21978020;16750892;06503598;)，[罗荣](http://www.cnki.net/KCMS/detail/search.aspx?dbcode=CJFQ&sfield=au&skey=罗荣&code=06503443;22519987;21978020;16750892;06503598;). (2009)杵针治疗失眠疗效观察.中国针灸 29：365-369.

[1283] Hu You-ping, Li Hong, Yin Zhong, Wang You-jun,Luo Rong. (2009)Observation on therapeutic effect of Chuzhen therapy on insomnia. Chinese Acupuncture & Moxibustion. 29:365-369.

[1284] 练汉健, 孔令深,黄柳和. (1999)磁场电脉冲配合经络穴位治疗中风运动功能障碍的临床观察.中国针灸S1：1-3.

[1284] Lian Han-jian, Kong Ling-shen, Huang Liu-he. (1999)Observation on therapeutic effect of pulsed electromagnetic field therapy with Acupoints of Channels and Collaterals for Stroke-induced Motor Dysfunction. Chinese Acupuncture & Moxibustion. S1:1-3.

[1285] 杨军, 杨改芬,王素芳. (1999)磁极针刺配合穴位药物封闭治疗子宫肌瘤.中国针灸S1：192-193.

[1285] Yang Jun, Yang Gai-fen, Wang Su-fang. (1999)Combined Magnetic Needle and acupoint injection therapy to treat hysteromyoma.Chinese Acupuncture & Moxibustion.S1:192-193.

[1286] 翟伟, 陈俊军, 熊健, [叶赞](http://www.cnki.net/KCMS/detail/search.aspx?dbcode=CJFQ&sfield=au&skey=叶赞&code=21702249;22398152;07442419;23777390;). (2009)聪脑通络法针刺治疗小儿脑瘫疗效观察.中国针灸29：868-872.

[1286] Zhai Wei, Chen Jun-jun, Xiong Jian , Ye Zan. (2009)Observation on therapeutic effect of treatment for children with cerebral palsy by acupuncture.29:868-872.

[1287] 林廷樾. (2005)大陵、"失眠"穴治疗顽固性失眠48例疗效观察.中国针灸5：331-332.

[1287] Lin Ting-yue. (2005)Observation on therapeutic effect of acupuncture at points Daling (PC7) and "Shimian" in 48 cases of refractory insomnia. Chinese Acupuncture & Moxibustion. 5:331-332.

[1288] 李锦鸣, 崔江云,许向明. (1999)胆囊切除术后针刺促进肠功能恢复的对比观察.中国针灸1999;07.

[1288]Li Jin-ming, Cui Jiang-yun , Xu Xiang-ming. (1999)Clinical observation of acupuncture on recovery of intestinal function after cholecystectomy. Chinese Acupuncture & Moxibustion.07.

[1289] 杨桂英，姚树汉，孙云双，[韩士伦](http://www.cnki.net/KCMS/detail/search.aspx?dbcode=CJFQ&sfield=au&skey=韩士伦&code=20836080;20299929;20904765;20975360;21016549;20600748;)，[王梅花](http://www.cnki.net/KCMS/detail/search.aspx?dbcode=CJFQ&sfield=au&skey=王梅花&code=20836080;20299929;20904765;20975360;21016549;20600748;)，等. (1994)导平针灸与超短波治疗膝关节退行性骨关节炎.中国针灸（S1):394-395.

[1289] Yang Gui-ying , Yao Shu-han Sun Yun-shuang Han Shi-lun, Wang Mei-hua, et al. (1994)Observation on therapeutic effect of treating knee osteoarthritis by sultrashort wave therapy and acupuncture therapy. Chinese Acupuncture & Moxibustion.(S1):394-395.

[1290] 农友. (1994)点刺四缝穴治疗小儿发热60例.中国针灸S1：120.

[1290] Nong You. (1994)Point-Puncturing on Sifeng Point in Treating 60 Cases of fever in children. Chinese Acupuncture & Moxibustion.S1:120.

[1291] 杜宏斌,李菊艳. (1999)电磁极针治疗面瘫100例疗效观察.中国针灸S1:151-152.

[1291] Du Hong-bin, LI Ju-yan. (1999)Observation on 100 Cases of [facioplegia](../../../../D:/Dict/5.4.43.3217/resultui/app:ds:facioplegia) Treated by Magnetic Needle. Chinese Acupuncture & Moxibustion. S1:151-152.

[1292] 赵庆伟, 刘靖, 曲向东,[李伟](http://search.cnki.com.cn/Search.aspx?q=author:李伟)，[王姝](http://search.cnki.com.cn/Search.aspx?q=author:王姝) 等. (2009)微电针并刺络放血拔罐结合饮食干预治疗急性痛风性关节炎.中国针灸9:711-713.

[1292] Zhao Qing-wei , Liu Jing , Qu Xiang-dong, Li Wei, Wang Shu, et al. (2009)Observation on therapeutic effect of electroacupuncture plus blood-letting puncture and cupping combined with diet intervention for treatment of acute gouty arthritis 9:711-713.

[1293] 付怀丹，蔡国伟. (1996)电针顶中线治疗小儿遗尿40例.中国针灸

[1293] Fu Huai-dan , Cai Guo-wei. (1996)Observation on 40 Cases of pediatric enuresis Treated by Acupuncture of Ding Zhong Xian. Chinese Acupuncture & Moxibustion.7:22.

[1294] 莫飞智, 李建强, 储莉, [雷立屏](http://search.cnki.com.cn/Search.aspx?q=author:雷立屏)，[赖新生](http://search.cnki.com.cn/Search.aspx?q=author:赖新生)，等. (2000)电针对血管性痴呆患者智力及其P_(300):的影响.中国针灸 11:687-690.

[1294] Mo Fei-zhi, Li Jian-qiang, Chu Li , Lei Li-ping, Lai Xin-sheng, et al. (2000)Effects of Electroacupuncture on Intelligence and P300 in the Patient of Vascular Dementia. Chinese Acupuncture & Moxibustion. 11:687-690.

[1295] 解秸萍, 刘桂玲, 乔晋琳, [顾群](http://search.cnki.com.cn/Search.aspx?q=author:顾群)，[盖亚男](http://search.cnki.com.cn/Search.aspx?q=author:盖亚男)，等. (2009)电针丰隆穴调节血脂的多中心随机对照研究.中国针灸 5:345-348.

[1295] Xie Jie-ping, Liu Gui-ling,Qiao Jin-lin , Gu Qun, Gai Ya-nan, et al. (2009)Multi-central randomized controlled study on electroacupuncture at Fenglong(ST 40)for regulating blood lipids. Chinese Acupuncture & Moxibustion. 5:345-348.

[1296] 刘丽军. (1995)电针加拔罐治疗肌纤维织炎40例.中国针灸 2:20.

[1296] Liu Li-jun. (1995)Observation on 40 Cases of myositis fibrosa Treated by [cupping](../../../../D:/Dict/5.4.43.3217/resultui/app:ds:cupping) [therapy](../../../../D:/Dict/5.4.43.3217/resultui/app:ds:therapy) with electric acupuncture. Chinese Acupuncture & Moxibustion.2:20

[1297] 张辉,高岚. (2004)电针加灸治疗肱骨外上髁炎35例.中国针灸 S1:21-22.

[1297] Zhang hui, Gao Lan. (2004)Clinical observation of the electricity needle plus to moxibustion to treat the lateral epicondylitis:48 cases . Chinese Acupuncture & Moxibustion. S1:21-22.

[1298] 高岚,张辉,肖雷. (2005)电针加牵引治疗神经根型颈椎病40例.中国针灸

[1298] Gao Lan, Zhang hui, Xiao Lei. (2005)Observation on 40 Cases of Nerve Root Cervical Spondylosis Treated by Traction Combined with electric acupuncture. Chinese Acupuncture & Moxibustion. S1:83-84.

[1299]来明. (2000)电针加穴位注射治疗颈性眩晕55例.中国针灸2000;S1:141-142.

[1299] Lai Ming. (2000)Combined electro- acupuncture and acupoint injection therapy to treating 55 cases of cervical vertigo.Chinese Acupuncture & Moxibustion. S1:141-142.

[1300] 张冬梅. (2002)电针加穴位注射治疗外伤性面瘫.中国针灸S1:56.

[1300]Zhang Dong-mei . (2002)Combined electro- acupuncture and acupoint injection therapy to treat traumatic facial palsy.Chinese Acupuncture & Moxibustion. S1:56.

[1301] 刘银妮, 张红星, 黄国付,[邹燃](http://search.cnki.com.cn/Search.aspx?q=author:邹燃)，[魏巍](http://search.cnki.com.cn/Search.aspx?q=author:魏巍). (2009)电针夹脊穴配合放血拔罐治疗带状疱疹疗效观察.中国针灸 11:887-890.

[1301] Liu Yin-ni , Zhang Hong-xing , Huang Guo-fu , Zou Ran, Wei Wei. (2009)Observation on therapeutic effect of electroacupuncture at Jiaji(EX-B 2)combined with blood-letting and cupping on herpes zoster. Chinese Acupuncture & Moxibustion. 11:887-890.

[1302] 曾才鑫, 李烦兵,彭华莎. (1999)电针夹脊穴治疗脊椎骨质增生60例疗效观察.中国针灸 3:153-154.

[1302] Zeng Cai-xin, LI Fan-bin, Peng Hua-sha. (1999)Observation on therapeutic effect of electroacupuncture at Jiaji(EX-B 2) on [osteoproliferation](../../../../D:/Dict/5.4.43.3217/resultui/app:ds:osteoproliferation). Chinese Acupuncture & Moxibustion. 3:153-154.

[1303] 金孟梓. (2000)电针配刺络治疗偏头痛32例临床观察.中国针灸 8:471-472.

[1303] Jin Meng-zi . (2000)Clinical observation on Migraine treated by Electro-acupuncture plus Blood-letting therapy:32 cases. Chinese Acupuncture & Moxibustion. 8:471-472.

[1304] 唐春林, 戴德纯, 赵桂凤, [朱伟芳](http://search.cnki.com.cn/Search.aspx?q=author:朱伟芳)，[梅麟凤](http://search.cnki.com.cn/Search.aspx?q=author:梅麟凤). (2009)电针配合穴位埋线治疗心脾两虚型单纯性肥胖临床观察.中国针灸 9:703-707.

[1304] Tang Chun-lin, Dai De-chun Zhao Gui-feng, Zhu wei-fang, Mei Lin-feng. (2009)Clinical observation on electroacupuncture combined with catgut implantation at acupoints for treatment of simple obesity of heart and spleen deficiency type. Chinese Acupuncture & Moxibustion. 9:703-707.

[1305] 万文俊, 马朝阳, 熊修安,[王鹂](http://search.cnki.com.cn/Search.aspx?q=author:王鹂),[丁琳](http://search.cnki.com.cn/Search.aspx?q=author:丁琳),等. (2009)电针曲池穴治疗原发性高血压病疗效观察及机制探讨.中国针灸5:349-352.

[1305] Wan Wen-jun ,Ma zhao-yang , Xiong Xiu-an, Wang Peng, Ding Lin, et al. (2009)Clinical observation on therapeutic effect of electroacupuncture at Quchi(LI 11)for treatment of essential hypertension. Chinese Acupuncture & Moxibustion. 5:349-352.

[1306] 周军, 秦正玉, 李伟莉,[张翠萍](http://search.cnki.com.cn/Search.aspx?q=author:张翠萍)，[田丽颖](http://search.cnki.com.cn/Search.aspx?q=author:田丽颖)，等. (2006)电针三阴交治疗围绝经期综合征临床观察.中国针灸9:617-620.

[1306] Zhou Jun, Qin Zheng-yu,Li wei-li ,Zhang Cui-ping,Tian Li-ying, et al. (2006) Tian LiyingClinical observation on therapeutic effect of electroacupuncture at Sanyinjiao (SP 6) on peri-menopausal syndrome. Chinese Acupuncture & Moxibustion. 9:617-620.

[1307] 王洪峰, 王富春, 王健,[张二力](http://search.cnki.com.cn/Search.aspx?q=author:张二力)，[东贵荣](http://search.cnki.com.cn/Search.aspx?q=author:东贵荣)，等. (2004)电针五脏俞治疗急性格林巴利综合征临床观察.中国针灸12:823-824.

[1307] Wang Hong-feng , Wang fu-chun, Wang Jian, Zhang Er-li, Dong Gui-rong, et al. (2004)Clinical observation on electroacupuncture at shu-points of the five zang-organs for treatment of acute Guillain-Barre syndrome. Chinese Acupuncture & Moxibustion. 12:823-824.

[1308] 司全明, 吴根诚,曹小定. (1999)电针治疗急性脑梗塞患者临床疗效观察.中国针灸3:137-139.

[1308] Si Quan-ming, Wu Gen-cheng, Cao Xiao-ding. (1999)Clinical observation on [acute](../../../../D:/Dict/5.4.43.3217/resultui/app:ds:acute) [cerebral](../../../../D:/Dict/5.4.43.3217/resultui/app:ds:cerebral) [infarction](../../../../D:/Dict/5.4.43.3217/resultui/app:ds:infarction) treated with electrotherapy . Chinese Acupuncture & Moxibustion. 3:137-139.

[1309] 蒋时习,丁正蓉. (2004)电针治疗肩周炎100例疗效观察.中国针灸S1:19.

[1309] Jiang Shi-xi, Ding Zheng-rong. (2004)Clinical observation on periarthritis of shoulder treated with electrotherapy:100 cases. Chinese Acupuncture & Moxibustion. S1:19.

[1310] 张杰, 刘朝东, 丁燕, [唐驱波](http://search.cnki.com.cn/Search.aspx?q=author:唐驱波). (2010)电针治疗慢性前列腺炎疗效观察及对患者尿道括约肌肌电图的影响.中国针灸1:13-17.

[1310] Zhang jie, Liu Chao-dong ,Ding Yan, Tang Qu-bo. (2010)Clinical observation On therapeutic effect of electroacupuncture on chronic prostatitis and detection of urethral sphincter EMG. Chinese Acupuncture & Moxibustion. 1:13-17.

[1311] 杨焱, 张越, 景年才,[卢义](http://search.cnki.com.cn/Search.aspx?q=author:卢义)，[肖宏宇](http://search.cnki.com.cn/Search.aspx?q=author:肖宏宇)，等. (2009)电针足三里穴治疗恶性肿瘤化疗所致恶心呕吐多中心随机对照研究.中国针灸12:955-958.

[1311] Yang Yan , Zhang Yue, Jin Nian-cai, Lu Yi, Xiao Hong-yu, et al. (2009)Electroacupuncture at Zusanli(ST 36)for treatment of nausea and vomiting caused by the chemotherapy of the malignant tumor:a multicentral randomized controlled trial. Chinese Acupuncture & Moxibustion. 12:955-958.

[1312] 张智龙, 吉学群, 赵淑华, [张景江](http://search.cnki.com.cn/Search.aspx?q=author:张景江)，[康涛](http://search.cnki.com.cn/Search.aspx?q=author:康涛)，等. (2008)调理脾胃针法治疗2型糖尿病合并冠心病随机对照研究.中国针灸9:629-633.

[1312] Zhang Zhi-long , Ji Xue-qun , Zhao Shu-hua, Zhang Jing-jiang , Kang Tao, et al. (2008)Randomized controlled study on effects of the needling method for regulating spleen-stomach on coronary heart disease complicated by type 2 diabetes mellitns complicated. Chinese Acupuncture & Moxibustion. 9:629-633.

[1313] 张萍, 刘占芬, 王春梅,等. (2007)调理脾胃针法治疗糖尿病胃轻瘫疗效观察.中国针灸3:165-168.

[1313] Zhang ping, Liu Zhan-fen, Wang Chun-mei, et al. (2007)Observation on the therapeutic effect of needling method for harmonizing spleen-stomach on diabetic gastroparesis. Chinese Acupuncture & Moxibustion. 3:165-168.

[1314] 李英. (1994)针刺治疗在痛证中的应用.中国针灸S1:278-279.

[1314] Li Ying. (1994)Acupuncture for relieving Pain. Chinese Acupuncture & Moxibustion. S1:278-279.8:504,.

[1315] 陈礼娇，田丽. (1997)对头针齐刺法与传统针刺疗法治疗肌萎缩178例疗效观察.中国针灸

[1315] Chen Li-jiao, Tian Li. (1997)Comparative study on the effects of Ping Qi acupuncture needles and [traditional](../../../../D:/Dict/5.4.43.3217/resultui/app:ds:traditional) acupuncture for [amyotrophy](../../../../D:/Dict/5.4.43.3217/resultui/app:ds:amyotrophy):178 cases. Chinese Acupuncture & Moxibustion.8:504.

[1316] 朱沁. (2007)多针浅刺为主对肥胖症患者腹围及体重的影响.中国针灸 S1:5-7.

[1316] Zhu Qin. (2007)Effects of multiple-superficial needling treatment on waist circumference and body mass index in obese patients. Chinese Acupuncture & Moxibustion. S1:5-7.

[1317] 吴波, 蒋存火, 周群英,[陈前明](http://search.cnki.com.cn/Search.aspx?q=author:陈前明)，[树瑜](http://search.cnki.com.cn/Search.aspx?q=author:树瑜)，等. (2007)耳穴贴压合穴位注射治疗带状疱疹后遗神经痛.中国针灸 11:807-809.

[1317] Wu Bo, Jiang Cun-huo, Zhou Qun-ying, Chen Qian-ming, Shu Yu, et al. (2007)Treatment of residual neuralgia of herpes zoster by ear point taping and pressing therapy combined with acupoint-injection. Chinese Acupuncture & Moxibustion. 11:807-809.

[1318] 龙昭贤. (2006)耳穴贴压中晚期妊娠引产200例.中国针灸 10:687-690.

[1318] Long Zhao-xian. (2006)Observation on the application of ear point taping and pressing therapy in induced abortion of pregnancy with complications in the second and third trimester:200 cases. Chinese Acupuncture & Moxibustion. 10:687-690.

[1319] 单秋华, 杨佃会, 贾真,[韩晶](http://med.wanfangdata.com.cn/Author/info/A000648606.aspx) ，[张彤](http://med.wanfangdata.com.cn/Author/info/A000225037.aspx)，等. (2006)耳穴综合疗法治疗发作期普通偏头痛疗效评价.中国针灸 10:687-690.

[1319]Shan Qiu-hua , Yang dain-hui, Jia Zhen, Han Jing, Zhang tong, et al. (2006)Evaluation of therapeutic effects of synthetic auricular point therapy for treatment of common migraine at the attack stage. Chinese Acupuncture & Moxibustion. 10:687-690.

[1320] 谢潇侠. (1994)耳压法与体针疗法对失眠疗效的对比观察.中国针灸S1:300.

[1320] Xie Xiao-xia. (1994)Comparative study of insomnia treated by ear point taping and acupuncture-therapy. Chinese Acupuncture & Moxibustion.S1:300.

[1321] 华英,杨金凤. (1999)分期针刺治疗周围性面瘫72例疗效分析.中国针灸7:394.

[1321]Hua Ying, Yang Jin-feng. (1999)Staging Acupuncture for peripheral facial paralysis: 72 cases observation. Chinese Acupuncture & Moxibustion.7:394.

[1322] 查和萍, 熊艳红,黄伟昌浮针治疗顽固性网球肘疗效观察.中国针灸 9:611-612

[1322]Zha He-ping, Xiong Yan-hong ,Huang Wei-chang. (2004)Therapeutic effects of superficial Needling and block therapy on obstinate tennis elbow. Chinese Acupuncture & Moxibustion. 9:611-612

[1323] 王新宇. (2007)改良穴位配合康复训练对脑梗死患者上肢活动能力及日常生活能力的影响.中国针灸 3:179-181.

[1323] Wang Xin-yu. (2007)Effects of the improved acupoints and rehabilitation exercise on locomotor ability of the upper limbs and ability of daily life in the patient of cerebral infarction. Chinese Acupuncture & Moxibustion. 3:179-181.

[1324] 徐放明, 谢鹏, 吕发金, [牟君](http://search.cnki.com.cn/Search.aspx?q=author:牟君) ，[李咏梅](http://search.cnki.com.cn/Search.aspx?q=author:李咏梅)，等. (2007)肝经、肺经脑内分布区域的fMRI研究.中国针灸10:749-752.

[1324]Fang Ming, Xie Peng, Lv Fa-jing, Mou Jun, LI Yong mei, et al. (2007)Study on Corresponding areas the Liver and Lung Channels in brain with fMRI. Chinese Acupuncture & Moxibustion. 10:749-752.

[1325] 陈兴华, 姚文敏, 邹春萍,[徐涵斌](http://search.cnki.com.cn/Search.aspx?q=author:徐涵斌)，等. (2009)合谷刺配合化脓灸治疗类风湿性关节炎活动期疗效观察.中国针灸 11:884-886.

[1325] Chen Xing-hua, Yao Wen-min,Zou Chun-ping , Xu Han-bin, et al. (2009)Observation on therapeutic effect of muscular needling combined with scarring moxibustion on active stage of rheumatoid arthritis. Chinese Acupuncture & Moxibustion.11:884-886.

[1326] 王麟鹏, 刘慧林, 刘志顺,[赵吉平](http://search.cnki.com.cn/Search.aspx?q=author:赵吉平)，[赵因](http://search.cnki.com.cn/Search.aspx?q=author:赵因)，等. (2006)贺氏三通法对缺血性中风患者神经功能缺损的影响多中心随机对照研究.中国针灸 5:309-312.

[1326] Wang Lin-peng, Liu Hui-lin , Liu Zhi-shun,, Zhao Ji-ping , Zhao Yin, et al. (2006)He's Santong needling method for neurological functional deficit in the patient of ischemic apoplexy: randomized controlled multi-central study. Chinese Acupuncture & Moxibustion.5:309-312.

[1327] 李和. (2009)贺氏三通法治疗寒冷性多形红斑疗效观察.中国针灸 10:807-809.

[1327] LI He. (2009)Observation on therapeutic effect of He's Santong needling methods on cold erythema multiforme. Chinese Acupuncture & Moxibustion.10:807-809.

[1328] 王桂玲, 李华岳, 谢新才,[贺普仁](http://www.cnki.net/KCMS/detail/search.aspx?dbcode=CJFQ&sfield=au&skey=贺普仁&code=22863036;22793048;22940337;22760134;). (2008)贺氏三通法治疗失眠临床观察.中国针灸S1:31-32.

[1328] Wang Gui-ling, Li Hua-yue, Xie Xin-cai, He Pu-ren. (2008)Effect of Treatment for insomnia by means of He's Therapy of San Tong. Chinese Acupuncture & Moxibustion.S1:31-32.

[1329] 卢爱军, 庞爱军, 修文福,[王志刚](http://search.cnki.com.cn/Search.aspx?q=author:王志刚)，[马小顺](http://search.cnki.com.cn/Search.aspx?q=author:马小顺)，等.(2007)缓控针植入穴位治疗功能性室性早搏临床观察.中国针灸 10:721-724.

[1329] Lu Ai-jun ,Pang Ai-jun ,Xiu Wen-fu, Wang Zhi-gang, Ma Xiao-shun, et al. (2007)Clinical observation on point implantation of slowly-releasing medication for treatment of functional ventricular premature. Chinese Acupuncture & Moxibustion. 10:721-724.

[1330] 杨丽艳, 卢得健,李艳慧. (2009)火针治疗腰椎间盘突出症疗效观察.中国针灸6:449-451.

[1330]Yang Li-yan ,Lu De-jian, Li Yan-hui. (2009)Observation on therapeutic effect of fire-needle therapy on lumbar intervertebral disc herniation. Chinese Acupuncture & Moxibustion. 6:449-451.

[1331] 黄元芳, 王泰芬, 刘艳, [张世兴](http://med.wanfangdata.com.cn/Author/info/张世兴.aspx) . (2008)极泉穴不同操作对神经根型颈椎病疗效的影响.中国针灸6:427-428.

[1331] Huang Yuan-fang, Wang Tai-fen, Liu Yan, Zhang shi-xing . (2008)Clinical observation on Jiquan (HT 1) for treatment of cervical spondylosis of nerve root type. Chinese Acupuncture & Moxibustion. 6:427-428.

[1332] 王建瑞. (1994)集刺法与针刺法治疗腰腿痛疗效观察.中国针灸,

[1332] Wang Jian-rui. (1994)Control study of therapeutic effects of multiple needling plus

and routine acupuncture method on low back pain . Chinese Acupuncture&Moxibustion, S1:78-79

[1333] 张德斌, 周彩霞,刘凌夹. (2007)脊穴温针灸治疗慢性疲劳综合征疗效观察.中国针灸S1:61-62.

[1333] Zhang De-bin , Zhou Cai-xia , Liu Lin-jia.. (2007)Observation on therapeutic effect of Warming Acupuncture of Jiaji Acupoints for Chronic Fatigue Syndrome. Chinese Acupuncture & Moxibustion. S1:61-62.

[1334] 徐世芬, 庄礼兴, 贾超,[陈兴华](http://search.cnki.com.cn/Search.aspx?q=author:陈兴华)，[吴思平](http://search.cnki.com.cn/Search.aspx?q=author:吴思平)，等. (2009)靳三针”对脑卒中偏瘫患者认知功能和日常生活能力的影响多中心随机对照研究.中国针灸 9:689-694.

[1334] Xu Shi-fen, Zhuang li-xing, Jia Chao, Chen XIng-hua, Wu Si-ping, et al. (2009)Effect of"Jin three-needle therapy"on cognitive function and activity of daily living in patients of hemiplegia after stroke:a multi-central randomized controlled study. Chinese Acupuncture & Moxibustion. 9:689-694.

[1335] 刘正口. (2000)三针治疗肩周炎38例临床观察.中国针灸 S1:34-35.

[1335] Liu Zheng-kou. (2000)Acupuncturing three different acupoints for Peripheral facial paralysis: 168 cases observation. Chinese Acupuncture & Moxibustion. S1:34-35.

[1336] 谢感共, 陈靖红.赵彩娇. (2007)灵龟八法治疗原发性痛经的疗效观察.中国针灸S1:65-66.

[1336] Xie Gan-gond, Chen Jing-hong , Zhao Cai-jiao. (2007)Clinical study on primary dysmenorrheal treated with eight methods of intelligentturtle. Chinese Acupuncture & Moxibustion. S1:65-66.

[1337] 苏秀海, 王晓蕴, 王元松, [李烨](http://search.cnki.com.cn/Search.aspx?q=author:李烨) . (2007)弥可保穴位注射治疗糖尿病周围神经病变疗效观察.中国针灸12:801-802.

[1337] Su Xiu-hai, Wang Xiao-yun, Wang Yuan-song, Li Ye. (2007)Observation on Therapeutic Effect of Acupoint-Injection of Mikebao on Diabetic Peripheral Neuropathies. Chinese Acupuncture & Moxibustion. 12:801-802.

[1338] 熊灿东. (2002)面神经炎急性期针刺的疗效观察.中国针灸 11:743.

[1338] Xiong Can-dong. (2002)Observation on Therapeutic Effect of Acupuncture on Facial Neuritis at Acute Stage. Chinese Acupuncture & Moxibustion. 11:743.

[1339] 朱红霞, 徐艳洁, 孟素峰, [冯琥](http://search.cnki.com.cn/Search.aspx?q=author:冯琥)，[刘苑](http://search.cnki.com.cn/Search.aspx?q=author:刘苑). (2010)内关穴注射预防妇科腹腔镜手术后恶心呕吐.中国针灸1:72-74.

[1339] Zhu Hong-xia , Xu Yan-jie, Meng Su-feng, Feng Hu, Liu Yuan. (2010)Preventive effect of acupoint injection at Neiguan(PC 6)on postoperative nausea and vomiting after laparoscopic gynecologic surgery. Chinese Acupuncture & Moxibustion. 1:72-74.

[1340] 朝励兵. (2005)偏头痛的针刺治疗回顾.中国针灸S1:18-19.
[truncated: 190,978 more chars]
